# NMR-based metabolic profiling of urine, serum, fecal, and pancreatic tissue samples from the Ptf1a-Cre; LSL-KrasG12D transgenic mouse model of pancreatic cancer

**DOI:** 10.1371/journal.pone.0200658

**Published:** 2018-07-17

**Authors:** Michelle J. Schmahl, Daniel P. Regan, Adam C. Rivers, William C. Joesten, Michael A. Kennedy

**Affiliations:** Department of Chemistry & Biochemistry, Miami University, Oxford, Ohio, United States of America; Vrije Universiteit Brussel, BELGIUM

## Abstract

Pancreatic cancer is the third leading cause of cancer deaths in the United States with more than 53,000 expected to be diagnosed with the disease in 2018. The median survival time after diagnosis is four to six months. The poor survival statistics are due in part to the fact that pancreatic cancer is typically asymptomatic until it reaches advanced stages of the disease. Although surgical resection provides the best chance of survival, pancreatic cancer is rarely detected when surgery is still possible due, in part, to lack of effective biomarkers for early detection. The goal of the research reported here was to determine if it was possible to identify metabolic biomarkers for detection of pre-cancerous pancreatic intraepithelial neoplasia (PanIN) that precede pancreatic adenocarcinoma. The transgenic Ptf1a-Cre; LSL-KrasG12D mouse strain was used as a model of pancreatic cancer progression. Nuclear magnetic resonance (NMR) spectroscopy was employed to compare metabolic profiles of urine, sera, fecal extracts, and pancreatic tissue extracts collected from control and study mice aged 5, 11, and 15 months, including 47 mice with tumors. We were able to identify the following potential biomarkers: decreased 3-indoxylsulfate, benzoate and citrate in urine, decreased glucose, choline, and lactate in blood, and decreased phenylalanine and benzoate and increased acetoin in fecal extracts. Potential biomarkers were validated by p-values, PLS-DA VIP scores, and accuracies based on area under ROC curve analyses. Essentially, all of the metabolic profiling changes could be explained as being associated with the consequences of bicarbonate wasting caused by a complete substitution of the normal pancreatic acinar tissue by tissue entirely composed of PanIN. Given the nature of the mouse model used here, our results indicate that it may be possible to use NMR-based metabolic profiling to identify biomarkers for detection of precancerous PanIN that immediately precede pancreatic cancer.

## Introduction

Pancreatic cancer is the third leading cause of cancer related deaths in the United States with a median survival time between four to six months and a five-year survival of about nine percent [1–8]. Despite great advances in cancer treatment therapies, physicians and researchers remain unable to detect pancreatic ductal adenocarcinoma (PDAC) in patients until the disease has progressed to advanced stages. Pancreatic cancer occurs in exocrine tissue in nearly 95% of all patients [9] and is usually asymptomatic until advanced stages of the disease [10, 11]. Surgical resection of the tumor is rarely effective, although it provides the greatest chance of survival. In the rare cases when the detected cancer is localized to the pancreas prior to metastasis, removal of the tumor by resection can increase the 5-year survival rate to 25%–40% [12, 13]. Unfortunately, pancreatic cancer is usually advanced to the point that resection is not possible at the time of diagnosis, which is the case in ~85% of patients [6, 14]. Current diagnostic methods are extremely invasive and no tests for early detection and diagnosis of pancreatic cancer are dependable at this time [14, 15].

Metabolic profiling, also referred to as “metabonomics” [16], is still an emerging field of research that can potentially be used to identify new metabolic biomarkers for early detection of pancreatic cancer, and possibly even to detect precancerous PanIN. Metabonomics is based on quantification and comparison of metabolite concentrations in biological samples, including urine, blood, fecal extracts and tissue extracts, among others [17–27]. Comparison of metabolic profiles of control and study populations can lead to the identification of potential biomarkers for disease. Multiple signaling networks have been implicated in pancreatic cancer [28], which underlies the potential for finding metabolic biomarkers associated with pancreatic cancer. Metabonomics has been used to search for novel metabolites in serum samples of patients diagnosed with pancreatic cancer [29]. In that study, sera samples from healthy patients, patients with pancreatic cancer, and patients with chronic pancreatitis could be distinguished using metabolic profiling. Urine and fecal extracts have also been shown to contain potentially useful biomarkers for detection of pancreatic cancer [30].

In this study, NMR based metabonomics has been used to explore the potential of metabolic profiling for identification of potential biomarkers for detection of precancerous pancreatic intraepithelial neoplasia (PanIN) that precede pancreatic cancer [4, 31–33]. The transgenic Ptf1a-Cre; LSL-KrasG12D mouse model used in this study has been extensively characterized and shown to display extensive PanIN that eventually progress to PDAC [4, 31–33]. The Ptf1a-Cre; LSL-KrasG12D mouse model of pancreatic cancer is considered to recapitulate important characteristics of human pancreatic cancer, including spontaneous pancreatic tumor formation that is preceded by a precancerous PanIN stage [4, 31, 33]. Another common feature is that mutations of the Kras gene are found in > 90% of human pancreatic adenocarcinoma tumors [34–36] as well as in > 95% of PanINs have been reported to have Kras mutations [37] and the Ptf1a-Cre; LSL-KrasG12D mouse model was constructed to cause activation of PanIN initiation and pancreatic tumor formation based on the introduction of the G12D Kras mutation with activation restricted to expression in the pancreatic tissue [4], presumably mimicking spontaneous somatic cell Kras mutations that occur in the initiation of human pancreatic cancer [28, 37]. Based on the genetic alterations common to both human pancreatic cancer and in the Ptf1a-Cre; LSL-KrasG12D mouse model, and based on the common progression through a precancerous PanIN phase that proceeds to prancreatic adenocarcinoma, it is commonly assumed that the Ptf1a-Cre; LSL-KrasG12D mouse strain is a good model for what is observed in the human clinical scenario. Here, the Ptf1a-Cre; LSL-KrasG12D mouse model was used to enable NMR-based metabolic profiling of urine, fecal, serum, and pancreatic tissue samples from healthy mice and mice in different PanIN stages, plus 47 mice with pancreatic tumors. Changes in the metabolite concentrations detected in each biological sample group are listed, validated, interpreted, and discussed. The accuracy of biomarkers from urine, serum and fecal extract samples for detection of precancerous PanIN and pancreatic tumors is also discussed.

## Materials and methods

### Mouse care and use

The procedures described below and carried out in this study have been approved by the Institutional Animal Care and Use Committee (IACUC) at Miami University through protocol numbers: 854 and 855. The protocols were approved by both the ethics committee and IACUC of Miami University (Animal Welfare Assurance Number: D16-00100). All procedures and dissections were conducted as previously described [32].

In-house breeding and experiments were conducted over a period of 36 months to enable generation of the number of mice needed for each age category. Initial breeding mice were acquired from the Jackson Laboratory (https://www.jax.org/strain/000664). After mice were anesthetized using isoflurane, blood samples were collected using a terminal heart puncture procedure followed by cervical dislocation to ensure euthanasia. Sacrifice dates were established for each mouse based on date of birth and fulfillment of age categories from 5 to 16 months. Mice determined to be too sick to continue in the study prior to their established sacrifice dates were considered to have reached a humane endpoint and were immediately sacrificed to minimize suffering and discomfort. A mouse was considered to have reached a humane endpoint when it displayed abnormal inactivity, failure to intake food or water, severe increase in body weight due to a large tumor, discomfort, excretion of diarrhea-like discharge from the anal region, or displaying any abnormal features like exhibiting a dome head characteristic, blindness, or malocclusion. Daily health monitoring of mice was conducted by the Miami University’s Animal Resources and Care Facility. Researchers were notified if immediate action needed to be taken. Out of 1024 mice included in the study (as described below), 14 mice were euthanized prior to their established sacrifice dates and 44 mice were found dead due to quick progression of the disease or other complications.

### Mice breeding

Breeding pairs were established based on genotyping results described below. The KrasG12D mouse strain, B6.129-Kras<tm4Tyj>, contained a mutated Kras gene encoding G12D-Kras silenced by a Lox-Stop-Lox cassette. The Cre mouse strain, B6.Ptf1a(tm1.1(cre)Cvw), contained cre-recombinase introduced by a cre knockin at the Ptf1a-p48 locus, that is primarily expressed in the pancreas [4]. Crossbreeding KrasG12D mice with Ptf1a-Cre mice resulted offspring that statistically consisted of 25% control mice (Ptf1a^Cre/-^;LSL-Kras^G12D/-^), 25% study mice (Ptf1a^Cre/+^;LSL-Kras^G12D/+^), and 50% of the mice that carried one of the two genes (Ptf1a^Cre/-^;LSL-Kras^G12D/+^ and Ptf1a^Cre/+^;LSL-Kras^G12D/-^). Mice were continuously bred until there were groups of 24 mice from both genders and at every month from 5 to 16 months of age to ensure sufficient statistical power for metabolic profiling analysis.

### Mouse genotyping

Ear punches were used to identify mice. Mice were restrained prior to ear clipping and ear clippings were carried out using an animal ear-tag punch (Fisher Scientific, Hampton, NH). Tissue obtained from ear punches was used to extract DNA for PCR-based genotyping of control and study mice. Tissue from the ear punches of the mice were stored at -80°C until performing the DNA extraction procedure. To extract DNA from the ear punch tissue samples, the tissue samples were transferred into a PCR tube, 25 μL of 25 mM NaOH/0.2 mM EDTA solution was added to the tube, and then tubes were placed in the Gene Amp PCR System 9700 thermocycler at 94.0°C for an hour to release the DNA, and then the temperature was returned to, and held at, 4°C when finished. After the DNA extraction cycle was complete, 25 μL mM of Tris-HCl solution was added to neutralize the pH in preparation for PCR amplification. A control PCR test was performed to ensure that DNA was present in the solution. After confirmation of the presence of DNA, the PCR-based genotyping procedure was conducted. The Kras and Cre primers were used according to the manufacturer’s protocols (The Jackson Laboratory, Bar Harbor, ME) to probe for the presence of the Kras or Cre genes using Gene Amp PCR System 9700 thermocycler. PCR amplification products were analyzed using agarose gel electrophoresis to identify the presence or absence of mice containing the Kras mutation, Cre-recombinase, and wildtype mice lacking both gene elements. An AlphaImager (Alpha Innotech, San Leandro, CA) was used to visualize the amplified DNA on agarose gels stained with ethidium bromide.

### Histological analysis of tissue sections

Mice pancreata were examined histologically to allow comparison of normal tissue in the control mice with the presence and abundance of PanIN and tumor burden in the study mice. Following dissection, pancreata were stored in formalin overnight and transferred to 70% ethanol. Tissues were processed using a Leica TP 1020 benchtop tissue processer (Leica Biosystems, Buffalo Grove, IL, USA) and embedded using a Shandon Histocenter (Thermo Fisher Scientific, Waltham, MA). Tissues were processed into 5 μm sections using a Thermo-Shandon Finesse ME Microtome (Thermo Fisher Scientific, Waltham, MA). Tissue sections were stained with hematoxylin and eosin and images were taken using an Olympus AX70 Light Microscope (Olympus, Tokyo, Japan) in the Center for Advanced Microscopy and Imaging at Miami University.

### Preparation of urine samples for metabolic profiling

Urine samples were collected using custom-built metabolism cages [17] designed to minimize cross contamination of urine and feces. The metabolism cages contained a metal base that supported the mouse and an inverted sink strainer enclosed in a funnel below. When urine is released, it flowed through the strainer and was collected into a container below containing mineral oil, while the fecal samples fell to the sides of the inverted strainer. After 12 hours of collection, the urine samples were separated from the mineral oil by pipet, placed into centrifuge tubes, and stored at -80°C. The samples were thawed and adjusted to pH 7.4 followed by centrifugation at 10000 x g for 10 minutes at 4°C. 540 μL of processed urine was transferred into new centrifuge tubes with 60 μL D_2_O and 66 μL of PBS buffer containing 0.1% w/v sodium azide and 1 mM trimethylsilylpropanoic acid (TSP). The mixture was centrifuged again at 10000 x g for 10 minutes at 4°C and then transferred to a 5 mm NMR tube for NMR analysis.

### Preparation of serum samples for metabolic profiling

Serum samples were collected from female control and study mice from ages 5-, 11-, and 15-months. After mice were anesthetized using isoflurane, blood was collected from each mouse at the time of pancreas removal with a 21G syringe via heart puncture [32]. Blood was placed in an untreated centrifuge tube after removal and allowed to coagulate for 30 minutes. The coagulated blood samples were then centrifuged to achieve separation and collection of sera. Sera samples were stored at -80°C until further use. In preparation for NMR analysis, sera were thawed on ice and passed through a 3K filter (Thermo Fisher Scientific, Waltham, MA USA) to remove protein and other larger molecular weight components. D_2_O containing PBS buffer and 0.58 mM TSP as a chemical shift reference and concentration standard were added to sera samples and the final prepared sera samples were placed into 3 mm NMR tubes for NMR analysis.

### Preparation of fecal extracts for metabolic profiling

The previously described metabolism cages were used to collect fecal samples from study and control mice. Fecal samples were collected using sterilized forceps, placed into centrifuge tubes, and stored at -80°C until processed. For each sample, 0.14 g of fecal sample was added to a 1:7 (fecal to water) ratio of deionized water and vortexed for 10 minutes. The mixtures were centrifuged for 10 minutes at 15,000 x g at 4°C. Supernatants were transferred to new centrifuge tubes by pipette, pH adjusted to 7.4, and centrifuged again. 540 μl of fecal extract supernatant were removed and added to 60 μL D2O and 66 μL of PBS buffer, containing 0.1% w/v sodium azide and 1 mM TSP. The pH was measured again and, if needed, adjusted to 7.4 then centrifuged again. 600 μl of final process sample was transferred to a 5 mm NMR tube for NMR analysis.

### Preparation of pancreatic tissues and pancreatic tumors for metabolic profiling

After pancreas removal at the time of dissection, a 0.14 g section was taken, placed into a centrifuge tube and snap frozen in liquid nitrogen. Tissues were stored at -80°C until further use. In preparation for NMR analysis, tissues were transferred into tubes prefilled with ceramic beads (Precellys, Ann Arbor, MI) and 4 ml/g cold methanol and 0.85ml/g cold deionized water was added to the tube. The tubes were placed in the Precellys 24 Lysis and Homogenization system (Precellys, Ann Arbor, MI) with an attached Cryolys temperature controller and samples were homogenized at 6400 rpm for 20 seconds separated by a 30 second break. Homogenates were transferred into new centrifuge tubes and 4 mL/g chloroform and 4.4 mL/g deionized water added. Mixtures were vortexed for 60 seconds and set on ice for 10 minutes to allow partitioning. Samples were centrifuged for 20 minutes at 17,949 x g allowing separation of hydrophilic (top) and lipophilic (bottom) layers separated by a precipitated protein layer. Hydrophilic and lipophilic layers were isolated and placed into new separate centrifuge tubes. Hydrophilic layers were dried by lyophilization and lipophilic layers were placed into a ventilated cold room until dry. To prepare for NMR analysis, 450 μL of deionized water and 50 μL PBS was added to the dry hydrophilic sample and placed into a 5 mm NMR tube containing 0.58 mM TSP. Lipophilic samples were stored at -80°C for later use.

### NMR data collection

NMR data for all samples were collected on a Bruker AVANCE at 600 MHz using Topspin 3.2. (Bruker Biospin, Billerica, MA USA). Standard 1D ^1^H presaturation (ZGPR), a 1D ^1^H NOESY, and a 1D ^1^H CPMG NMR experiments all collected at 298 K using a spectral width of 20.0 ppm. The ZGPR experiment was collected for every sample to ensure that the presaturation and shimming were sufficient. To ensure acceptable shimming for each sample, the linewidth of the internal TSP standard added to every sample was monitored. Shimming was considered acceptable when the TSP peak’s full width at half height was below 0.9 Hz. The ZGPR was collected using 8 transients with 2 dummy scans at 65 K points per spectrum for 2.73 seconds with 0.30 Hz line broadening. Once the shimming was judged to be acceptable, the other two experiments were collected. The 1D 1^st^ increment of the ^1^H NOESY was collected using 64 transients with 4 dummy scans at 65 K points per spectrum using 2.73 seconds of acquisition time and apodized with 0.30 Hz line broadening. The CPMG experiment was collected to obtain high quality NMR spectra of metabolites free from the interference of broad peaks of any high molecular weight molecules. CPMG spectra were collected using 64 transients with 4 dummy scans at 65 K points per spectrum for 2.66 seconds acquisition time with 0.30 Hz line broadening.

### Statistical significance analysis

NMR spectra were processed, phase adjusted, baseline corrected, and the internal TSP reference peak set to 0.0 ppm using Topspin 3.2. (Bruker Biospin, Billerica, MA USA). Spectra were manually bucketed using the AMIX software package (Bruker Biospin, Billerica, MA USA). All discernable peaks were individually bucketed to generate bucket tables containing peak areas. The bucket tables were exported to excel for further statistical analysis. A Welch’s t-test was used to determine statistically significant differences between buckets of control and study groups. A critical alpha value of 0.5 was used as the most generous level of significance testing. In addition, a Bonferroni correction to the critical alpha value was used to define the most conservative value for significance testing [38]. The Bonferroni correction was applied by dividing the uncorrected critical alpha value by the number of NMR peaks that were included in the bucket table, used for both statistical significance testing and the subsequent PCA and PLS-DA described below. The proper correction to the critical alpha value for multiple testing is complicated in the case of NMR metabolic profiling analysis since the Bonferroni correction assumes that all of the significance tests are independent, whereas the intensities of multiple peaks that belong to the same metabolite will behave in a dependent manner, thus decreasing the Bonferroni correction factor below the number of NMR peaks being considered from the same dataset. Therefore, the results are discussed both in terms of their significance relevant to the uncorrected critical alpha value and the Bonferroni corrective alpha value, that will be too conservative since many of the metabolites have multiple resonances that will behave in a dependent manner, thus violating the underlying assumption of the Bonferroni correction. Principal component analysis (PCA) was used to visualize global differences between control and study groups through analysis of the scores and loading plots. An F-test was used to determine if cluster separations in PCA scores plots between control vs. study populations were significantly different [39]. PCA loadings plots combined with statistical analysis were used to quantify the statistical significance of differences in metabolic profiles [38]. Partial least squares—discriminant analysis (PLS-DA) was conducted using the SIMCA-P software package (Umetrics, Sartorious Stedim, Sweden). R^2^ values, measures of quality of the model fit, and Q^2^ values, measure of predictive power the data, were reported for each analysis [40].

### Volcano plot analyses

The volcano plots were made using R Project for Statistical Computing (R). The calibrate software package within R allowed for labeling of values within the plot. Volcano plots allow for the easy identity of changes within datasets by plotting significance vs. fold change. In these plots, the unknown peaks that were significant by either p-value or VIP number were included. Values with both a significant p-value and fold change of over 1 were labeled and colored differently to from other values. Values with a significant p-value were colored differently from other values, but not labeled. Volcano plots were made for each biological sample and are shown in the results section.

### Metabolite identification

ChenomX Profiler (Edmonton, Alberta, CA) (https://www.chenomx.com/about/) was used to identify peaks with statistically significant differences between groups. Metabolite identifications were confirmed using the Biological Magnetic Resonance Data Bank (BMRB) [41] and the Human Metabolome Database (HMDB) [42–45]. If a metabolite was considered to be present in the spectra, a concentration was recorded and identity was further confirmed using 2D spectra and ranked according to an ordinal ranking system developed in our laboratory [46]. The ranking system can be used to assign confidence levels to all metabolite identifications. The rank levels range from 1 to 5, with 1 being the lowest confidence and 5 being the highest confidence in the metabolite identification. Assignment of rank levels of confidence to metabolite identifications is guided by a detailed decision tree as previously described [46].

### Pathway analysis

The MetaboAnalyst 3.0 software package [47–51] (www.metaboanalyst.ca) was used to investigate the pathways implicated by the identified metabolites. Metabolome view plots were generated to allow identification and analysis of the most significantly impacted pathways [50, 52].

### ROC analyses

The area under the curve of the receiver operating characteristic (AUROC) was conducted using the MetaboAnalyst 3.0 software suite (www.metaboanalyst.ca). All spectra were uploaded into the database and all peaks, identified and unidentified, were analyzed. The receiver operating characteristic (ROC) analysis allows for the measurement of sensitivity (the true positive rate) and specificity (the true negative rate) of the putative biomarker. In addition, the AUROC, which measures the area under the curve for a plot of the sensitivity versus the false positive rate (i.e. 1 –specificity), defines the accuracy of a putative biomarker. The AUC is a single number between 0–1, with a value of 0.5 indicating no better accuracy than a random prediction and values approaching a value of 1 indicating a near perfect biomarker with 100% sensitivity and 0% false positive rate.

### Heat map generation

Heat maps were generated using Excel. The shading intensity of the cells was based on the p-values and VIP scores for a given metabolite calculated in the following way. If a fold change was positive, then the shading was a function of -Log(p) + VIP. The maximum value of -Log(p) + VIP was set as the upper limit for the shading gradient. The minimum of the shading gradient was set as -Log(0.05) + 1.0 = 2.3. This sum was based on the critical α-value used to determine a significant p-value and the threshold of a VIP score = 1. Sums of -Log(p) + VIP < 2.3 were not shaded. If the fold change was negative, then the shading was a function of +Log(p)—VIP. The lower limit for shading for negative fold changes using this algorithm was -2.3. Red shading indicated a study/control fold change greater than 1, i.e. the metabolite concentration was higher in the study group, and these values were reported as positive fold changes. Blue shading indicated a study/control fold change less than 1, i.e. the metabolite concentration was higher in the control group, and these values were reported as negative fold changes.

## Results and discussion

### Genotyping analysis

**Fig 1:**
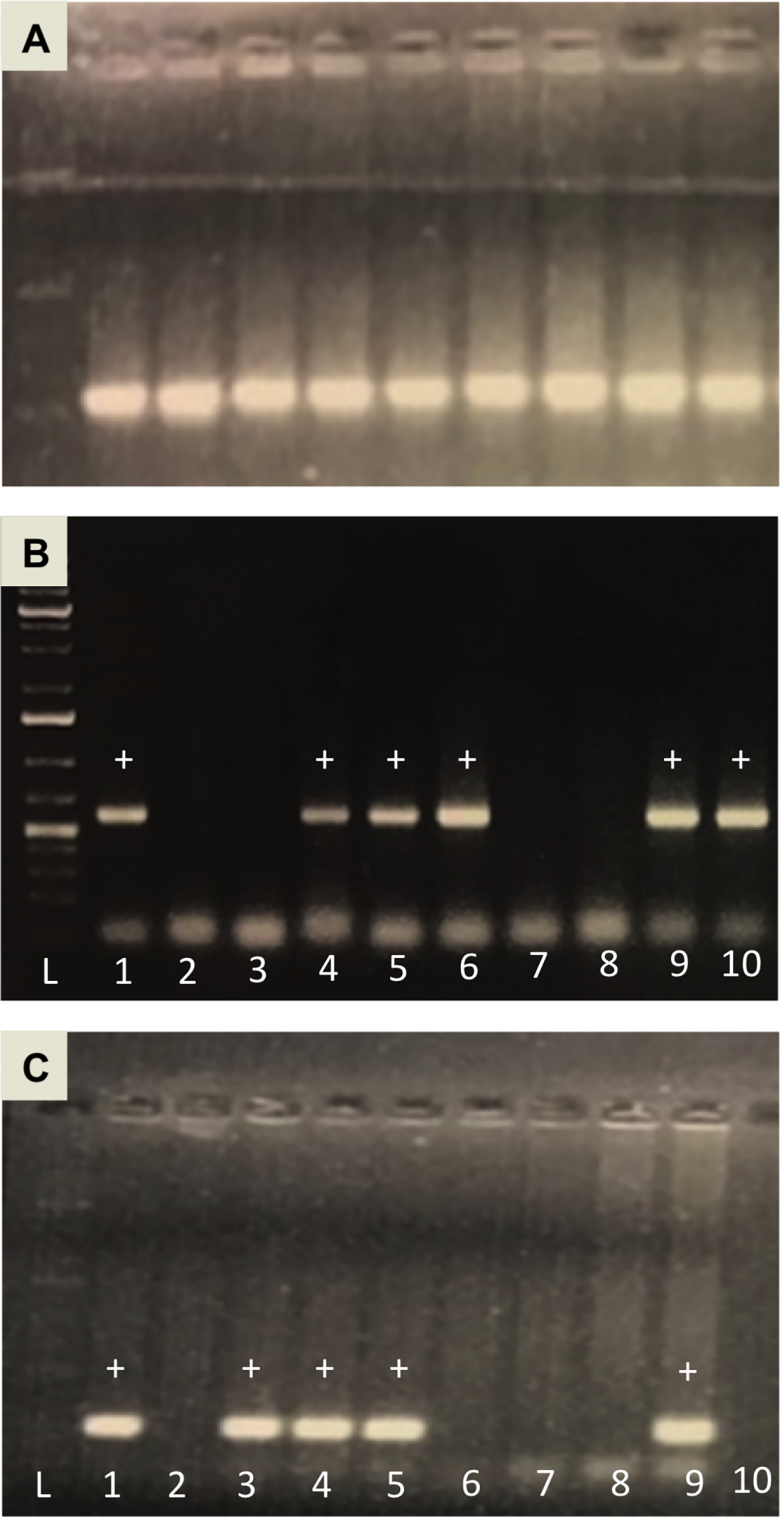
Example of ethidium bromide stained agarose gels used for genotyping. (A) Control gel to ensure DNA was present. (B) Gel screening for the LSL-KrasG12D mutation at 550 bp. (C) Gel screening for the Ptf1a-Cre knock-in at 250 bp.

Mice of the KrasG12D mouse strain, B6.129-Kras<tm4Tyj>, that contained a mutated Kras gene were bred with mice of the Cre mouse strain, B6.Ptf1a(tm1.1(cre)Cvw), that contained cre-recombinase at the Ptf1a-p48 locus. By crossbreeding these two strains of mice, three types of pups were produced, mice positive for both (Ptf1a^Cre/+^;LSL-Kras^G12D/+^), mice negative for both (Ptf1a^Cre/-^;LSL-Kras^G12D/-^), and mice positive for one or the other (Ptf1a^Cre/+^;LSL-Kras^G12D/-^ or Ptf1a^Cre/-^;LSL-Kras^G12D/+^). **Fig 1** shows an example of an ethidium bromide stained agarose gel used to determine the genotyping of offspring. A control gel was run to ensure that DNA was successfully extracted from the ear clipping (**Fig 1A**). For each lane for which the presence of genomic DNA was confirmed, it was then possible to interpret the PCR results probing for the presence of either the LSL-KrasG12D mutation (**Fig 1B**) or the Ptf1a-Cre knock-in (**Fig 1C**). For example, when a sample was positive for both KrasG12D and Ptf1a-Cre (**lane 1, Fig 1B and 1C**) the mouse was determined to be a study mouse (Ptf1a^Cre/+^;LSL-Kras^G12D/+^). When the sample was negative for both KrasG12D and Ptf1a-Cre (**lane 2, Fig 1B and 1C**) the mouse was determined to be a control mouse (Ptf1a^Cre/-^;LSL-Kras^G12D/-^). When the sample was positive for Ptf1a-Cre but negative for KrasG12D (**lane 3, Fig 1B and 1C**) the mouse was determined to be a (Ptf1a^Cre/+^;LSL-Kras^G12D/-^) breeding mouse. Finally, when the sample was positive for KrasG12D but negative for Ptf1a-Cre (**lane 3, Fig 1B and 1C**), the mouse was determined to be a (Ptf1a^Cre/-^;LSL-Kras^G12D/+^) breeding mouse. Just over 3,200 mice were genotyped to identify ~512 male and female study mice (Ptf1a^Cre/+^;LSL-Kras^G12D/+^), ~512 male and female control mice (Ptf1a^Cre/-^;LSL-Kras^G12D/-^), and ~1024 mice that carried one or the other gene modification (Ptf1a^Cre/+^;LSL-Kras^G12D/-^ or Ptf1a^Cre/-^;LSL-Kras^G12D/+^) that were used for further breeding.

### Histological analyses of pancreas tissue sections

**Fig 2:**
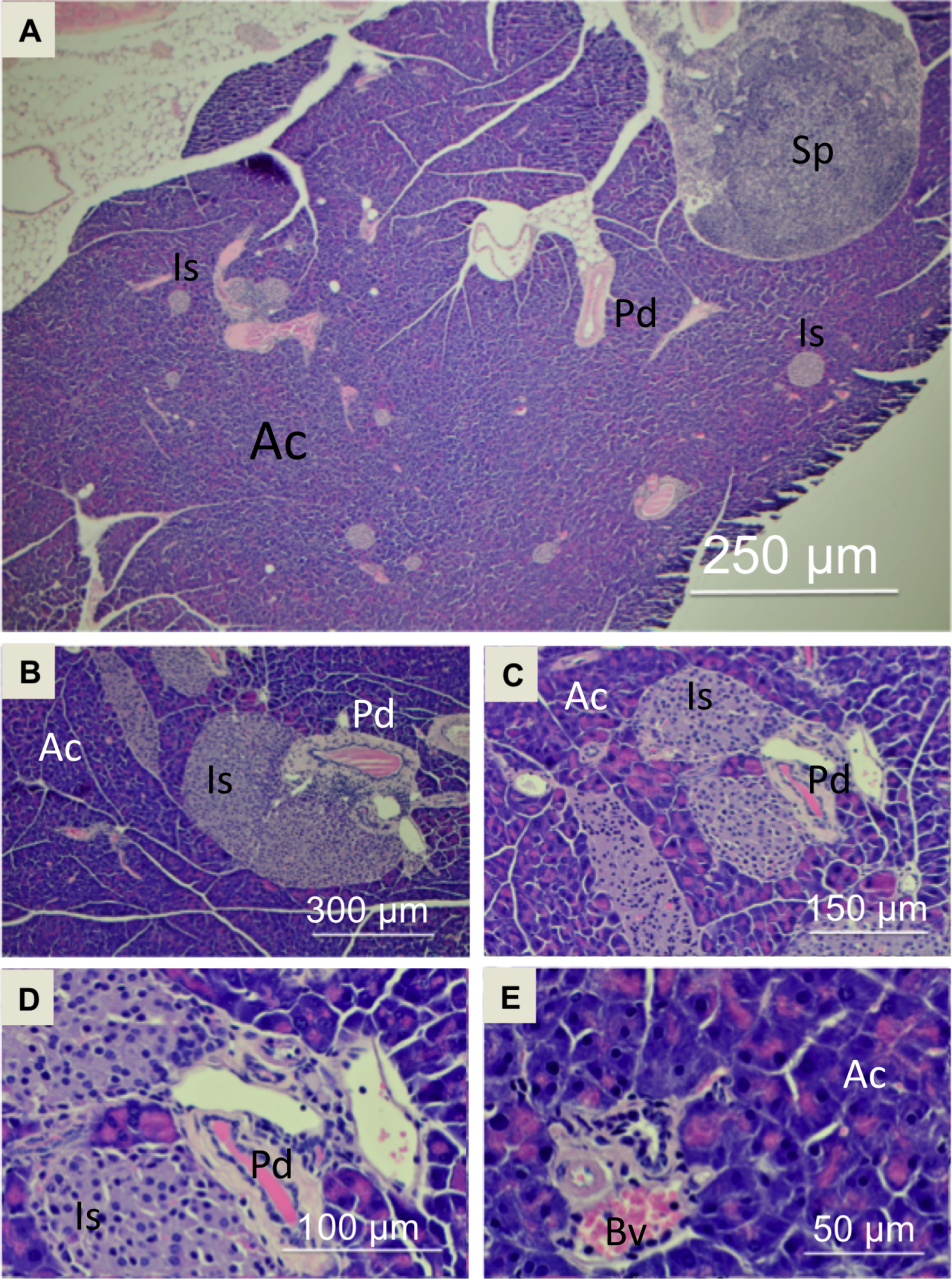
Representative hematoxylin and eosin stained images from a 15-month old female control mouse. Magnification of the images are at (A) 4X, (B) 10X, (C) 20X, (D) 40X and (E) 60X. Length bars are included in each image as a guide. Pancreas structures are labeled as follows: normal acinar tissue (Ac), islets of Langerhans (Is), pancreatic duct (Pd), blood vessel (Bv), attached spleen (Sp).

**Fig 3:**
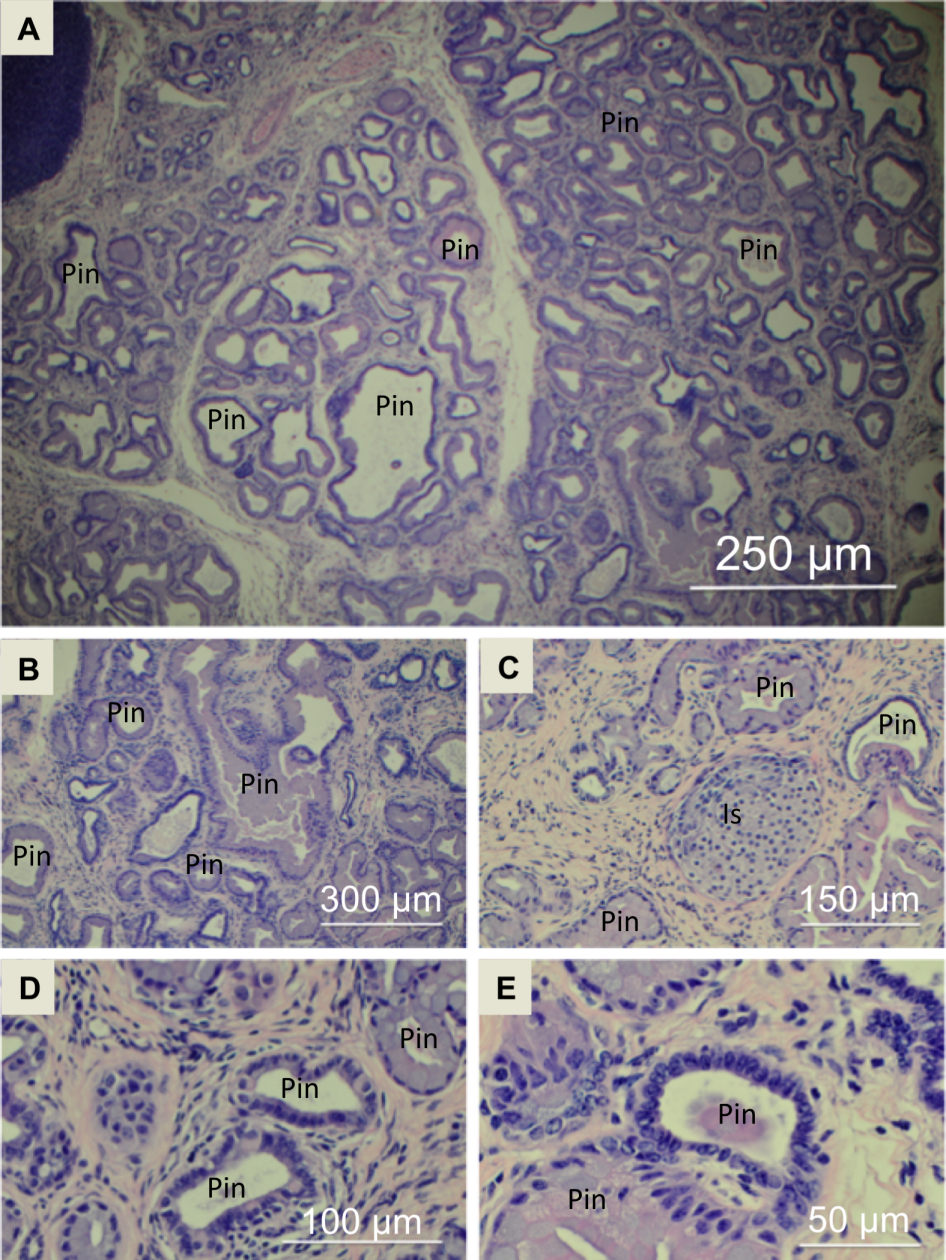
Representative hematoxylin and eosin stained images from a 15-month old female study mouse. Magnification of the images are at (A) 4X, (B) 10X, (C) 20X, (D) 40X and (E) 60X. Length bars are included in each image as a guide. The following structures are labeled in the images: PanIN (Pin) and islets (Is).

Histological analysis was used to characterize pancreas tissue in both healthy control mice and in study mice. Representative histology images of a normal healthy pancreas obtained from a female control mouse are shown in **Fig 2** illustrating normal acinar tissue (Ac), islets (Is), pancreatic ducts (Pd) and blood vessels (Bv). Representative sections are shown at low magnification (**Fig 2A**) and increasing magnification in **Fig 2B–2E**. Histology sections were analyzed to determine the extent of PanIN present in study mice. Representative histology sections from a 15-month old female study mouse are shown in **Fig 3**. At low magnification (**Fig 3A**), it can be seen that virtually the entire pancreas tissue has been transformed by a process of acinar-to-ductal metaplasia that results in effectively complete replacement of normal acinar tissue with PanIN (Pin). With increasing magnification, one can look more closely at the cellular organization of the PanIN (**Fig 3B–3E**). As expected, the PanIN burden on the pancreatic tissue increased dramatically with age, with pancreatic tissue from 5-month old study mice showed the lowest PanIN burden (typically on the order of 25% of the total tissue) and was mostly composed of normal acinar tissue, the pancreatic tissue from 11-month old mice showed increased PanIN (typically on the order of 65% of the total tissue) compared to 5-month mice with a decrease in the amount of normal acinar tissue, and the 15 month mice pancreatic tissue consistently exhibited almost completely transitioned PanIN tissue (typically near 100% of the total tissue) with very minimal amounts of normal acinar tissue, as can be seen in **Fig 3A**. Histology images for the other gender and age categories can be seen in Figures A–J in S1 File. Histological analysis was also used as an independent method to ensure that no mice were misidentified due to a genotyping error and mistakenly misclassified either into the control or study groups.

### Metabolic profiling analysis of urine samples from precancerous 15-month old male control and study mice

**Fig 4:**
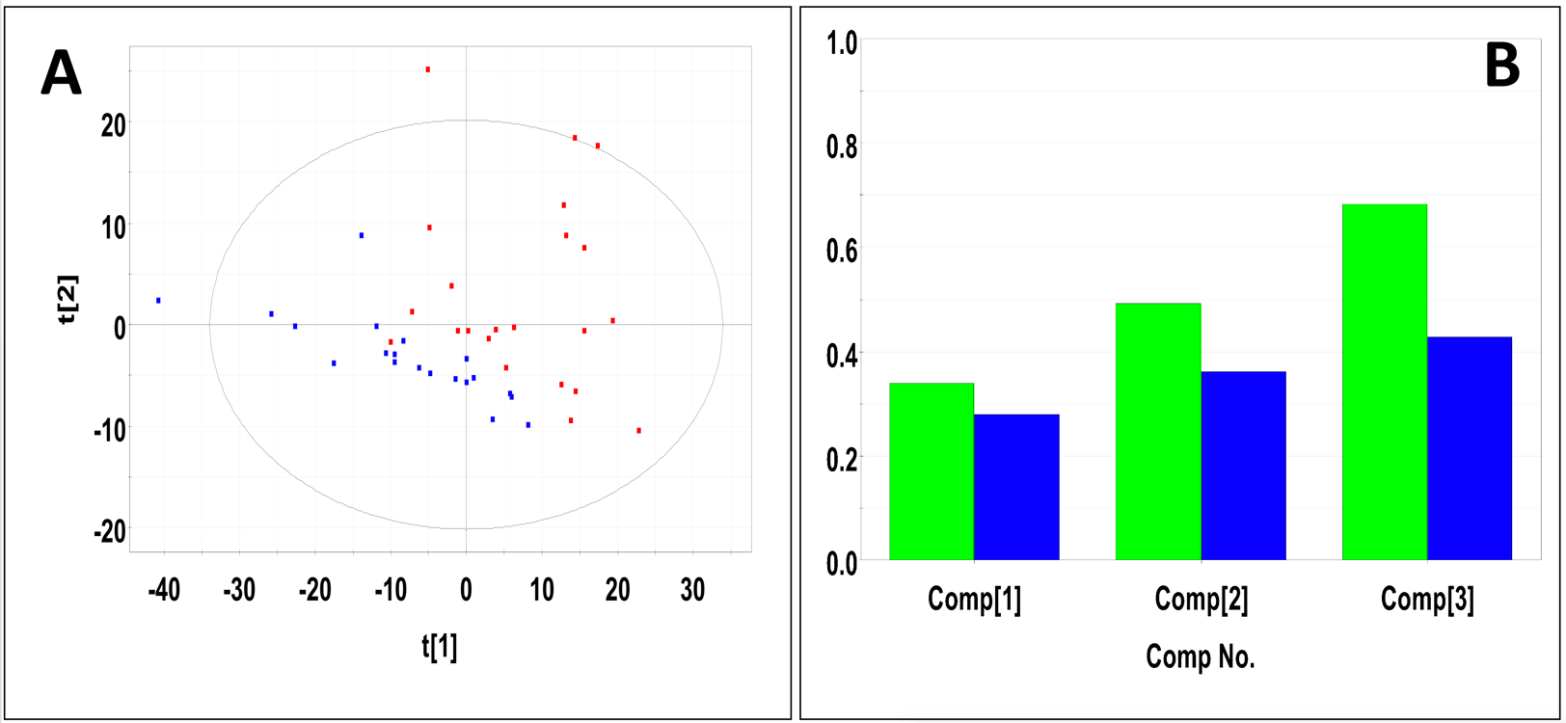
PLS-DA of urine samples obtained from 15-month old male control and study mice. (A) PLS-DA scores plot calculated using the first two principal components. The blue points indicate the control mice and the red points indicate the study mice. (B) Plot of R^2^Y and Q^2^ for the first three principal components. The green bars indicate the accumulated R^2^Y values and the blue bars indicated the accumulated Q^2^ values.

**Fig 5:**
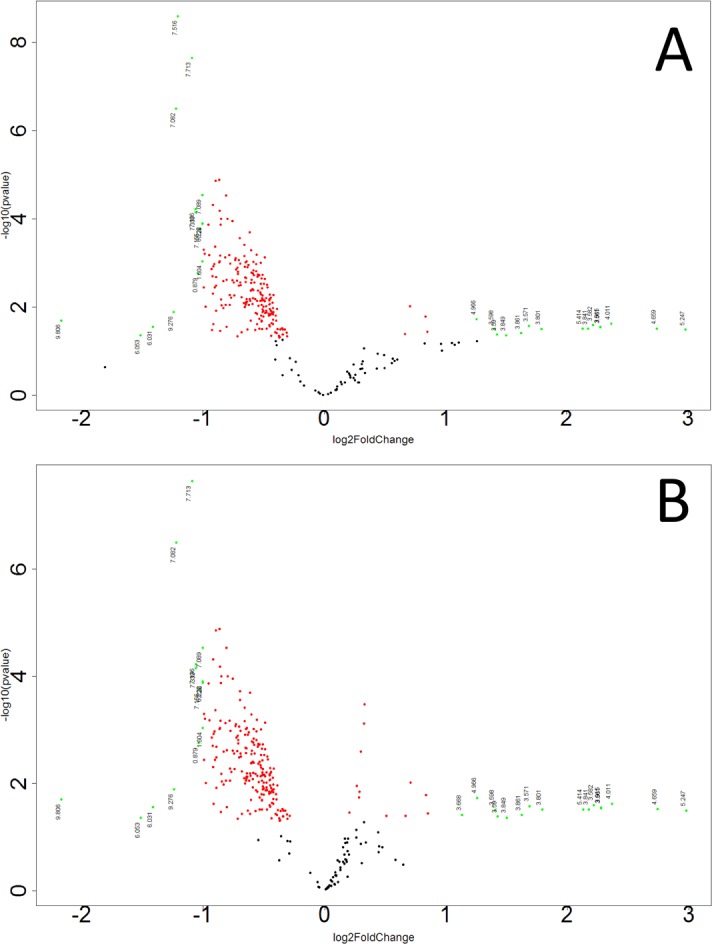
Volcano plot analysis of unidentified buckets in urine samples from 15-month male study mice. The volcano plots are presented (A) male and (B) female sample analysis. Buckets are plotted as points with the Log_2_(Fold Change) along the x-axis and -Log(p-value) along the y-axis. Buckets with |Log_2_(Fold Change)|>1 and -Log(p-value) >1.3, i.e. p <0.05, are colored green. Buckets with |Log_2_(Fold Change)|<1 and -Log(p-value) >1.3, i.e. p <0.05, are colored red.

Representative 1D 1H NMR spectra of 15-month old male control and study mice are shown in Figure K in S1 File. NMR spectra of 15-month old male control and study mice urine samples did not separate into distinct clusters in the PCA scores plot (Mahalanobis distance = 0.77, F-statistic = 3.05, F-critical = 3.24) (Figure L in S1 File). Statistical significance indicated 276 potentially significant buckets based on p-values < 0.05 43 of which could be identified and 18 buckets significant based on a Bonferroni-corrected alpha value = 1.03E-4, five of which could be identified. Potentially significant metabolites included 2-oxoglutarate, 3-indoxylsulfate, benzoate, citrate, creatinine, fructose, glucose, hippuric acid, methylamine, taurine, and trans-aconitate. PLS-DA of 15-month old male control and study mice produced a scores plot (**Fig 4A**) in which group separation was statistically significant (Mahalanobis distance = 1.93, F-statistic = 19.0, F-critical = 3.24). Cross-validation of the PLS-DA model using three principal components yielded R^2^ = 0.68 indicating good fit with the model, and Q^2^ = 0.43, indicating weak predictive capability (**Fig 4B**). PLS-DA yielded 208 significant buckets based on a VIP score > 1, 43 of which were identified. Significant metabolites based on VIP scores > 1 included 2-oxoglutarate, 3-indoxylsulfate, benzoate, creatine, creatinine, fructose, glucose, hippuric acid, methylamine, taurine, trans-aconitate, and trigonelline. Concentrations and fold changes for the metabolites are reported in **Table 1**. Potentially important buckets that could not be identified were evaluated using volcano plot analysis (**Fig 5A**). Buckets with p-values < 0.05 and fold changes greater than 2-fold, i.e. a log_2_ (fold change) > 1 are colored green in **Fig 5A** and listed in **Table 2**. Buckets with p-values < 0.05 but less than a two-fold changes were colored red in **Fig 5A** and listed in Table A in S2 File. Intensity distribution plots for the two most significant buckets are shown in Figure M in S1 File.

**Table 1:** Significant metabolites identified in urine samples from precancerous 15-month old male study mice.

ppm	VIP	p-value	Identification			Rank		AUC
		
				Control Concentration mM (StDev)	Study Concentration mM (StDev)		Fold Change (Error)	
7.699	1.68	1.50E-05	3-indoxylsulfate	4.65 (3.69)	0.99 (0.60)	2	-1.80 (0.33)	0.90
7.282	1.59	2.26E-06	3-indoxylsulfate	4.65 (3.69)	0.99 (0.60)	2	-1.93 (0.52)	0.90
7.271	1.56	1.74E-05	3-indoxylsulfate	4.65 (3.69)	0.99 (0.60)	2	-2.05 (0.29)	0.93
7.207	1.38	1.62E-05	3-indoxylsulfate	4.65 (3.69)	0.99 (0.60)	2	-1.95 (0.48)	0.89
7.197	1.01	1.28E-03	3-indoxylsulfate	4.65 (3.69)	0.99 (0.60)	2	-1.78 (0.56)	0.81
7.352	0.81	1.09E-02	3-indoxylsulfate	4.65 (3.69)	0.99 (0.60)	2	-1.54 (0.66)	0.78
3.023	1.47	6.85E-01	creatine	9.42 (7.25)	6.67 (4.43)	4	1.11 (2.16)	0.55
7.464	1.35	5.05E-05	benzoate	3.02 (4.20)	3.71 (4.74)	3	-2.38 (0.32)	0.91
7.477	1.16	2.38E-03	benzoate	3.02 (4.20)	3.71 (4.74)	3	-1.54 (0.49)	0.80
8.831	1.30	4.59E-01	trigonelline	0.96 (0.76)	0.51 (0.38)	4	-1.15 (0.75)	0.55
2.446	1.29	9.20E-01	2-oxoglutarate	19.69 (24.74)	14.06 (15.21)	4	-1.03 (1.77)	0.54
2.435	1.28	8.53E-01	2-oxoglutarate	19.69 (24.74)	14.06 (15.21)	4	-1.05 (1.70)	0.58
2.426	0.97	4.19E-03	2-oxoglutarate	19.69 (24.74)	14.06 (15.21)	4	-1.36 (0.40)	0.74
2.982	0.96	2.17E-03	2-oxoglutarate	19.69 (24.74)	14.06 (15.21)	4	-1.43 (0.41)	0.78
2.42	0.90	8.64E-03	2-oxoglutarate	19.69 (24.74)	14.06 (15.21)	4	-1.50 (0.37)	0.73
3.543	1.18	2.40E-01	fructose	60.31 (105.51)	113.76 (195.19)	2	1.48 (2.27)	0.52
4.124	1.17	2.10E-02	fructose	60.31 (105.51)	113.76 (195.19)	2	4.98 (9.63)	0.68
3.814	1.15	2.50E-02	fructose	60.31 (105.51)	113.76 (195.19)	2	4.97 (10.36)	0.65
3.554	1.15	4.65E-02	fructose	60.31 (105.51)	113.76 (195.19)	2	4.19 (9.29)	0.57
3.896	1.15	2.83E-02	fructose	60.31 (105.51)	113.76 (195.19)	2	4.99 (10.38)	0.60
3.789	1.15	3.40E-02	fructose	60.31 (105.51)	113.76 (195.19)	2	3.91 (9.02)	0.57
3.779	1.14	4.95E-02	fructose	60.31 (105.51)	113.76 (195.19)	2	2.35 (5.69)	0.54
3.549	1.12	2.95E-02	fructose	60.31 (105.51)	113.76 (195.19)	2	4.76 (28.35)	0.52
4.107	1.11	6.22E-02	fructose	60.31 (105.51)	113.76 (195.19)	2	1.62 (3.47)	0.59
3.411	1.16	2.84E-02	glucose	30.71 (102.30)	164.32 (308.72)	3	6.46 (13.32)	0.57
3.5005	1.16	3.12E-02	glucose	30.71 (102.30)	164.32 (308.72)	3	5.70 (11.58)	0.60
3.24	1.15	3.37E-02	glucose	30.71 (102.30)	164.32 (308.72)	3	5.13 (10.82)	0.55
3.731	1.15	3.14E-02	glucose	30.71 (102.30)	164.32 (308.72)	3	4.53 (9.30)	0.59
3.517	1.15	4.88E-02	glucose	30.71 (102.30)	164.32 (308.72)	3	2.77 (6.39)	0.53
3.537	1.15	3.28E-02	glucose	30.71 (102.30)	164.32 (308.72)	3	4.52 (12.40)	0.51
3.396	1.15	2.96E-02	glucose	30.71 (102.30)	164.32 (308.72)	3	5.97 (12.39)	0.58
3.703	1.15	2.84E-02	glucose	30.71 (102.30)	164.32 (308.72)	3	3.73 (8.35)	0.60
3.824	1.15	3.49E-02	glucose	30.71 (102.30)	164.32 (308.72)	3	3.45 (7.57)	0.58
3.833	1.14	3.74E-02	glucose	30.71 (102.30)	164.32 (308.72)	3	3.37 (7.93)	0.56
3.691	1.14	3.91E-02	glucose	30.71 (102.30)	164.32 (308.72)	3	2.24 (6.38)	0.54
4.632	1.13	1.83E-01	glucose	30.71 (102.30)	164.32 (308.72)	3	1.36 (2.43)	0.53
3.531	1.12	2.86E-02	glucose	30.71 (102.30)	164.32 (308.72)	3	4.33 (22.14)	0.55
5.217	1.12	4.80E-02	glucose	30.71 (102.30)	164.32 (308.72)	3	1.45 (1.54)	0.67
3.451	1.09	1.36E-01	glucose	30.71 (102.30)	164.32 (308.72)	3	1.67 (3.92)	0.51
4.041	1.15	2.60E-02	creatinine	8.19 (6.38)	7.44 (5.28)	5	4.47 (9.10)	0.61
3.474	1.15	3.26E-02	taurine	62.69 (45.49)	77.27 (90.89)	3	5.61 (11.45)	0.59
3.254	1.13	3.63E-02	taurine	62.69 (45.49)	77.27 (90.89)	3	3.52 (8.11)	0.56
6.577	1.12	2.81E-04	trans-aconitate	6.68 (7.54)	2.46 (2.61)	3	-1.96 (0.53)	0.83
1.329	1.10	1.18E-01	lactate	1.85 (1.10)	5.26 (9.21)	5	1.78 (7.84)	0.52
3.954	1.09	1.11E-01	hippuric acid	24.56 (19.22)	33.78 (11.46)	5	1.41 (2.78)	0.55
3.961	1.07	2.63E-01	hippuric acid	24.56 (19.22)	33.78 (11.46)	5	1.21 (1.72)	0.53
2.596	1.05	9.87E-04	L-methylamine	0.13 (0.11)	0.08 (0.07)	2	-1.44 (0.39)	0.78
2.563	0.93	4.91E-02	citrate	19.28 (23.22)	4.32 (5.85)	4	-1.98 (0.89)	0.77
2.535	0.93	2.40E-02	citrate	19.28 (23.22)	4.32 (5.85)	4	-1.87 (1.01)	0.77
2.684	0.91	4.86E-02	citrate	19.28 (23.22)	4.32 (5.85)	4	-1.84 (0.86)	0.75

**Table 2:** Significant unassigned buckets identified from the volcano plot analysis of urine samples from precancerous 15-month old male study mice.

ppm	VIP	p-value	Fold Change (Error)	AUC
7.516	2.44	2.59E-09	-2.31 (0.42)	0.95
7.713	2.35	2.29E-08	-2.13 (0.30)	0.95
7.082	1.68	3.21E-07	-2.33 (0.39)	0.95
7.089	1.48	2.93E-05	-2.01 (0.32)	0.91
7.136	1.28	5.95E-05	-2.09 (0.35)	0.89
7.339	1.39	7.03E-05	-2.09 (0.27)	0.91
6.222	1.16	1.26E-04	-2.01 (0.58)	0.84
7.260	1.38	1.31E-04	-2.01 (0.23)	0.91
7.155	1.18	1.73E-04	-2.04 (0.39)	0.88
1.604	1.14	9.21E-04	-2.00 (0.25)	0.86
0.879	1.20	1.76E-03	-2.05 (0.25)	0.85
9.276	0.83	1.30E-02	-2.36 (0.54)	0.75
4.966	1.15	1.87E-02	2.39 (2.25)	0.51
9.806	0.87	2.00E-02	-4.49 (7.68)	0.69
4.011	1.15	2.39E-02	5.15 (10.23)	0.66
3.582	1.15	2.56E-02	4.65 (9.03)	0.66
3.571	1.15	2.67E-02	3.22 (7.43)	0.61
6.031	0.75	2.79E-02	-2.66 (0.26)	0.83
3.915	1.15	2.83E-02	4.84 (9.82)	0.63
3.561	1.16	2.88E-02	4.85 (9.20)	0.66
4.659	1.16	3.05E-02	6.69 (13.48)	0.58
5.414	1.14	3.07E-02	4.37 (8.92)	0.62
3.841	1.15	3.09E-02	4.52 (9.50)	0.60
3.801	1.15	3.12E-02	3.46 (7.33)	0.59
3.598	1.09	3.13E-02	2.65 (5.08)	0.64
5.247	1.15	3.24E-02	7.89 (17.34)	0.62
3.861	1.14	3.92E-02	3.08 (6.84)	0.56
3.590	1.13	4.15E-02	2.68 (6.08)	0.57
3.849	1.15	4.37E-02	2.83 (5.88)	0.55
6.053	0.68	4.41E-02	-2.86 (0.25)	0.81

### Metabolic profiling analysis of urine samples from precancerous 15-month old female control and study mice

Representative ^1^H NMR CPMG spectra of urine from 15-month control and study female mice are shown in Figure N in S1 File. Female control and study mice did not separate into distinct clusters in the PCA scores plot (Mahalanobis distance = 0.081, F-Statistic = 0.034, F-Critical = 3.24) (Figure O in S1 File). Statistical significance analysis indicated 30 buckets significant based on a p-value < 0.05, 16 of which could be identified, but none were significant based on a Bonferroni-corrected alpha value = 1.03E-4. Potentially significant metabolites based on a p-value < 0.05 included 3-indoxylsulfate, benzoate, citrate, creatinine, and hippuric acid. PLS-DA produced a scores plot (Figure P in S1 File) in which group separation was statistically significant (Mahalanobis distance = 2.14, F-statistic = 23.41, F-critical of 3.24. Cross-validation of the PLS-DA model yielded R^2^ = 0.74 indicating good data agreement with the model, and Q^2^ = 0.46, indicating weak model predictive power (Figure P in S1 File). PLS-DA analysis yielded 208 buckets with VIP scores > 1 (Figure P in S1 File) from which 21 metabolites could be identified including benzoate, citrate, creatine, fructose, hippuric acid, pseudouridine, and trigonelline. Concentrations and fold changes for significant metabolites based either on p-values or VIP scores are reported in **Table 3**. Potential importance of buckets that could not be identified was evaluated using volcano plot analysis (**Fig 5B**). Buckets that had p-values < 0.05 and fold changes greater than 2 are highlighted (colored green) in **Fig 5B** and listed in **Table 4**. Buckets that had a p-value < 0.05 but less than a two-fold changes are colored red in **Fig 5B**. Unidentified buckets indicated in **Fig 5B** are listed in Table B in S2 File. Intensity distribution plots for the two most significant buckets are shown in Figure Q in S1 File.

**Table 3:** Significant metabolites identified in urine samples from precancerous 15-month old female study mice.

ppm	VIP	p-value	Identification	Control Concentration mM (StDev)	Study Concentration mM(StDev)	Rank	Fold Change (Error)	AUC
3.967	2.10	1.35E-03	hippuric acid	58.48 (85.43)	176.34 (173.51)	5	1.25 (0.36)	0.77
7.643	2.01	1.93E-03	hippuric acid	58.48 (85.43)	176.34 (173.51)	5	1.24 (0.38)	0.77
7.838	2.00	2.18E-03	hippuric acid	58.48 (85.43)	176.34 (173.51)	5	1.24 (0.38)	0.77
7.556	1.94	3.79E-03	hippuric acid	58.48 (85.43)	176.34 (173.51)	5	1.23 (0.40)	0.77
7.630	1.77	1.46E-02	hippuric acid	58.48 (85.43)	176.34 (173.51)	5	1.21 (0.51)	0.76
7.590	1.54	3.56E-02	hippuric acid	58.48 (85.43)	176.34 (173.51)	5	1.19 (0.49)	0.67
3.961	1.43	6.36E-02	hippuric acid	58.48 (85.43)	176.34 (173.51)	5	1.14 (0.24)	0.68
7.699	1.69	8.88E-03	3-indoxylsulfate	6.08 (7.38)	5.92 (9.16)	2	-1.34 (0.24)	0.75
7.282	1.64	7.23E-03	3-indoxylsulfate	6.08 (7.38)	5.92 (9.16)	2	-1.46 (0.43)	0.76
7.207	1.62	7.89E-03	3-indoxylsulfate	6.08 (7.38)	5.92 (9.16)	2	-1.49 (0.52)	0.76
7.271	1.51	1.63E-02	3-indoxylsulfate	6.08 (7.38)	5.92 (9.16)	2	-1.35 (0.52)	0.75
4.107	1.65	1.13E-01	fructose	64.41 (105.05)	153.81 (209.16)	2	1.15 (0.33)	0.70
3.543	1.19	6.90E-01	fructose	64.41 (105.05)	153.81 (209.16)	2	1.07 (0.35)	0.69
7.464	1.39	2.39E-02	benzoate	5.27 (9.53)	4.54 (4.90)	3	-1.43 (0.55)	0.74
2.684	1.19	2.95E-01	citrate	25.50 (50.32)	74.97 (212.80)	5	-1.84 (3.62)	0.59
2.535	1.19	2.85E-01	citrate	25.50 (50.32)	74.97 (212.80)	5	-1.87 (3.42)	0.59
2.563	1.18	2.89E-01	citrate	25.50 (50.32)	74.97 (212.80)	5	-1.98 (3.54)	0.59
4.150	1.08	5.24E-01	pseudouridine	3.67 (4.25)	3.03 (3.79)	1	-1.13 (0.34)	0.50
8.831	1.04	9.47E-01	trigonelline	2.08 (2.89)	4.22 (6.07)	3	-1.014 (1.14)	0.53
3.023	1.02	9.95E-01	creatine	17.08 (23.88)	16.39 (24.80)	3	-1.001 (1.24)	0.57
4.041	1.01	9.19E-01	creatinine	19.58 (22.26)	33.96 (39.53)	3	1.03 (0.73)	0.65

**Table 4:** Significant unassigned buckets identified from the volcano plot analysis of urine samples from precancerous 15-month old female study mice.

ppm	VIP	p-value	Fold Change (Error)	AUC
7.713	1.89	2.29E-08	-2.13 (0.29)	0.59
7.082	1.31	3.21E-07	-2.33 (0.51)	0.66
7.089	0.58	2.93E-05	-2.01 (1.15)	0.60
7.136	1.12	5.95E-05	-2.09 (0.52)	0.58
7.339	1.42	7.03E-05	-2.09 (0.54)	0.57
6.222	0.89	1.26E-04	-2.01 (0.87)	0.74
7.260	1.34	1.31E-04	-2.01 (0.60)	0.62
7.155	1.13	1.73E-04	-2.04 (0.49)	0.58
1.604	0.80	9.21E-04	-2.00 (0.49)	0.52
0.879	0.81	1.76E-03	-2.05 (0.47)	0.68
9.276	0.91	1.30E-02	-2.36 (1.30)	0.50
4.966	1.01	1.87E-02	2.39 (0.57)	0.62
9.806	1.03	2.00E-02	-4.49 (1.24)	0.53
4.011	0.97	2.39E-02	5.15 (0.80)	0.73
3.582	0.93	2.56E-02	4.65 (0.83)	0.53
3.571	1.002	2.67E-02	3.22 (0.58)	0.53
6.031	0.48	2.79E-02	-2.66 (0.71)	0.56
3.915	0.88	2.83E-02	4.84 (0.90)	0.62
3.561	0.93	2.88E-02	4.85 (0.82)	0.54
4.659	0.92	3.05E-02	6.69 (1.55)	0.58
5.414	0.79	3.07E-02	4.37 (0.84)	0.52
3.841	0.87	3.09E-02	4.52 (0.98)	0.54
3.801	0.95	3.12E-02	3.46 (0.63)	0.54
3.598	0.76	3.13E-02	2.65 (0.66)	0.59
5.247	0.70	3.24E-02	7.89 (1.78)	0.62
3.668	1.07	3.89E-02	2.19 (0.42)	0.51
3.861	0.93	3.92E-02	3.08 (0.66)	0.55
3.590	0.93	4.15E-02	2.68 (0.55)	0.63
3.849	0.995	4.37E-02	2.83 (0.58)	0.53
6.053	0.68	4.41E-02	-2.86 (0.70)	0.53

### Metabolic profiling analysis of urine samples collected from male and female mice with pancreatic tumors

**Fig 6:**
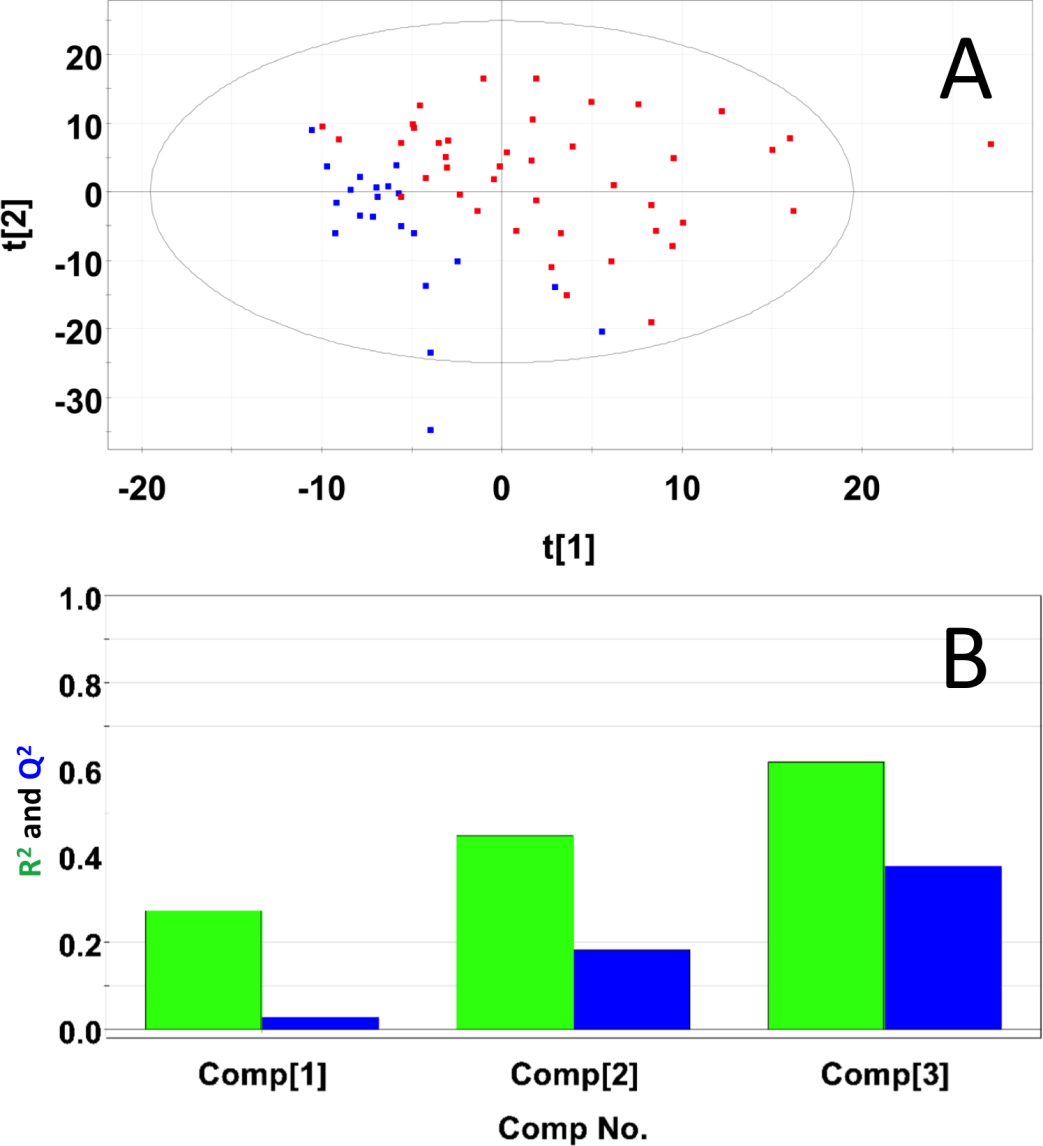
PLS-DA of urine samples from male and female mice with pancreatic tumors. **(A)** PLS-DA scores plot calculated using the first two principal components. The blue points indicate the control mice and the red points indicate the study mice. (B) Plot of R^2^Y and Q^2^ for the first three principal components. The green bars indicate the accumulated R^2^Y values and the blue bars indicated the accumulated Q^2^ values.

Urine samples were collected from 17 male and 25 female mice with pancreatic tumors. Urine samples from an equal number of gender matched male and female control mice were selected as control samples. PLS-DA produced a scores plot (**Fig 6A**) in which group separation was statistically significant (Mahalanobis distance = 1.87, F-statistic = 24.23, F-critical = 3.15). Cross-validation of the PLS-DA model using three principal components yielded R^2^ = 0.62 indicating good fit with the model, and Q^2^ = 0.37, indicating weak predictive capability (**Fig 6B**). Significant metabolites based on VIP scores > 1 included 3-indoxylsulfate, benzoate, fructose, glucose, creatinine, taurine, citrate, trigonelline, pseudouridine, 2-oxoglutarate, and creatine. Concentrations and fold changes for the metabolites are reported in **Table 5**. Potentially important buckets that could not be identified were evaluated using volcano plot analysis. Buckets with p-values < 0.05 and fold changes greater than 2-fold are listed in **Table 6**.

**Table 5:** Significant metabolites identified in urine samples from male and female mice containing pancreatic tumors.

ppm	VIP	p-value	Identification	Control Concentration mM (StDev)	Study Concentration mM (StDev)	Rank	Fold Change(Error)	AUC
7.282	2.26	3.12E-04	3-indoxylsulfate	5.37 (5.54)	6.53 (8.34)	2	-1.69 (0.43)	0.83
7.271	2.16	3.20E-04	3-indoxylsulfate	5.37 (5.54)	6.53 (8.34)	2	-1.55 (0.47)	0.82
7.699	2.26	1.61E-03	3-indoxylsulfate	5.37 (5.54)	6.53 (8.34)	2	-1.44 (0.26)	0.81
7.207	1.92	3.65E-03	3-indoxylsulfate	5.37 (5.54)	6.53 (8.34)	2	-1.48 (0.44)	0.77
7.352	1.14	8.89E-02	3-indoxylsulfate	5.37 (5.54)	6.53 (8.34)	2	-1.22 (0.58)	0.65
7.464	1.51	2.31E-02	benzoate	4.15 (6.87)	5.14 (6.96)	3	-1.38 (0.53)	0.72
4.124	1.31	2.76E-02	fructose	62.36 (105.28)	159.9 (330.10)	2	2.52 (6.77)	0.74
3.896	1.30	3.31E-02	fructose	62.36 (105.28)	159.9 (330.10)	2	2.75 (8.17)	0.73
3.814	1.30	2.76E-02	fructose	62.36 (105.28)	159.9 (330.10)	2	2.62 (7.72)	0.73
3.779	1.30	4.27E-02	fructose	62.36 (105.28)	159.9 (330.10)	2	1.75 (4.21)	0.68
4.107	1.25	1.70E-02	fructose	62.36 (105.28)	159.9 (330.10)	2	1.39 (2.19)	0.68
3.789	1.29	3.90E-02	fructose	62.36 (105.28)	159.9 (330.10)	2	2.45 (8.20)	0.66
3.554	1.28	6.56E-02	fructose	62.36 (105.28)	159.9 (330.10)	2	2.50 (7.91)	0.65
3.543	1.20	2.72E-01	fructose	62.36 (105.28)	159.9 (330.10)	2	1.24 (1.45)	0.59
3.549	1.24	2.67E-02	fructose	62.36 (105.28)	159.9 (330.10)	2	2.63 (17.95)	0.58
3.703	1.31	3.19E-02	glucose	30.71 (102.30)	145.58 (398.73)	3	2.16 (5.99)	0.71
3.731	1.30	3.56E-02	glucose	30.71 (102.30)	145.58 (398.73)	3	2.67 (7.75)	0.73
3.824	1.30	3.13E-02	glucose	30.71 (102.30)	145.58 (398.73)	3	2.28 (6.40)	0.71
3.240	1.29	4.48E-02	glucose	30.71 (102.30)	145.58 (398.73)	3	2.95 (9.04)	0.71
3.833	1.30	3.45E-02	glucose	30.71 (102.30)	145.58 (398.73)	3	2.21 (6.85)	0.70
3.5005	1.29	4.20E-02	glucose	30.71 (102.30)	145.58 (398.73)	3	3.15 (9.75)	0.68
3.517	1.29	4.12E-02	glucose	30.71 (102.30)	145.58 (398.73)	3	2.00 (5.46)	0.68
3.531	1.26	1.93E-02	glucose	30.71 (102.30)	145.58 (398.73)	3	2.79 (16.11)	0.68
3.691	1.26	2.12E-02	glucose	30.71 (102.30)	145.58 (398.73)	3	1.58 (3.96)	0.67
5.217	1.22	4.82E-02	glucose	30.71 (102.30)	145.58 (398.73)	3	1.31 (1.10)	0.67
3.451	1.14	4.47E-02	glucose	30.71 (102.30)	145.58 (398.73)	3	1.64 (3.03)	0.63
3.537	1.26	3.59E-02	glucose	30.71 (102.30)	145.58 (398.73)	3	2.40 (10.95)	0.60
4.041	1.31	3.46E-02	creatinine	13.89 (14.32)	17.33 (19.31)	3	2.38 (6.47)	0.71
3.474	1.29	4.08E-02	taurine	62.69 (45.49)	118.03 (183.04)	3	3.30 (10.24)	0.67
3.254	1.25	2.25E-02	taurine	62.69 (45.49)	118.03 (183.04)	3	2.53 (6.72)	0.67
2.684	1.21	7.84E-02	citrate	22.39 (-13.55)	63.07 (148.68)	4	1.66 (2.69)	0.63
2.563	1.21	8.82E-02	citrate	22.39 (-13.55)	63.07 (148.68)	4	1.67 (2.73)	0.62
2.535	1.20	9.24E-02	citrate	22.39 (-13.55)	63.07 (148.68)	4	1.60 (2.57)	0.61
8.831	1.15	2.38E-02	trigonelline	1.52 (1.83)	3.40 (5.20)	3	1.45 (1.20)	0.66
4.150	1.08	6.15E-01	pseudouridine	3.67 (4.25)	2.76 (3.18)	1	1.09 (0.55)	0.63
2.435	1.02	1.87E-02	2-oxoglutarate	19.69 (24.74)	47.49 (81.31)	4	1.49 (2.12)	0.61
2.426	1.02	1.40E-01	2-oxoglutarate	19.69 (24.74)	47.49 (81.31)	4	1.15 (0.59)	0.61
2.446	1.01	1.84E-02	2-oxoglutarate	19.69 (24.74)	47.49 (81.31)	4	1.57 (2.57)	0.60
3.023	0.96	2.51E-02	creatine	13.25 (15.57)	12.18 (16.58)	3	1.51 (2.36)	0.60

**Table 6:** Significant unassigned buckets identified from the volcano plot analysis of urine samples from male and female mice with pancreatic tumors.

ppm	VIP	p-value	Fold Change (Error)	AUC
2.895	1.37	1.83E-04	2.89 (5.82)	0.71
2.905	1.29	1.26E-02	2.08 (8.58)	0.69
3.395	1.29	3.50E-02	3.40 (10.69)	0.69
3.409	1.25	2.86E-02	3.42 (10.49)	0.66
3.426	1.25	1.43E-02	2.44 (5.37)	0.67
3.440	1.21	6.90E-03	2.10 (3.79)	0.68
3.561	1.31	3.65E-02	2.64 (6.94)	0.74
3.571	1.31	3.05E-02	2.10 (5.99)	0.68
3.582	1.30	2.60E-02	2.64 (7.18)	0.75
3.801	1.31	3.31E-02	2.10 (5.64)	0.70
3.841	1.30	3.70E-02	2.60 (7.96)	0.68
3.861	1.29	3.71E-02	2.05 (5.87)	0.66
3.915	1.30	3.47E-02	2.66 (7.78)	0.72
4.011	1.31	2.89E-02	2.60 (7.40)	0.73
5.247	1.27	4.58E-02	3.94 (14.52)	0.68
5.414	1.28	4.54E-02	2.60 (8.37)	0.70
9.806	1.11	6.28E-03	2.37 (4.96)	0.60

### ROC analysis of potential urinary biomarkers distinguishing control and study groups from precancerous 15-month old male and female mice and mice with pancreatic tumors

The accuracy of the potential urinary biomarkers for distinguishing between control and pre-cancerous 15-month old study mice was evaluated using area under the receiver operator characteristic (AUROC) curve analysis (**Tables 1 and 3**). Inspection of the AUCs listed in **Tables 1 and 3** indicated that some of the most significant metabolites that distinguished between control mice and 15-month old precancerous study mice based on p-value and PLS-DA VIP scores were common to both male and female mice, and that some of these metabolites had very high AUC values as well. For example, 3-indoxylsulfate was the most significant metabolite identified in the male group comparison by both p-value and VIP score (**Table 1**), and it also had 2^nd^ highest accuracy with an AUC value of 90%. 3-indoxylsulfate was also the 2^nd^ most significant metabolite identified in the female group of mice and had the highest accuracy of 75% (**Table 3**). As another example, benzoate was the highest accuracy urine biomarker in the male group with an accuracy of 91%, which corresponded to the 2^nd^ most significant metabolite by p-value (**Table 1**), and benzoate was the 3^rd^ highest accuracy biomarker in the female group at 74%. In addition to the identified metabolites, there were 11 unidentified peaks in the male group comparison that had accuracies >85% (**Table 2**), however none of the unidentified metabolites in the female groups comparison had an accuracy > 73.9% (**Table 4**).

Evaluation of the urine obtained from mice with pancreatic tumors yielded very similar results. For example, 3-indoxylsulfate was again the most significant urinary metabolite distinguishing between healthy control mice and mice with pancreatic tumors based on VIP scores, p-values and accuracy followed by benzoate (**Table 5**), which was consistent with the results from the comparisons made with precancerous 15-month old mice. It is important to note that the urine samples obtained from the mice with pancreatic tumors were analyzed completely independently from the urines of the precancerous 15-month old mice, and yet the list of significant metabolites distinguishing between the healthy controls and mice with pancreatic tumors was essentially identical.

### Metabolic profiling of serum samples from precancerous 15-month old female control and study mice

**Fig 7:**
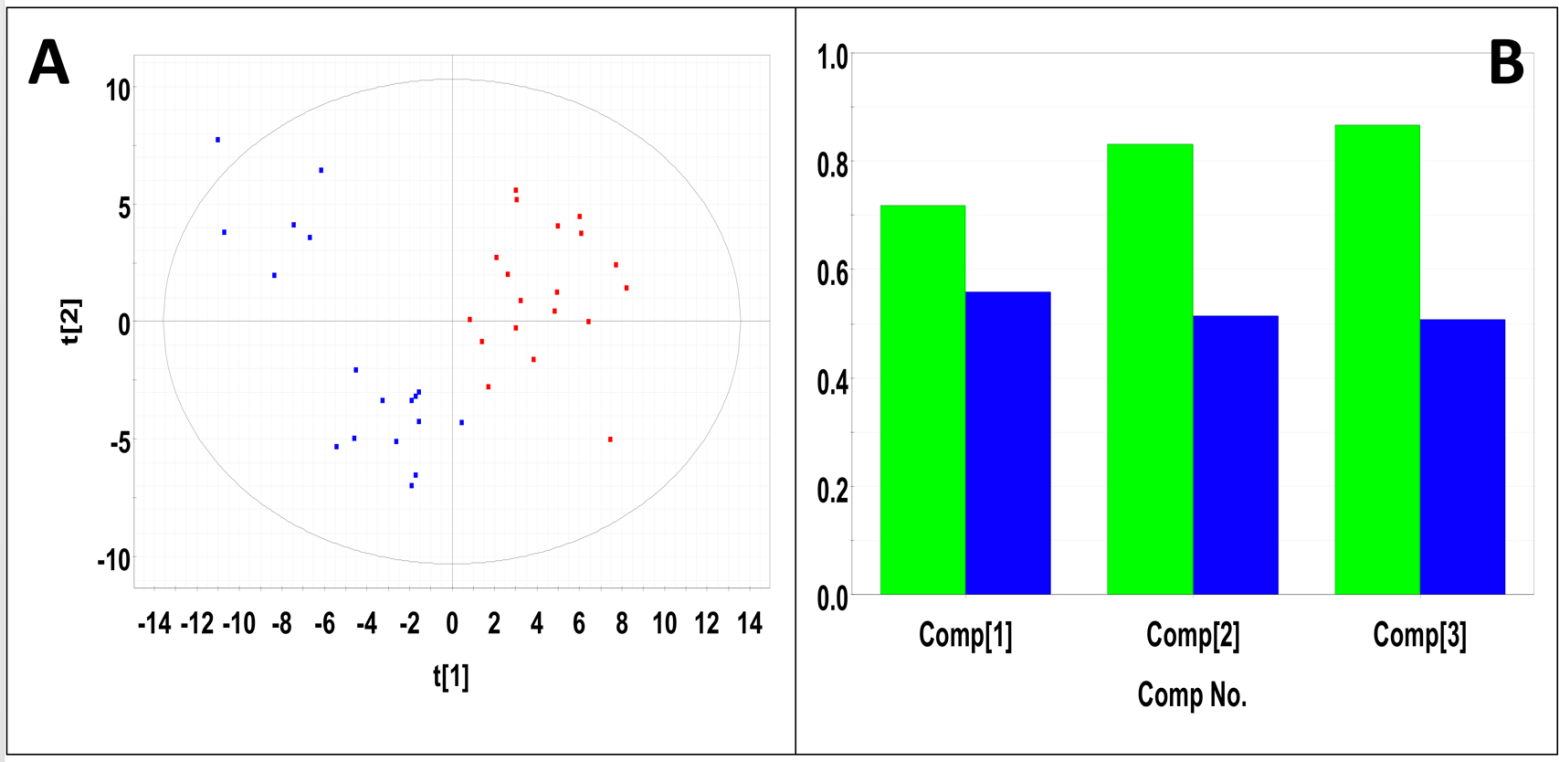
PLS-DA of serum samples from precancerous 15-month old female control and study mice. (A) PLS-DA scores plot calculated using the first two principal components. The blue points indicate the control mice and the red points indicate the study mice. (B) Plot of R^2^Y and Q^2^ for the first three principal components. The green bars indicate the accumulated R^2^Y values and the blue bars indicated the accumulated Q^2^ values.

Representative ^1^H NMR spectra of sera samples from a control mouse and a study mouse in the 15-month age group are shown in Figure R in S1 File. Analysis of the PCA scores plot (Figure S in S1 File) indicated that the NMR spectra of the control mice and the diseased mice did not separate into statistically distinct clusters (Mahalanobis distance = 0.24, F-statistic = 0.26, F-critical of 3.24). Statistical significance analysis indicated 22 buckets with p< 0.05 out of 64 binned), 11 of which were significant by the Bonferroni corrected alpha value of 2.09E-4. Significant compounds included 1,3-dihydroxyacetone, choline, citrate, glucose, glycerol, lactate, and pyruvate. PLS-DA produced a scores plot with statistically significant group separation (Mahalanobis distance = 4.31, F-statistic = 83.5, F-critical = 3.2) (**Fig 7A**). PLS-DA cross-validation (40) indicated excellent goodness of fit (R^2^ = 0.867) and moderate predictive power (Q^2^ = 0.508) (**Fig 7B**). 78 buckets had VIP scores > 1 from which 23 metabolites were identified. These compounds included 1,3-dihydroxyacetone, choline, citrate, glucose, glycerol, lactate, and pyruvate. These metabolite concentration and fold changes are reported in **Table 7**. Potential importance of buckets that could not be identified was evaluated using volcano plot analysis (Figure T in S1 File). Buckets that had p-values < 0.05 and fold changes greater than two are highlighted (colored green) in Figure T in S1 File and listed in **Table 8**. Buckets that had a p-value < 0.05 but less than a two-fold changes are colored red in Figure T in S1 File and are listed in Table C in S2 File. Bucket intensity distribution plots for the two most significant buckets are shown in Figure U in S1 File. The most significant bucket, which corresponded to an unidentified metabolite (Figure U in S1 File), had a p-value equal to 1.71 x10^-9^. The mean intensity of the control group occurred just outside the 95% confidence interval of the study group. The second most significant bucket (Figure U in S1 File) belonged to choline and had a p-value equal to 1.22 x10^-4^. This bucket intensity distribution of the control group again was slightly outside the 95% confidence interval of the study group.

**Table 7:** Significant metabolites identified from serum samples from precancerous 15-month old female study mice.

ppm	VIP	p-value	Identification	Control Concentration mM (StDev)	Study Concentration mM (StDev)	Rank	Fold Change (Error)	AUC
3.522	2.37	9.44E-07	glucose	88.33 (19.58)	74.79 (26.31)	4	-1.54 (0.27)	0.91
3.380	1.47	6.71E-03	glucose	88.33 (19.58)	74.79 (26.31)	4	-1.93 (0.24)	0.71
3.398	1.12	4.62E-02	glucose	88.33 (19.58)	74.79 (26.31)	4	-1.44 (0.27)	0.67
4.073	2.21	1.34E-05	choline	5.44 (2.30)	0.76 (0.30)	2	-1.52 (0.17)	0.89
4.065	2.09	4.94E-05	choline	5.44 (2.30)	0.76 (0.30)	2	-1.43 (0.17)	0.85
4.077	2.12	3.38E-05	lactate	182.09 (32.99)	169.34 (26.46)	4	-1.54 (0.16)	0.88
4.081	1.82	4.33E-04	lactate	182.09 (32.99)	169.34 (26.46)	4	-1.38 (0.18)	0.84
2.555	1.76	5.84E-04	citrate	2.12 (0.36)	2.46 (0.62)	2	1.33 (0.77)	0.85
4.426	1.25	4.18E-02	1,3-dihydroxyacetone	0.43 (0.45)	0.18 (0.12)	1	-2.18 (0.31)	0.65
2.359	1.21	4.00E-02	pyruvate	4.06 (0.99)	3.95 (1.06)	1	-1.31 (0.21)	0.67
3.572	1.13	4.02E-02	glycerol	23.01 (4.78)	5.11 (0.87)	4	-1.15 (0.17)	0.68

**Table 8:** Significant unassigned buckets identified from the volcano plot analysis of serum samples from the precancerous 15-month old female study mice.

ppm	VIP	p-value	Fold Change (Error)	AUC
1.173	2.77	1.71E-09	3.54 (1.36)	1.00
1.183	2.35	1.34E-06	2.05 (0.67)	0.92
3.205	2.33	5.16E-06	-2.38 (0.19)	0.94
7.846	1.45	4.51E-03	2.16 (1.57)	0.78
7.570	1.43	7.71E-03	2.06 (1.54)	0.74
7.643	1.25	1.57E-02	2.25 (1.95)	0.73
4.240	1.30	3.54E-02	-2.13 (1.31)	0.67

### Metabolic profiling of serum samples from precancerous 15-month old male control and study mice

The serum samples of the 15-month old study mice were reserved for proteomics analysis and were not subjected to metabolic profiling analysis.

### Metabolic profiling of serum samples collected from mice with pancreatic tumors

**Fig 8:**
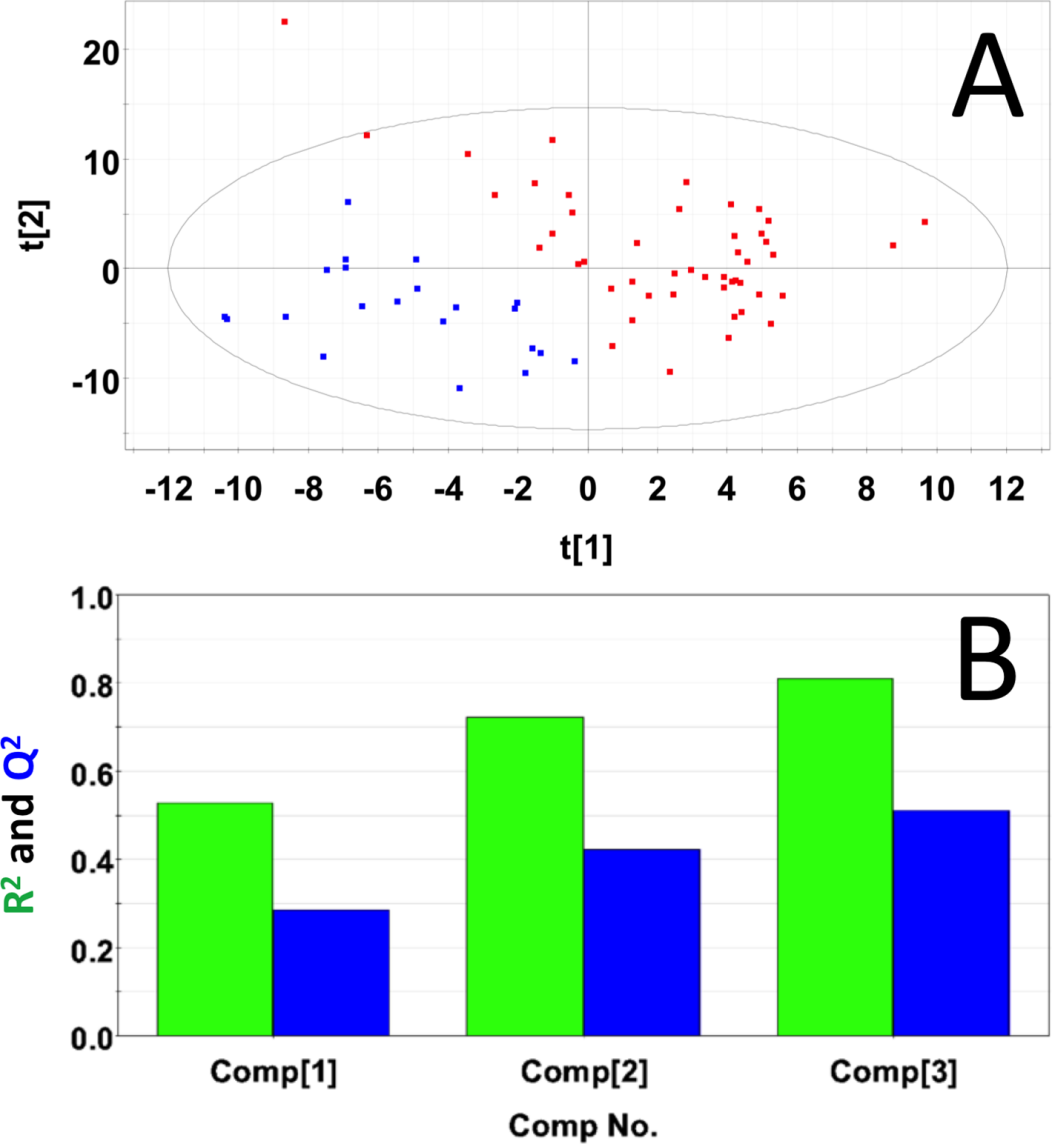
PLS-DA of sera samples from male and female mice with pancreatic tumors. **(A)** PLS-DA scores plot calculated using the first two principal components. The blue points indicate the control mice and the red points indicate the study mice. (B) Plot of R^2^Y and Q^2^ for the first three principal components. The green bars indicate the accumulated R^2^Y values and the blue bars indicated the accumulated Q^2^ values.

Sera samples were collected from 20 male and 27 female mice with pancreatic tumors. Sera samples from an equal number of gender matched male and female control mice were selected as control samples. PLS-DA produced a scores plot (**Fig 8A**) in which group separation was statistically significant (Mahalanobis distance = 3.45, F-statistic = 85.06, F-critical = 3.14). Cross-validation of the PLS-DA model using three principal components yielded R^2^ = 0.81 indicating excellent fit with the model, and Q^2^ = 0.51, indicating moderate predictive capability (**Fig 8B**). Significant metabolites based on VIP scores > 1 included glucose, glycerol, lactate, choline and citrate. Concentrations and fold changes for the metabolites are reported in **Table 9**. Potentially important buckets that could not be identified were evaluated using volcano plot analysis. Buckets with p-values < 0.05 and fold changes greater than 2-fold, are listed in **Table 10**.

**Table 9:** Significant metabolites identified from urine samples from male and female mice containing pancreatic tumors.

ppm	VIP	p-value	Identification	Control Concentration mM (StDev)	Study Concentration mM(StDev)	Rank	Fold Change(Error)	AUC
3.522	2.64	7.09E-09	glucose	88.33 (19.58)	64.40 (28.15)	4	-1.59 (0.33)	0.91
3.380	1.24	3.37E-02	glucose	88.33 (19.58)	64.40 (28.15)	4	-1.56 (0.43)	0.60
3.572	1.89	4.83E-04	glycerol	23.01 (4.78)	2.88 (1.29)	4	-1.29 (0.18)	0.80
4.077	1.82	1.12E-05	lactate	182.09 (32.99)	105.34 (29.86)	4	-1.64 (0.82)	0.93
4.081	1.45	2.20E-04	lactate	182.09 (32.99)	105.34 (29.86)	4	-1.53 (1.11)	0.91
4.073	1.75	2.17E-05	choline	5.44 (2.30)	1.59 (0.87)	2	-1.56 (0.84)	0.93
4.065	1.31	1.91E-03	choline	5.44 (2.30)	1.59 (0.87)	2	-1.33 (0.82)	0.84
2.555	1.09	2.20E-02	citrate	2.12 (0.36)	1.97 (0.72)	2	1.13 (0.85)	0.64

**Table 10:** Significant unassigned buckets identified from the volcano plot analysis of urine samples from male and female mice with pancreatic tumors.

ppm	VIP	p-value	Fold Change (Error)	AUC
3.205	2.90	6.47E-07	-2.66 (0.26)	0.95
7.755	1.26	2.81E-03	2.50 (2.90)	0.66

### ROC analysis of 15-month female serum samples and serum samples obtained from mice with pancreatic tumors

Four metabolites stood out as having promising accuracy for distinguishing between the control and study groups. At the top of the list was increased glucose, which had VIP scores as high as 2.37, p-values as low as 9.44E-07, and an accuracy of 91%. Next in line was choline, for which all its peaks had VIP scores > 2, p-values on the order of 10^−5^, and accuracies between 85 and 90%. Lactate was also promising with VIP scores ranging from 1.8 to 2.1, p-values ranging from 10^−4^ to 10^−5^, and accuracies ranging from 84 to 88%. Finally, citrate was also promising with VIP = 1.75, a p-value of 5.8 x10^-4^, and an accuracy of 85%. Among the unidentified buckets, three displayed exceptional scores (**Table 8**). For example, one peak at 1.173 ppm had a VIP = 2.77, p-value = 1.71x10^-9^ and an accuracy of 100%. Two other peaks (1.183 ppm and 3.205 ppm) had fold changes > 2.3, p-values on the order of 10^−6^, and accuracies ranging from 92–94%.

Evaluation of the serum obtained from mice with pancreatic tumors again yielded very similar results to blood obtained from precancerous mice at age 15 months old. Glucose was the most significant serum metabolite distinguishing between healthy control mice and mice with pancreatic tumors based on VIP scores as high as 2.64, p-values as low as 10^−9^ and accuracy > 90% (**Table 9**), consistent with the results from the comparisons made with sera of precancerous 15-month old mice. Lactate and choline were also among the five significant metabolites, both of which had accuracies > 90% (**Table 9**), and both which were among the top four candidate biomarkers identified from the sera of precancerous mice at age 15 months. In addition to the metabolites just discussed, there was one unassigned NMR resonance peak that had an accuracy of 95% and a p-value on the order of 10^−7^ (**Table 10**), indicating that it may be important to identify this peak in future studies.

### Metabolic profiling analysis of 15-month fecal extracts from female mice

**Fig 9:**
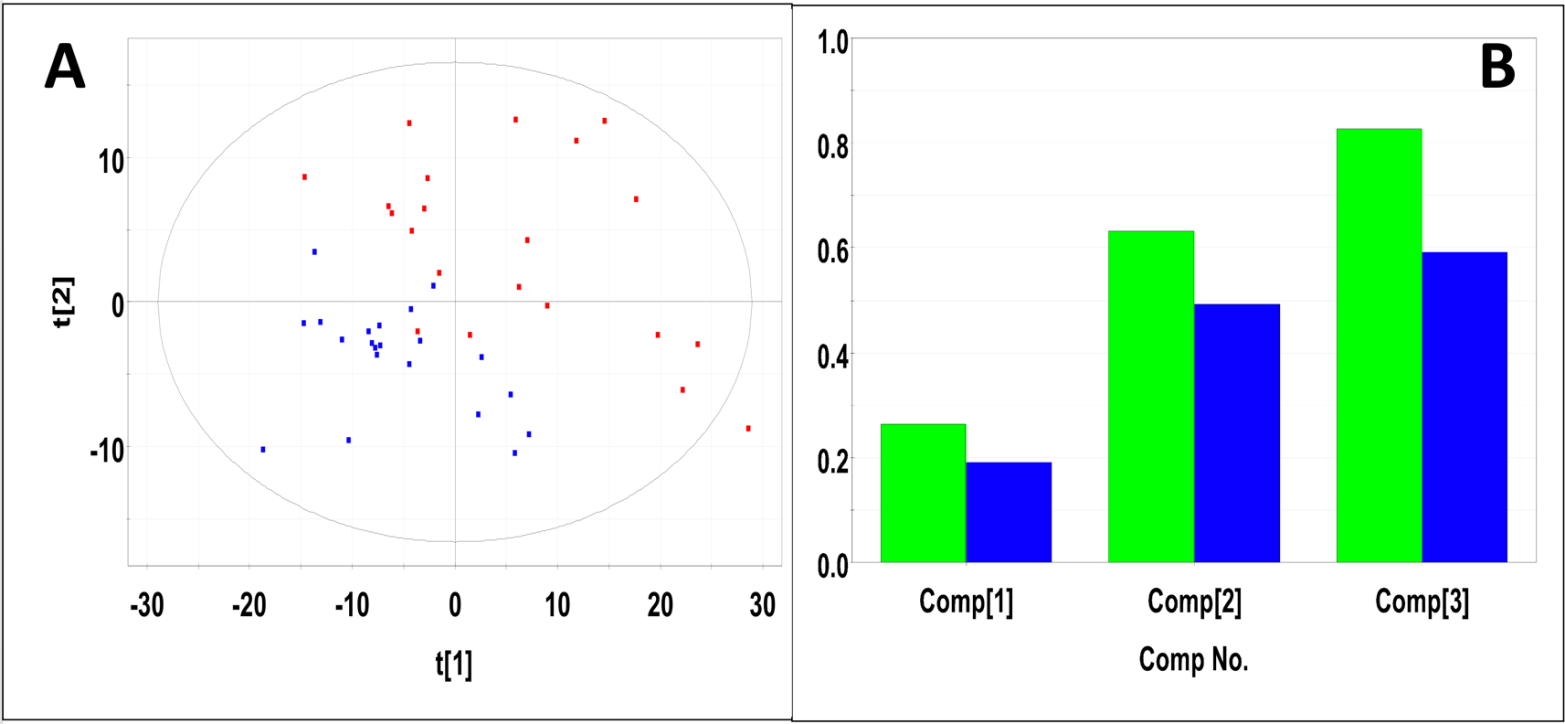
PLS-DA of fecal samples from precancerous 15-month old female control and study mice. (A) PLS-DA scores plot calculated using the first two principal components. The blue points indicate the control mice and the red points indicate the study mice. (B) Plot of R^2^Y and Q^2^ for the first three principal components. The green bars indicate the accumulated R^2^Y values and the blue bars indicated the accumulated Q^2^ values.

Representative ^1^H NMR CPMG spectra from the 15-month female control and diseased mice are shown in Figure V in S1 File. A scores plot analysis of the first two PCs from 15-month female, shown in Figure W in S1 File, indicated that the NMR spectra of the control and diseased mice did not separate into two distinct clusters in the PCA scores plot (Mahalanobis distance = 0.45, F-statistic = 1.03, F-critical = 3.24). Statistical significance analysis indicated 136 potentially significant buckets based on a p-value < 0.05, of which 29 were identified, and 7 that were significant based on a Bonferroni corrected alpha value = 1.21E-4. These metabolites included 2-oxoisocaproate, benzoate, glucose, glutamate, lactate, phenylalanine, and valine. Control and study group separation was statistically significant in the PLS-DA scores plot (Mahalanobis distance = 2.55, F-statistic = 33.37, F-critical = 3.24) (**Fig 9A**). Cross-validation of the PLS-DA (**Fig 9B**) yielded an R^2^ = 0.83 indicating excellent fit of the data to the model and a Q^2^ = 0.59 indicating moderate predictive power of the model. In the PLS-DA analysis, 157 significant buckets were identified with VIP > 1, of which 29 were identified, including acetate, acetoin, benzoate, glucose, glutamate, L-Alanine, lactate, phenylalanine, propionic acid, taurine, and valine. The concentration and fold changes for each metabolite are reported in **Table 11**. Potential importance of buckets that could not be identified was evaluated using volcano plot analysis (Figure X in S1 File). Buckets that had p-values < 0.05 and fold changes greater than two are highlighted (colored green) in Figure X in S1 File and listed in **Table 12**. Buckets that had a p-value < 0.05 but less than a two-fold changes are colored red in Figure X in S1 File and are listed in Table D in S2 File. Intensity distribution plots of the two most significant buckets are shown in Figure Y in S1 File.

**Table 11:** Significant metabolites identified from fecal samples of precancerous 15-month old female study mice.

ppm	VIP	p-value	Identification			Rank		AUC
				Control Concentration mM (StDev)	Study Concentration mM (StDev)		Fold Change (Error)	
		
4.429	1.69	5.75E-02	acetoin	2.04 (1.62)	7.53 (15.13)	5	1.30 (1.03)	0.70
3.262	1.55	1.40E-01	taurine	6.03 (7.72)	15.89 (40.76)	2	1.29 (1.07)	0.64
3.418	1.25	3.99E-01	glucose	9.51 (11.23)	40.81 (104.33)	4	1.13 (1.13)	0.57
3.249	1.25	2.59E-01	glucose	9.51 (11.23)	40.81 (104.33)	4	1.22 (1.46)	0.57
3.424	1.24	2.59E-01	glucose	9.51 (11.23)	40.81 (104.33)	4	1.24 (1.72)	0.54
3.409	1.21	3.56E-01	glucose	9.51 (11.23)	40.81 (104.33)	4	1.22 (2.06)	0.5
3.392	1.20	4.19E-01	glucose	9.51 (11.23)	40.81 (104.33)	4	1.60 (1.68)	0.51
3.48	1.20	5.40E-01	glucose	9.51 (11.23)	40.81 (104.33)	4	1.12 (1.67)	0.52
4.644	1.20	3.00E-01	glucose	9.51 (11.23)	40.81 (104.33)	4	1.26 (1.82)	0.53
3.465	1.14	7.95E-01	glucose	9.51 (11.23)	40.81 (104.33)	4	1.05 (1.44)	0.51
3.469	1.12	5.82E-01	glucose	9.51 (11.23)	40.81 (104.33)	4	1.11 (1.62)	0.52
3.438	1.11	8.16E-01	glucose	9.51 (11.23)	40.81 (104.33)	4	1.03 (0.79)	0.56
7.352	1.20	5.84E-04	phenylalanine	2.24 (1.13)	2.71 (3.51)	3	-1.80 (0.37)	0.84
7.323	1.13	1.92E-02	phenylalanine	2.24 (1.13)	2.71 (3.51)	3	-1.40 (0.85)	0.65
7.381	1.04	2.01E-02	phenylalanine	2.24 (1.13)	2.71 (3.51)	3	-1.35 (0.94)	0.67
7.31	1.00	3.18E-02	phenylalanine	2.24 (1.13)	2.71 (3.51)	3	-1.37 (0.78)	0.67
7.42	0.94	4.09E-03	phenylalanine	2.24 (1.13)	2.71 (3.51)	3	-1.45 (1.03)	0.72
7.368	0.93	1.27E-02	phenylalanine	2.24 (1.13)	2.71 (3.51)	3	-1.46 (0.53)	0.65
7.433	0.88	2.36E-02	phenylalanine	2.24 (1.13)	2.71 (3.51)	3	-1.35 (1.25)	0.65
7.476	1.19	5.06E-03	benzoate	2.63 (1.84)	2.74 (3.44)	4	-1.81 (1.06)	0.77
7.489	1.13	6.59E-03	benzoate	2.63 (1.84)	2.74 (3.44)	4	-1.87 (1.17)	0.77
7.869	1.09	7.97E-03	benzoate	2.63 (1.84)	2.74 (3.44)	4	-1.92 (1.31)	0.75
7.558	1.02	1.29E-02	benzoate	2.63 (1.84)	2.74 (3.44)	4	-1.74 (0.77)	0.72
7.545	0.84	1.32E-02	benzoate	2.63 (1.84)	2.74 (3.44)	4	-1.41 (0.70)	0.72
2.152	1.19	9.62E-01	propionic Acid	4.47 (2.79)	13.45 (21.63)	3	1.01 (0.70)	0.53
1.904	1.17	3.76E-01	acetate	74.29 (35.81)	210.81 (337.72)	3	1.11 (1.22)	0.51
0.975	1.14	5.22E-03	valine	6.41 (4.10)	10.69 (13.93)	3	-1.47 (0.63)	0.71
1.037	0.99	3.68E-03	valine	6.41 (4.10)	10.69 (13.93)	3	-1.46 (0.62)	0.75
0.987	0.97	4.73E-03	valine	6.41 (4.10)	10.69 (13.93)	3	-1.45 (0.61)	0.75
2.261	0.91	1.20E-02	valine	6.41 (4.10)	10.69 (13.93)	3	-1.33 (0.55)	0.71
2.269	0.85	2.78E-02	valine	6.41 (4.10)	10.69 (13.93)	3	-1.27 (0.57)	0.68
3.779	1.04	6.42E-01	L-alanine	9.75 (5.10)	19.44 (32.10)	4	-1.05 (0.82)	0.50
2.346	1.03	8.43E-03	glutamate	17.84 (9.94)	10.50 (4.82)	1	-1.496 (0.63)	0.71
2.118	0.99	1.71E-02	glutamate	17.84 (9.94)	10.50 (4.82)	1	-1.34 (0.69)	0.69
2.141	0.93	1.85E-02	glutamate	17.84 (9.94)	10.50 (4.82)	1	-1.39 (0.91)	0.70
2.038	0.90	1.44E-02	glutamate	17.84 (9.94)	10.50 (4.82)	1	-1.31 (0.64)	0.71
2.13	0.90	4.01E-02	glutamate	17.84 (9.94)	10.50 (4.82)	1	-1.28 (0.81)	0.66
2.33	0.88	1.33E-02	glutamate	17.84 (9.94)	10.50 (4.82)	1	-1.35 (0.73)	0.71
2.359	0.84	2.04E-02	glutamate	17.84 (9.94)	10.50 (4.82)	1	-1.37 (0.82)	0.68
2.369	0.81	2.90E-02	glutamate	17.84 (9.94)	10.50 (4.82)	1	-1.32 (0.72)	0.67
0.869	0.98	9.42E-04	butyrate	2.55 (2.03)	6.73 (9.69)	4	-1.42 (0.52)	0.77
2.622	0.98	3.13E-03	L-methionine	0.39 (0.23)	0.12 (0.29)	4	-1.44 (0.94)	0.75
2.647	0.98	3.72E-03	L-methionine	0.39 (0.23)	0.12 (0.29)	4	-1.49 (0.82)	0.76
0.932	0.93	7.65E-03	2-oxoisocaproate	0.98 (0.35)	1.14 (1.30)	4	-1.36 (0.72)	0.73
2.593	0.82	1.17E-02	2-oxoisocaproate	0.98 (0.35)	1.14 (1.30)	4	-1.339 (0.64)	0.72
2.608	0.82	2.23E-02	2-oxoisocaproate	0.98 (0.35)	1.14 (1.30)	4	-1.31 (1.11)	0.72
1.311	0.85	1.52E-02	lactate	10.10 (5.61)	52.85 (147.99)	3	-1.50 (0.27)	0.72
4.089	0.81	3.71E-02	lactate	10.10 (5.61)	52.85 (147.99)	3	-1.246 (0.67)	0.67

**Table 12:** Significant unassigned buckets identified from the volcano plot analysis of fecal samples from precancerous 15-month old female study mice.

ppm	VIP	p-value	Fold Change (Error)	AUC
1.189	2.59	3.83E-08	2.69 (0.92)	0.94
0.769	1.86	1.78E-07	2.29 (0.69)	0.91
9.487	1.38	1.97E-03	2.27 (0.79)	0.76

### Metabolic profiling analysis of 15-month fecal extracts from male mice

**Fig 10:**
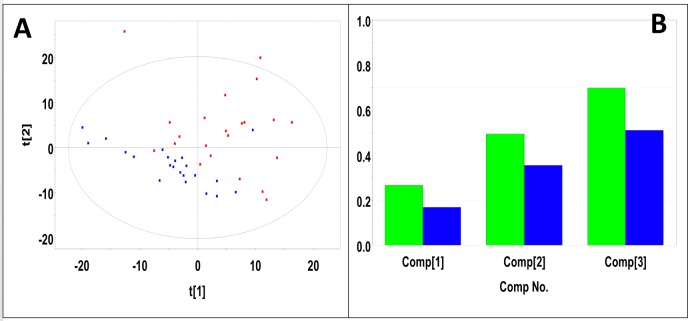
PLS-DA of fecal samples from precancerous 15-month old male control and study mice. (A) PLS-DA scores plot calculated using the first two principal components. The blue points indicate the control mice and the red points indicate the study mice. (B) Plot of R^2^Y and Q^2^ for the first three principal components. The green bars indicate the accumulated R^2^Y values and the blue bars indicated the accumulated Q^2^ values.

Representative NMR spectra of fecal extracts from 15-month old male control and study mice are shown in Figure Z in S1 File. PCA indicated that the control and study mice did not separate into distinct clusters (Mahalanobis distance = 0.47, F-statistic = 1.18, F-critical = 3.2) (Figure AA in S1 File). Statistical significance analysis indicated 89 potentially significant buckets. With p < 0.05, 24 of which were identified, and 1 significant bucket based a Bonferroni-corrected alpha value = 1.17E-4. These metabolites corresponded to 2-oxoisocaproate, acetoin, glutamate, L-alanine, L-methionine, phenylalanine, and valine. Group separation in the PLS-DA scores plot (**Fig 10A**) was statistically significant (Mahalanobis distance = 1.93, F-statistic = 20.04, F-critical = 3.22). PLS-DA cross-validation **(Fig 10B**) yielded an R^2^ = 0.70 indicating good data fit with the model and a Q^2^ of 0.51 indicating moderate predictive power. 173 buckets were considered significant based on VIP scores > 1, of which 50 were identified. The corresponding metabolites included 2-oxoisocaproate, acetoin, fructose, glucose, glutamate, glycine, L-methionine, phenylalanine, and valine. Concentration and fold changes for each known compound are reported in **Table 13**. Potential importance of buckets that could not be identified was evaluated using volcano plot analysis (Figure AB in S1 File). Buckets that had p-values < 0.05 and fold changes greater than 2 are highlighted (colored green) in Figure AB in S1 File and listed in **Table 14**. Buckets that had a p-value < 0.05 but less than a two-fold changes are colored red in Figure AB in S1 File and are listed in Table E in S2 File. Intensity distribution plots of the two most significant buckets are shown in Figure AC in S1 File.

**Table 13:** Significant metabolites identified from fecal samples from precancerous 15-month old male study mice.

ppm	VIP	p-value	Identification	Control Concentration mM	Study Concentration mM	Rank	Fold Change (Error)	AUC
				(StDev)	(StDev)			
7.352	1.65	1.38E-02	phenylalanine	1.95 (0.78)	2.03 (1.37)	3	-1.39 (0.95)	0.75
7.42	1.01	2.12E-01	phenylalanine	1.95 (0.78)	2.03 (1.37)	3	-1.21 (1.25)	0.69
3.132	1.00	7.18E-01	phenylalanine	1.95 (0.78)	2.03 (1.37)	3	-1.04 (0.67)	0.54
7.310	0.94	3.18E-02	phenylalanine	1.95 (0.78)	2.03 (1.37)	3	-1.33 (0.49)	0.69
4.429	1.47	1.93E-02	acetoin	0.88 (0.73)	4.13 (3.50)	3	1.63 (1.50)	0.67
1.375	1.1	5.61E-02	acetoin	0.88 (0.73)	4.13 (3.50)	3	1.55 (2.17)	0.60
3.553	1.32	1.18E-01	glycine	8.21 (3.68)	5.24 (2.44)	4	1.44 (1.41)	0.67
2.101	1.22	4.95E-01	glutamate	10.51 (6.06)	8.99 (6.33)	4	-1.13 (0.74)	0.56
3.737	1.09	5.21E-01	glutamate	10.51 (6.06)	8.99 (6.33)	4	1.19 (1.35)	0.52
2.328	1.07	2.08E-02	glutamate	10.51 (6.06)	8.99 (6.33)	4	-1.36 (0.52)	0.72
2.359	1.06	1.79E-02	glutamate	10.51 (6.06)	8.99 (6.33)	4	-1.52 (0.63)	0.73
2.346	1.05	1.92E-02	glutamate	10.51 (6.06)	8.99 (6.33)	4	-1.50 (0.63)	0.74
3.747	1.04	6.05E-01	glutamate	10.51 (6.06)	8.99 (6.33)	4	1.11 (1.14)	0.52
2.141	1.04	2.27E-02	glutamate	10.51 (6.06)	8.99 (6.33)	4	-1.51 (0.81)	0.74
2.369	1.04	2.30E-02	glutamate	10.51 (6.06)	8.99 (6.33)	4	-1.46 (0.61)	0.72
2.117	1.02	3.64E-02	glutamate	10.51 (6.06)	8.99 (6.33)	4	-1.39 (0.64)	0.70
2.336	0.97	4.82E-02	glutamate	10.51 (6.06)	8.99 (6.33)	4	-1.28 (0.51)	0.69
3.46	1.21	4.86E-01	glucose	11.73 (19.84)	28.07 (32.97)	4	1.32 (1.88)	0.56
3.232	1.19	3.76E-01	glucose	11.73 (19.84)	28.07 (32.97)	4	1.30 (1.64)	0.56
3.409	1.18	4.61E-01	glucose	11.73 (19.84)	28.07 (32.97)	4	1.36 (2.09)	0.54
3.393	1.18	4.54E-01	glucose	11.73 (19.84)	28.07 (32.97)	4	1.34 (1.89)	0.57
3.249	1.17	3.68E-01	glucose	11.73 (19.84)	28.07 (32.97)	4	1.39 (1.90)	0.57
3.424	1.16	4.41E-01	glucose	11.73 (19.84)	28.07 (32.97)	4	1.35 (1.93)	0.55
3.496	1.16	4.12E-01	glucose	11.73 (19.84)	28.07 (32.97)	4	1.28 (1.67)	0.55
3.469	1.15	3.88E-01	glucose	11.73 (19.84)	28.07 (32.97)	4	1.37 (1.85)	0.59
3.514	1.13	5.39E-01	glucose	11.73 (19.84)	28.07 (32.97)	4	1.14 (1.41)	0.50
3.476	1.13	3.73E-01	glucose	11.73 (19.84)	28.07 (32.97)	4	1.31 (1.50)	0.61
3.727	1.12	4.48E-01	glucose	11.73 (19.84)	28.07 (32.97)	4	1.29 (1.62)	0.55
3.719	1.12	4.48E-01	glucose	11.73 (19.84)	28.07 (32.97)	4	1.30 (1.70)	0.58
3.465	1.11	3.43E-01	glucose	11.73 (19.84)	28.07 (32.97)	4	1.40 (1.91)	0.61
4.644	1.11	5.07E-01	glucose	11.73 (19.84)	28.07 (32.97)	4	1.31 (1.94)	0.58
3.536	1.1	4.44E-01	glucose	11.73 (19.84)	28.07 (32.97)	4	1.16 (1.10)	0.58
3.418	1.08	4.22E-01	glucose	11.73 (19.84)	28.07 (32.97)	4	1.28 (1.57)	0.56
3.438	1.08	3.59E-01	glucose	11.73 (19.84)	28.07 (32.97)	4	1.22 (1.18)	0.60
3.452	1.05	2.05E-01	glucose	11.73 (19.84)	28.07 (32.97)	4	1.36 (1.68)	0.62
3.699	1.05	5.28E-01	glucose	11.73 (19.84)	28.07 (32.97)	4	1.16 (1.26)	0.50
3.712	1.03	5.73E-01	glucose	11.73 (19.84)	28.07 (32.97)	4	1.25 (1.90)	0.51
3.529	1.02	6.25E-01	glucose	11.73 (19.84)	28.07 (32.97)	4	1.08 (1.06)	0.51
3.217	1.00	5.00E-01	glucose	11.73 (19.84)	28.07 (32.97)	4	1.08 (0.97)	0.55
2.623	1.17	7.31E-03	L-methionine	0.26 (0.12)	0.23 (0.18)	3	-1.38 (0.77)	0.72
2.647	1.08	1.85E-02	L-methionine	0.26 (0.12)	0.23 (0.18)	3	-1.43 (0.66)	0.73
2.608	1.12	1.23E-02	2-oxoisocaproate	1.15 (0.43)	0.49 (0.34)	3	-1.32 (0.70)	0.69
0.932	0.97	4.28E-02	2-oxoisocaproate	1.15 (0.43)	0.49 (0.34)	3	-1.25 (0.38)	0.70
4.106	1.08	2.15E-01	fructose	7.32 (13.21)	9.54 (13.85)	4	1.19 (1.07)	0.61
2.262	1.02	3.54E-02	valine	7.61 (4.10)	6.94 (5.29)	4	-1.30 (0.48)	0.71
2.275	1.02	4.09E-02	valine	7.61 (4.10)	6.94 (5.29)	4	-1.34 (0.50)	0.69
2.281	1.02	3.21E-02	valine	7.61 (4.10)	6.94 (5.29)	4	-1.31 (0.52)	0.72
2.269	1.01	4.17E-02	valine	7.61 (4.10)	6.94 (5.29)	4	-1.29 (0.48)	0.68
1.038	1.01	2.79E-02	valine	7.61 (4.10)	6.94 (5.29)	4	-1.46 (0.66)	0.73
0.987	0.97	3.85E-02	valine	7.61 (4.10)	6.94 (5.29)	4	-1.42 (0.60)	0.72
0.975	0.96	4.20E-02	valine	7.61 (4.10)	6.94 (5.29)	4	-1.43 (0.89)	0.73
1.477	0.96	3.34E-02	L-alanine	11.53 (5.25)	10.57 (9.54)	4	-1.36 (0.39)	0.66

**Table 14:** Significant unassigned buckets identified from the volcano plot analysis of the precancerous 15-month old male fecal samples.

ppm	VIP	p-value	Fold Change (Error)	AUC
0.167	3.25	1.80E-07	-2.60 (0.16)	0.93
8.032	1.26	6.99E-03	-2.01 (1.11)	0.82
9.129	1.72	2.17E-03	-2.55 (1.77)	0.76
4.129	1.39	9.51E-03	2.58 (6.01)	0.67
1.341	1.32	1.19E-02	2.65 (6.85)	0.64
1.327	1.30	1.30E-02	2.52 (6.49)	0.64
9.487	1.54	1.61E-02	-2.13 (0.73)	0.67

### Metabolic profiling analysis of fecal extracts from male and female mice with pancreatic tumors

**Fig 11:**
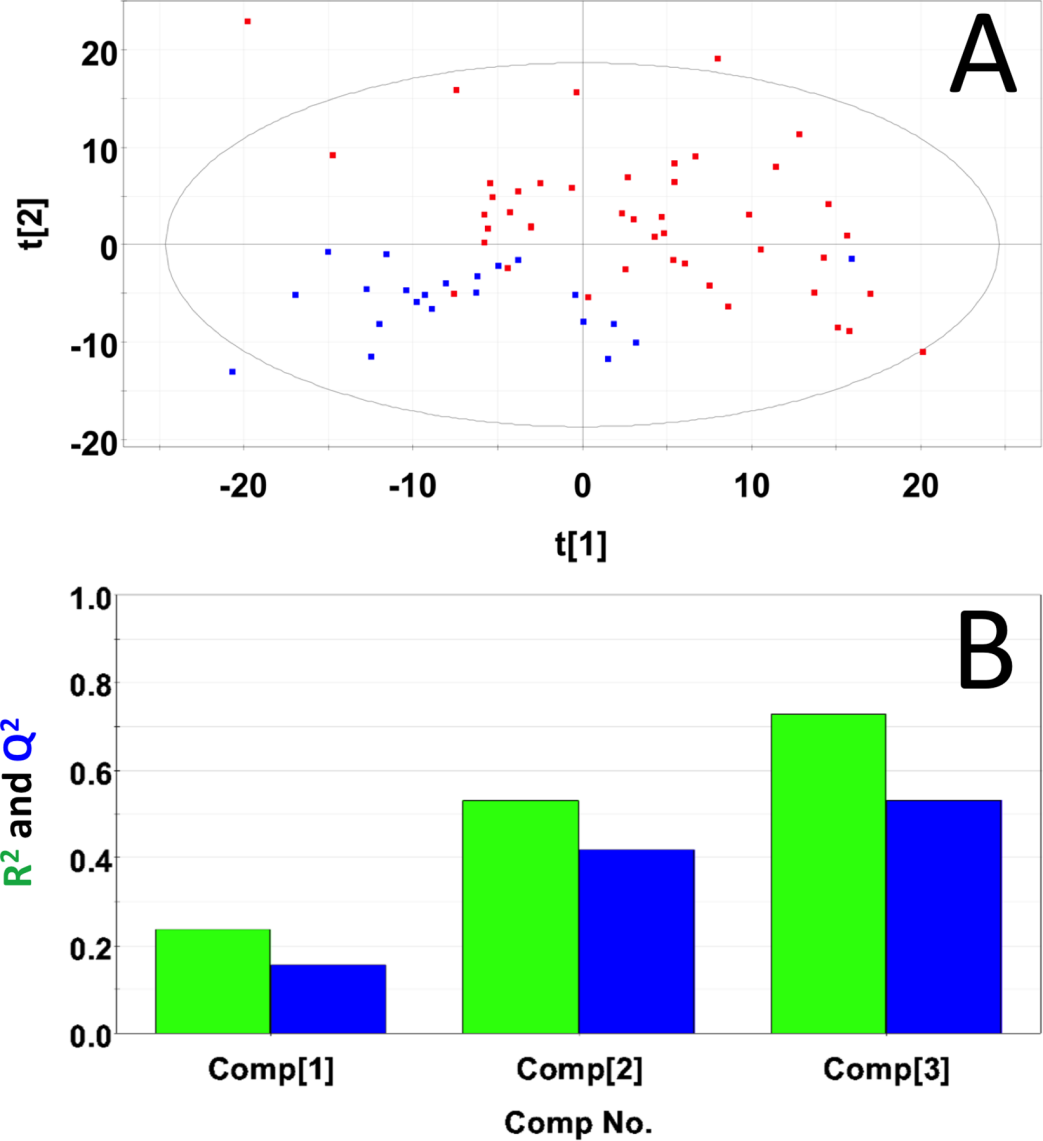
PLS-DA of fecal extracts from male and female mice with pancreatic tumors. **(A)**PLS-DA scores plot calculated using the first two principal components. The blue points indicate the control mice and the red points indicate the study mice. (B) Plot of R^2^Y and Q^2^ for the first three principal components. The green bars indicate the accumulated R^2^Y values and the blue bars indicated the accumulated Q^2^ values.

Fecal extract samples were collected from 19 male and 25 female mice with pancreatic tumors. Fecal extract samples from an equal number of gender matched male and female control mice were selected as control samples. PLS-DA of fecal extracts produced a scores plot [**Fig 11A**] in which group separation was statistically significant (Mahalanobis distance = 2.23, F-statistic = 36.16, F-critical = 3.14). Cross-validation of the PLS-DA model using three principal components yielded R^2^ = 0.73 indicating excellent fit with the model, and Q^2^ = 0.53, indicating moderate predictive capability [**Fig 11B**]. Significant metabolites based on VIP scores > 1 included glucose, acetoin, propionic acid, phenylalanine, L-methionine, glutamate, L-alanine, and taurine. Concentrations and fold changes for the metabolites are reported in **Table 15**. Potentially important buckets that could not be identified were evaluated using volcano plot analysis. Buckets with p-values < 0.05 and fold changes greater than 2-fold, are listed in **Table 16**.

**Table 15:** Significant metabolites identified in fecal extracts from male and female mice containing pancreatic tumors.

ppm	VIP	p-value	Identification	Control Concentration mM (StDev)	Study Concentration mM (StDev)	Rank	Fold Change (Error)	AUC
3.465	1.38	5.11E-01	glucose	10.62(15.54)	21.17 (61.17)	4	1.14 (1.23)	0.54
3.452	1.37	6.83E-01	glucose	10.62(15.54)	21.17 (61.17)	4	1.06 (1.06	0.51
3.480	1.36	2.67E-01	glucose	10.62(15.54)	21.17 (61.17)	4	1.22 (1.36)	0.57
3.409	1.35	2.15E-01	glucose	10.62(15.54)	21.17 (61.17)	4	1.31 (1.46)	0.58
3.392	1.34	2.65E-01	glucose	10.62(15.54)	21.17 (61.17)	4	1.25 (1.26)	0.58
3.496	1.34	3.60E-01	glucose	10.62(15.54)	21.17 (61.17)	4	1.14 (1.32)	0.54
3.424	1.33	1.24E-01	glucose	10.62(15.54)	21.17 (61.17)	4	1.35 (1.37)	0.61
4.644	1.33	1.43E-01	glucose	10.62(15.54)	21.17 (61.17)	4	1.37 (1.45)	0.59
3.232	1.33	6.20E-01	glucose	10.62(15.54)	21.17 (61.17)	4	1.07 (1.18)	0.51
3.249	1.32	1.48E-01	glucose	10.62(15.54)	21.17 (61.17)	4	1.31 (1.31)	0.61
3.469	1.31	3.44E-01	glucose	10.62(15.54)	21.17 (61.17)	4	1.18 (1.25)	0.56
3.719	1.26	3.93E-01	glucose	10.62(15.54)	21.17 (61.17)	4	1.14 (1.19)	0.54
3.418	1.24	2.77E-01	glucose	10.62(15.54)	21.17 (61.17)	4	1.18 (1.17)	0.59
3.727	1.22	4.69E-01	glucose	10.62(15.54)	21.17 (61.17)	4	1.12 (1.10)	0.54
3.438	1.20	2.53E-01	glucose	10.62(15.54)	21.17 (61.17)	4	1.12 (1.10)	0.57
3.699	1.13	9.54E-01	glucose	10.62(15.54)	21.17 (61.17)	4	1.01 (1.25)	0.56
3.514	1.12	6.65E-01	glucose	10.62(15.54)	21.17 (61.17)	4	1.05 (1.55)	0.54
3.712	1.02	8.64E-01	glucose	10.62(15.54)	21.17 (61.17)	4	1.03 (1.32)	0.58
4.429	1.35	4.53E-03	acetoin	1.46 (1.18)	5.00 (9.68)	3	1.41 (1.38)	0.67
2.152	1.26	2.97E-01	propionic acid	4.47 (2.79)	8.73 (15.34)	3	1.12 (0.86)	0.55
7.352	1.17	2.28E-03	phenylalanine	2.10 (0.96)	2.13 (1.96)	3	-1.62 (0.38)	0.78
7.323	0.995	3.02E-02	phenylalanine	2.10 (0.96)	2.13 (1.96)	3	-1.29 (0.81)	0.66
7.420	0.89	2.50E-03	phenylalanine	2.10 (0.96)	2.13 (1.96)	3	-1.39 (1.11)	0.75
7.433	0.89	3.72E-02	phenylalanine	2.10 (0.96)	2.13 (1.96)	3	-1.26 (1.42)	0.68
7.310	0.83	3.31E-02	phenylalanine	2.10 (0.96)	2.13 (1.96)	3	-1.29 (0.51)	0.62
2.623	1.17	6.57E-05	L-methionine	0.33 (0.18)	0.27 (0.33)	3	-1.51 (0.63)	0.77
2.647	1.09	1.75E-04	L-methionine	0.33 (0.18)	0.27 (0.33)	3	-1.55 (0.71)	0.78
3.737	1.15	7.42E-01	glutamate	14.80 (8.00)	31.00 (26.86)	1	1.04 (1.07)	0.50
3.747	1.12	6.59E-01	glutamate	14.80 (8.00)	31.00 (26.86)	1	-1.04 (1.14)	0.58
2.038	0.96	1.58E-02	glutamate	14.80 (8.00)	31.00 (26.86)	1	-1.26 (0.59)	0.67
2.101	0.95	3.48E-02	glutamate	14.80 (8.00)	31.00 (26.86)	1	-1.37 (0.64)	0.66
2.118	0.95	1.69E-02	glutamate	14.80 (8.00)	31.00 (26.86)	1	-1.30 (0.74)	0.70
2.346	0.93	9.69E-03	glutamate	14.80 (8.00)	31.00 (26.86)	1	-1.42 (0.67)	0.71
2.330	0.90	1.04E-02	glutamate	14.80 (8.00)	31.00 (26.86)	1	-1.31 (0.65)	0.71
2.141	0.88	1.09E-02	glutamate	14.80 (8.00)	31.00 (26.86)	1	-1.36 (1.00)	0.73
2.359	0.87	1.78E-02	glutamate	14.80 (8.00)	31.00 (26.86)	1	-1.32 (0.85)	0.69
2.369	0.87	3.15E-02	glutamate	14.80 (8.00)	31.00 (26.86)	1	-1.27 (0.79)	0.68
3.779	1.14	5.83E-01	L-alanine	10.64(5.18)	12.47 (20.15)	4	1.05 (1.29)	0.50
3.262	1.03	1.77E-01	taurine	6.03 (7.72)	3.3.8 (5.25)	2	1.26 (1.21)	0.61
1.037	0.97	1.63E-03	valine	7.01 (4.10)	7.21 (9.45)	3	-1.47 (0.77)	0.76
2.262	0.97	9.20E-03	valine	7.01 (4.10)	7.21 (9.45)	3	-1.31 (0.50)	0.71
0.987	0.96	2.11E-03	valine	7.01 (4.10)	7.21 (9.45)	3	-1.47 (0.79)	0.75
0.975	0.96	6.25E-03	valine	7.01 (4.10)	7.21 (9.45)	3	-1.43 (0.90)	0.72
2.269	0.91	1.88E-02	valine	7.01 (4.10)	7.21 (9.45)	3	-1.26 (0.55)	0.70
2.608	0.97	2.77E-03	2-oxoisocaproate	1.07 (0.39)	0.87 (0.94)	3	-1.30 (0.65)	0.71
2.593	0.93	3.69E-03	2-oxoisocaproate	1.07 (0.39)	0.87 (0.94)	3	-1.33 (0.49)	0.72
0.932	0.91	5.06E-03	2-oxoisocaproate	1.07 (0.39)	0.87 (0.94)	3	-1.31 (0.61)	0.74
7.476	0.91	1.27E-02	benzoate	2.63 (1.84)	2.13 (1.92)	4	-1.50 (0.88)	0.71
7.489	0.79	2.76E-02	benzoate	2.63 (1.84)	2.13 (1.92)	4	-1.47 (1.02)	0.68
7.545	0.70	3.78E-02	benzoate	2.63 (1.84)	2.13 (1.92)	4	-1.27 (0.67)	0.67
7.869	0.69	4.52E-02	benzoate	2.63 (1.84)	2.13 (1.92)	4	-1.44 (1.18)	0.67
1.311	0.84	4.52E-02	lactate	10.10 (5.61)	41.26 (107.71)	3	-1.47 (0.24)	0.69

**Table 16:** Significant unassigned buckets identified in fecal extracts from male and female mice containing pancreatic tumors.

ppm	VIP	p-value	Fold Change (Error)	AUC
0.673	1.42	1.07E-04	-2.41 (1.27)	0.88
0.692	1.28	2.37E-04	-2.11 (1.43)	0.87
0.708	2.00	6.68E-07	-2.20 (0.71)	0.89
0.769	2.27	6.83E-09	-2.32 (0.52)	0.93
2.409	1.71	1.58E-04	2.33 (4.22)	0.77

### ROC analysis of fecal extracts obtained pre-cancerous 15-month old mice and from mice with pancreatic tumors

The highest accuracy biomarker distinguishing control and study mice in the female group was phenylalanine (**Table 13**), which had 84% accuracy along with maximum VIP score of only 1.2 but significant p-values with a minimum value of 5.84E-04. Phenylalanine turned out to also be the top ranked biomarker distinguishing control and study mice in the male group (**Table 13**) with a top accuracy equal to 75% along with VIP scores as high as 1.65 and some peaks with p-values less than 0.05. Acetoin was also near the top for both female and male mice. Acetoin was 70% accurate in female mice with a reasonably high VIP score of 1.69 and p-value = 0.057, whereas in male mice, acetoin had an accuracy as high as 67% with a maximum VIP score of 1.47 and lowest p-value of 0.019. Other metabolites that reached at least 70% accuracy in both the male and female groups included valine, glutamate, methionine, and 2-oxoisocaproate. Other metabolites that reached 70% accuracy in the female comparison included benzoate, lactate and butyrate. Other promising biomarkers that distinguished control and study groups in the male mice included an unidentified bucket at 0.167 ppm which had an accuracy of 93% supported by a strong VIP score of 3.25 and a good p-value of 1.80E-07. Three additional buckets at 1.26 ppm, 1.39 ppm and 1.76 ppm had p-values less than .001 and accuracies ranging from 67 to 87% (**Table 13)**.

Evaluation of the fecal extracts obtained from mice with pancreatic tumors again yielded very similar results to fecal extracts obtained from precancerous male and female mice at age 15 months old. Phenylalanine had the highest accuracy for distinguishing between healthy control mice and mice with pancreatic tumors with and AUC as high as 78% (**Table 15**). Several other metabolites had AUC values between 70 and 80%, including methionine, glutamate, valine, 2-oxoisocaproate, and benzene, all of which were identified in the list of top metabolites distinguishing between fecal extracts of healthy control mice and precancerous 15-month old male and female mice (**Table 13**), and both which were among the top four candidate biomarkers identified from the sera of precancerous mice at age 15 months. In addition to the metabolites just discussed, there were five unidentified NMR resonances that had accuracies > 77%, with the two highest accuracy peaks having AUC values between 89% and 93% and these had p-values on the order of 10^−7^ and 10^−9^, respectively, **(Table 16)**, indicating that the metabolites corresponding to these unassigned peaks may be of significant interest warranting future identification.

### Metabolic profiling of pancreas tumor extracts

**Fig 12:**
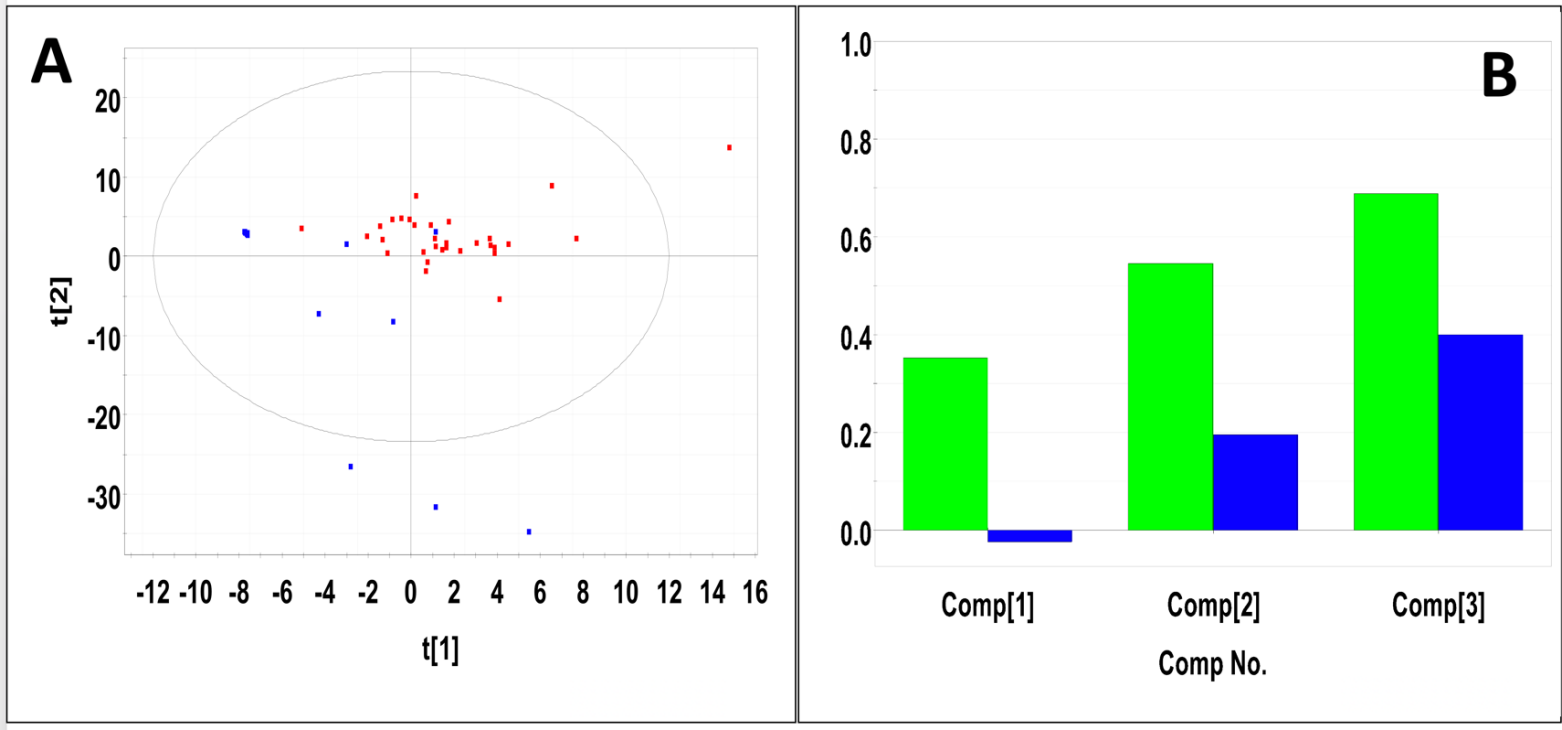
PLS-DA of healthy pancreatic tissue extracts and tumor extracts from male and female control and study mice. (A) PLS-DA scores plot calculated using the first two principal components. The blue points indicate the control mice and the red points indicate the study mice. (B) Plot of R^2^Y and Q^2^ for the first three principal components. The green bars indicate the accumulated R^2^Y values and the blue bars indicated the accumulated Q^2^ values.

Representative ^1^H NMR CPMG spectra of extracts from the pancreatic tissue of a control mouse and from the extract of a tumor from a study mouse is shown in Figure AD in S1 File. 31 tumor extracts (6 male and 25 female) and 14 control tissue samples were subjected to multivariate statistical data analysis to identify differences in metabolic profiles. Control and study mice grouped into distinct clusters in the PCA scores plot (Mahalanobis distance = 1.06, F-statistic = 5.35, F-critical = 3.25) (Figure AE in S1 File). 19 buckets were potentially significant based on p-values < 0.05, three of which were significant based on a Bonferroni corrected alpha value = 2.08E-4. Significant metabolites included glucose, taurine, and tyrosine. The PLS-DA scores plot (**Fig 12A**) produced statistically significant group separation (Mahalanobis distance = 2.31, F-statistic = 25.17, F-critical = 3.25). Cross-validation of the PLS-DA (**Fig 12B**) yielded R^2^ = 0.69 indicating moderately good data agreement with the model and Q^2^ = 0.40 indicating weak predictive power. PLS-DA indicated 81 VIP significant buckets, 19 of which could be identified, including asparagine, aspartate, cytidine, glucose, lactate, niacinamide, o-phosphocholine, phenylalanine, taurine, tyrosine, and xanthine. Concentrations and fold changes for identified metabolites are reported in **Table 17**. Potential importance of buckets that could not be identified was evaluated using volcano plot analysis (Figure AF in S1 File). Buckets that had p-values < 0.05 and fold changes greater than 2 are highlighted (colored green) in Figure AF in S1 File and listed in **Table 18**. One bucket at 5.398 ppm had a p-value < 0.05 but less than a two-fold changes in Figure AF in S1 File with a VIP score of 1.31, p-value of 3.95E-02 and AUC = 0.69. Intensity distribution plots for the two most significant buckets are shown in Figure AG in S1 File.

**Table 17:** Significant metabolites identified in pancreas tumor extracts of male and female study mice.

ppm	VIP	p-value	Identification			Rank		AUC
				Control Concentration mM (StDev)	Study Concentration mM (StDev)		Fold Change (Error)	
		
3.041	2.4	3.41E-04	tyrosine	2.16 (3.41)	2.28 (1.51)	4	2.465 (1.07)	0.83
3.934	1.35	1.30E-01	tyrosine	2.16 (3.41)	2.28 (1.51)	4	1.55 (0.60)	0.72
3.058	1.11	1.63E-01	tyrosine	2.16 (3.41)	2.28 (1.51)	4	1.60 (1.02)	0.73
3.237	1.87	3.44E-03	taurine	21.35 (20.00)	22.50 (14.35)	3	2.20 (1.17)	0.78
3.391	1.28	8.65E-02	taurine	21.35 (20.00)	22.50 (14.35)	3	1.68 (0.93)	0.71
3.465	1.80	8.46E-03	glucose	3.58 (2.93)	4.76 (2.95)	2	2.07 (1.03)	0.78
3.479	1.59	2.74E-02	glucose	3.58 (2.93)	4.76 (2.95)	2	1.90 (0.97)	0.76
3.493	1.40	5.92E-02	glucose	3.58 (2.93)	4.76 (2.95)	2	1.74 (0.91)	0.73
4.643	1.35	1.59E-02	glucose	3.58 (2.93)	4.76 (2.95)	2	1.46 (3.59)	0.76
3.201	1.46	7.45E-02	o-phosphocholine	1.80 (1.56)	1.90 (1.19)	1	1.68 (0.75)	0.72
7.838	1.40	1.66E-01	cytidine	1.23 (1.49)	0.34 (0.33)	2	-2.54 (0.50)	0.6
8.231	1.30	2.12E-01	niacinamide	0.70 (0.48)	0.37 (0.20)	1	-2.29 (0.60)	0.53
2.677	1.28	2.89E-01	aspartate	9.93 (11.26)	3.00 (1.88)	1	-1.83 (0.29)	0.63
2.661	1.07	4.27E-01	aspartate	9.93 (11.26)	3.00 (1.88)	1	-1.50 (0.31)	0.64
2.834	1.21	3.38E-01	asparagine	2.65 (2.82)	1.57 (0.86)	1	-1.69 (0.29)	0.64
7.849	1.21	2.12E-01	xanthine	2.06 (4.50)	undetected	1	-2.08 (0.72)	0.56
7.301	1.04	3.08E-01	phenylalanine	2.04 (3.10)	2.25 (1.55)	3	1.46 (0.60)	0.75
4.100	1.02	2.52E-01	lactate	10.00 (11.18)	11.38 (7.77)	3	1.44 (0.68)	0.72

**Table 18:** Significant unassigned buckets identified from the volcano plot analysis of pancreas tumor extract samples.

ppm	VIP	p-value	Fold Change (Error)	AUC
6.802	2.28	1.13E-04	3.58 (2.62)	0.82
5.580	1.78	1.37E-04	4.03 (4.43)	0.86
5.570	1.73	1.45E-04	4.76 (5.77)	0.86
5.589	1.72	3.06E-04	3.53 (3.70)	0.85
5.237	1.79	3.34E-03	2.27 (1.45)	0.79
7.766	1.48	7.46E-03	2.60 (3.16)	0.74
4.423	1.45	1.68E-02	2.33 (3.15)	0.73
4.399	1.35	2.42E-02	2.36 (4.18)	0.70
4.657	1.35	2.96E-02	2.87 (5.72)	0.78
5.288	1.33	3.28E-02	2.95 (6.01)	0.76
4.382	1.34	3.31E-02	2.07 (3.32)	0.68
4.558	1.36	3.40E-02	2.65 (5.06)	0.72
5.675	1.29	4.35E-02	3.49 (10.37)	0.71

### ROC analysis of pancreas tumor extracts

The highest accuracy biomarker distinguishing control and study tissues was tyrosine which had a maximum accuracy of 83%, VIP score of 2.4 and p-value of 3.41E-04 (**Table 16**). Other promising biomarkers included taurine and glucose, which reached accuracies as high as 78% (**Table 16**). Another handful of unidentified peaks had accuracies exceeding 80% and associated significant p-values on the order of 10^−4^ and VIP scores exceeding 1.7 **(Table 17)**.

### Identification of earliest urine metabolic profiling changes that predict mice with pancreatic tumors

**Fig 13:**
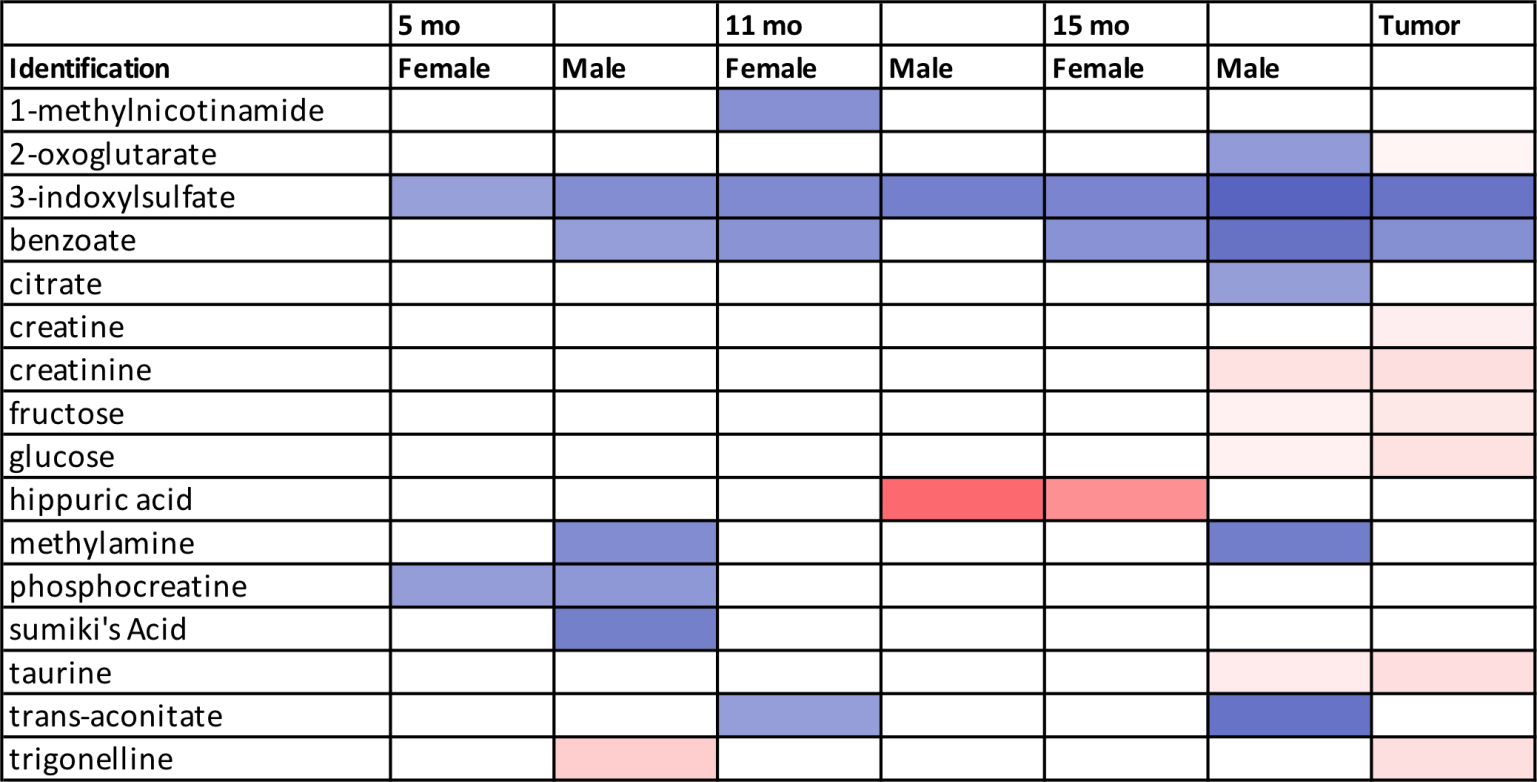
Heat map analysis of temporal changes in significant urine metabolite concentrations. Metabolites considered significant by either the p-value or VIP score for at least one age group comparison are included in the table. Red shading indicated a study/control fold change > 1, i.e. the metabolite concentration was higher in the study group, and these values were reported as positive fold changes. Blue shading indicated a study/control fold change < 1, i.e. the metabolite concentration was higher in the control group, and these values were reported as negative fold changes. Calculation of the shading intensities is described in the Materials and Methods section.

Based on the ROC analysis of the 15-month urine samples of male and female study mice, 3-indoxylsulfate and benzoate were the highest accuracy potential biomarkers common to both male and female mice. Given that the mice were generally in a complete precancerous PanIN phase at this stage, these biomarkers appear to be good urinary biomarkers of precancerous PanIN transformation accompanying acinar to ductal metaplasia. The question then becomes what is the earliest time point at which the biomarkers become detectable in the earlier stages of acinar to ductal metaplasia? To address this question, we evaluated urine samples of control and study mice from 5-month old mice (Figures AH–AO in S1 File) and 11-month old mice (Figures AP–AW in S1 File) using the analytical procedures described above for the 15-month old mice. We summarize the changes in metabolic profiles using a heat map representation (**Fig 13**). As can be seen in **Fig 13**, the presence of 3-indoxylsulfate is significantly decreased in the urine of both male and female study mice even as early as 5-months of age, making this a potentially useful biomarker to detect even the earliest PanIN stage in these mice. It is quite encouraging that 3-indoxylsulfate is also significantly decreased in the urine of mice that have established tumors, also indicated in **Fig 13**, especially since the urine samples were analyzed completely independently of the precancerous age group samples. Similarly, benzoate was significantly decreased in the urine of precancerous male and female mice at an age of 15 months, and decreased benzoate was also observed as early as 11-months of age in female mice (**Fig 13**) and was significantly decreased in male mice at 5-months of age. Again, as can be seen in **Fig 13**, benzoate was also significantly decreased in the urine of male and female mice that had undergone the transition from the precancerous PanIN state to having significant tumor burden. The consistent behavior in terms of decreased concentrations of benzoate in the urine at the earliest PanIN stages also indicates the potential value of benzoate as a biomarker of early PanIN stages that precede pancreatic cancer. Other changes in metabolite concentrations that were consistent with those observed in PanIN stages by not as dramatic included increases in urine concentrations of fructose, glucose, creatinine, taurine and trigonelline (**Fig 13**).

### Identification of earliest serum metabolic profiling changes that predict mice with pancreatic tumors

**Fig 14:**
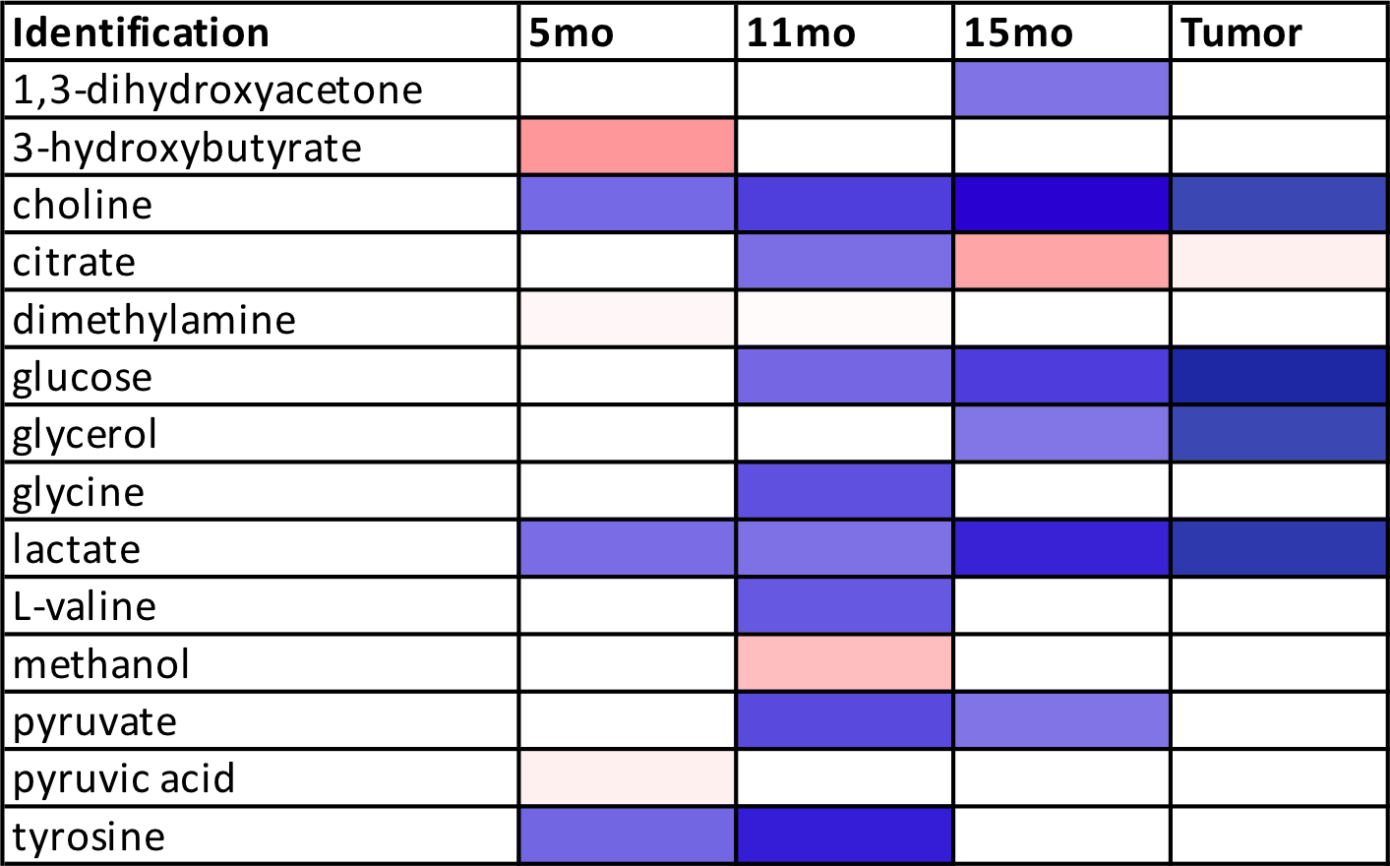
Heat map analysis of temporal changes in significant serum metabolite concentrations. Metabolites considered significant by either the p-value or VIP score for at least one age group comparison were included in the table. Red shading indicated a study/control fold change > 1, i.e. the metabolite concentration was higher in the study group, and these values were reported as positive fold changes. Blue shading indicated a study/control fold change < 1, i.e. the metabolite concentration was higher in the control group, and these values were reported as negative fold changes. Calculation of the shading intensities is described in the Materials and Methods section.

The serum samples of female control and study mice from 5-month old mice (Figures AX–BA in S1 File) and 11-month old mice (Figures BB–BE in S1 File) using the analytical procedures described above for the 15-month old mice. The ROC analysis indicate that the accuracy of glucose decreased concentrations in the blood reached as high as 91% accuracy in distinguishing between control and study mice. Inspection of the heat map for concentration changes of the significant metabolites (**Fig 14**) indicated that significant decreases in serum glucose concentration could be detected as early as 11-months of age. The fact that the serum glucose concentrations continued to strongly decrease in male and female mice with established tumors (**Fig 14**) indicate that detection of a growing decrease in serum glucose concentration could a potentially useful biomarker of formation of precancerous PanINs following by early stages of tumor formation. Choline was next in line reaching accuracies in the range of 85–90% for predicting mice belonging to the precancerous PanIN group. Inspection of the heat map in **Fig 14** indicates that significant decreased in serum choline could be detected even in 5-month old mice. Again, it is important that this trend was strongly maintained in the sera of mice that went on to form pancreatic tumors as can be seen in **Fig 14**, again indicating that detection of decreasing serum choline levels may a marker of the precancerous PanIN state and the trend continues up to the point the pancreatic tumors become established. Finally, the observed decreased levels of serum lactate had promising accuracies in the range 84–88% for predicting that mice had established the PanIN state. Decreased serum lactate levels were even more strongly apparent in mice that went on to form pancreatic tumors, indicating that detection of this pattern may be useful for early detection of the precancerous state preceding pancreatic cancer. Again, inspection of the heat map indicates that decreased levels of serum lactate could be detected as early as 5-months of age.

### Identification of earliest fecal metabolic profiling changes that predict mice with pancreatic tumors

**Fig 15:**
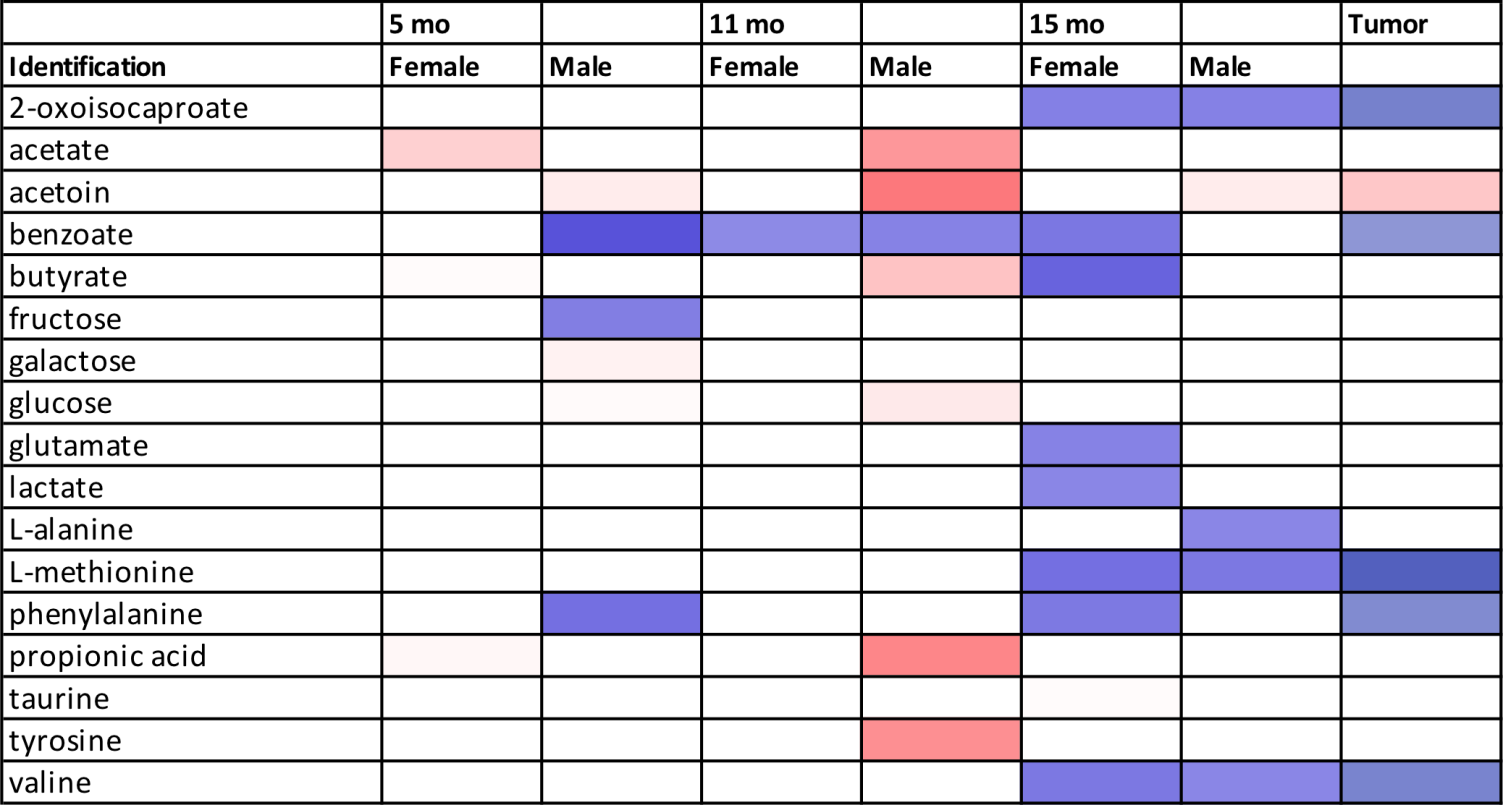
Heat map analysis of temporal changes in significant fecal metabolite concentrations. Metabolites considered significant by either the p-value or VIP score for at least one age group comparison were included in the table. Red shading indicated a study/control fold change > 1, i.e. the metabolite concentration was higher in the study group, and these values were reported as positive fold changes. Blue shading indicated a study/control fold change < 1, i.e. the metabolite concentration was higher in the control group, and these values were reported as negative fold changes. Calculation of the shading intensities is described in the Materials and Methods section.

Fecal samples of 5-month old female control and study mice (Figures BF–BI in S1 File), 11-month old female control and study mice (Figures BJ–BM in S1 File), 5-month old male control and study mice (Figures BN–BQ in S1 File) and 11-month old male control and study mice (Figures BR–BU in S1 File) were using the analytical procedures described above for the 15-month old mice. The ROC analysis indicated that phenylalanine was one of the highest accuracy biomarkers distinguishing control and study mice. The heat map analysis indicated that phenylalanine was detected in the 15-month female study mice, and can be detected as early as 5-months in the male study mice (**Fig 15**). This trend was consistent with decreased phenylalanine in the fecal extracts of male and female mice with established pancreatic tumors (**Fig 15**). The heat map analysis indicated that 2-oxoisocaproate was significantly decreased in 15-month old male and female mice, but this decrease was not significant in 5- and 11-month old mice. Albeit that the decrease in 2-oxoisocaproate in the fecal extracts was only detectable at 15 months of age, this trend persisted in a strong manner in male and female mice that had established pancreatic tumors (**Fig 15**). L-methionine and valine were also significantly decreased in the fecal extracts of male and female mice at 15 months of age, but not before, and this trend was strongly consistent with decreased L-methionine and valine observed in the fecal extracts of male and female mice with established pancreatic tumors (**Fig 15**). Benzoate was significantly decreased in mice ranging from 5-months to 15 months. Decreased benzoate in the fecal extracts of 15-month old mice was also found to have an accuracy of 72% to 77% by the ROC analysis, and decreased benzoate was also persistent in the fecal extracts of mice with established pancreatic tumors (**Fig 15**). Acetoin increased in the 15-month male study mice, and can be detected as early as 5-months in the male study mice, and increased acetoin was also observed in the fecal extracts of male and female mice with pancreatic tumors (**Fig 15**).

### Altered pathways in urine samples from study mice

**Fig 16:**
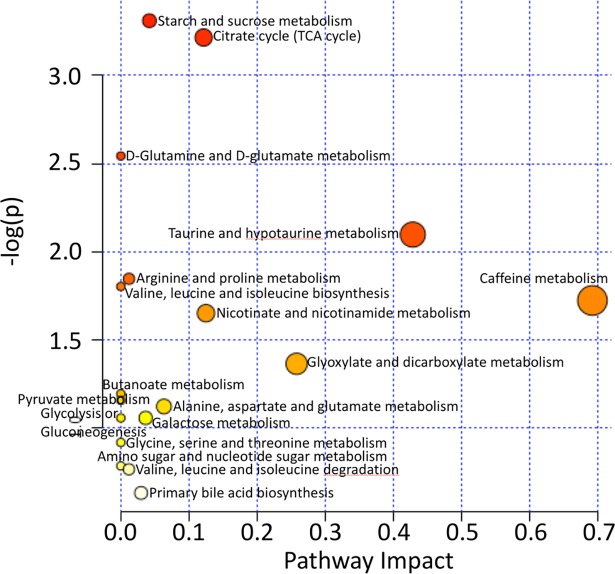
Pathway analysis from urine samples. The “metabolome view” from MetaboAnalyst 3.0 showing the pathway impact on the x-axis versus the negative log (p-values) on the y-axis for the metabolic pathways. Pathway names have been added.

Pathway analysis using the combined list of significant metabolites from all age and gender categories is shown in **Fig 16**. The top five pathways included starch and sucrose metabolism, citrate cycle (TCA cycle), D-Glutamine and D-glutamate metabolism, taurine and hypotaurine metabolism, and arginine and proline metabolism. **Table 19** displays the metabolic pathway and corresponding metabolites identified within that pathway.

**Table 19:** Identified pathways from Metaboanalyst 3.0 pathway analysis of the 5-, 11-, and 15-month female and male urine samples and the corresponding pathway-associated metabolites.

Pathway Name	Metabolites
starch and sucrose metabolism	beta-D-fructose, D-glucose
citrate cycle (TCA cycle)	oxoglutaric acid, citric acid
D-glutamine and D-glutamate metabolism	oxoglutaric acid
taurine and hypotaurine metabolism	taurine
arginine and proline metabolism	creatine, phosphocreatine
valine, leucine and isoleucine biosynthesis	4-methyl-2-oxopentanoate
caffeine metabolism	paraxanthine
nicotinate and nicotinamide metabolism	1-methylnicotinamide
glyoxylate and dicarboxylate metabolism	citric acid
butanoate metabolism	oxoglutaric acid
pyruvate metabolism	L-lactic acid
alanine, aspartate and glutamate metabolism	oxoglutaric acid
glycolysis or gluconeogenesis	L-lactic acid
galactose metabolism	D-glucose
glycine, serine and threonine metabolism	creatine
amino sugar and nucleotide sugar metabolism	beta-D-fructose
valine, leucine and isoleucine degradation	4-methyl-2-oxopentanoate
primary bile acid biosynthesis	taurine

### Interpretation of altered urine pathway relationships

The changes in urinary 3-indoxylsulfate, which decreased in the urine of 15-month study mice, was supported by VIP scores > 1.5, p-values on the order of 10^−5^ and accuracies ~90%, thus validating its importance for consideration as a potential biomarker distinguishing control and study mice. 3-indoxylsulfate in urine originates from degradation of tryptophan to indole by intestinal microbiota followed by microsomal oxidation to indoxyl and sulfonation in the liver followed by excretion [53]. Decreased 3-indoxylsulfate in the urine of 15-month old study mice suggests either a reduced gut microbial degradation of tryptophan due to gut microbial dysbiosis or reduced availability of dietary tryptophan due to reduced digestion associated with bicarbonate wasting [54].

Benzoate, which also decreased in the urine of 15-month study mice, was supported by VIP scores ranging from 1-2-1.4, p-values on the order of 10^−3^–10^−5^ and accuracies in the range 80–90%, also validating its importance for consideration as a potential biomarker distinguishing control and study mice. Benzoate levels in the urine reflect a balance of benzoate production in the intestine from gut bacterial deamination of phenylalanine [55] and consumption of benzoate in the body through conjugation with glycine to form hippurate [55], which occurs primarily in the kidney [56], but also occurs in the liver and intestines [56]. Microbial hydrolysis of hippurate is also a source of production of benzoic acid and glycine [57]. Altered urinary levels of benzoate are also known to report on abnormalities in gut microbial dysbiosis [55, 58]. Reduced benzoate levels detected in the urine of 15-month old study mice could either be due to gut microbial dysbiosis that lowers urinary benzoate production in the intestines. However, hippurate increased in the urine of 15-month old male and female study mice. As just mentioned, hippurate is formed via conjugation of benzoate with glycine primarily in the kidney, liver and intestines [56]. On the one hand, the amount of hippurate conjugated from benzoate depends on the amount of available benzoate taken up across the intestinal epithelia. Increased hippurate and decreased benzoate in the urine of 15-month study mice could reflect efficient conjugation of available benzoate in the kidney, which effectively depletes benzoate excretion. Essentially, the benzoate supply lags behind hippurate synthesis. Again, the lag in benzoate supply could be caused as a side effect of biocarbonate wasting which leads to gut microbial dysbiosis that results in reduced benzoate production by gut microbes [57].

Citrate also decreased significantly in the urine of 15-month female and male study mice compared to control mice having accuracies in the range 75–77% in the female group. Low levels of citrate in the urine, a condition known as hypocitraturia, can be caused by bowel dysfunction [59]. Intestinal malabsorption syndrome is a form of bowel dysfunction associated with low urinary citrate excretion [60] that has been linked to bicarbonate wasting [59, 61, 62]. Bicarbonate wasting occurs when bicarbonate is no longer secreted at adequate levels by the pancreas. The normal healthy pancreas is known to secrete high concentrations of bicarbonate to neutralize the highly acidic environment of the stomach to preserve the function of digestive enzymes [63]. Bicarbonate wasting can be caused by loss of pancreas tissue [64] leading to reduced digestion, nutrient absorption and gastrointestinal malabsorption [54]. The appearance of the pancreas sections in the 15-month study mice indicates virtually total loss of normal acinar tissue, consistent with loss of pancreas tissue that would cause bicarbonate wasting. Bicarbonate wasting also causes acidification of the pancreatic juice that can cause premature activation of proteases inside the pancreas and development of pancreatitis [54]. The pancreas sections of the 15-month study mice strongly resemble the morphology of a pancreas exhibiting chronic pancreatitis [65]. Bicarbonate wasting and resulting acidification of the pancreatic juice decreases its antimicrobial activity which can also cause gut microbial dysbiosis.

### Altered pathways in serum samples from study mice

**Fig 17:**
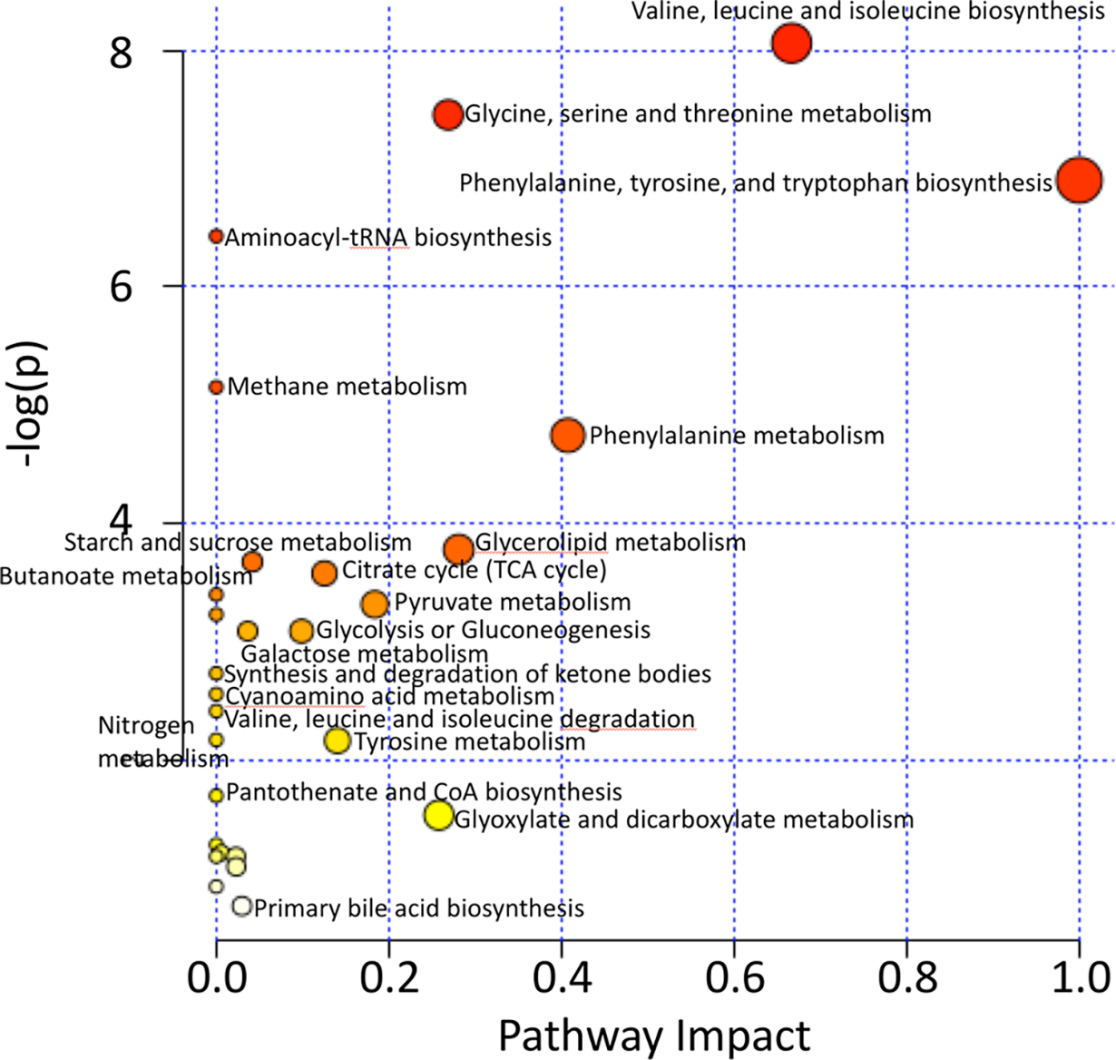
Pathway analysis from female serum samples. The “metabolome view” from MetaboAnalyst 3.0 showing the pathway impact on the x-axis versus the negative log (p-values) on the y-axis for the metabolic pathways. Pathway names have been added.

Pathway analysis for metabolic profiling changes measured in sera from a combination of all age categories and their given metabolites identified is shown in **Fig 17**. The top five pathways were valine, leucine, and isoleucine biosynthesis, glycine, serine, and threonine metabolism, phenylalanine, tyrosine, and tryptophan biosynthesis, aminoacyl-tRNA biosynthesis, and methane metabolism. **Table 20** displays the metabolic pathway and corresponding metabolites identified within that pathway.

**Table 20:** Pathways identified from analysis of serum samples and corresponding pathway-associated metabolites.

Pathway Name	Metabolites
pyruvate metabolism	pyruvic acid, L-lactic acid
starch and sucrose metabolism	beta-D-fructose, D-glucose
glycolysis or gluconeogenesis	pyruvic acid, L-lactic acid
primary bile acid biosynthesis	glycine
porphyrin and chlorophyll metabolism	glycine
galactose metabolism	D-glucose, glycerol
tyrosine metabolism	L-tyrosine, p-hydroxyphenylacetic acid
valine, leucine and isoleucine degradation	L-valine, L-isoleucine
glycine, serine and threonine metabolism	choline, betaine, glycine, pyruvic acid
glutathione metabolism	glycine
glyoxylate and dicarboxylate metabolism	citric acid
phenylalanine metabolism	L-phenylalanine, L-tyrosine
phenylalanine, tyrosine and tryptophan biosynthesis	L-phenylalanine, L-tyrosine
valine, leucine and isoleucine biosynthesis	L-valine, L-isoleucine, pyruvic acid
aminoacyl-tRNA biosynthesis	L-phenylalanine, glycine, L-valine, L-isoleucine, L-tyrosine
synthesis and degradation of ketone bodies	(r)-3-hydroxybutyric acid
butanoate metabolism	(r)-3-hydroxybutyric acid, pyruvic acid
amino sugar and nucleotide sugar metabolism	beta-D-fructose
ubiquinone and other terpenoid-quinone biosynthesis	L-tyrosine
cysteine and methionine metabolism	pyruvic acid
alanine, aspartate and glutamate metabolism	pyruvic acid
glycerolipid metabolism	dihydroxyacetone, glycerol
cyanoamino acid metabolism	glycine
glycerophospholipid metabolism	choline
citrate cycle (TCA cycle)	citric acid, pyruvic acid
pantothenate and CoA biosynthesis	L-valine
nitrogen metabolism	glycine
methane metabolism	glycine, methanol

### Interpretation of altered serum pathway relationships

The most significant change in the serum metabolic profiles in 15-month study mice based was decreased concentrations of glucose (VIP scores 1.12–2.37, p-values 10^−2^–10^−7^, and accuracies 67%–91%). Decrease blood glucose was consistent with reduced digestion of consumed food resulting in lower absorption of glucose into the blood stream. Reduced digestion of consumed food would be caused either by loss of digestive enzyme activity associated with loss of acinar tissue mass, loss of digestive enzyme activity associated with reduced neutralization of the acidic environment of the gut caused by reduced bicarbonate secretion, and altered gut microbial contribution to digestion associated with gut microbial dysbiosis caused by bicarbonate wasting.

The next most significant serum metabolite was choline, which decreased in serum of 15-month study mice (VIP scores 2.1–2.2, p-values 10^−5^, accuracies 85–89%). Choline is an essential nutrient that is present in some foods. The body needs choline to synthesize phosphatidylcholine and sphingomyelin, two major phospholipids vital for cell membranes, and therefore animal cells require choline to preserve structural integrity [66]. The most common sources of choline in foods are fat-soluble phospholipids, water soluble phosphocholine and glycerolphosphocholine, and free choline [66]. When these compounds are ingested, pancreatic and mucosal enzymes liberate free choline from some of the fat-soluble and water-soluble ingested forms [67]. Reduced serum choline levels in 15-month study mice are consistent with reduced digestion of ingested choline derivatives caused by inhibited digestive enzymes associated with bicarbonate wasting.

Lactate decreased in sera of 15-month study mice (VIP scores 1.82–2.12, p-values 10^−4^–10^−5^, accuracies 84%–88%). Blood lactate levels originate as a product of anaerobic glycolysis blood lactate levels in normal ranges reflect a homeostatic level of glycolytic activity in cells. During periods of intense exercise or in the presence of certain diseases, blood lactate levels are known to increase, for example it is known that pancreatitis can cause of metabolic acidosis that can increase blood lactate levels [68]. Since normal blood lactate levels reflect a normal homeostatic level of activity and associated glycolytic activity, it is possible that the reduced blood lactate in 15-month-old mice reflected a below normal glycolytic activity associated with reduced activity in the 15-month-old study group in comparison to the control group. Interestingly, lactate levels decreased in the pancreata of rats with chronic pancreatitis [69].

### Altered pathways in fecal extracts from study mice

**Fig 18:**
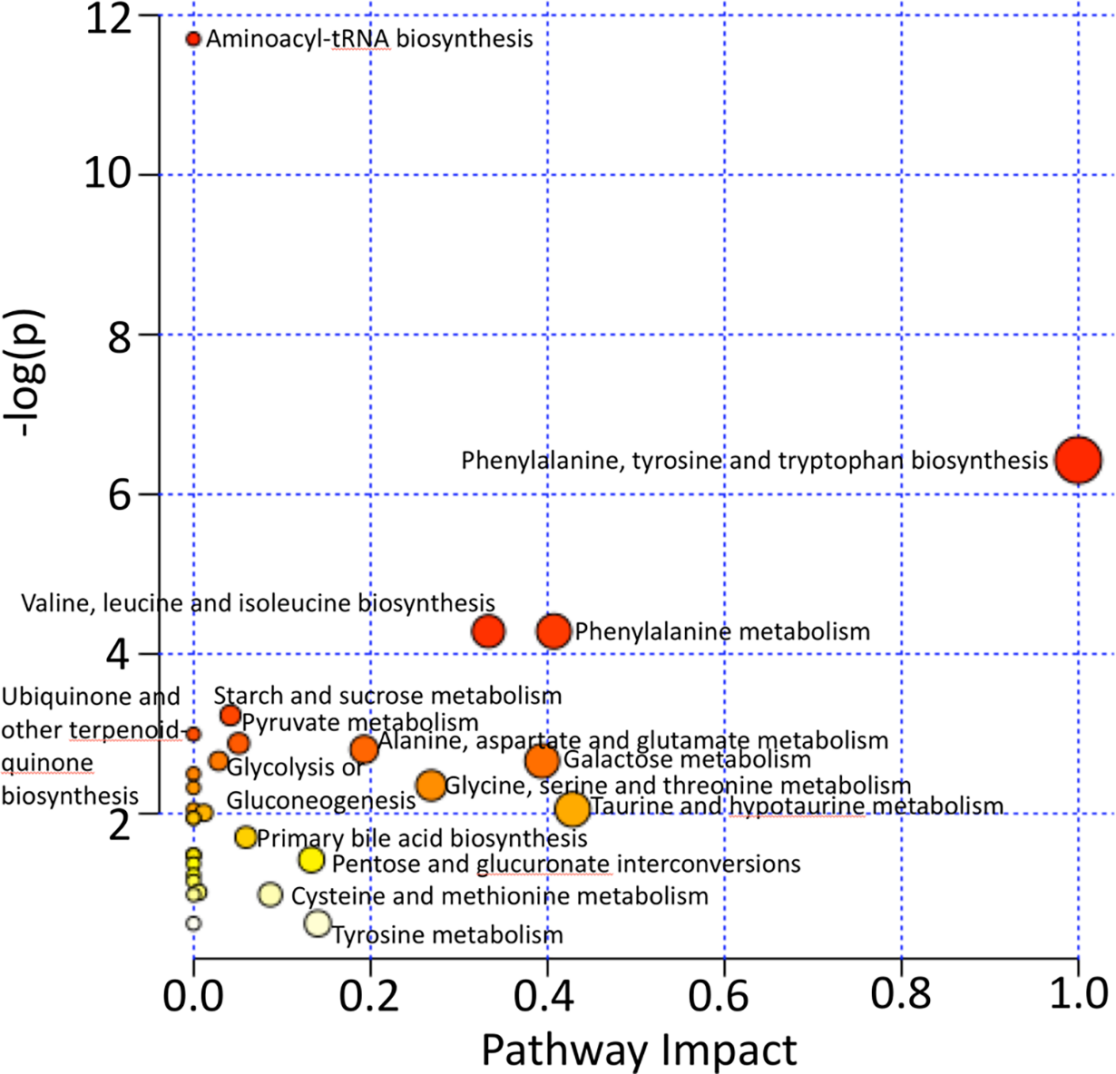
Pathway analysis from fecal samples. The “metabolome view” from MetaboAnalyst 3.0 showing the pathway impact on the x-axis versus the negative log(p-values) on the y-axis for the metabolic pathways. Pathway names have been added.

Pathway analysis for all combined age (5-, 11-, and 15-months) and gender (female and male) groups is shown in **Fig 18**. The top five pathways identified were aminoacyl-tRNA biosynthesis, phenylalanine, tyrosine, and tryptophan biosynthesis, phenylalanine metabolism, valine, leucine, and isoleucine biosynthesis, and starch and sucrose metabolism. **Table 21** lists the metabolic pathway and corresponding metabolites identified within that pathway.

**Table 21:** Pathways identified from 5-, 11-, and 15-month female and male fecal samples and the pathway-associated metabolites.

Pathway Name	Metabolites
tyrosine metabolism	L-tyrosine
taurine and hypotaurine metabolism	taurine
galactose metabolism	D-galactose, D-glucose
amino sugar and nucleotide sugar metabolism	D-galactose, beta-D-fructose
pantothenate and CoA biosynthesis	L-valine
starch and sucrose metabolism	beta-D-fructose, D-glucose
beta-alanine metabolism	L-aspartic acid
ubiquinone and other terpenoid-quinone biosynthesis	L-tyrosine
aminoacyl-tRNA biosynthesis	L-phenylalanine, glycine, L-aspartic acid, L-methionine, L-valine, L-alanine, L-threonine, L-tyrosine
valine, leucine and isoleucine biosynthesis	L-valine, 4-methyl-2-oxopentanoate
histidine metabolism	L-aspartic acid
pyruvate metabolism	L-lactic acid, acetic acid
primary bile acid biosynthesis	glycine, taurine
pentose and glucuronate interconversions	D-xylose
methane metabolism	glycine
glycolysis or Gluconeogenesis	L-lactic acid, acetic acid
glutathione metabolism	glycine
Arginine and proline metabolism	L-aspartic acid
phenylalanine metabolism	L-phenylalanine, L-tyrosine
glycine, serine and threonine metabolism	glycine, L-threonine
nitrogen metabolism	glycine
porphyrin and chlorophyll metabolism	glycine
phenylalanine, tyrosine and tryptophan biosynthesis	L-phenylalanine, L-tyrosine
valine, leucine and isoleucine degradation	L-valine, 4-methyl-2-oxopentanoate
propanoate metabolism	propionic acid
butanoate metabolism	butyric acid
selenoamino acid metabolism	L-alanine
cysteine and methionine metabolism	L-methionine
alanine, aspartate and glutamate metabolism	L-aspartic acid, L-alanine
D-glutamine and D-glutamate metabolism	D-glutamic acid
cyanoamino acid metabolism	glycine

### Interpretation of altered pathway relationships in fecal extracts

One of the most significant changes in the metabolic profiles of fecal extracts in 15-month study mice was decreased phenylalanine in both male and female mice (VIPs ranging from 0.94–1.65, p-values 10−1–10^−4^, and accuracies 54%-84%). Digestion of dietary proteins provides a source of amino acids in the intestine. One explanation for reduced phenylalanine in fecal extracts would be reduced enzymatic digestion of proteins into their free amino acid building blocks caused by inactivation of digestive enzymes due to bicarbonate wasting. One fate of phenylalanine generated by digestion of dietary protein can be absorption across the intestine as a nutrient. Phenylalanine is also known to be degraded in the intestine by gut microbiota [70] by deamination producing benzoate. However, benzoate levels in the fecal extracts were also lower than in controls, suggesting that a reduced supply of phenylalanine due to reduced enzymatic digestion of dietary protein was a better explanation of lower phenylalanine levels rather than elevated gut microbial degradation of dietary derived phenylalanine associated with gut microbial dysbiosis.

Among the most significant changes in the metabolic profile in the fecal extracts of 15-month male and female study mice was increased acetoin (VIP ranging from 1.1–1.7, p-values on the order 10^−2^, and accuracies ranging from 60%–70%). Acetoin, also known as 3-hydroxybutanone, is used as an external energy store by many fermentative bacteria and is an important physiological metabolite excreted by many microorganisms [71]. It is natural that we would find acetoin in the fecal extracts given the abundance of microbes in the mouse gut. The increase in acetoin in 15-month study mice again indicates increased gut microbial secretion of acetoin associated with an altered gut microbiome.

Benzoate significantly decreased in 15-month fecal extracts from female study mice (VIP ranging from 0.84–1.19, p-values on the order 10^−2^–10^−3^, and accuracies ranging from 72%–77%). Benzoate is primarily produced in the intestine from gut bacterial deamination of phenylalanine [55]. Phenylalanine occurs as a product of protein digestion in the intestines. There was no detected decrease in dietary consumption over the course of the study. Therefore, reduced benzoate production in the feces was consistent with reduced deamination of phenylalanine by gut microbes in the intestines. This result is also consistent with the reduced levels of phenylalanine observed in the fecal extracts (see above). This result was also consistent with the reduced levels of benzoate observed in the urine of 15-month study mice. All of these observations could be explained by reduced enzymatic digestion of dietary protein into individual amino acids caused by inactivation of digestive enzymes caused by bicarbonate wasting.

### Altered pathways in pancreas tissue extracts from study mice

**Fig 19:**
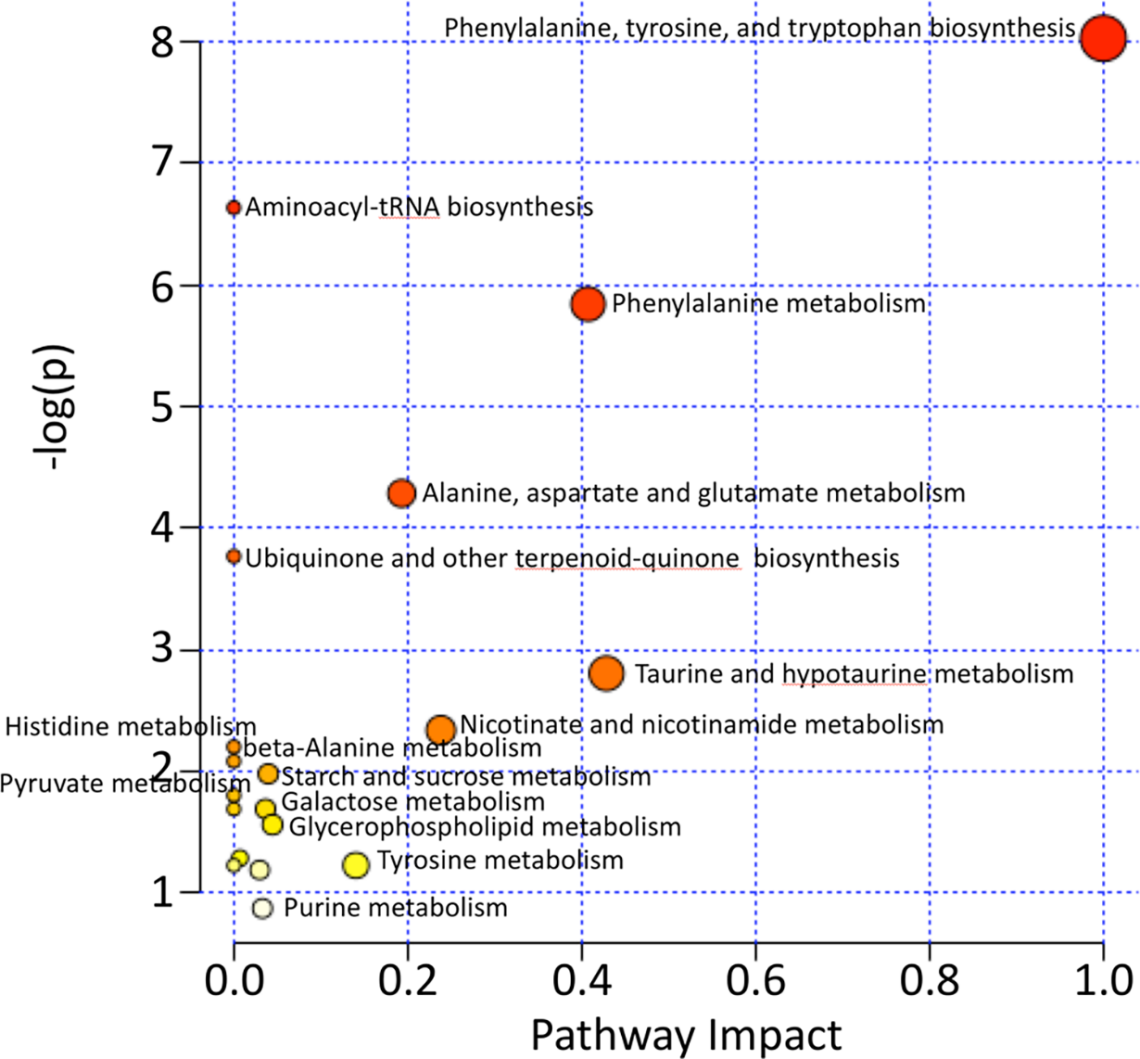
Pathway analysis from pancreas tissue samples. The “metabolome view” from MetaboAnalyst 3.0 showing the pathway impact on the x-axis versus the negative log(p-values) on the y-axis for the metabolic pathways. Pathway names have been added.

Pathway analysis indicated 19 metabolic pathways (**Fig 19**). The top five pathways were phenylalanine, tyrosine, and tryptophan biosynthesis, aminoacyl-tRNA biosynthesis, phenylalanine metabolism, alanine, aspartate and glutamate metabolism, ubiquinone and other terpenoid-quinone biosynthesis. The complete list of metabolic pathways and corresponding metabolites identified within that pathway are included in **Table 22**.

**Table 22:** Pathways altered in pancreatic tissue samples and the corresponding pathway-associated metabolites.

Pathway Name	Metabolites
glycolysis or gluconeogenesis	L-lactic acid
purine metabolism	xanthine
pyruvate metabolism	L-lactic acid
nicotinate and nicotinamide metabolism	niacinamide
alanine, aspartate and glutamate metabolism	L-aspartic acid, L-asparagine
glycerophospholipid metabolism	phosphorylcholine
primary bile acid biosynthesis	taurine
beta-alanine metabolism	L-aspartic acid
galactose metabolism	D-glucose
pyrimidine metabolism	cytidine
tyrosine metabolism	L-tyrosine
phenylalanine, tyrosine and tryptophan biosynthesis	L-phenylalanine, L-tyrosine
aminoacyl-tRNA biosynthesis	L-asparagine, L-phenylalanine, L-aspartic acid, L-tyrosine
arginine and proline metabolism	L-aspartic acid
taurine and hypotaurine metabolism	taurine
histidine metabolism	L-aspartic acid
starch and sucrose metabolism	D-glucose
phenylalanine metabolism	L-phenylalanine, L-tyrosine
ubiquinone and other terpenoid-quinone biosynthesis	L-tyrosine

### Interpretation of altered pathway relationships in pancreas tissue extracts

The most significant difference between the metabolic profiles of normal acinar pancreas tissue extracts and extracts of pancreata from 15-month study mice was increased tyrosine (VIP ranging from 1.1–2.4, p-values on the order 10^−1^–10^−4^, and accuracies ranging from 72%–83%). Amino acids are normally imported as monomers to support cell growth, but alternative routes can include uptake of proteins via micropinocytosis and subsequent lysosomal degradation of the proteins as a source of amino acids [72]. A simple relationship between the disease state of the pancreas and the concentrations of free amino acids in the tissue is not clear, as it has been reported that in pancreatic cancer, the tumor tissue deficient in glutamine and serine, but accumulated essential amino acids [72]. Since the PanIN present in the study mice tissue represents a precancerous state that resembles that of chronic pancreatitis, it is impossible to relate the alteration of tyrosine levels in the PanIN tissues to that present in pancreas tumor tissue.

Taurine levels were also significantly elevated in 15-month pancreas tissue extracts from study mice (VIP ranging from 1.28–1.87, p-values on the order 10^−2^–10^−3^, and accuracies ranging from 71%–78%). This result is consistent with elevated levels of taurine reported in early pancreatic cancer using high resolution magic angle spinning to probe pancreatic tissue samples taken from the same mouse model studied here [73]. In this same study, lactate levels were also reported to be elevated in the study mice tissues, consistent with elevated lactate observed in 15-month study mice pancreas tissue extracts reported here.

Glucose levels were increased in the pancreatic tissue of 15-month-old mice (VIP ranging from 1.35–1.80, p-values on the order 10^−2^–10^−3^, and accuracies ranging from 73%–78%). One possible explanation for the increased glucose levels in the PanIN tissue was reduced consumption of glucose by PanIN tissue cells in comparison to the normal functioning acinar cells that dominate the healthy pancreas.

## Conclusions

This study represents one of the largest systematic and comprehensive histological and metabolic profiling investigations of the most well characterized mouse model of pancreatic cancer, namely the Ptf1a-Cre; LSL-KrasG12D transgenic mouse model, reported to date. Aspects of the study that stand out include the substantial range of mouse ages, from 5-months to 16-months, included in the study, and the strong statistical power supporting the comparisons of the age-matched and gender-matched samples over the entire age range. The fact that the exact status of the pancreas was established by histological analysis for every mouse from which samples were collected for metabolic profiling also increased the power of the study.

One of the most remarkable results is the dramatic transformation that takes place in the pancreas morphology as the study mice age, starting from a healthy normal looking pancreas and changing to a morphology characterized by complete substitution of healthy acinar tissue by a universal PanIN morphology [33] by 15–16 months of age. It is well established that the morbidity of pancreatic cancer is strongly associated with the type of pancreatic cancer, e.g. endocrine versus exocrine [9], and that histological analysis plays a critical role in the diagnosis of the type of pancreatic cancer [9, 33]. While the histological analysis of each pancreas matched with urine, fecal and blood samples was originally intended to enable a precise staging of the disease in each mouse, it became apparent early on in the study that it would be difficult to assign precise percentages of each PanIN stage to each mouse, since large fractions of each pancreas were generally transformed into what we broadly refer to as the PanIN state, and that generally all PanIN stages were observed in each tissue actively undergoing transformation. Indeed, many of the histological features observed in the pancreata were difficult or impossible to classify using the existing PanIN definitions, which is an issue that we have addressed in a separate publication [33]. Part of the difficulty in making PanIN staging assignments using the original classification scheme [74, 75] originates from the assumption that PanINs represent a deformation or transformation of an existing pancreatic duct into a PanIN, whereas our data reported in this paper and in our previous work, as well as that of others [76], illustrates that rather than PanINs being derived from individuals duct, there is a wholesale replacement of acinar tissue with structures that take on the appearance of PanINs. These structures, while still classified as PanINs, appear to be largely derived from acinar-to-ductal metaplasia [76], and so many of the histological features observed in the pancreata sections defy a simple classification using the existing and recognized PanIN stages. Rather, the structures involved in acinar-to-ductal metaplasia seem more appropriately defined by the model more recently introduced by Chuvin et al. [76] than the PanIN-1 through PanIN-3 convention that has long been used [74, 75]. Therefore, interpretation of the metabolic profiling results reported here is more appropriate in the context of the more recent PanIN terminology associated with acinar-to-ductal metaplasia [76].

Another preliminary observation that may be relevant to interpretation of these data is the status of the endocrine function of the pancreas in the presence of acinar to ductal metaplasia. Based on a preliminary histological of endocrine function using fluorescent antibodies to detect insulin and glucagon production by beta and alpha cells, respectively, we were able to detect the presence of both functioning alpha and beta cells even in the presence of mostly PanIN tissue, albeit that the islets appeared to be disorganized, no longer spherical, and the islet cells appeared to be organizing along the PanIN epithelial cells. While these preliminary observations indicate persistent pancreatic endocrine function even in mice that had fully transitioned to a PanIN phenotype, a more thorough investigation of the endocrine function in these mice will be the subject of a future investigation.

While the morphological changes in the pancreata of the study mice were remarkable, the pancreas transformation proceeded to adenocarcinoma in only about 50 of the study mice. This means that metabolic profiling changes reported in this study largely reflect changes associated with the transformation of the healthy pancreas to the precancerous PanIN state. The morphology of the transformed pancreata of the study mice strongly resembles that of human patients diagnosed with chronic pancreatitis [65]. It is perhaps remarkable that the mice continue to thrive after the entire pancreas has undergone a transformation to a PanIN morphology that is devoid of acinar tissue. Given the well-known function that the acinar cells secrete critical digestive enzymes along with a substantial amount of bicarbonate to prevent denaturation of the digestive enzymes by neutralizing the highly acidic environment of the stomach, it is perhaps not surprising that the metabolic profiling changes that were observed could largely be explained as being caused by, or a consequence of, bicarbonate wasting due to loss of bicarbonate production. Given the dramatic gross morphological changes observed in the pancreas tissue, it was surprising that more dramatic changes in the metabolic profiles of the urine and blood from these animals were not observed. Nonetheless, the statistical power allowed detection of significant changes in the metabolic profiles of each of the biological fluids analyzed, and because of the longitudinal nature of the study, it was possible to identify changes in the metabolic profiles that were present in male and female mice that had established pancreatic tumors, and to look backwards in time to younger mice with a smaller PanIN burden to determine the earliest time at which the particular metabolic biomarkers were useful in predicting the precancerous PanIN state. The absence of more dramatic changes in the metabolic profiles of these biofluids may be an indicator of at least one reason why it has been so difficult to identify biomarkers for early detection of pancreatic cancer. Indeed, the absence of visible signs of distress in the mice as their pancreata progressed towards a complete transformation to the precancerous PanIN state may be representative of the indolent state of the disease of pancreatic cancer in asymptomatic humans, or at least its precancerous PanIN phase, which is insidious in that the indolent and asymptomatic state remains impossible to detect using any existing biomarkers.

The substantial statistical power provided by the experimental design was leveraged by employing multiple independent methods to validate the significance of the metabolic changes including p-values, VIP scores, and accuracies. Our confidence in identifying important metabolic changes in the study group was increased when a putative metabolite was strongly supported by all three of these metrics. Focusing on the three biofluids that can be obtained by minimally invasive means, i.e. urine, blood, and feces, we were able to identify the following putative biomarkers: decreased 3-indoxylsulfate, benzoate and citrate in urine, decreased glucose, choline, and lactate in blood, and decreased phenylalanine and benzoate and increased acetoin in fecal extracts. Again, it should be emphasized that these changes in the metabolic profiles were independently validated in these biofluids from mice that had established pancreatic tumors, and then it was possible to confirm not only that these same changes occurred in male and female mice in the precancerous PanIN stages, but it was also possible to determine at what age it was possible to detect the change the putative biomarker. It should be also noted that some of the top scoring peaks in the NMR spectra remained unidentified. However, we subjected all of the unidentified peaks to the same rigor of analysis and reported all of the analysis either in the main body of the manuscript or within the supporting supplementary material. This information should be valuable for comparison with future studies allowing for confirmation of important changes that occurred within the NMR spectra of the study mice, but that could not be associated with a specific metabolite at this time. Due to the nature of the design study, the important metabolites and NMR resonances were only identified post-hoc, and therefore it was not possible to conduct a time-dependent modeling analysis regarding these potential biomarkers, however, such an investigation would be valuable to further validate these biomarkers in a future study.

As with any study, there were certain limitations of this study. For example, we were limited to studying a transgenic mouse model of pancreatic cancer, whereas the obvious application is intended to be applied to spontaneous human cancers. It is equally obvious that it is impossible to conduct a comprehensive longitudinal study of pancreatic cancer, or for that matter, progression of the precancerous phase that occurs prior to adenocarcinoma, in a human study. So, while this limitation exists in our study, it is insurmountable given that it is not possible to conduct a comparable study in humans. Notwithstanding, the results from this study have led to identification of certain metabolic profiling patterns that can be investigated in human patients for further validation when appropriate samples are available.

As far as future work is concerned, this study was also limited in that only NMR based metabolic profiling was conducted. An obvious next step could be to expand to technology to include LC/MS based profiling to complement the information established from the NMR based data analysis. Another aspect that requires addition research is to complete the identification of the peaks that showed great promise as potential biomarkers, but remain unidentified.

An intriguing aspect of our study is that the results report on the precancerous phase prior to initiation of adenocarcinoma. This means that the putative metabolic biomarkers could potentially be useful for identification of a precancerous stage of pancreatic cancer progression in humans. Given the promising accuracy of the biomarkers identified in this study, one can imagine a future diagnostic metabolic profiling-based test that assays for a combined analysis of urine, blood, and feces that could potentially be used identify individuals at risk for developing pancreatic cancer prior to the disease.

In closing, improving the prognosis for pancreatic cancer patients is obviously a multifaceted problem with many challenges. One important requirement to achieving this goal is that much more effective means of treating pancreatic cancer once it has been detected are urgently needed. However, given the significant improvement in survival associated with catching the disease when it can still be treated by surgical resection, there must also be a persistent effort to discover more effective means for early detection of the disease. The results reported in this manuscript demonstrate that metabolic profiling results obtained from multiple biological fluids obtained non-invasively can be used to identify biomarkers with high accuracy for identifying the precancerous stage of pancreatic cancer. The next challenge is to design an experimental study in humans that can be used to confirm the results obtained from this study.

## Supporting information

S1 File This supplementary material file contains 73 supplementary figures labeled from Fig A to Fig BU. (PDF)

S2 File This supplementary material file contains five supplementary tables. (PDF)
