# Supplementary material for: NMR-based metabolic profiling of urine, serum, fecal, and pancreatic tissue samples from the Ptf1a-Cre; LSL-KrasG12D transgenic mouse model of pancreatic cancer
Source: PLoS One. 2018 Jul 17;13(7):e0200658. doi: 10.1371/journal.pone.0200658 (PMC6049928; doi:10.1371/journal.pone.0200658)
Supplement: S1 File — (PDF) [file pone.0200658.s001.pdf]

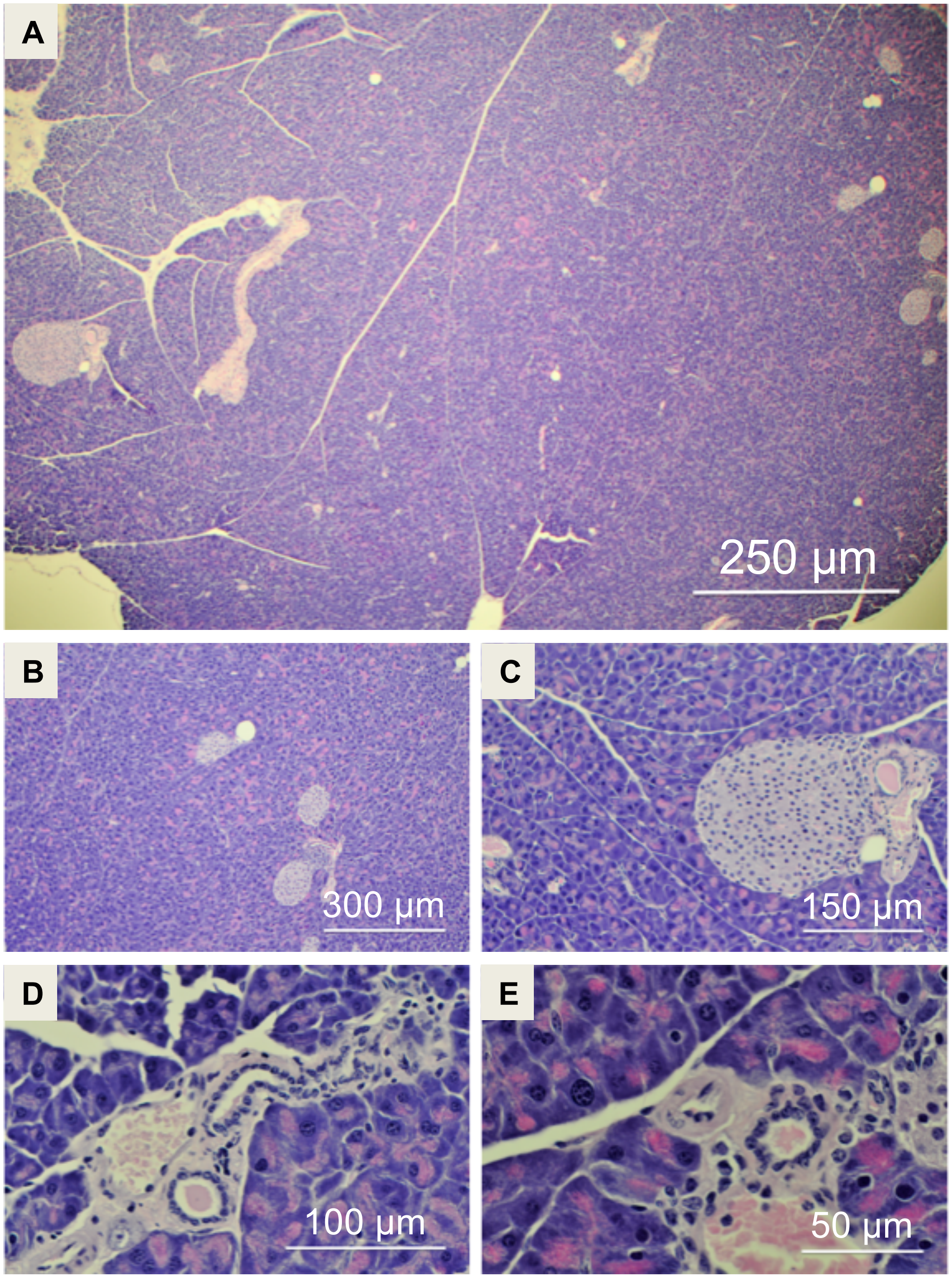

Fig A. Representative Hematoxylin and Eosin Stained Images from an 11-Month Old Female Control Mouse. (A) Magnification at 4X. (B) Magnification at 10X. (C) Magnification at 20X. (D) Magnification at 40X. (E) Magnification at 60X.

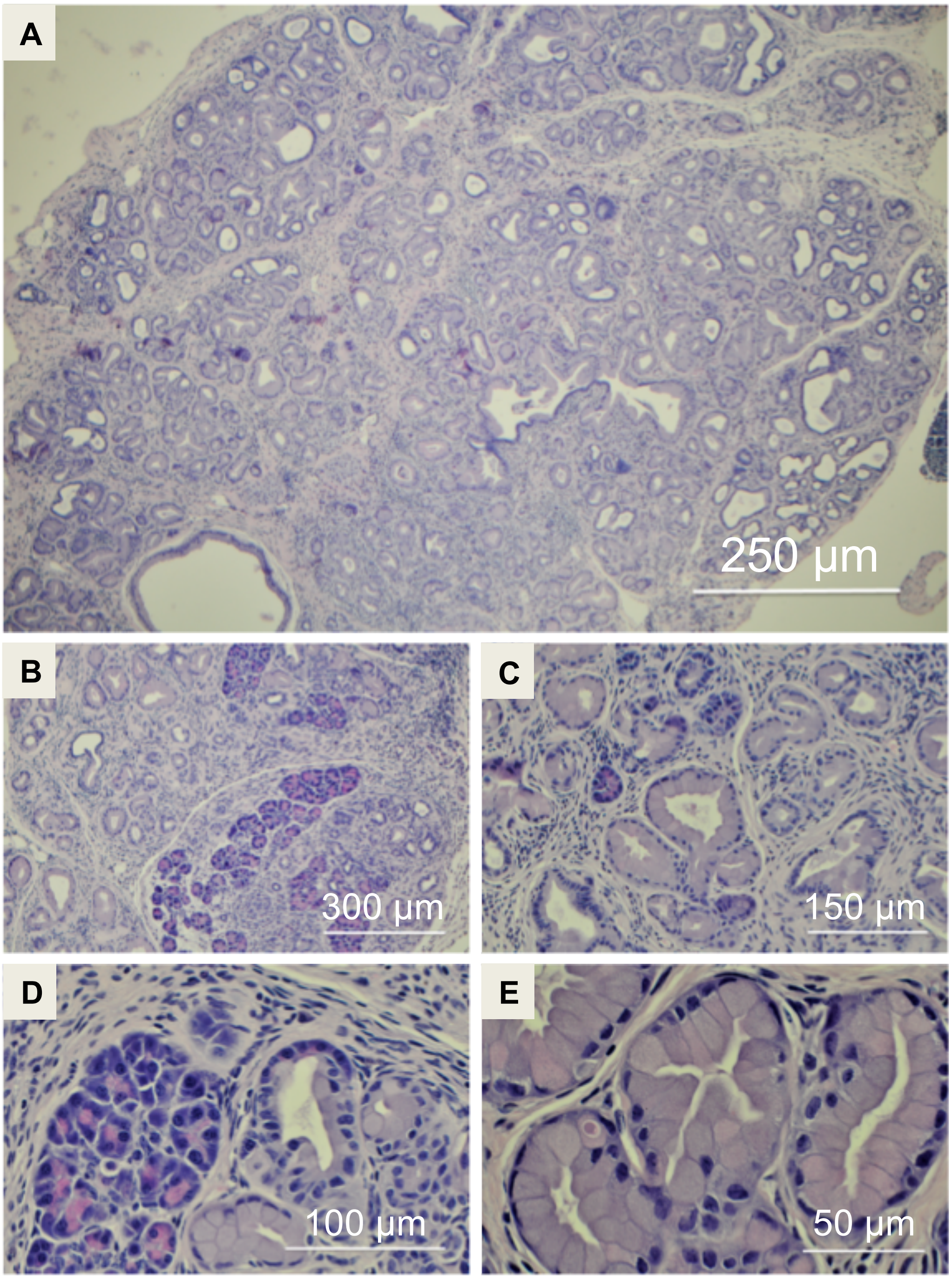

Fig B. Representative Hematoxylin and Eosin Stained Images from an 11-Month Old Female Study Mouse. (A) Magnification at 4X. (B) Magnification at 10X. (C) Magnification at 20X. (D) Magnification at 40X. (E) Magnification at 60X.

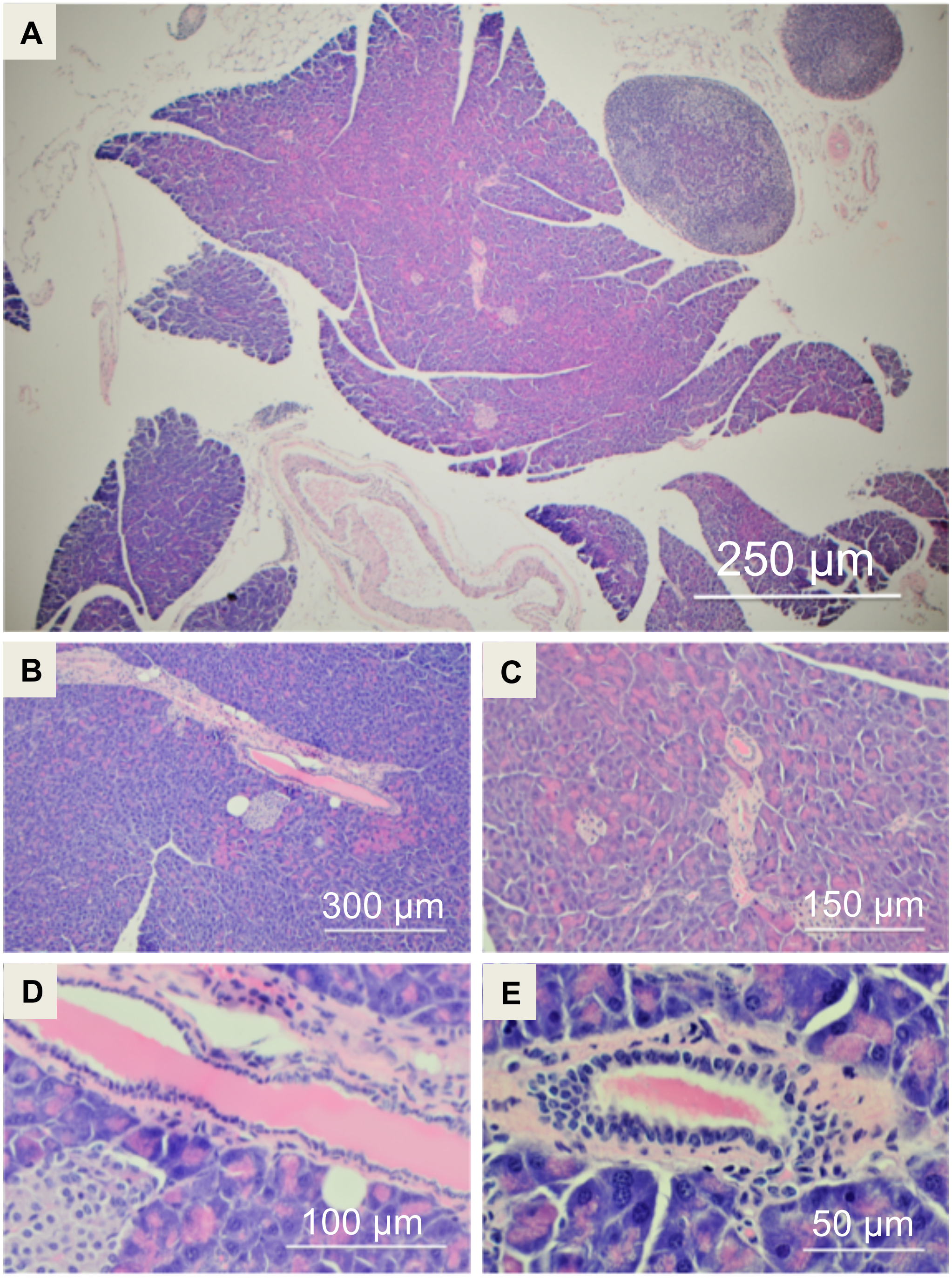

Fig C. Representative Hematoxylin and Eosin Stained Images from a 5-Month Old Female Control Mouse. (A) Magnification at 4X. (B) Magnification at 10X. (C) Magnification at 20X. (D) Magnification at 40X. (E) Magnification at 60X.

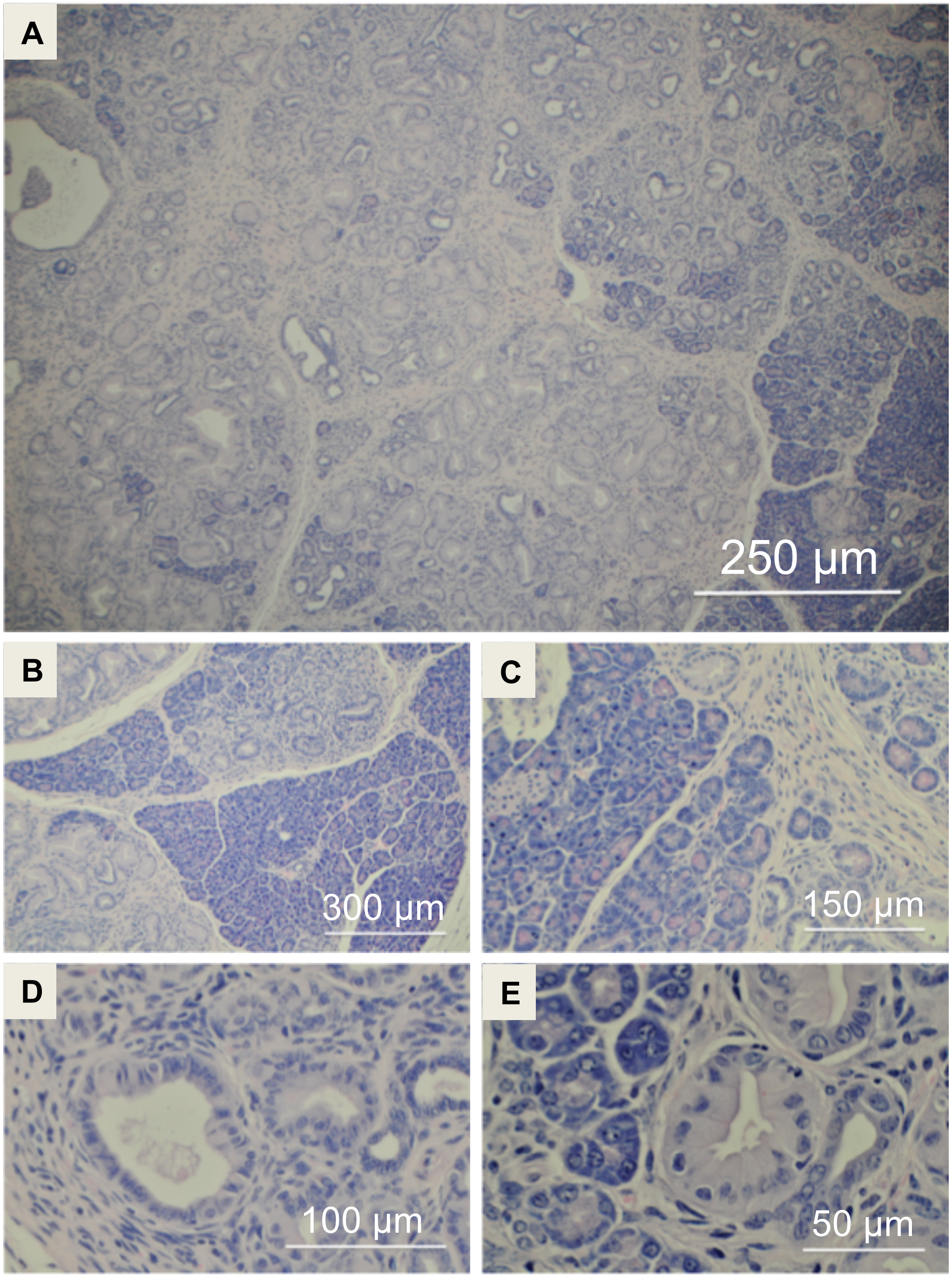

Fig D. Representative Hematoxylin and Eosin Stained Images from a 5-Month Old Female Study Mouse. (A)Magnification at 4X. (B) Magnification at 10X. (C) Magnification at 20X. (D) Magnification at 40X. (E) Magnification at 60X.

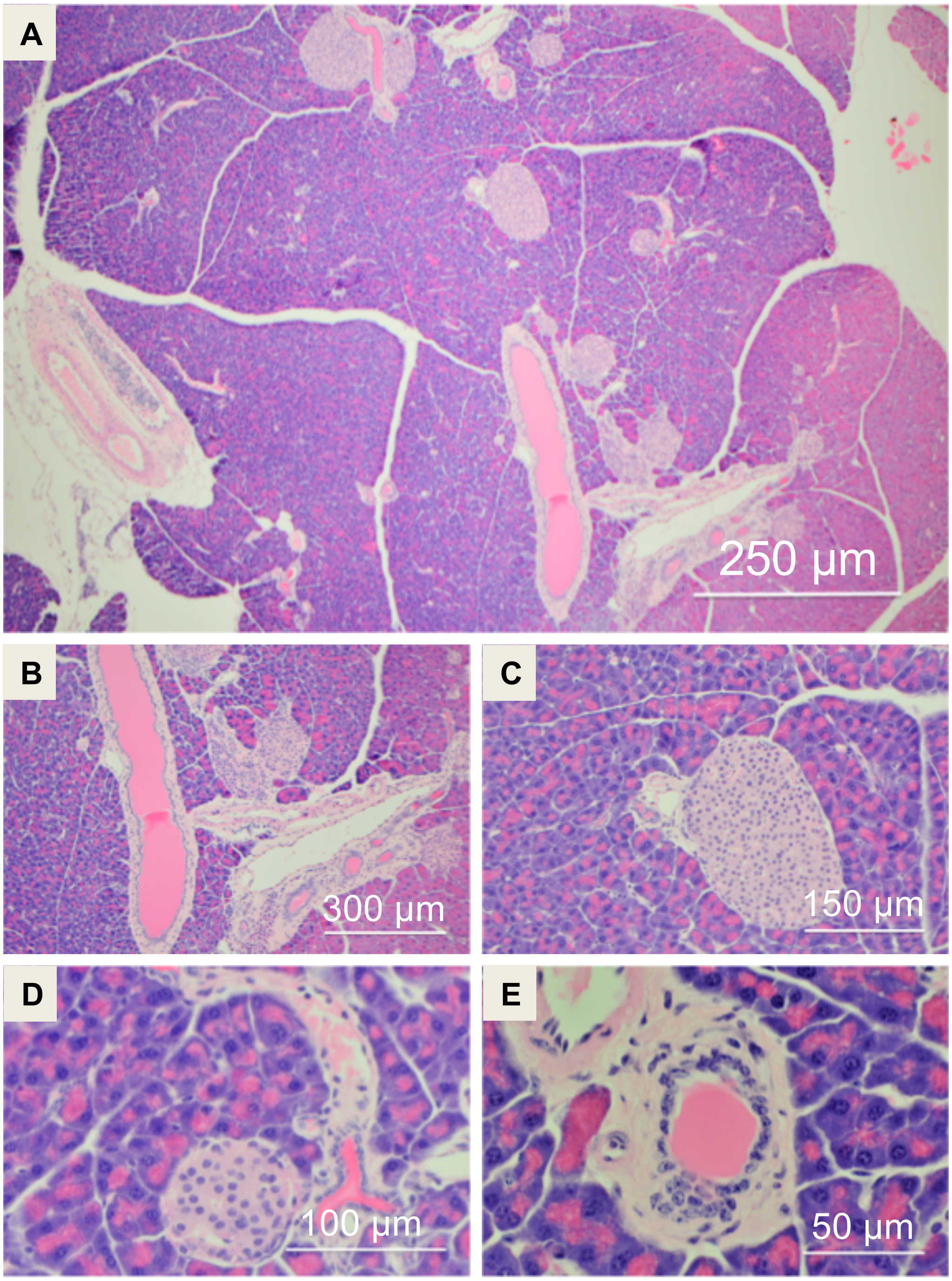

Fig E. Representative Hematoxylin and Eosin Stained Images from a 15-Month Old Control Male Mouse. (A) Magnification at 4X. (B) Magnification at 10X. (C) Magnification at 20X. (D) Magnification at 40X. (E) Magnification at 60X.

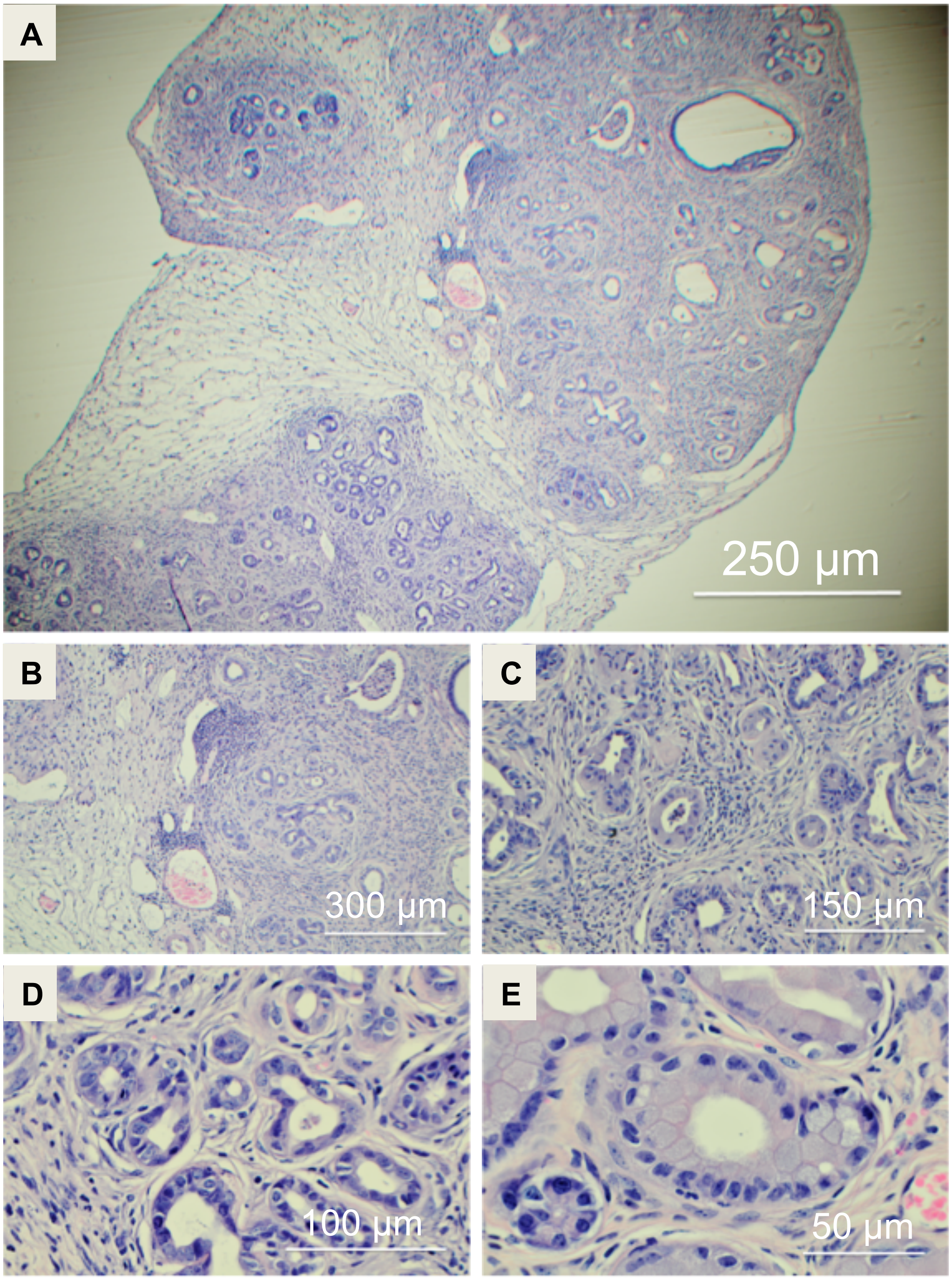

Fig F. Representative Hematoxylin and Eosin Stained Images from a 15-Month Old Male Study Mouse. (A) Magnification at 4X. (B) Magnification at 10X. (C) Magnification at 20X. (D) Magnification at 40X. (E) Magnification at 60X.

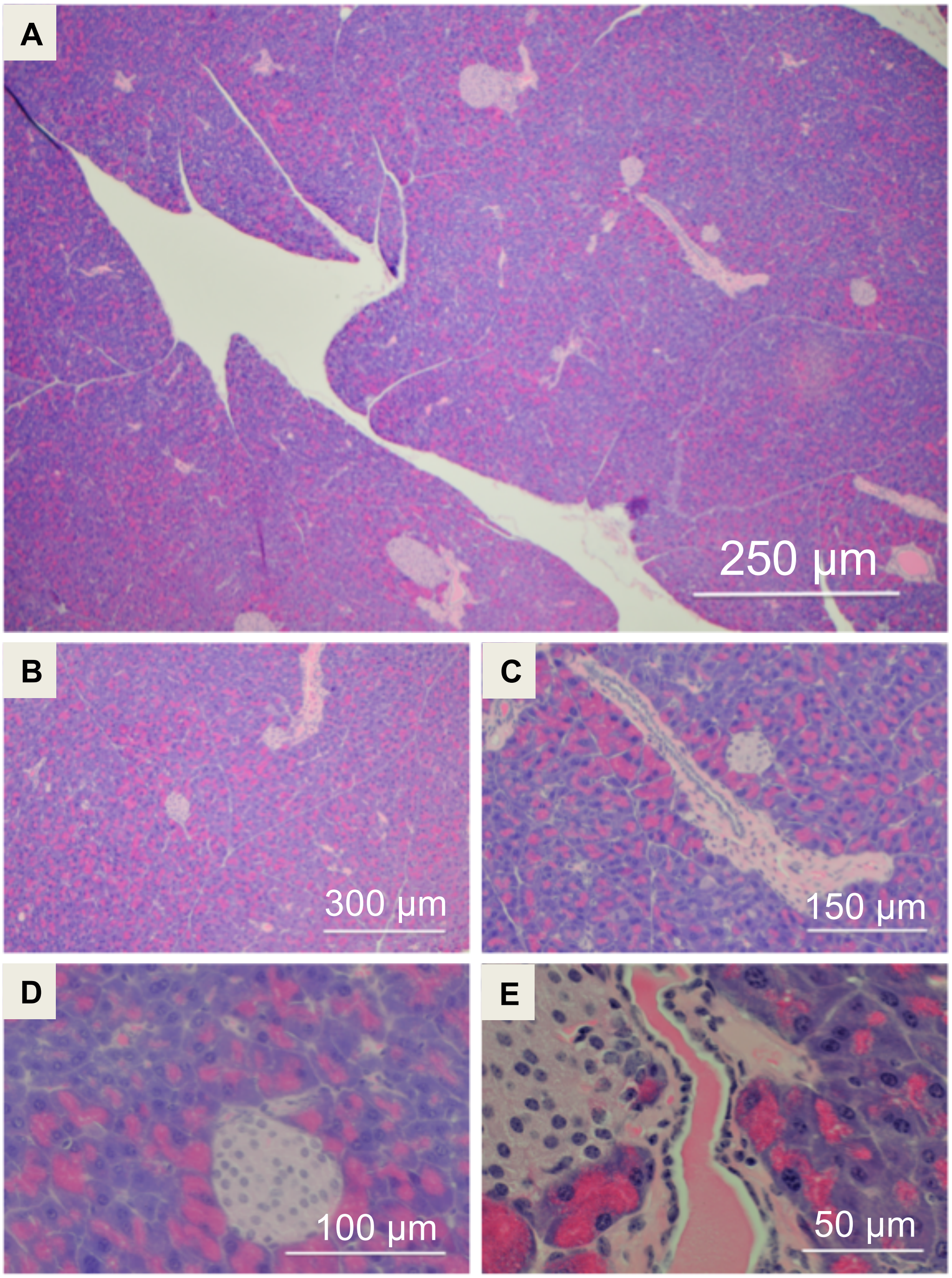

Fig G. Representative Hematoxylin and Eosin Stained Images from an 11-Month Old Male Control Mouse. (A) Magnification at 4X. (B) Magnification at 10X. (C) Magnification at 20X. (D) Magnification at 40X. (E) Magnification at 60X.

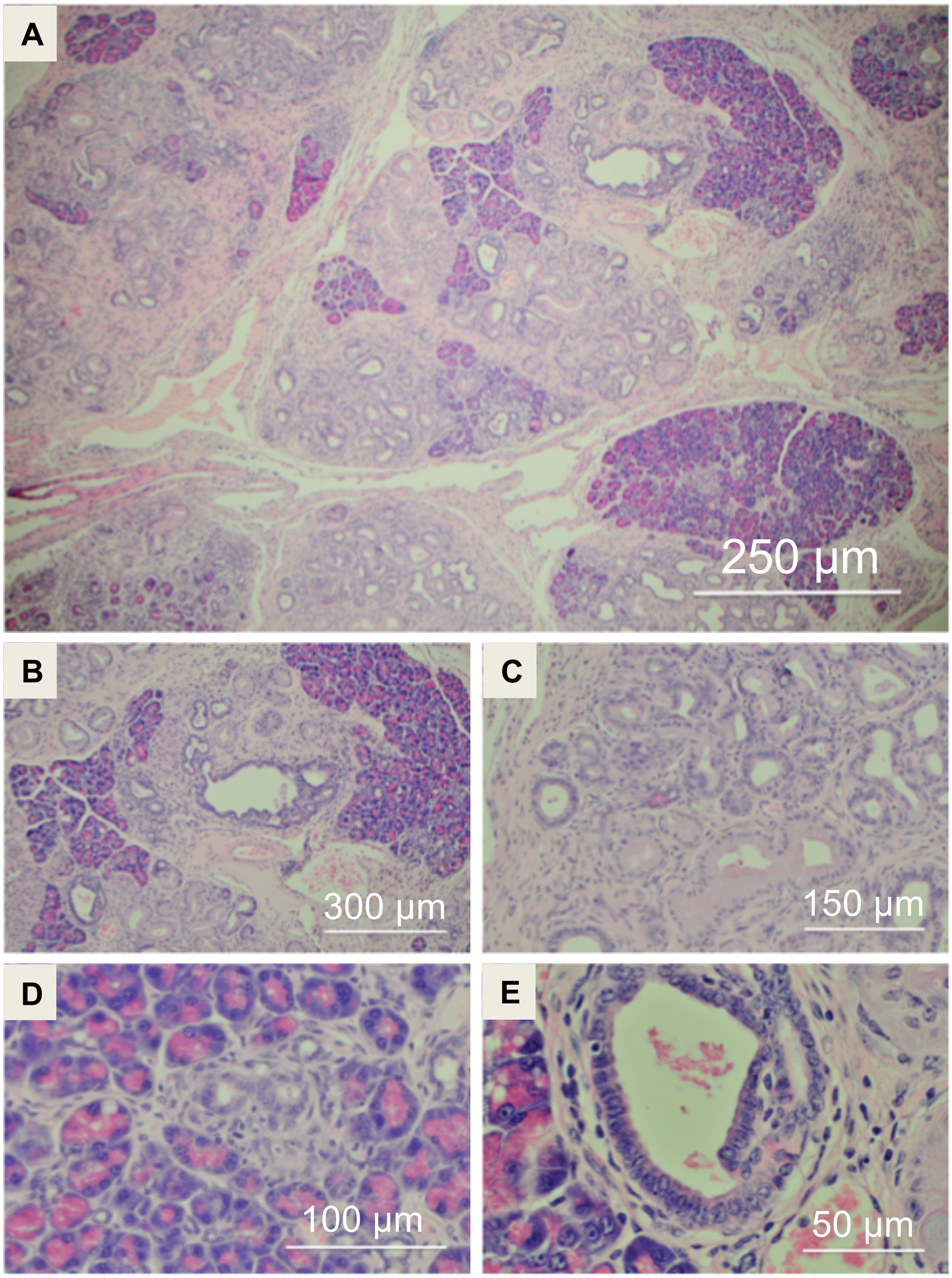

Fig H. Representative Hematoxylin and Eosin Stained Images from an 11-Month Old Male Study Mouse. (A) Magnification at 4X. (B) Magnification at 10X. (C) Magnification at 20X. (D) Magnification at 40X. (E) Magnification at 60X.

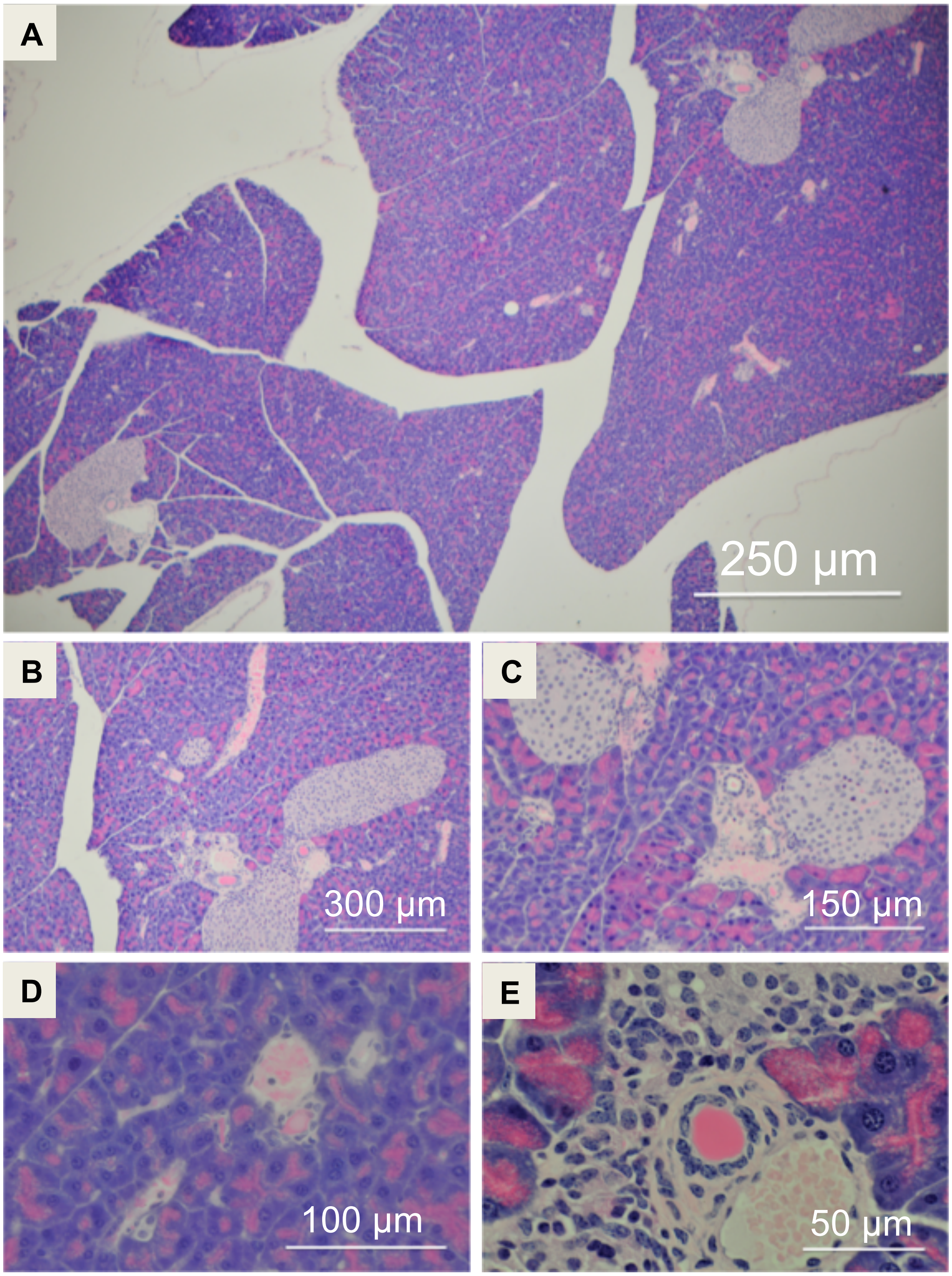

Fig I. Representative Hematoxylin and Eosin Stained Images from a 5-Month Old Male Control Mice. (A) Magnification at 4X. (B) Magnification at 10X. (C) Magnification at 20X. (D) Magnification at 40X. (E) Magnification at 60X.

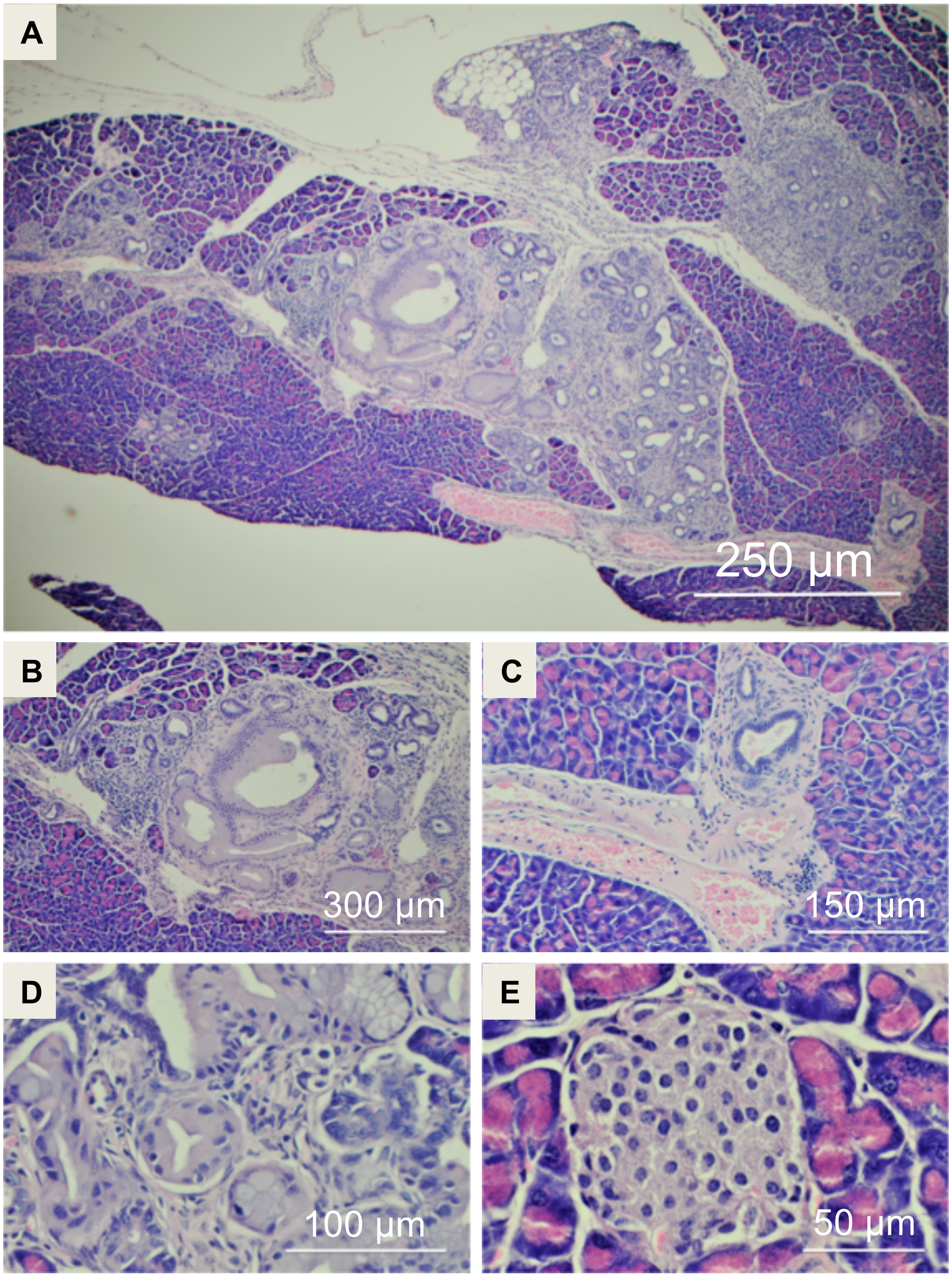

Fig J. Representative Hematoxylin and Eosin Stained Images from a 5-Month Old Male Study Mouse. (A) Magnification at 4X. (B) Magnification at 10X. (C) Magnification at 20X. (D) Magnification at 40X. (E) Magnification at 60X.

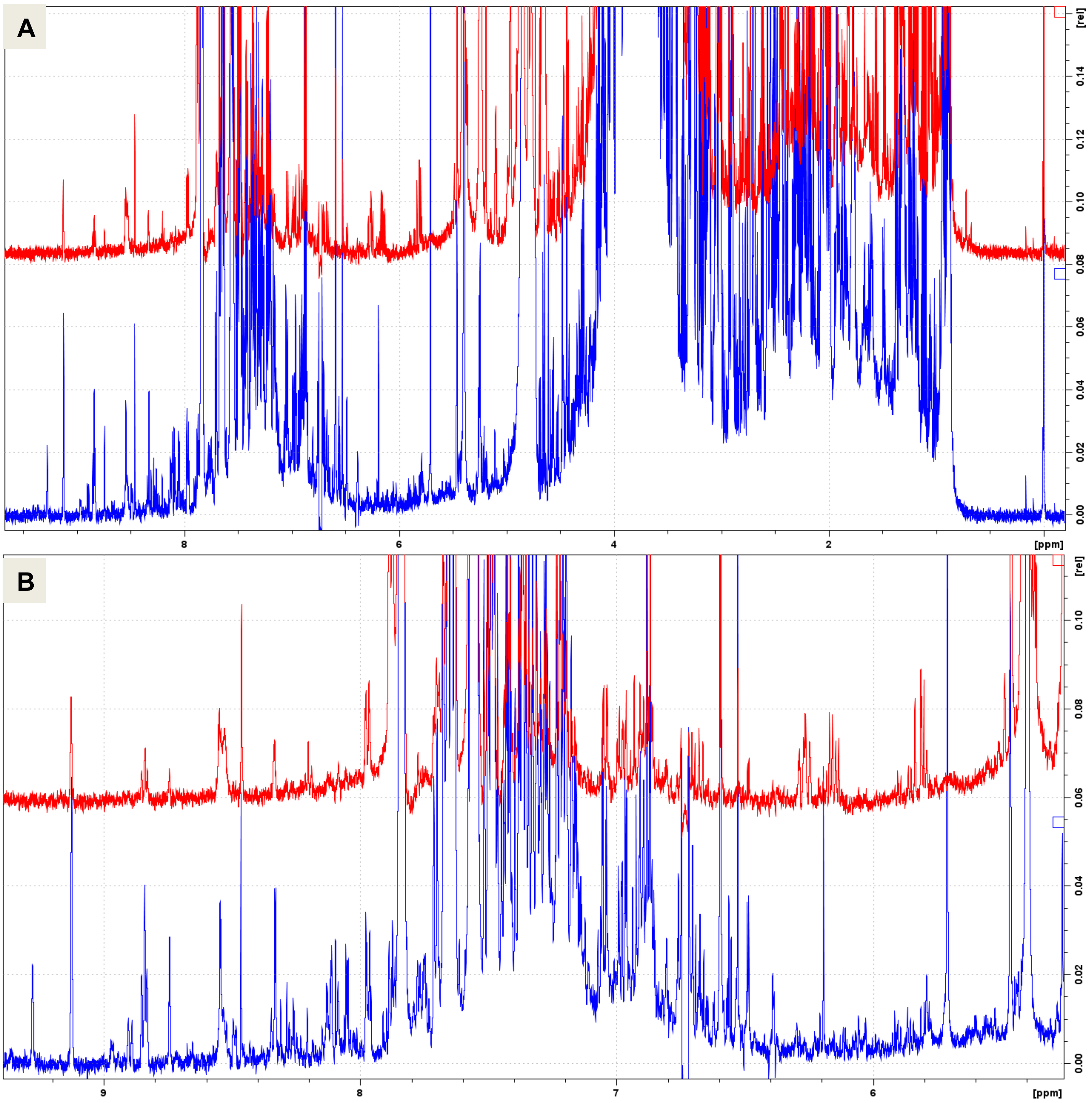

Fig K. Representative ^{1}H NMR CPMG Spectra of 15-Month Old Male Urine Samples. (A) Image displays the control spectra in blue and the study spectra in red. (B) Zoomed in image of the control (blue) and red (study) spectra so all visual differences are easier to be seen.

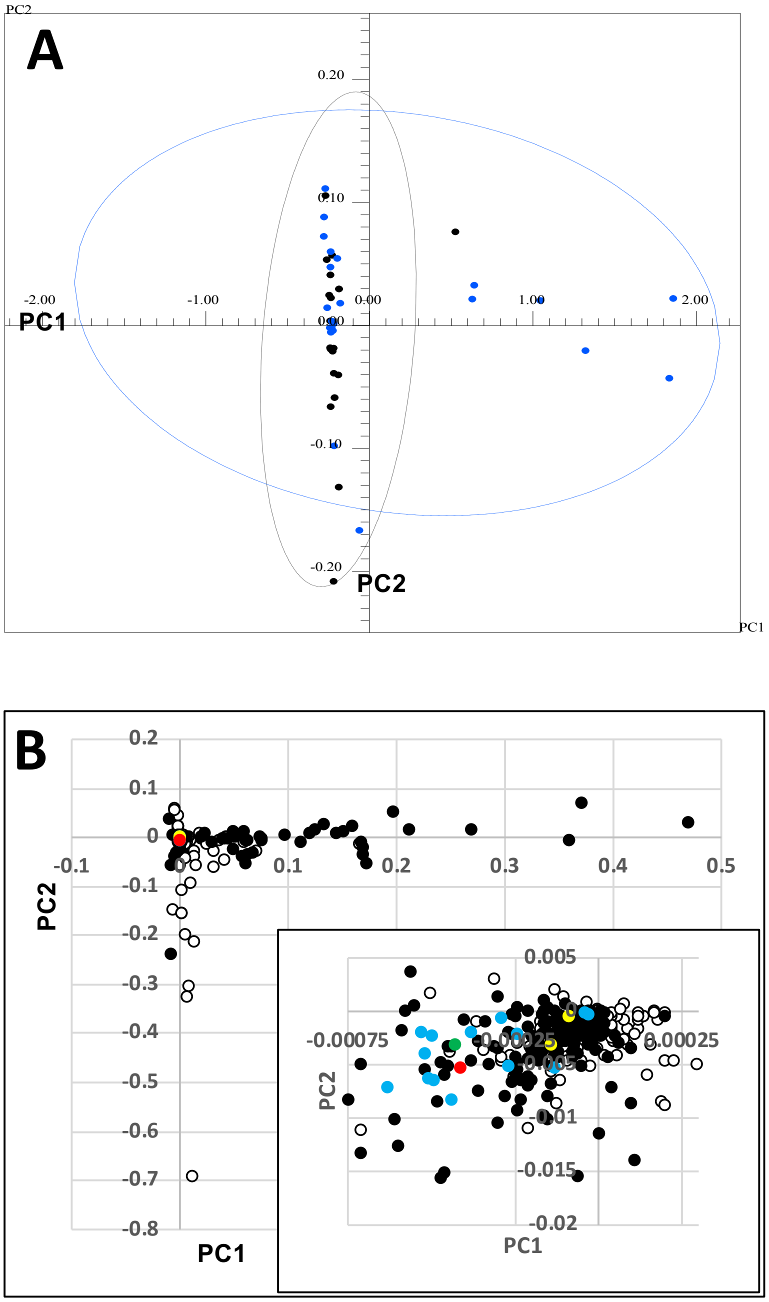

Fig L. PCA of 15-Month Old Male Urine Samples. (A) PCA scores plot for comparison between the control and study groups. (B) PCA loadings plot displaying the color-coded buckets. Red (10-9 – 10-13), yellow (10-9 – 10-7), green (10-7 – 10-5), blue (10-5 – 1.82X10-4), black closed (<0.05), black open (>0.05).

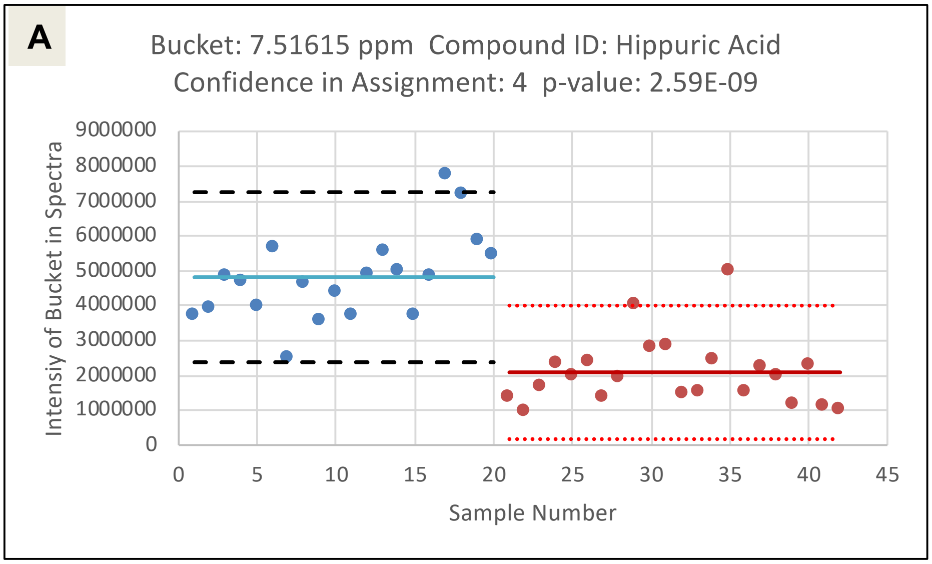

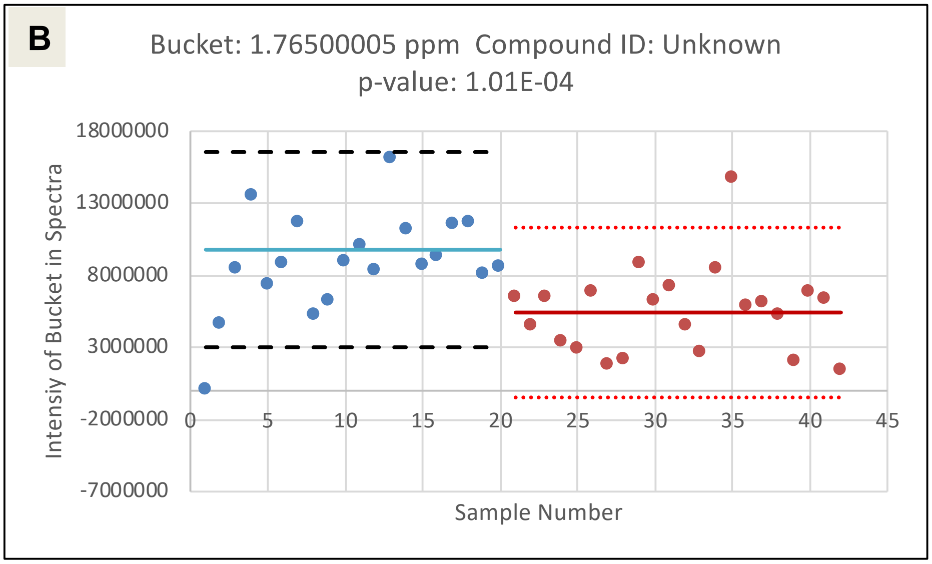

Fig M. Intensity Plots for 15-Month Old Male Urine Samples. (A) Unidentified bucket at 7.52 ppm. (B) Unidentified bucket at 1.76 ppm.

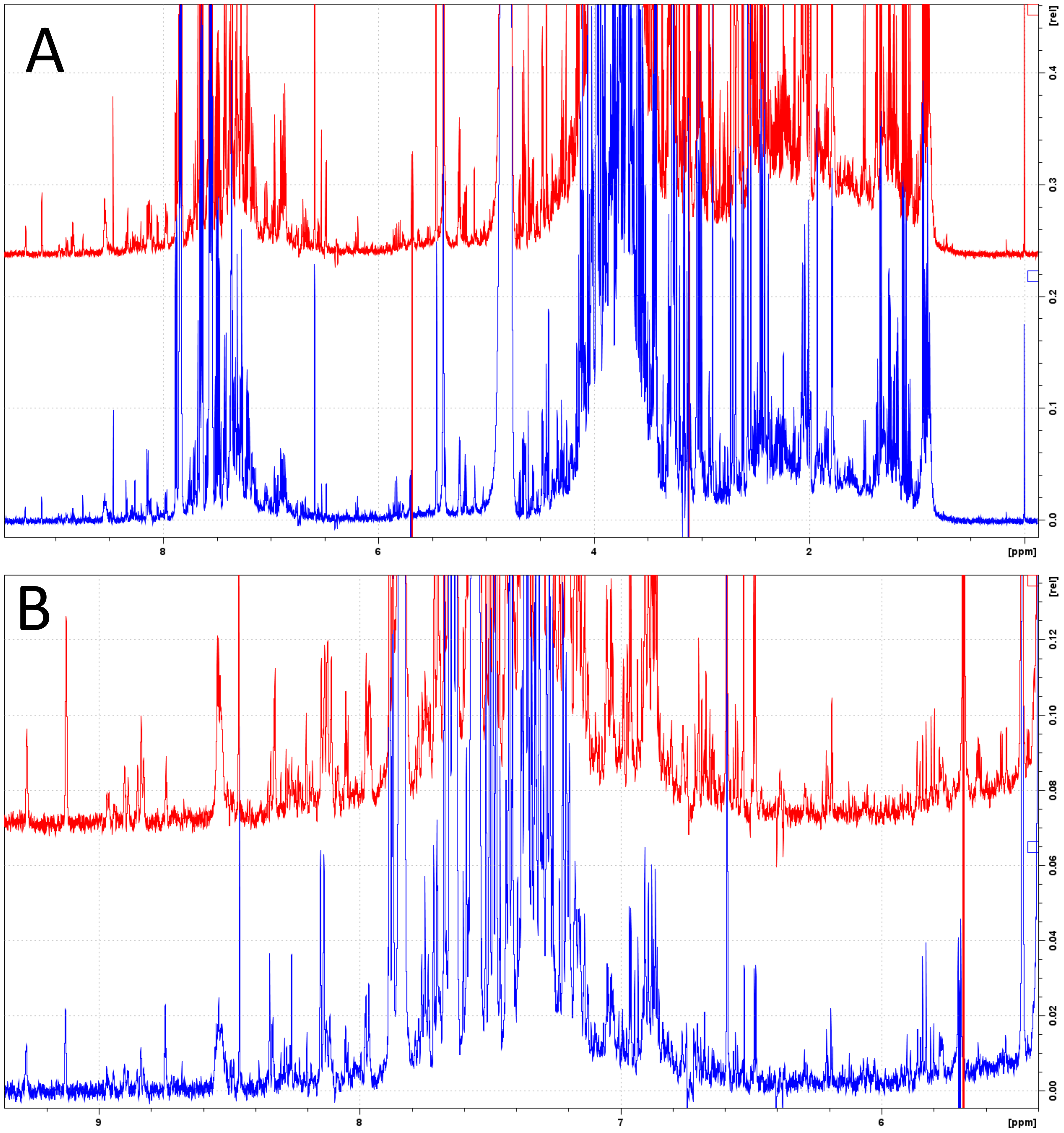

Fig N. Representative ^{1}H NMR CPMG Spectra of 15-Month Old Female Urine Samples. (A) Control spectra (blue) and study spectra (red). (B) Zoomed in image of the control (blue) and red (study) spectra to make visual differences easier to see.

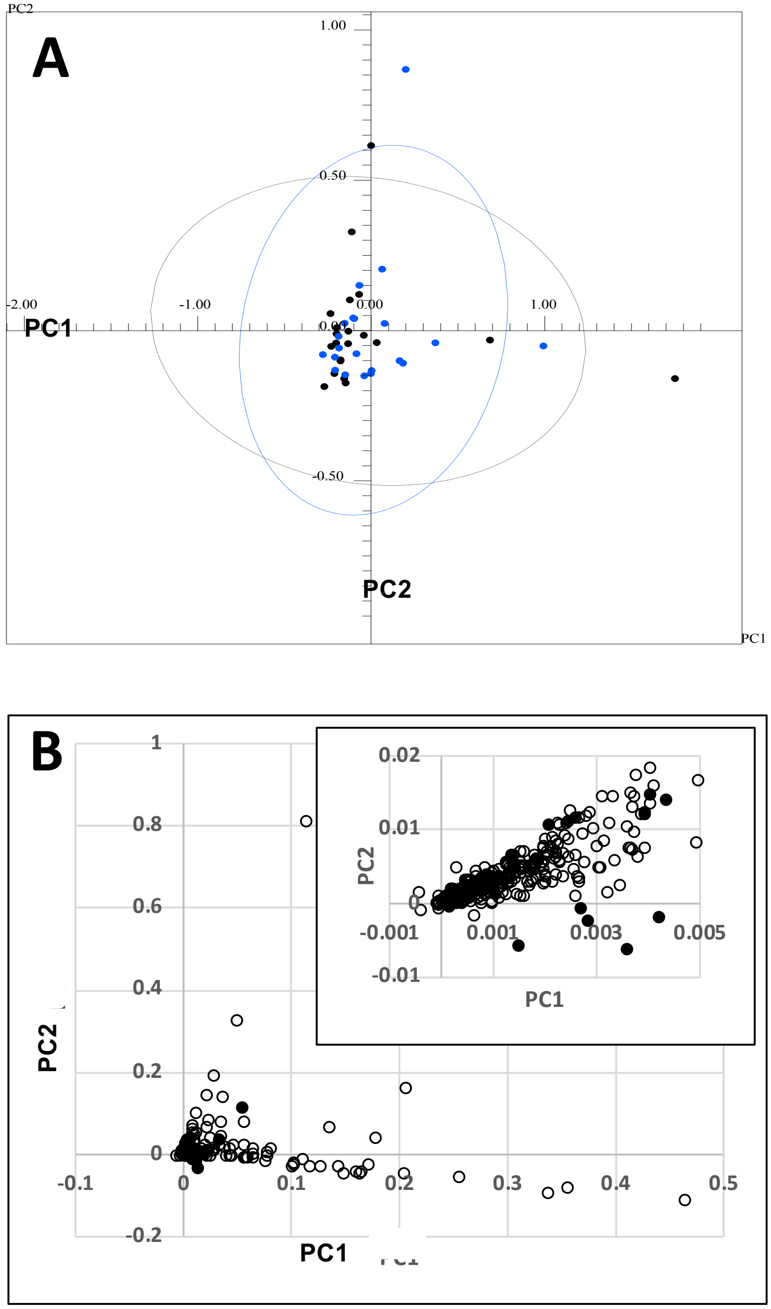

Fig O. PCA of 15-Month Old Female Urine Samples. (A)PCA scores plot for comparison between the control and study groups. (B) PCA loadings plot displaying the color-coded buckets. Red (10-9 – 10-13), yellow (10-9 – 10-7), green (10-7 – 10-5), blue (10-5 – 1.82X10-4), black closed (<0.05), black open (>0.05).

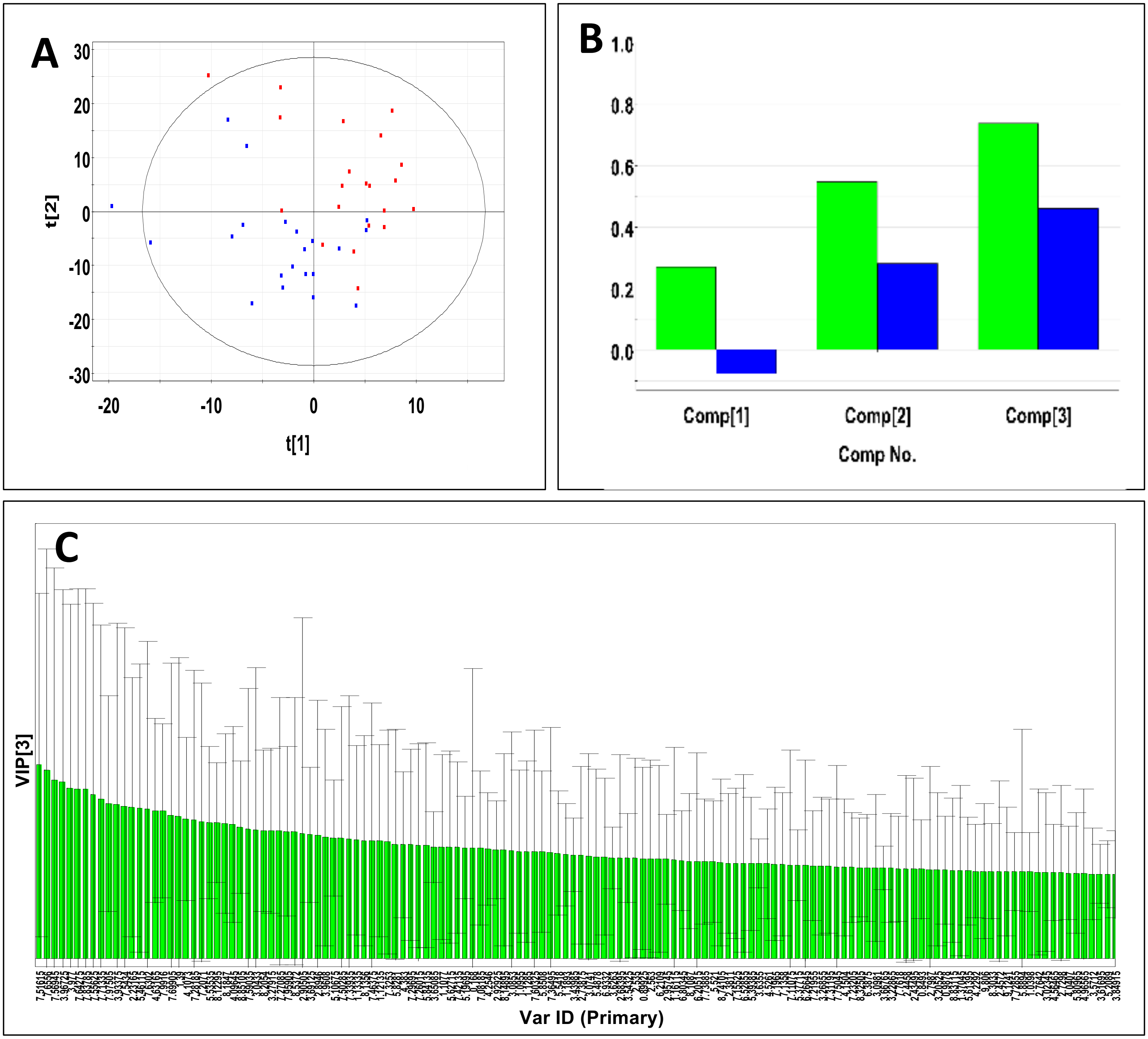

Fig P. PLS-DA of 15-Month Old Female Urine Samples. (A) PLS-DA scores plot. (B) A plot of the first three PCs displaying the R2Y explained variation and the Q2 predicted variation. (C) VIP numbers from the PLS-DA analysis that are greater than or less than 1.

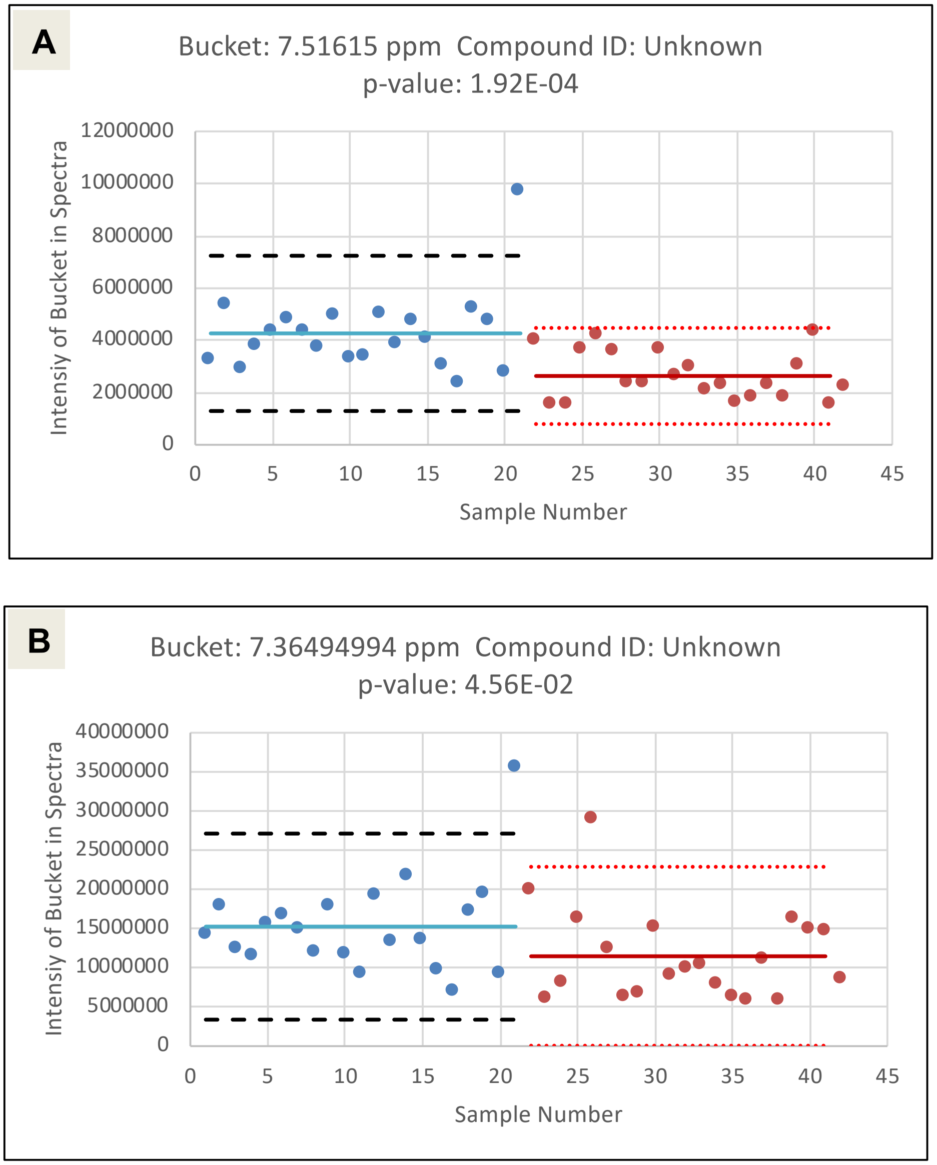

Fig Q. Intensity Plots for 15-Month Old Female Urine Samples. (A) Unidentified bucket at 7.52 ppm. (B) Unidentified bucket at 7.36 ppm.

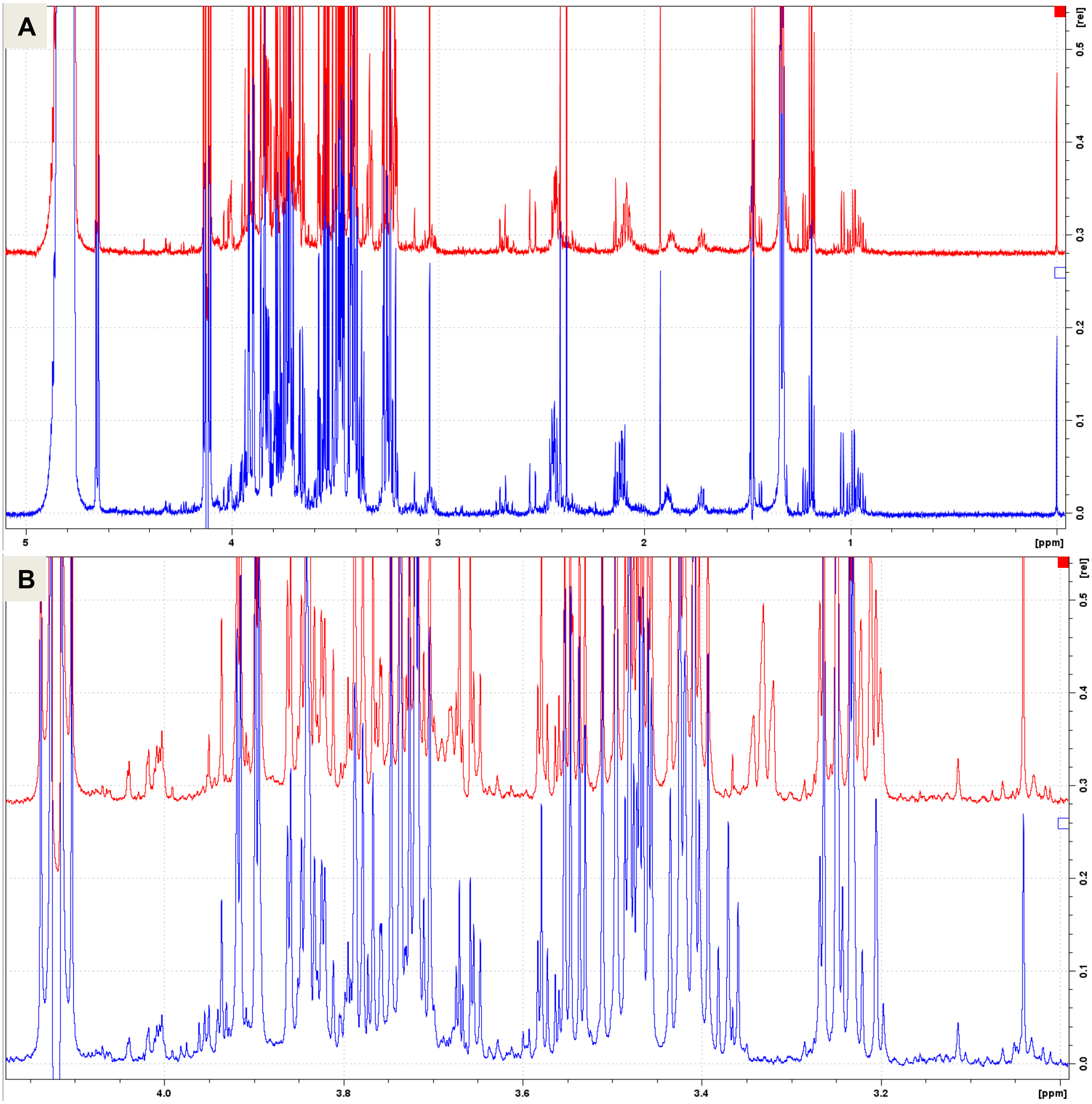

Fig R. Representative ^{1}H NMR CPMG Spectra of 15-Month Female Serum Samples. (A) Control spectra (blue) and study spectra (red). (B) Zoomed in image of the control (blue) and red (study) spectra so visual differences are easier to see.

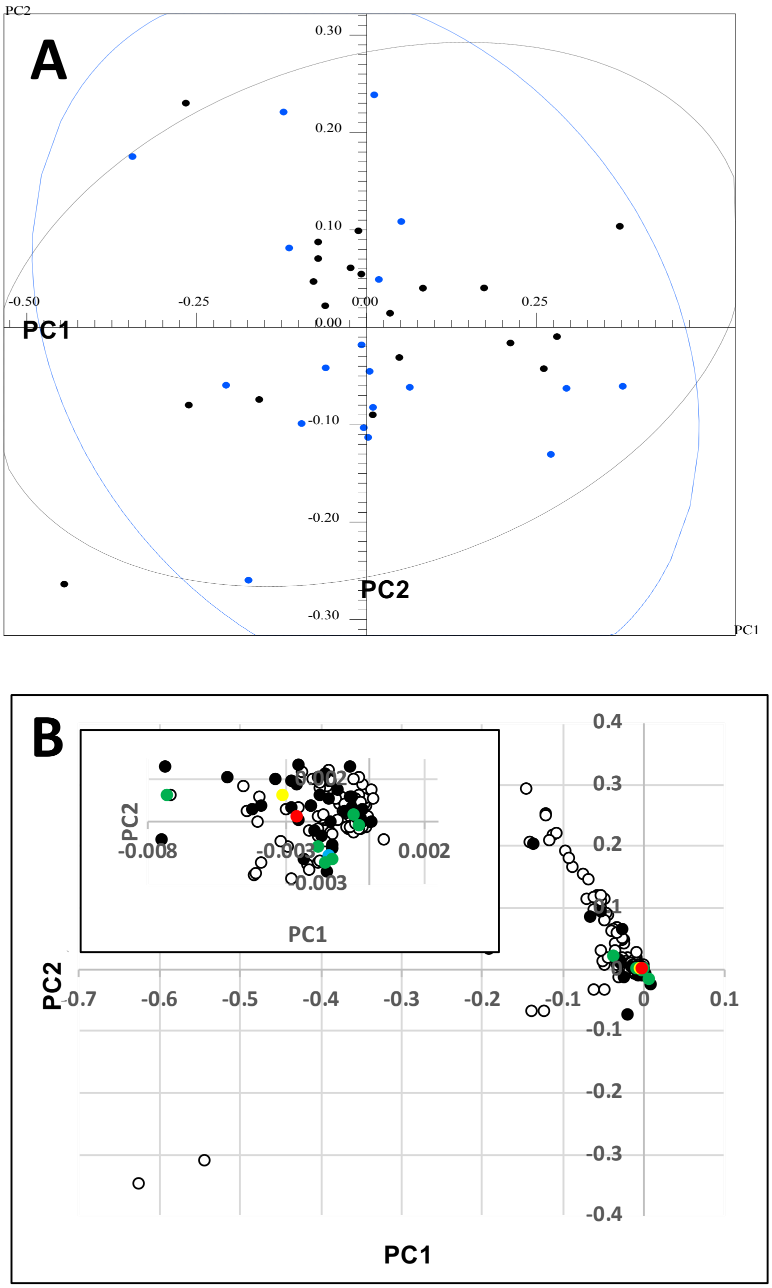

Fig S. PCA of 15-Month Old Female Serum Samples. (A) PCA scores plot for comparison between the control and study groups. (B) PCA loadings plot displaying the color-coded buckets. Red (10-9 – 10-13), yellow (10-9 – 10-7), green (10-7 – 10-5), blue (10-5 – 1.82X10-4), black closed (<0.05), black open (>0.05).

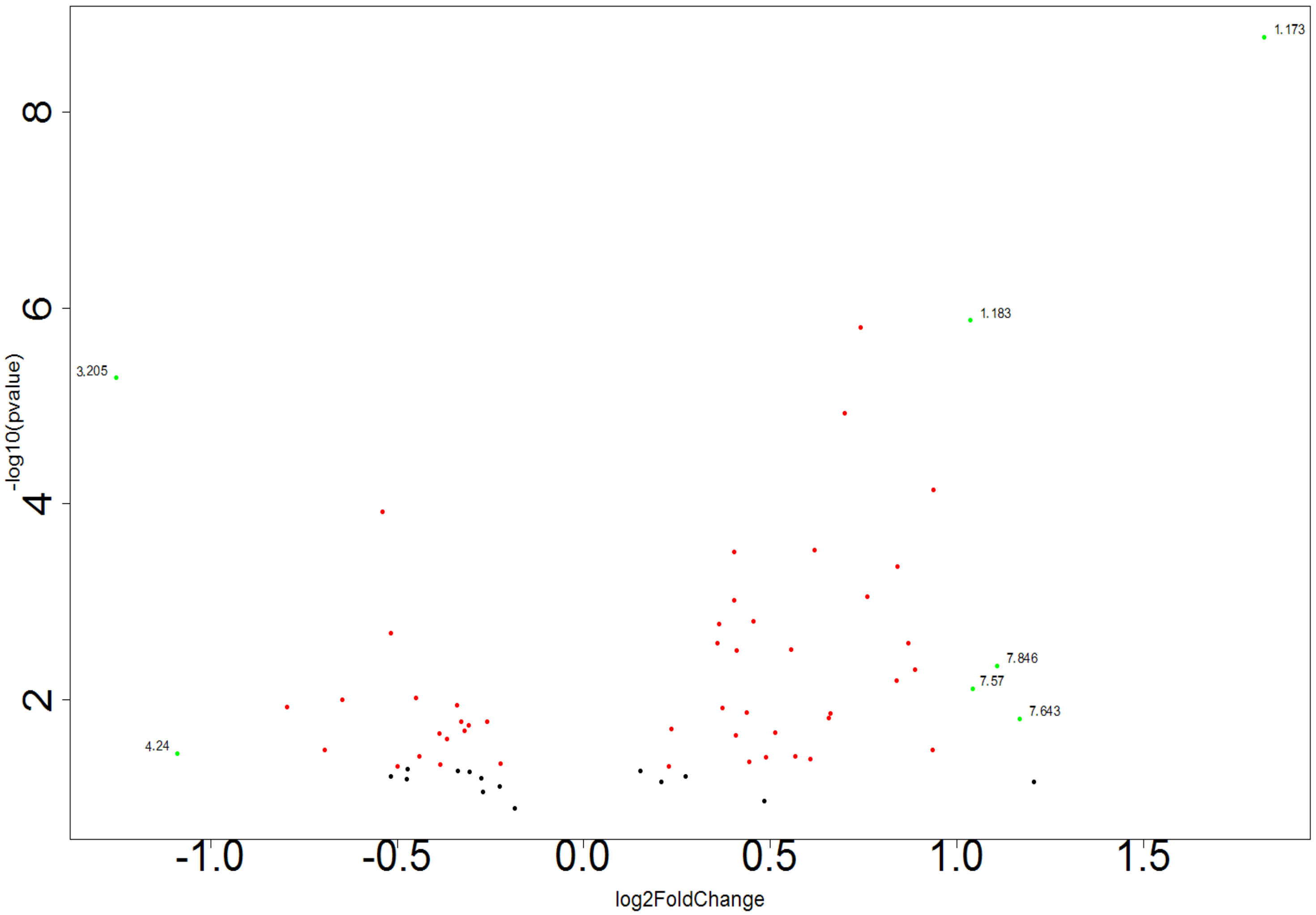

Fig T. Volcano Plot of Unidentified Peaks for 15-Month Old Female Serum Samples. The data are plotted with -log(p) along the y axis and log2(fold change) along the x-axis. Points colored red have p-values < 0.05. Points colored green have p-values < 0.05 and > 2 fold-change.

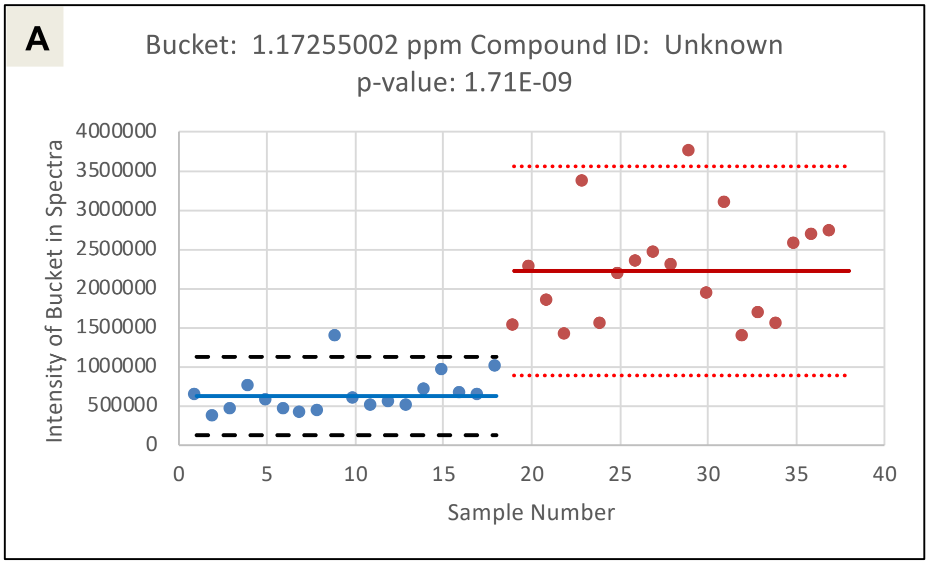

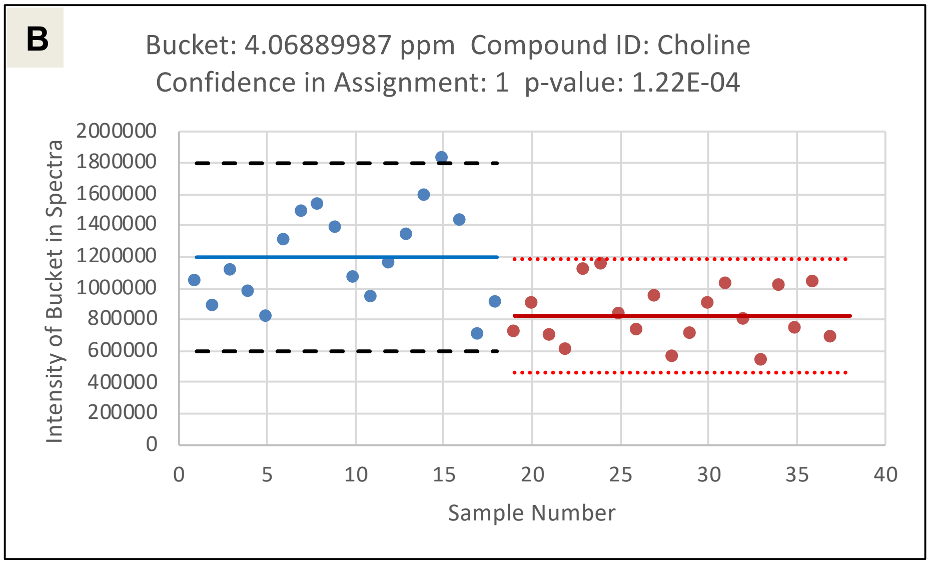

Fig U. Intensity Plots for 15-Month Old Female Serum Samples.
(A) Unidentified bucket at 1.17 ppm. (B) Choline bucket at 4.069 ppm.

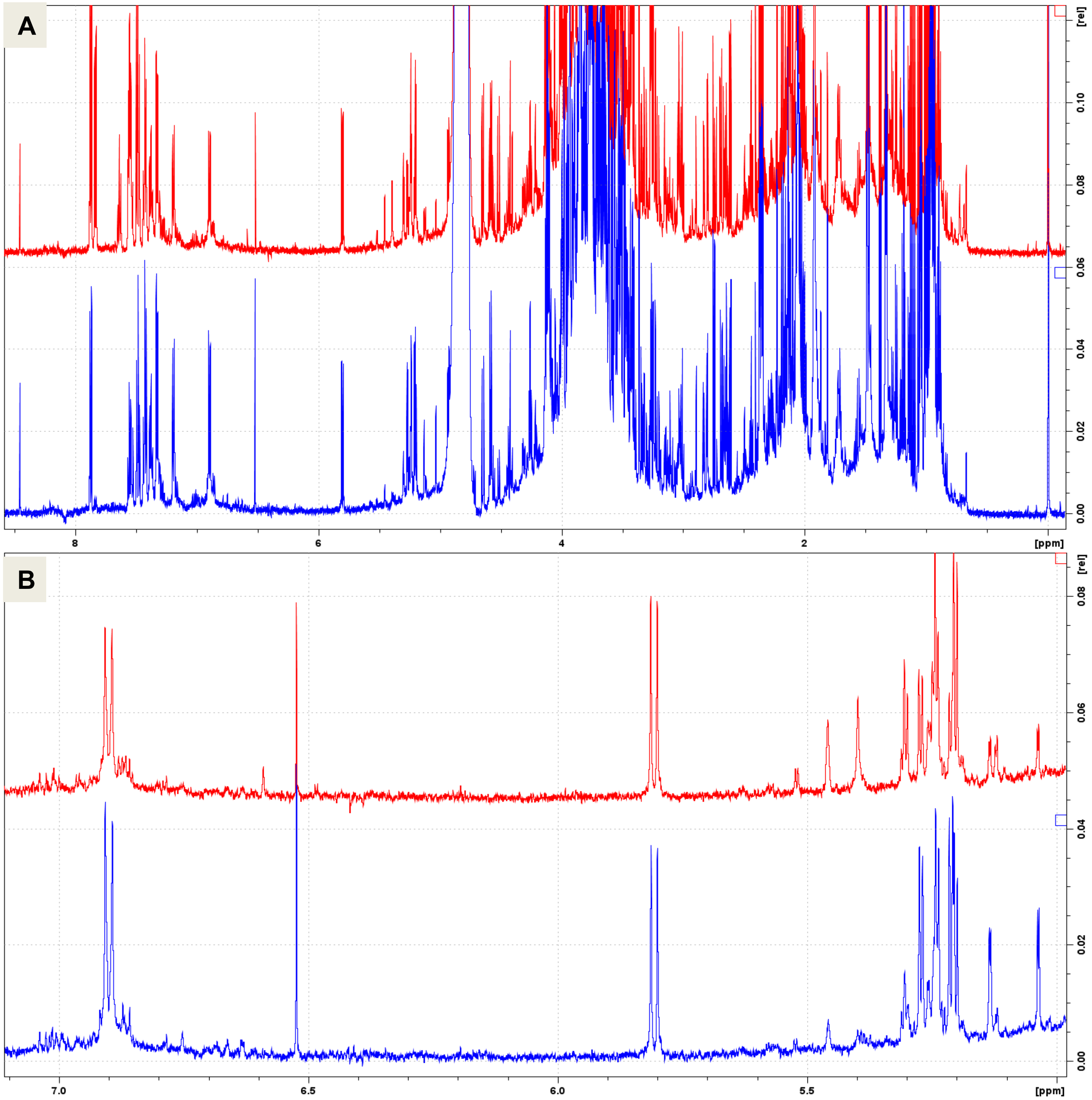

Fig V. Representative ^{1}H NMR CPMG Spectra of 15-Month Old Female Fecal Extract Samples. (A) Image displays the control spectra in blue and the study spectra in red. (B) Zoomed in image of the control (blue) and red (study) spectra so all visual differences are easier to be seen.

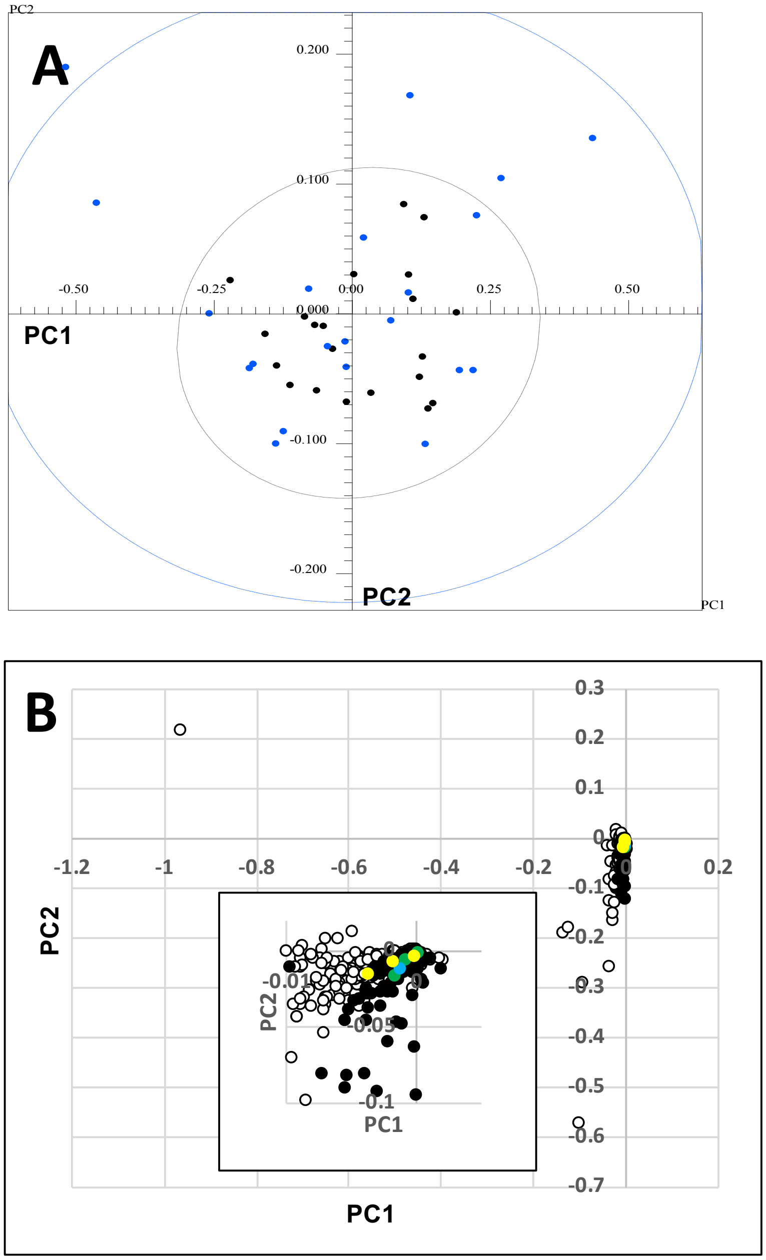

Fig W. PCA of 15-Month Old Female Fecal Extract Samples. (A) PCA scores plot for comparison between the control and study groups. (B) PCA loadings plot displaying the color-coded buckets. Red (10-9 – 10-13), yellow (10-9 – 10-7), green (10-7 – 10-5), blue (10-5 – 1.82X10-4), black closed (<0.05), black open (>0.05).

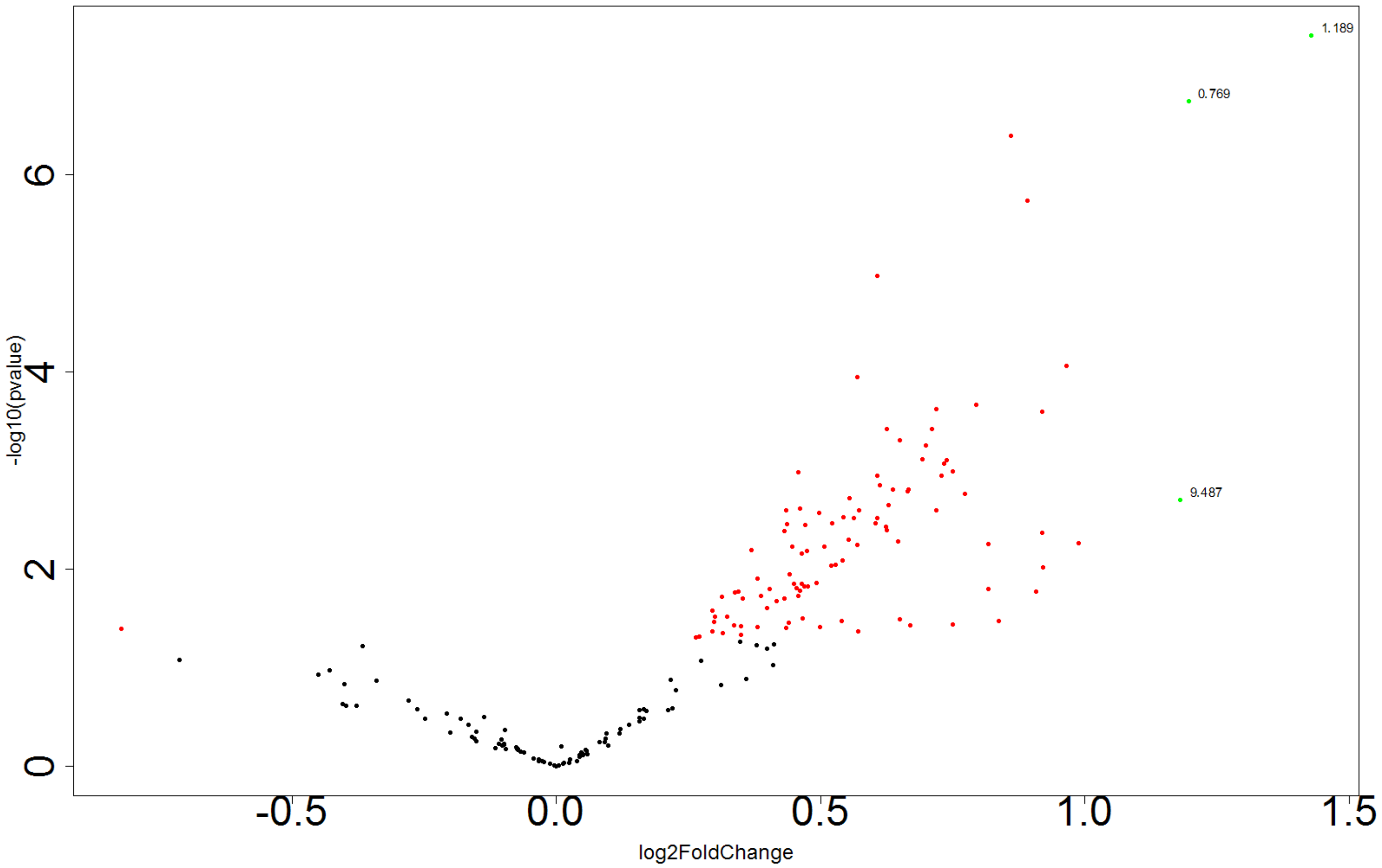

Fig X. Volcano Plot of Unidentified Peaks for 15-Month Old Female Fecal Extract Samples. Plot of -log(p) versus log2(fold change). Points colored red have p-values < 0.05. Points colored green have p-values < 0.05 and fold-change > 2.

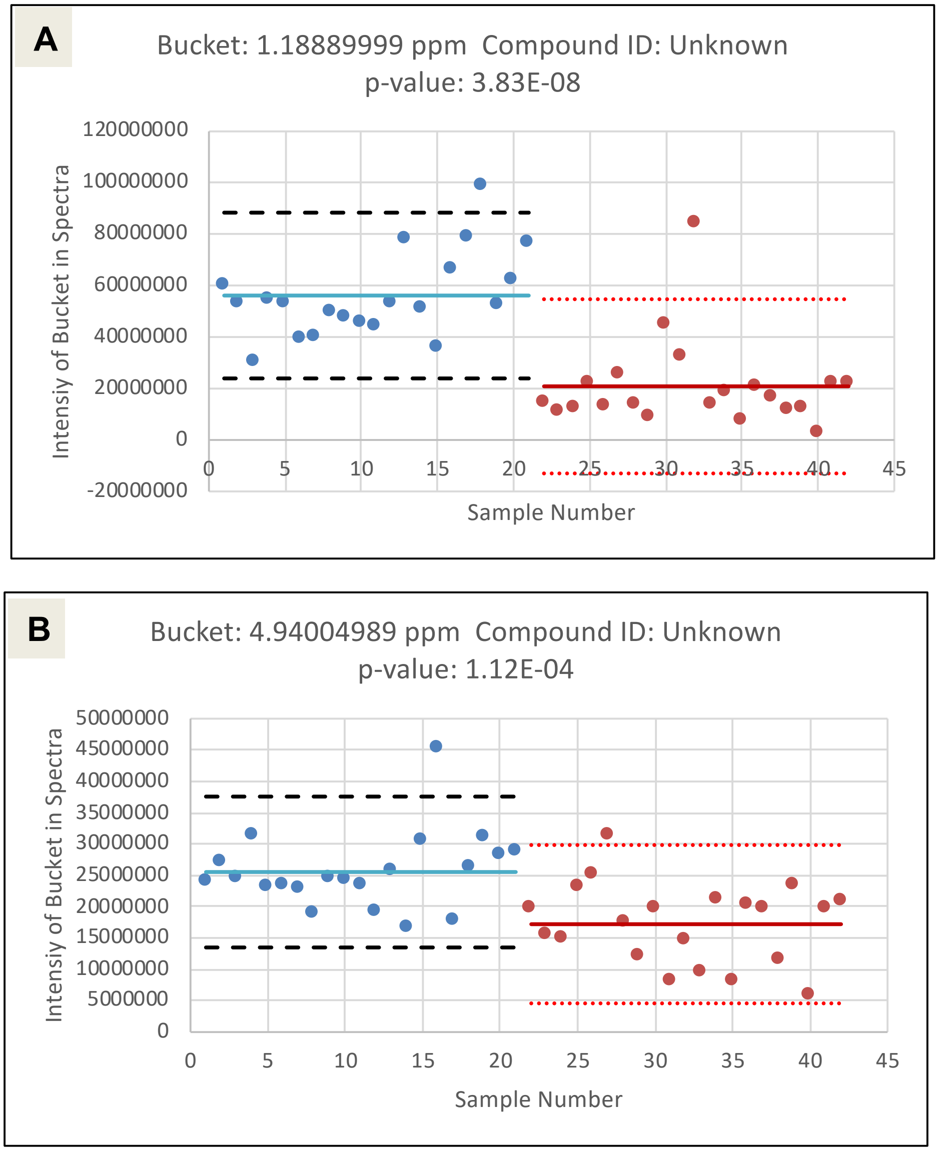

Fig Y. Intensity Plots for 15-Month Female Fecal Extract Samples. (A) Unidentified bucket at 1.19 ppm. (B) unknown bucket at 4.94 ppm.

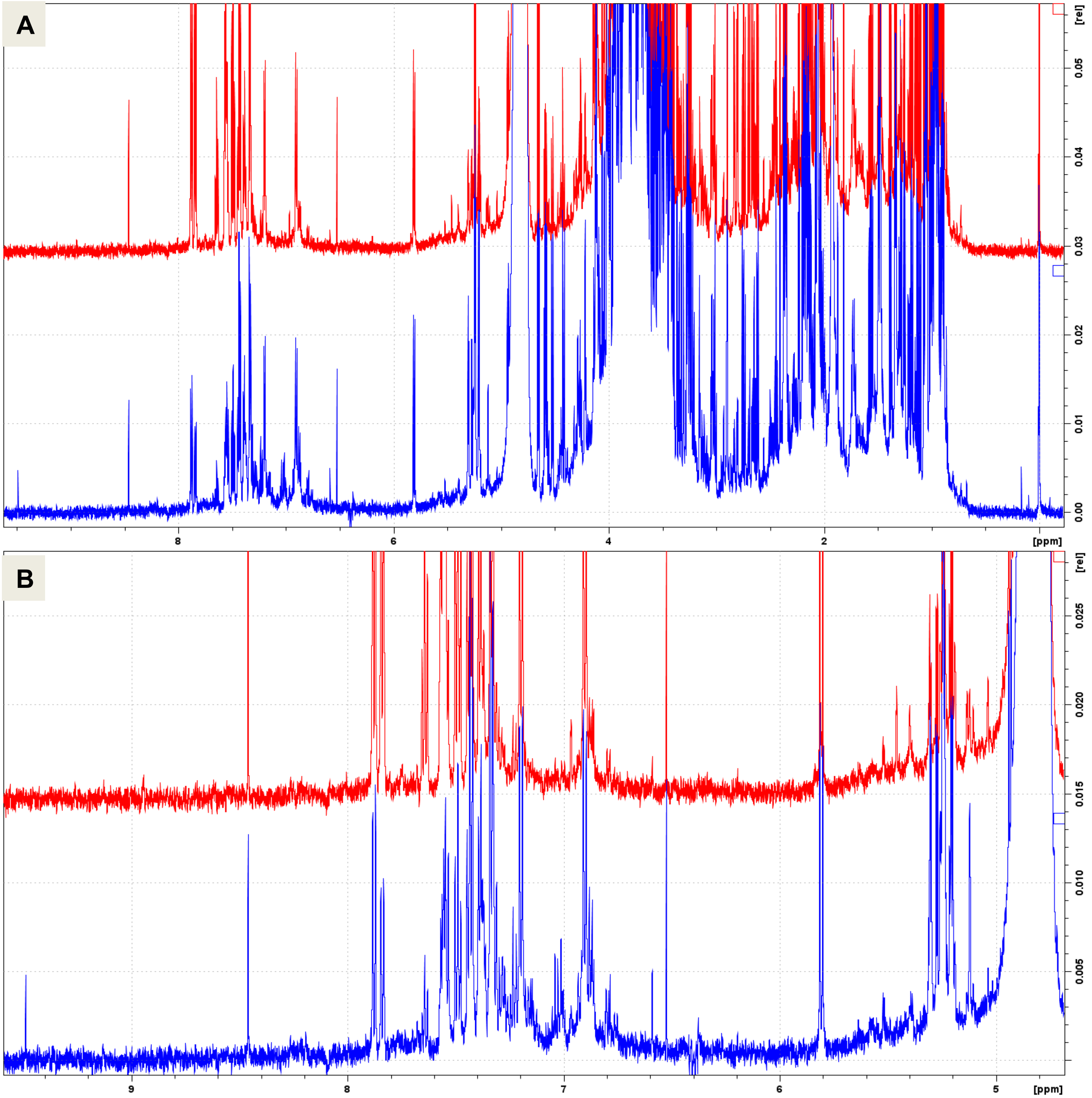

Fig Z. Representative ^{1}H NMR CPMG Spectra of 15-month Male Fecal Extract Samples. (A) Control spectra in blue and study spectra in red. (B) Zoomed in image of the control (blue) and red (study) spectra so visual differences are easier to see.

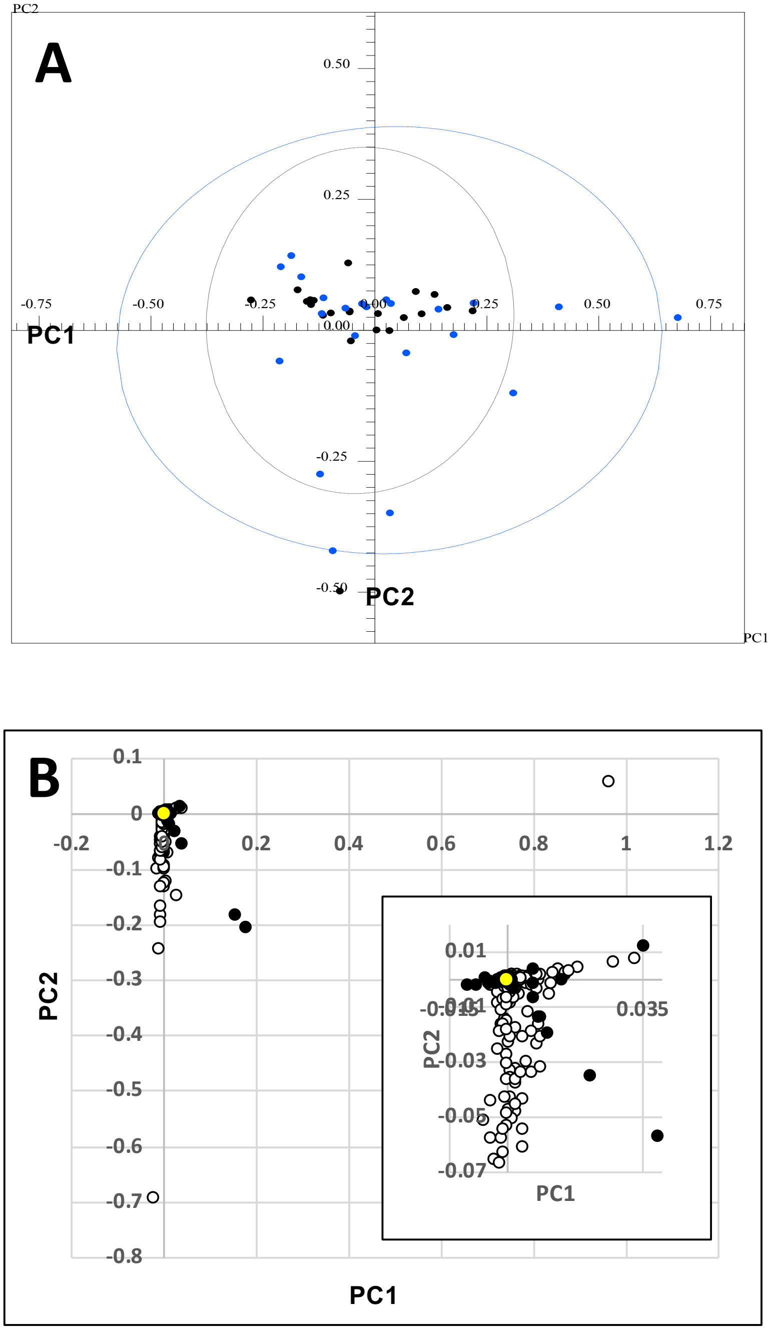

Fig AA. PCA of 15-Month Old Male Fecal Extract Samples. (A) PCA scores plot for comparison between the control and study groups. (B) PCA loadings plot displaying the color-coded buckets. Red (10-9 – 10-13), yellow (10-9 – 10-7), green (10-7 – 10-5), blue (10-5 – 1.82X10-4), black closed (<0.05), black open (>0.05).

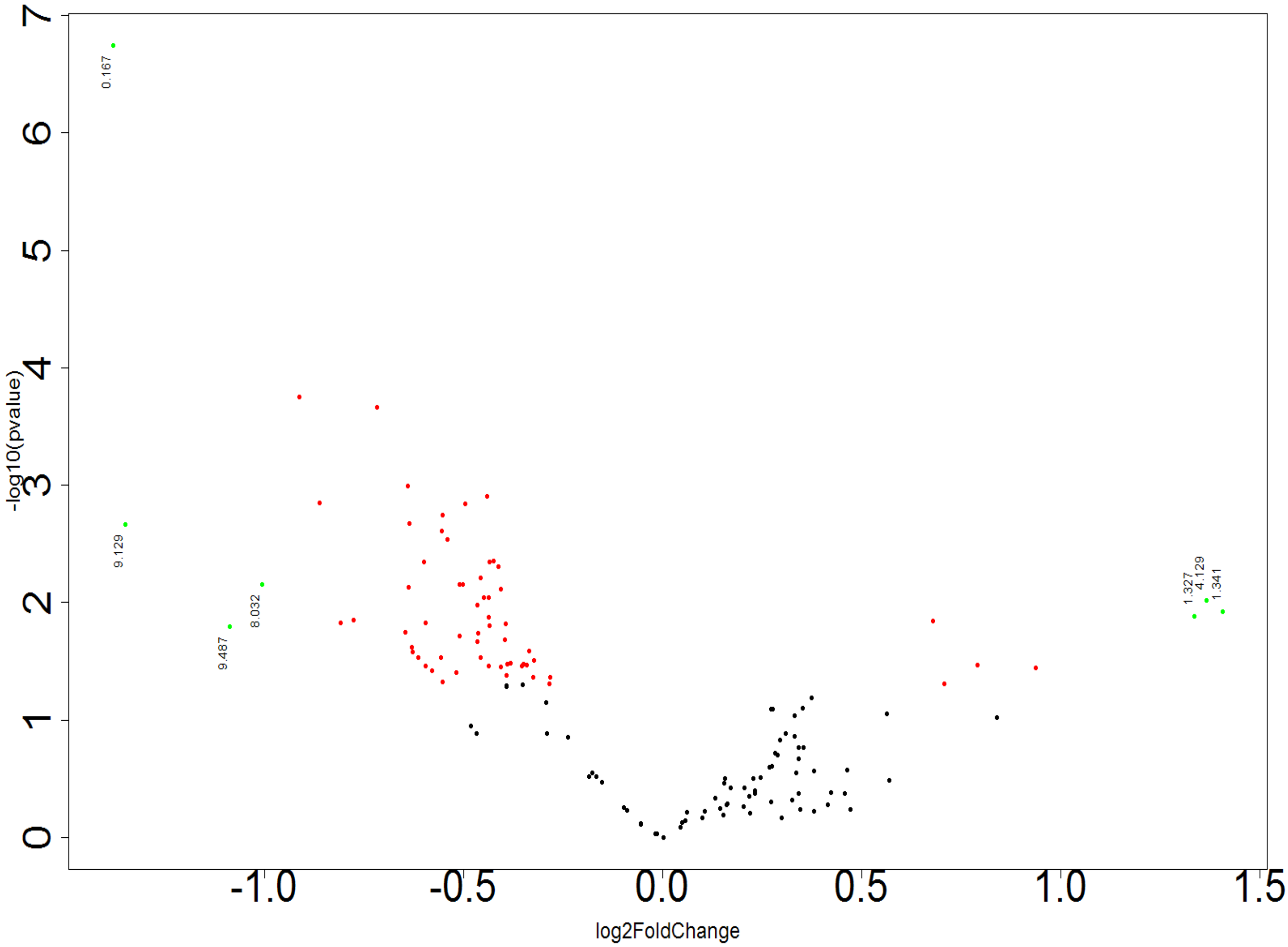

Fig AB. Volcano Plot of Unidentified Peaks for 15-Month Male Fecal Extract Samples. Plot of -log(p) versus log2(fold change). Points colored red have p-values < 0.05. Points colored green have p-values < 0.05 and fold-change > 2.

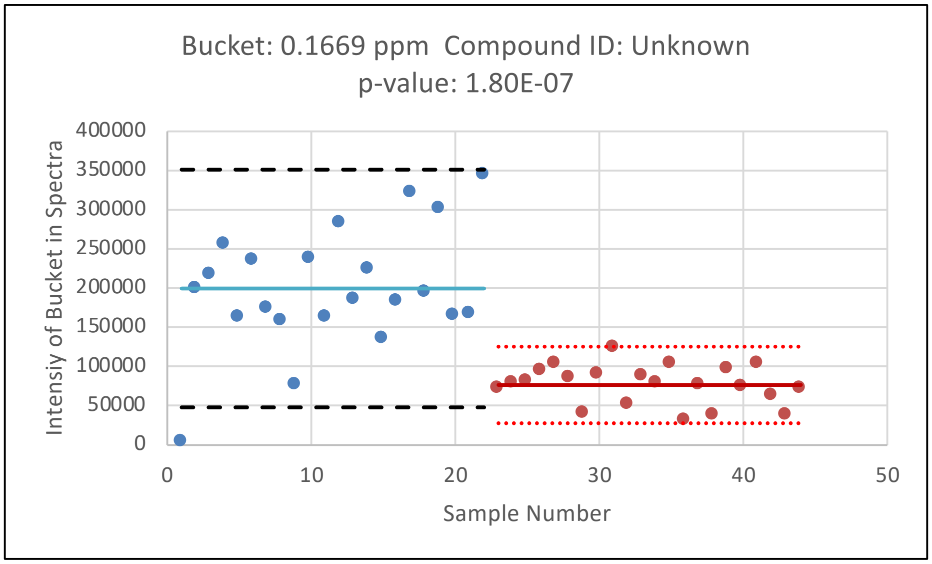

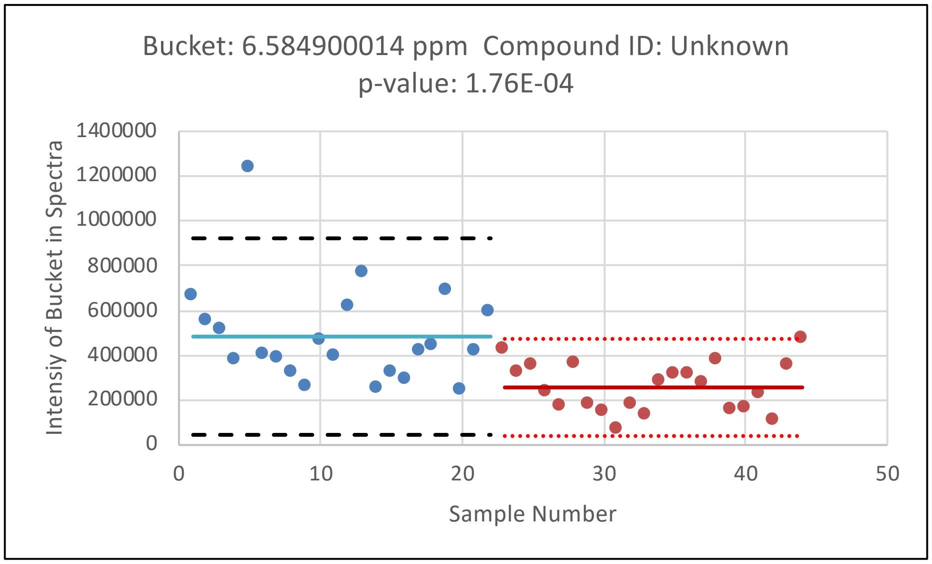

Fig AC. Intensity Plots for 15-Month Old Male Fecal Extract Samples. (A) Unidentified bucket at 0.167 ppm. (B) unknown bucket at 6.58 ppm.

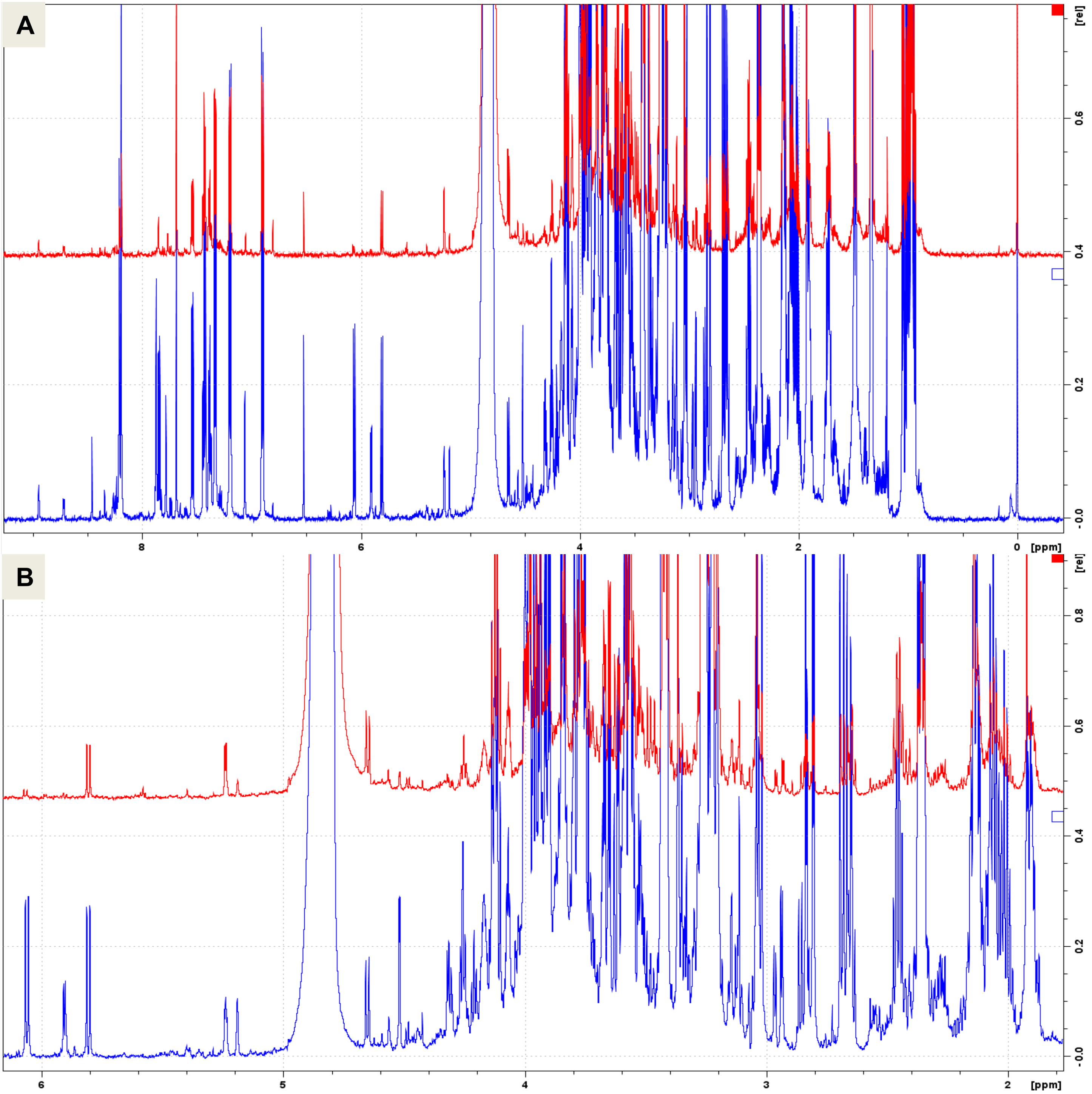

Fig AD. Representative ^{1}H NMR CPMG Spectra of 15-Month Pancreas Tissue Extract Samples. (A) Image displays the control spectra in blue and the study spectra in red. (B) Zoomed in image of the control (blue) and red (study) spectra so that visual differences are easier to see.

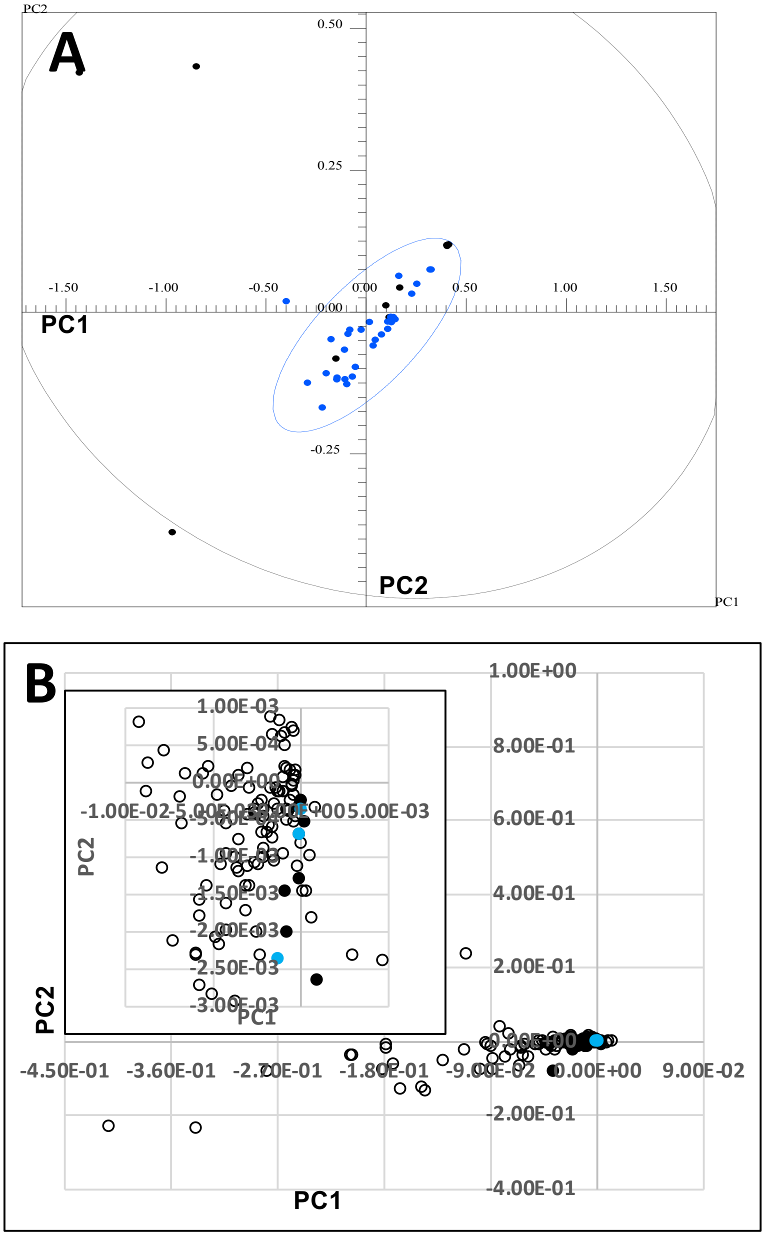

Fig AE. PCA of Pancreas Tissue Extract Samples from 15-Month Old Mice. (A) PCA scores plot for comparison between the control and study groups. (B) PCA loadings plot displaying the color-coded buckets. Red (10-9 – 10-13), yellow (10-9 – 10-7), green (10-7 – 10-5), blue (10-5 – 1.82X10-4), black closed (<0.05), black open (>0.05).

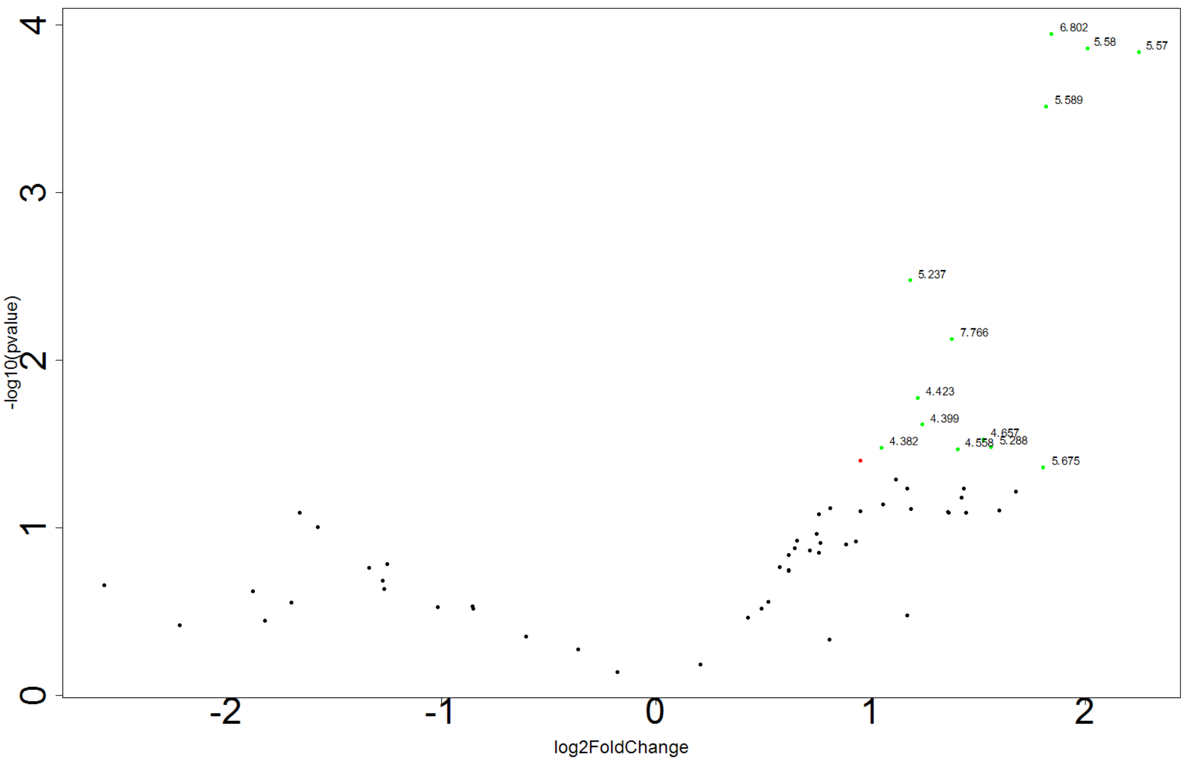

Fig AF. Volcano Plot of Unidentified Peaks for Pancreas Tissue Extract Samples from 15-Month Old Mice. Plot of -log(p) versus log2(fold change). Points colored red have p-values < 0.05. Points colored green have p-values < 0.05 and fold-change > 2.

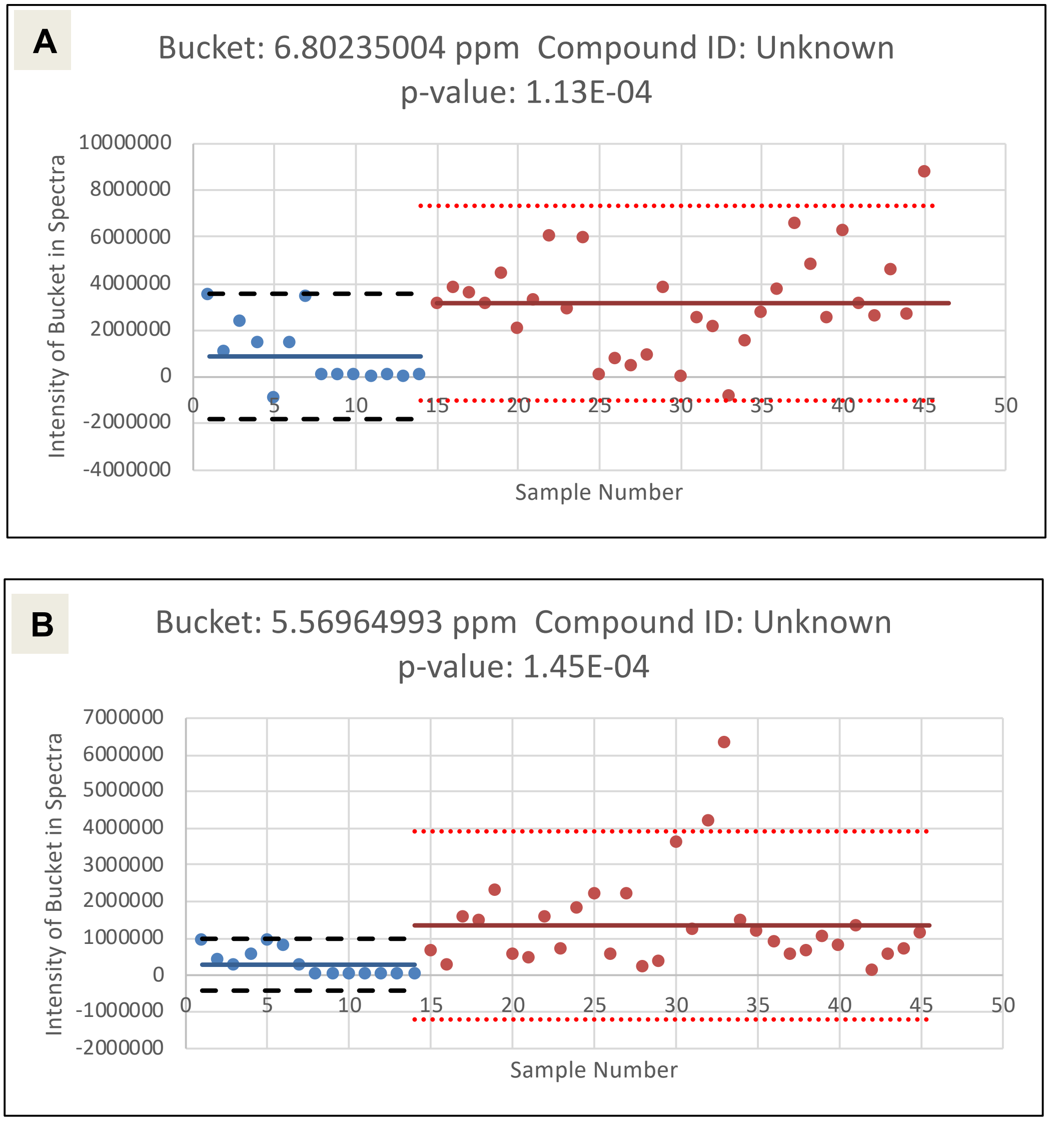

Fig AG. Intensity Plots for Pancreas Tissue Extract Samples from 15-Month Old Mice. (A) Unidentified bucket at 6.80 ppm. (B) unknown bucket at 5.57 ppm.

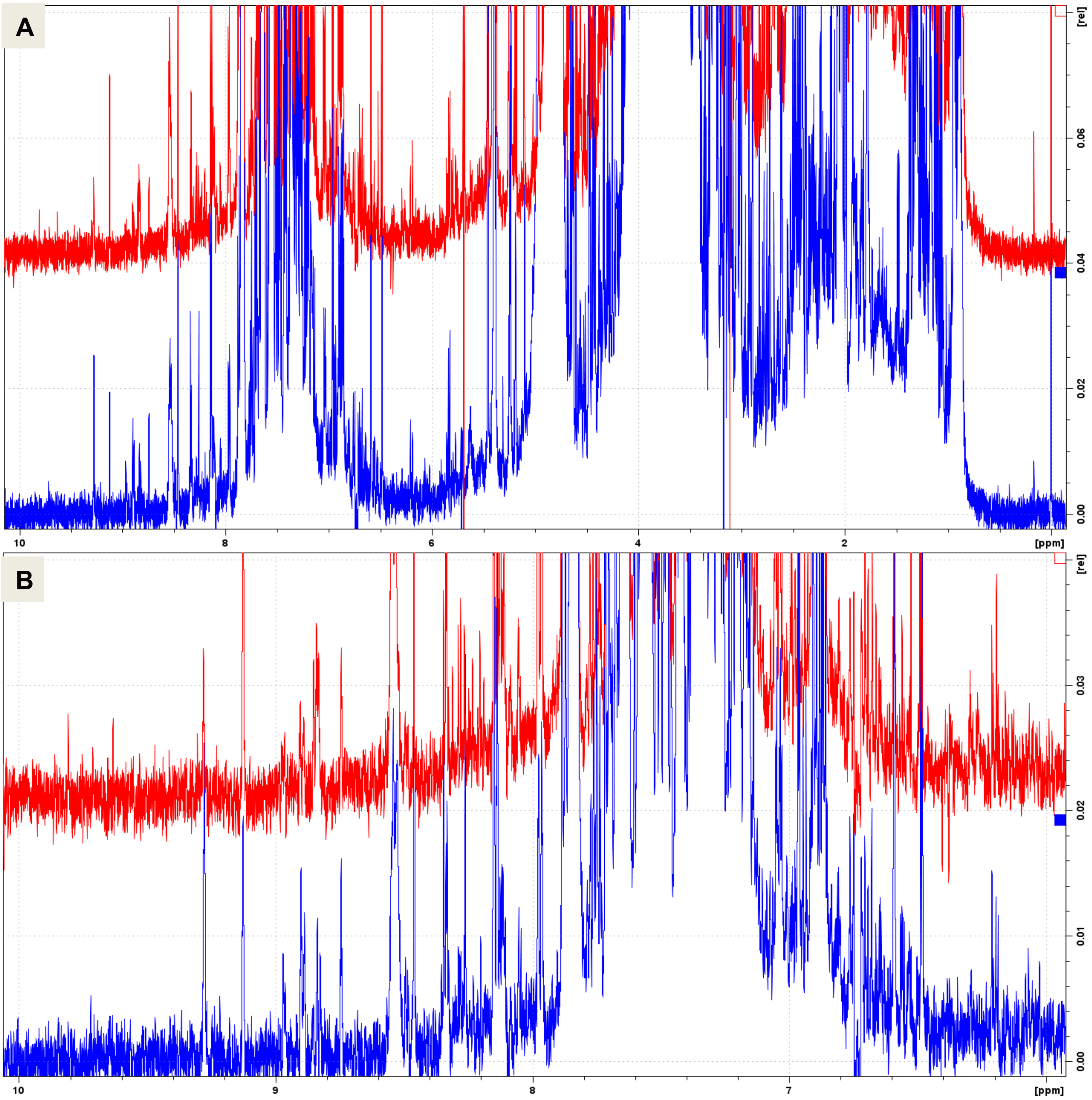

Fig AH. Representative ^{1}H NMR CPMG Spectra of 5-Month Female Urine Samples. (A) Image displays the control spectra in blue and the study spectra in red. (B) Zoomed in image of the control (blue) and red (study) spectra so all visual differences are easier to be seen.

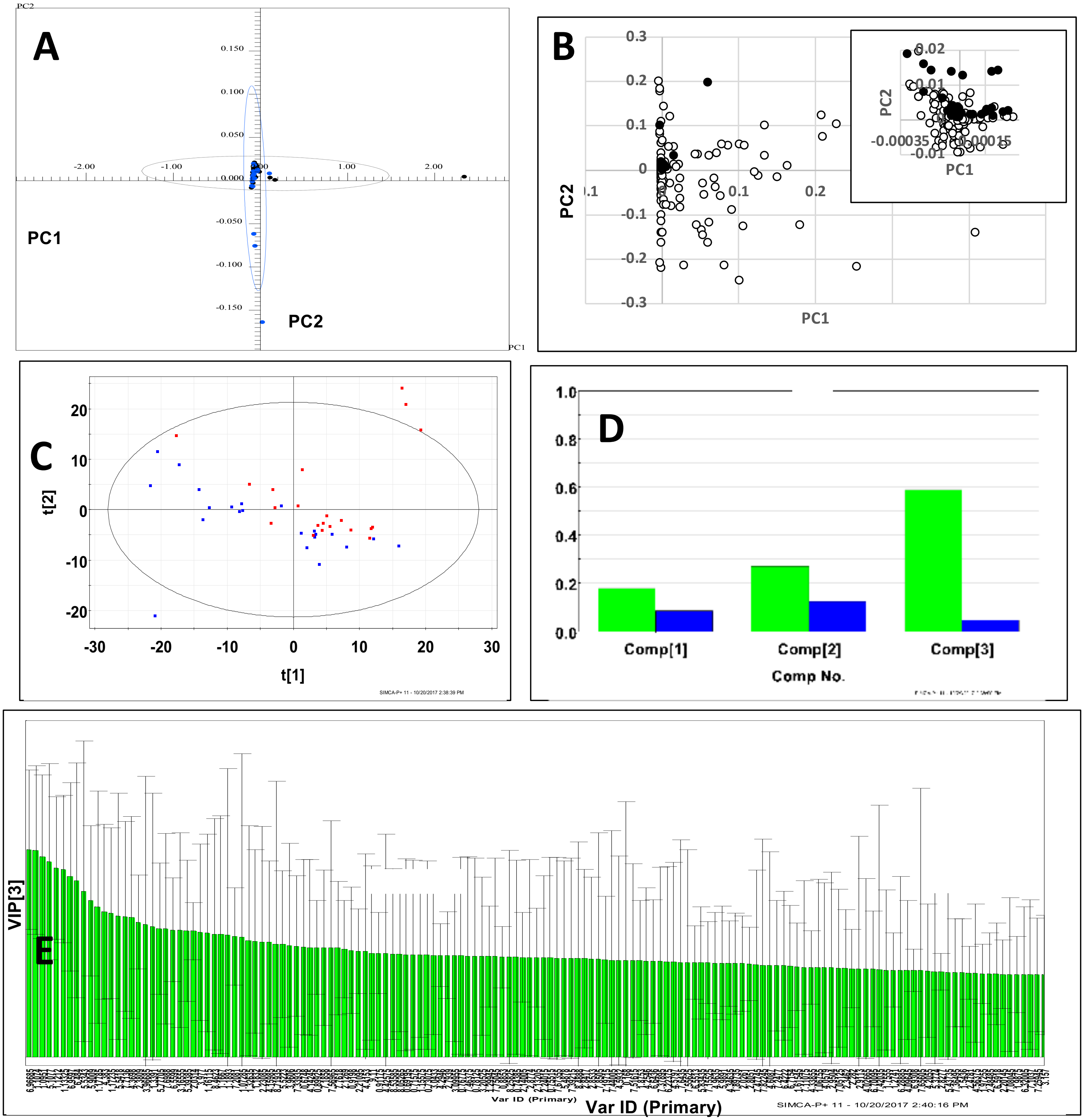

Fig AI. PCA and PLS-DA of Female 5-Month Urine Samples. (A) PCA scores plot analysis showing no separation between the control and study groups. (B) PCA loadings plot displaying the color-coded buckets. Red (10-9 – 10-13), yellow (10-9 – 10-7), green (10-7 – 10-5), blue (10-5 – 1.10X10-4), black closed (<0.05), black open (>0.05). (C) PLS-DA scores plot. (D) A plot of the first three PCs displaying the R2Y explained variation and the Q2 predicted variation. (E) VIP numbers from the PLS-DA analysis that are greater than or less than 1.

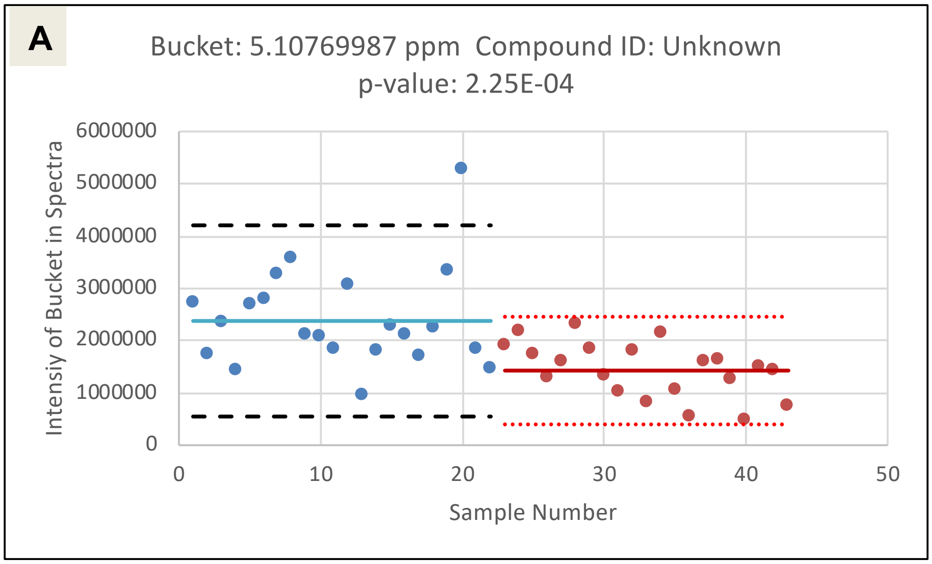

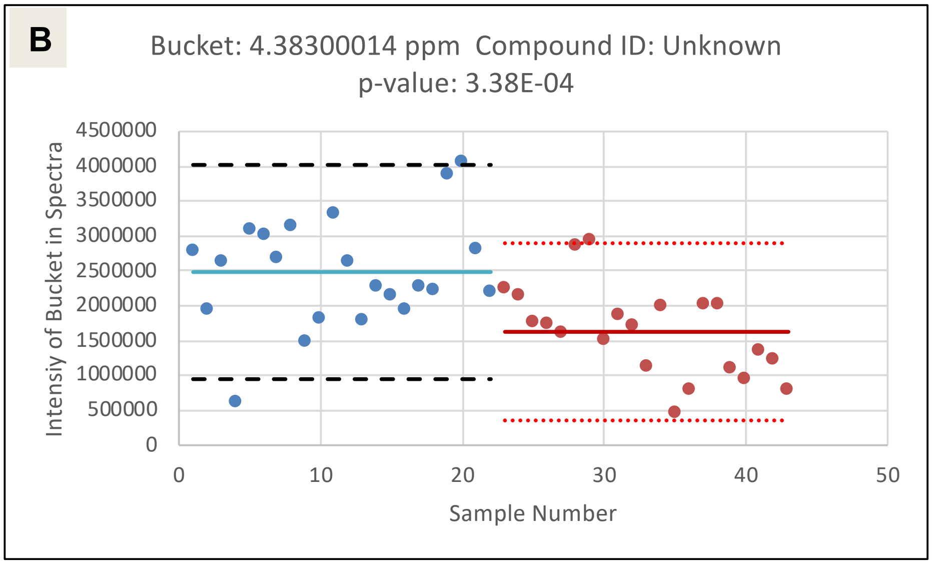

Fig AJ. Intensity Plots of 5-Month Old Female Urine Samples. (A) Unidentified bucket at 5.11 ppm with a p-value of 2.25E-04. (B) Unidentified bucket at 4.38 ppm with a p-value of 3.38E-04.

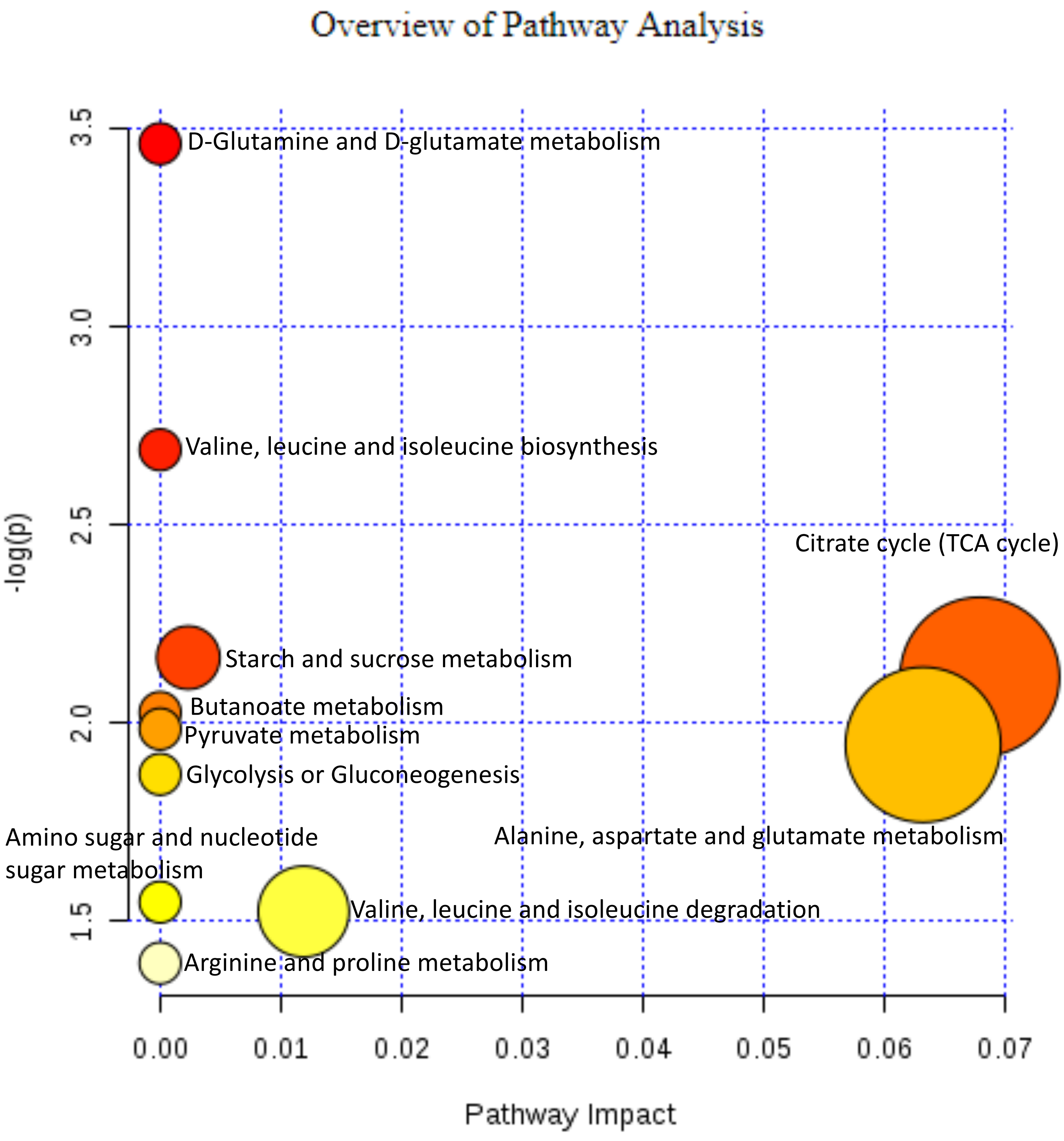

Fig AK. Pathway Analysis from 5-Month Female Urine Samples. The "metabolome view" from MetaboAnalyst 3.0 showing the pathway impact on the x-axis versus the negative log p values on the y-axis for the metabolic pathways. Pathway names have been added.

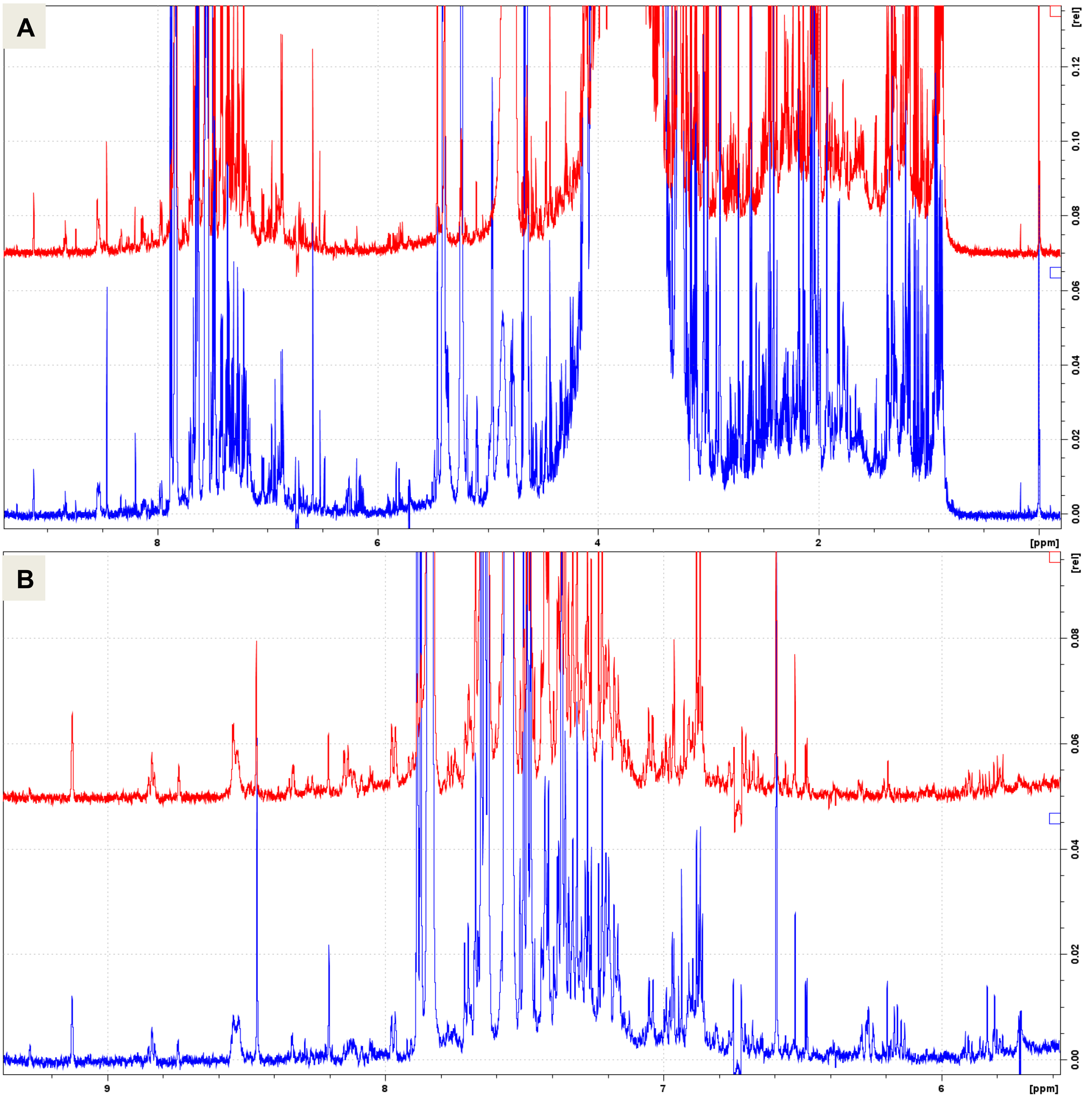

Fig AL. Representative ^{1}H NMR CPMG Spectra of 5-Month Male Urine Samples. (A) Image displays the control spectra in blue and the study spectra in red. (B) Zoomed in image of the control (blue) and red (study) spectra so all visual differences are easier to be seen.

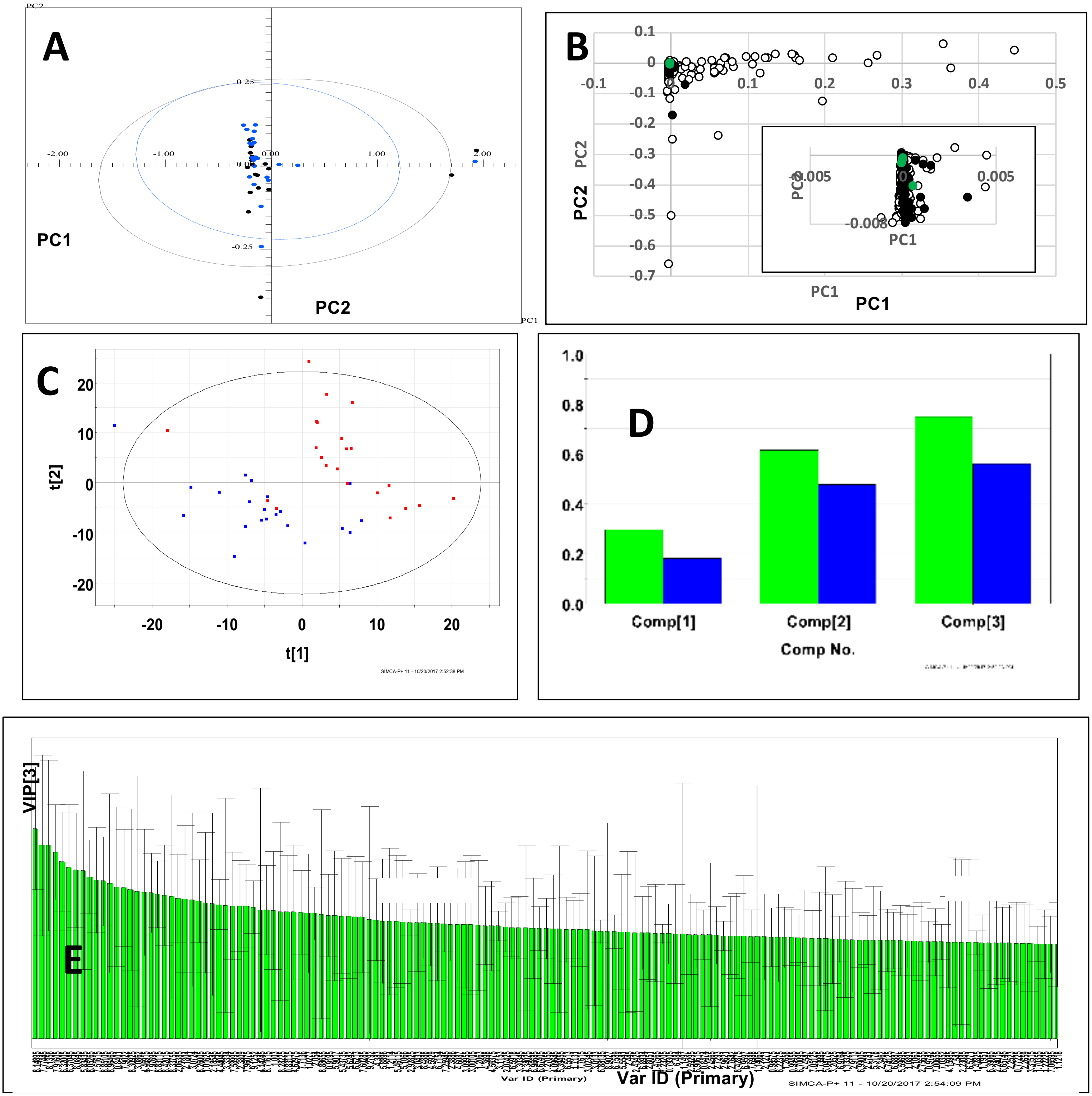

Fig AM. PCA and PLS-DA of Male 5-Month Urine Samples. (A) PCA scores plot for comparison between the control and study groups. (B) PCA loadings plot displaying the color-coded buckets. Red (10-9 – 10-13), yellow (10-9 – 10-7), green (10-7 – 10-5), blue (10-5 – 1.12X10-4), black closed (<0.05), black open (>0.05). (C) PLS-DA scores plot. (D) A plot of the first three PCs displaying the R2Y explained variation and the Q2 predicted variation. (E) VIP numbers from the PLS-DA analysis that are greater than or less than 1.

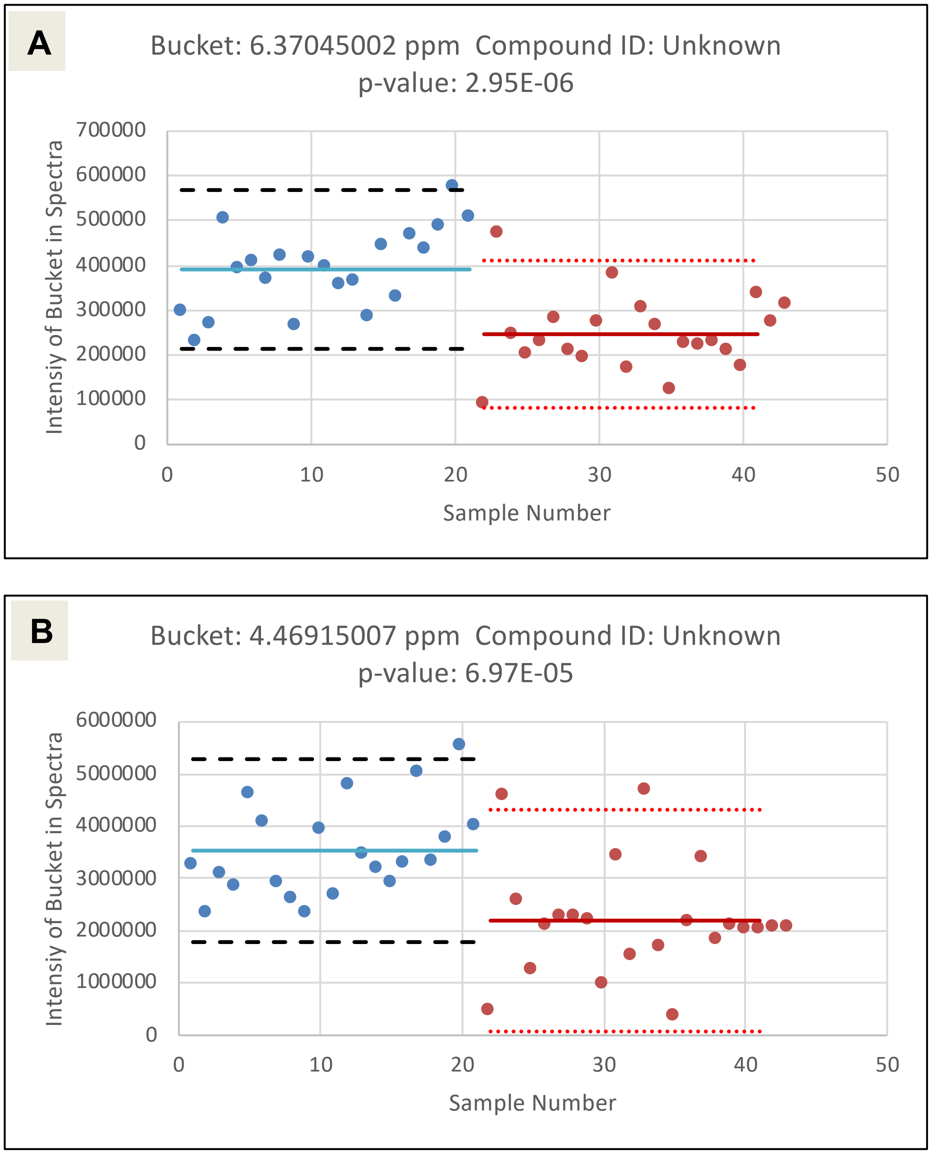

Fig AN. Intensity Plots of 5-Month Male Urine Samples. (A) Unidentified bucket at 6.37 ppm with a p-value of 2.95E-06. (B) Unidentified bucket at 4.47 ppm with a p-value of 6.97E-05.

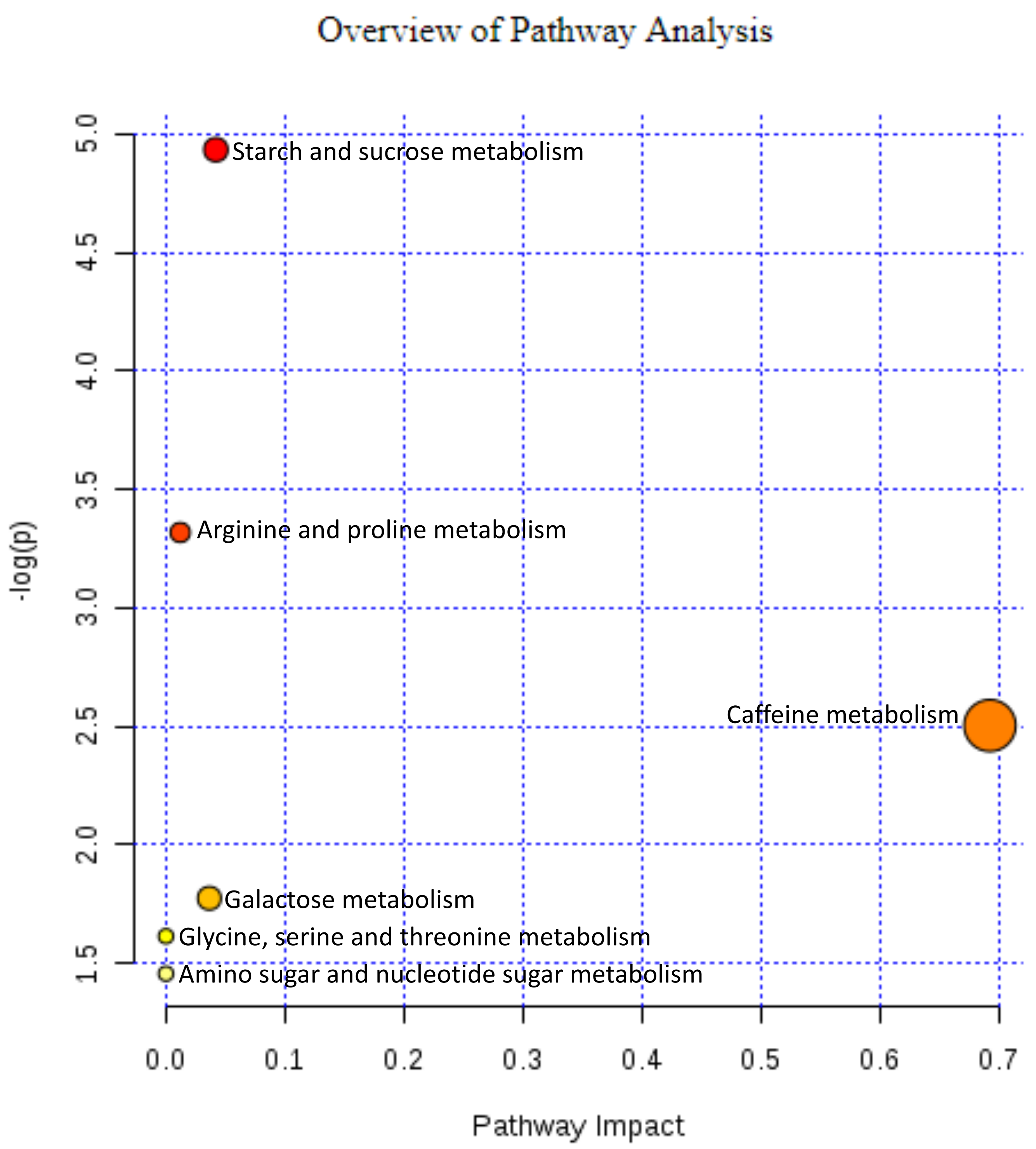

Fig AO. Pathway Analysis from the 5-Month Male Urine Samples. The "metabolome view" from MetaboAnalyst 3.0 showing the pathway impact on the x-axis versus the negative log p values on the y-axis for the metabolic pathways. Pathway names have been added.

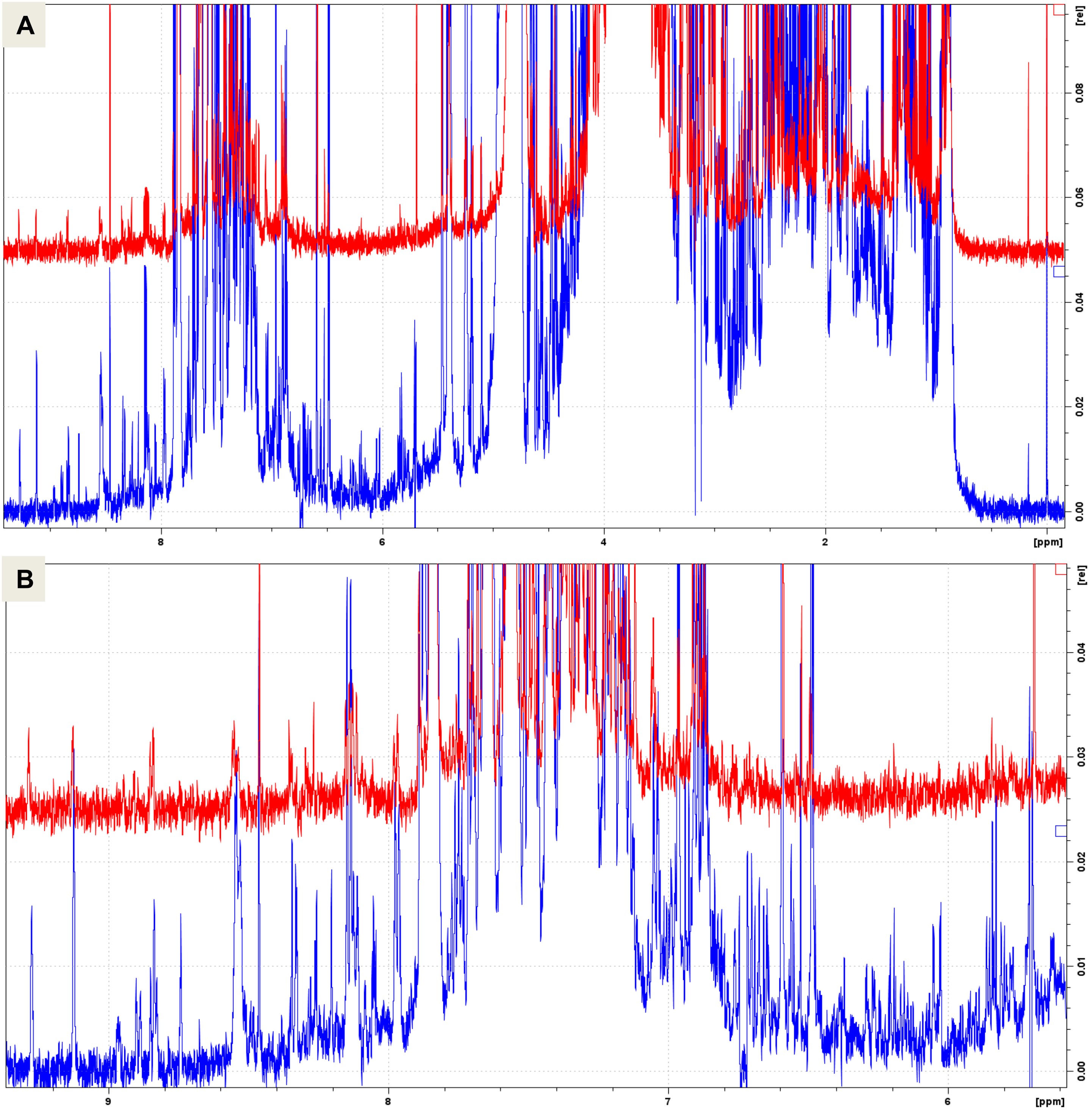

Fig AP. Representative ^{1}H NMR CPMG Spectra of 11-Month Female Urine Samples. (A) Image displays the control spectra in blue and the study spectra in red. (B) Zoomed in image of the control (blue) and red (study) spectra so all visual differences are easier to be seen.

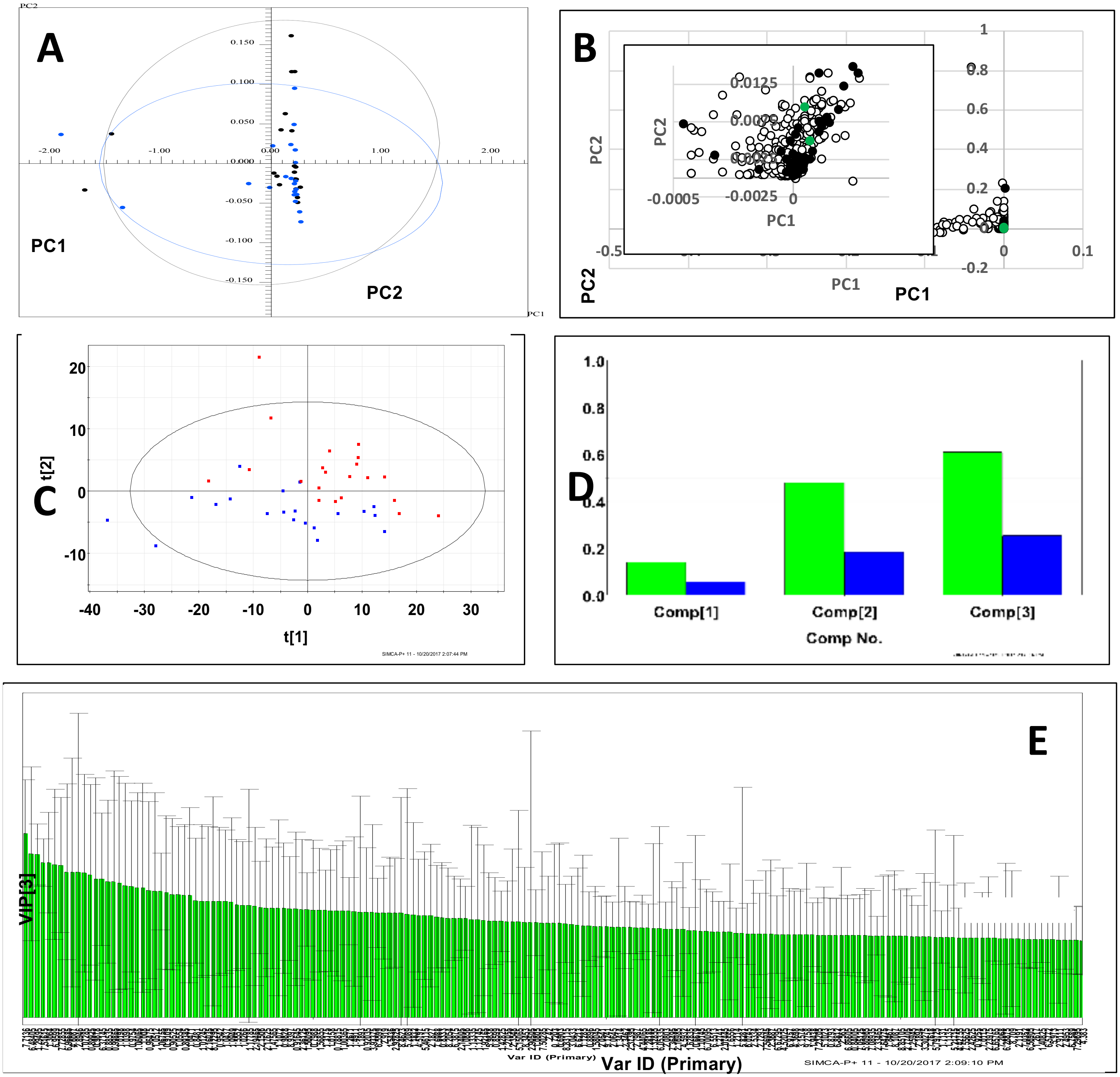

Fig AQ. PCA and PLS-DA of Female 11-Month Urine Samples. (A) PCA scores plot for comparison between the control and study groups. (B) PCA loadings plot displaying the color-coded buckets. Red (10-9 – 10-13), yellow (10-9 – 10-7), green (10-7 – 10-5), blue (10-5 – 1.02X10-4), black closed (<0.05), black open (>0.05). (C) PLS-DA scores plot. (D) A plot of the first three PCs displaying the R2Y explained variation and the Q2 predicted variation. (E) VIP numbers from the PLS-DA analysis that are greater than or less than 1.

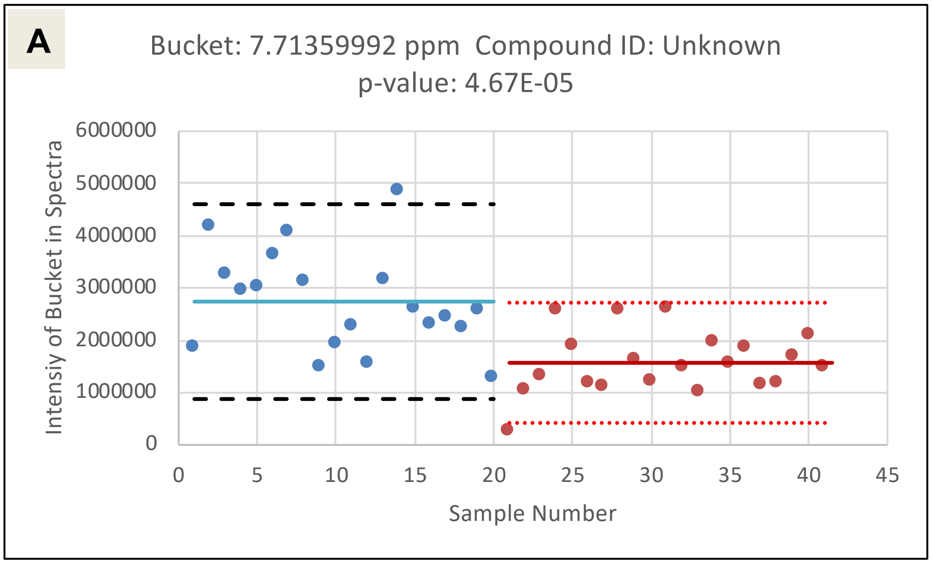

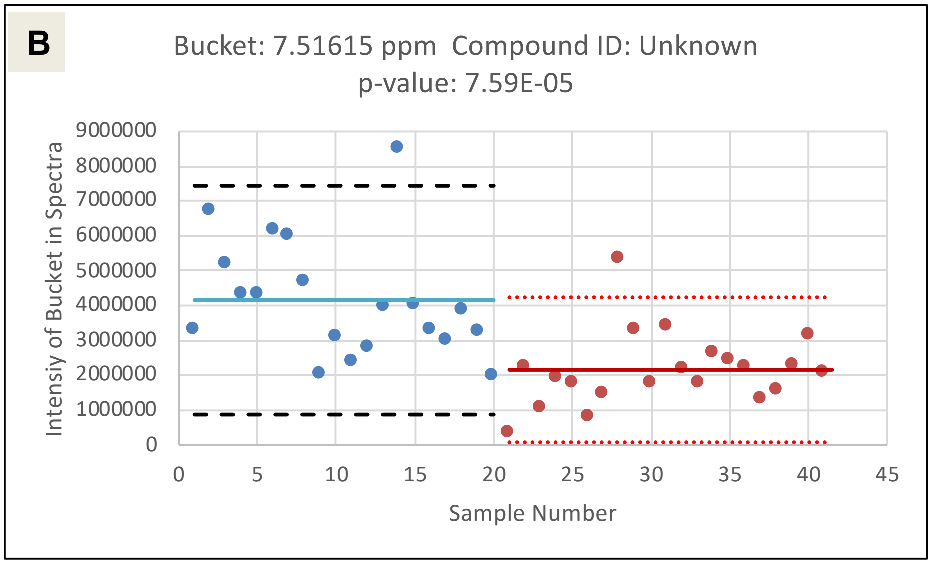

Fig AR. Intensity Plots of 11-Month Female Urine Samples. (A) Unidentified bucket at 7.71 ppm with a p-value of 4.67E-05. (B) Unidentified bucket at 7.51 ppm with a p-value of 7.59E-05.

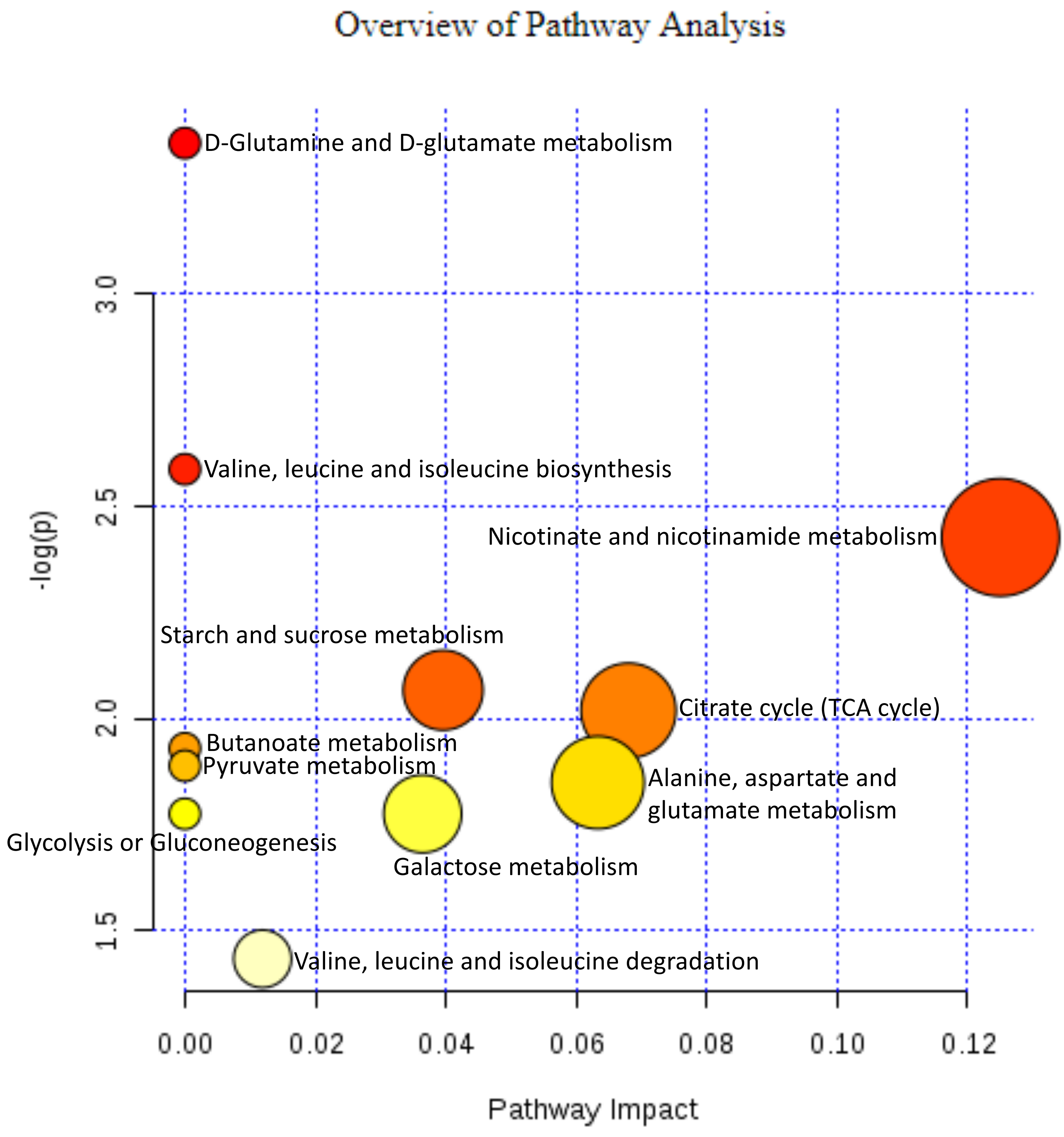

Fig AS. Pathway Analysis from 15-Month Female Urine Samples. The "metabolome view" from MetaboAnalyst 3.0 showing the pathway impact on the x-axis versus the negative log p values on the y-axis for the metabolic pathways. Pathway names have been added.

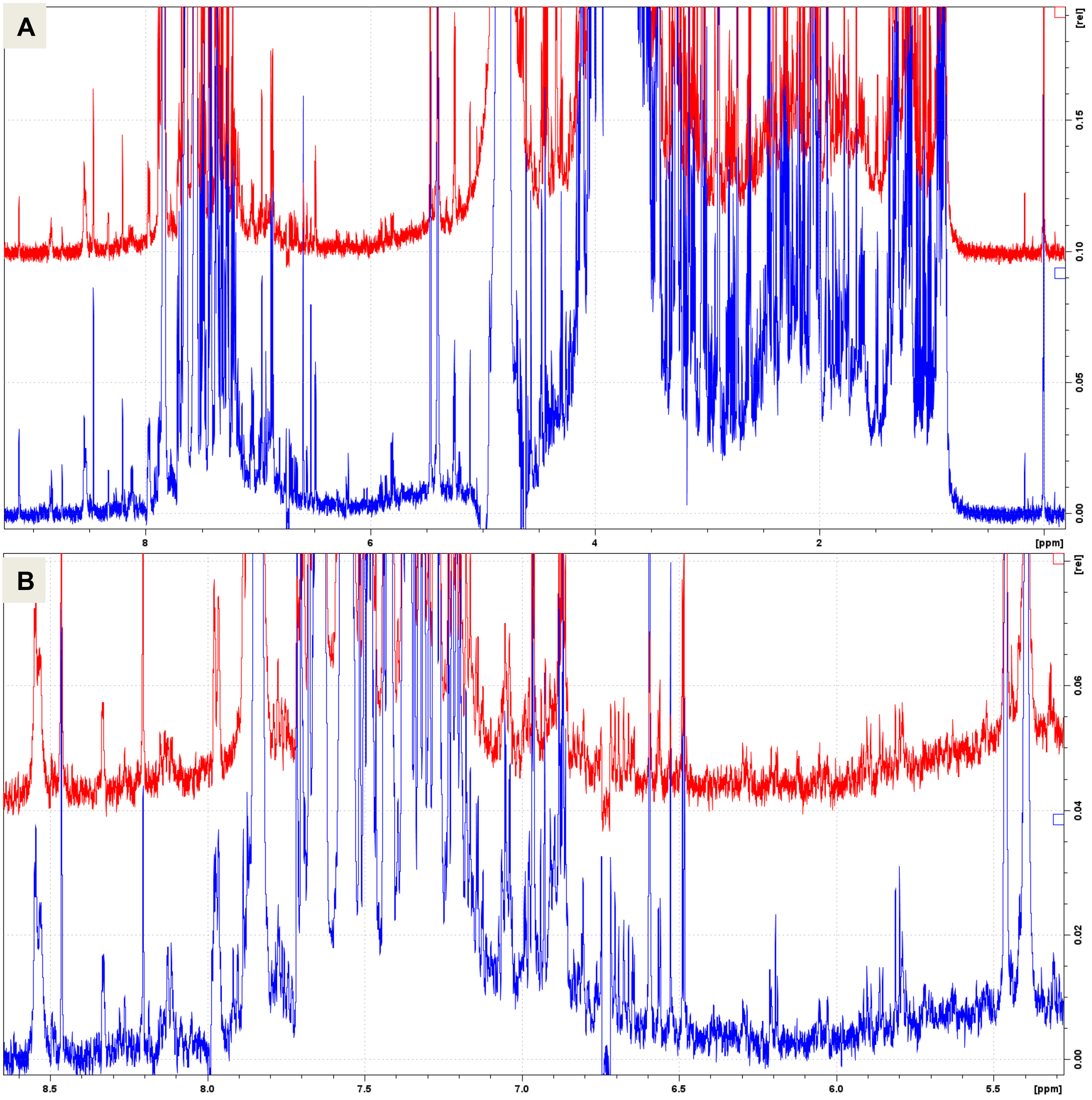

Fig AT. Representative ^{1}H NMR CPMG spectra of 11-Month Male Urine Samples. (A) Image displays the control spectra in blue and the study spectra in red. (B) Zoomed in image of the control (blue) and red (study) spectra so all visual differences are easier to be seen.

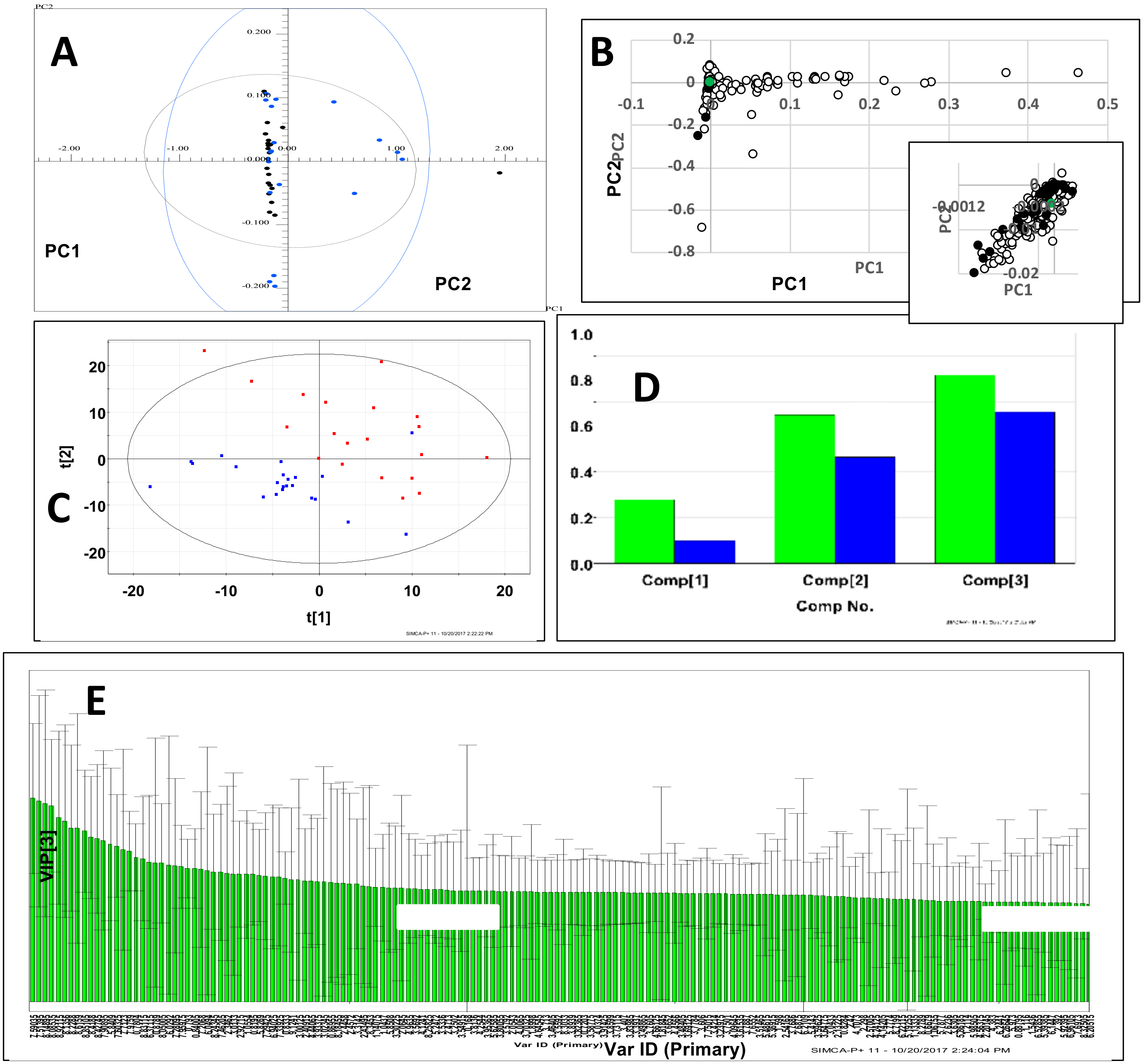

Fig AU. PCA and PLS-DA of Male 11-Month Urine Samples. (A) PCA scores plot for comparison between the control and study groups. (B) PCA loadings plot displaying the color-coded buckets. Red (10-9 – 10-13), yellow (10-9 – 10-7), green (10-7 – 10-5), blue (10-5 – 1.10X10-4), black closed (<0.05), black open (>0.05). (C) PLS-DA scores plot. (D) A plot of the first three PCs displaying the R2Y explained variation and the Q2 predicted variation. (E) VIP numbers from the PLS-DA analysis that are greater than or less than 1.

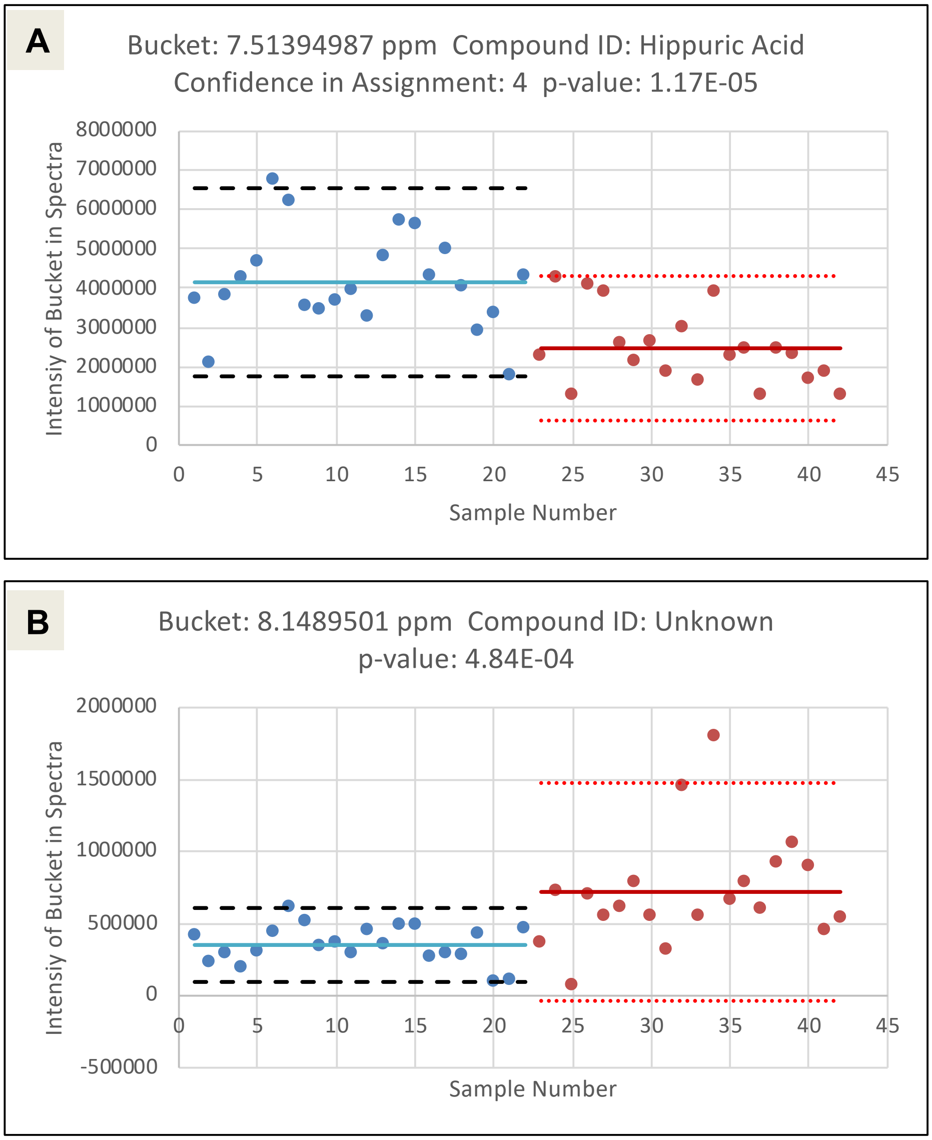

Fig AV. Intensity Plots of 11-Month Male Urine Samples. (A) Unidentified bucket at 7.51 ppm with a p-value of 1.17E-05. (B) Unidentified bucket at 8.15 ppm with a p-value of 8.84E-04.

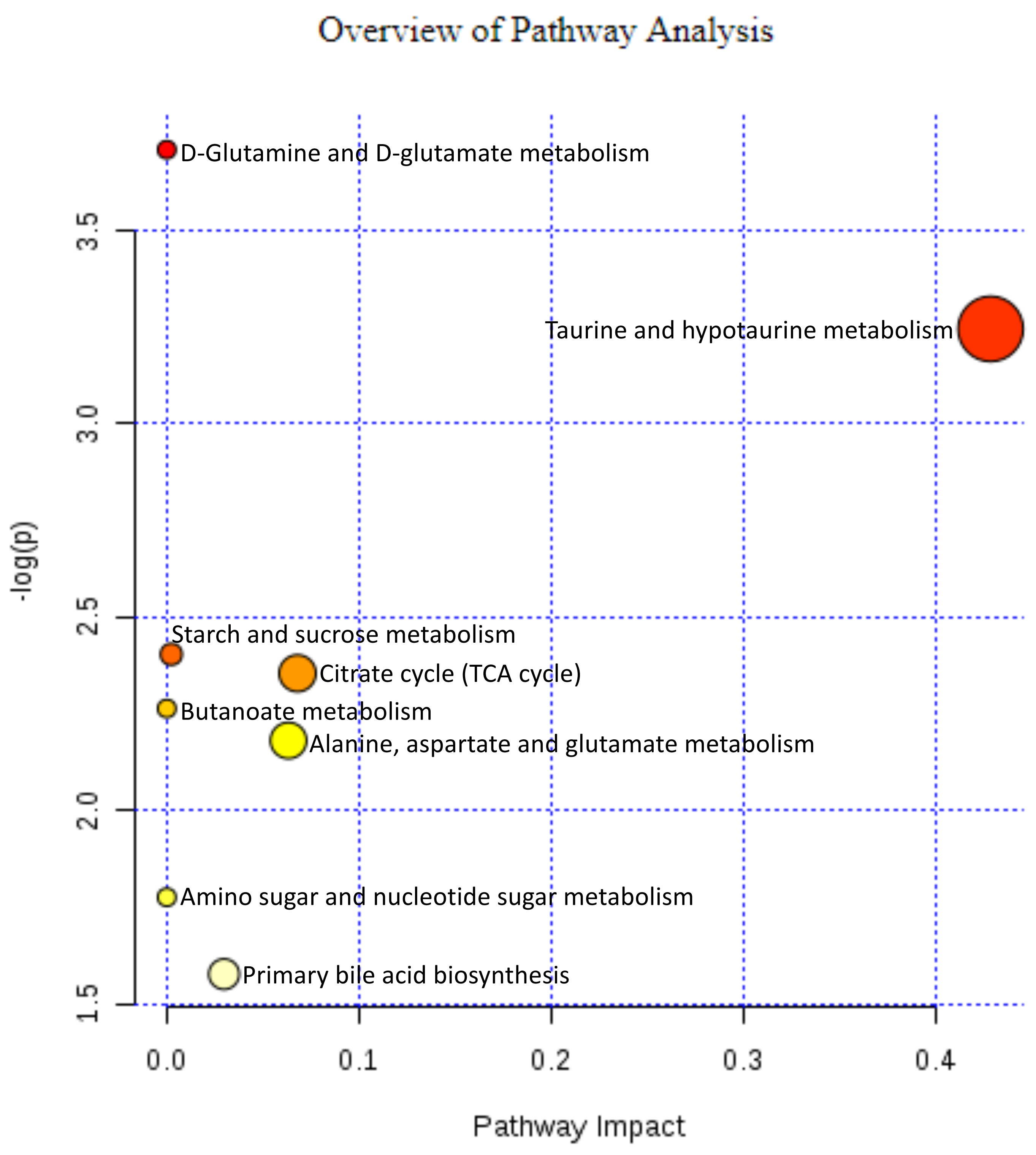

Fig AW. Pathway Analysis from the 11-Month Male Urine Samples. The "metabolome view" from MetaboAnalyst 3.0 showing the pathway impact on the x-axis versus the negative log p values on the y-axis for the metabolic pathways. Pathway names have been added.

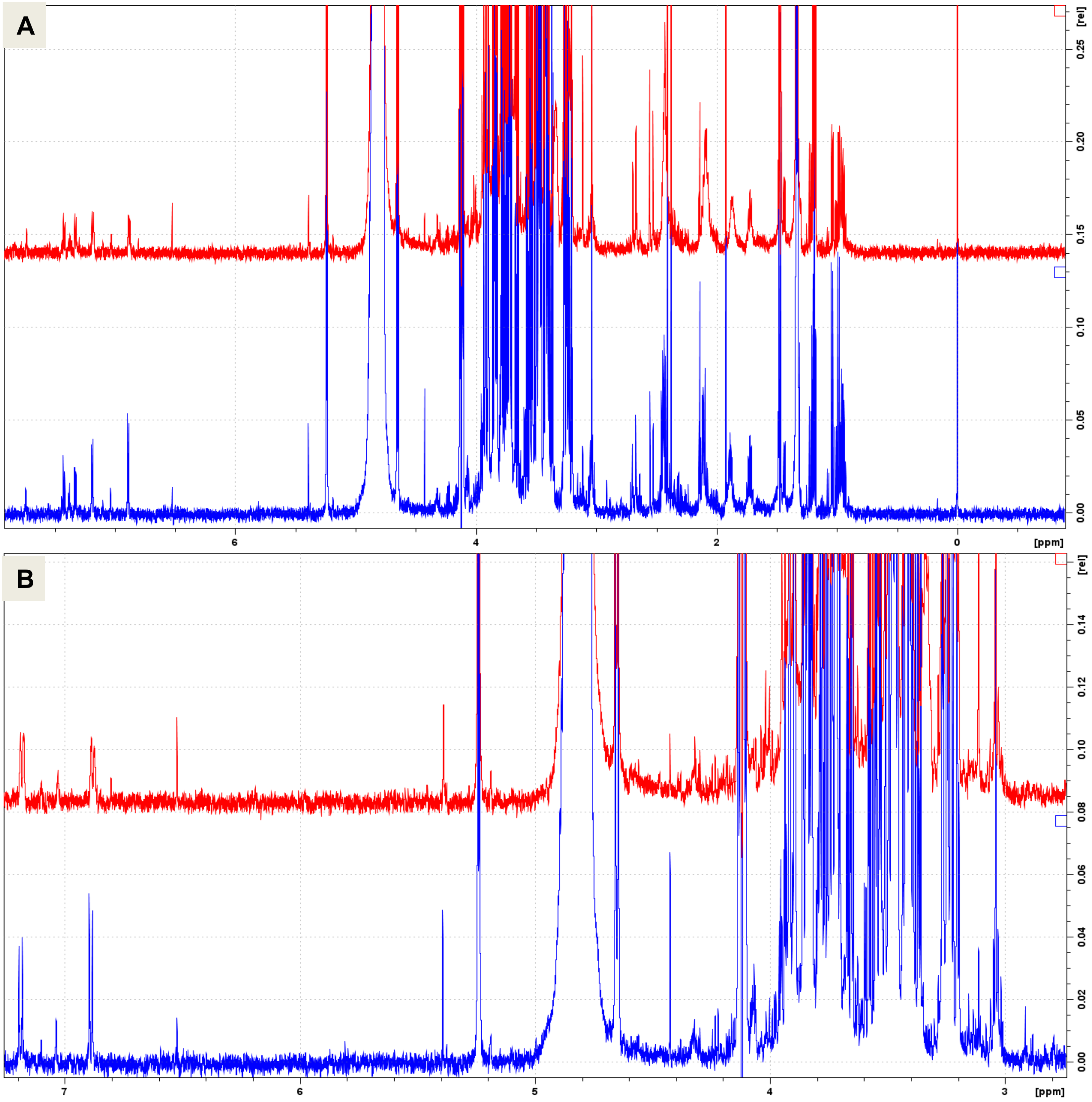

Fig AX. Representative ^{1}H NMR CPMG Spectra of 5-Month Female Serum Samples. (A) Image displays the control spectra in blue and the study spectra in red. (B) Zoomed in image of the control (blue) and red (study) spectra so all visual differences are easier to be seen.

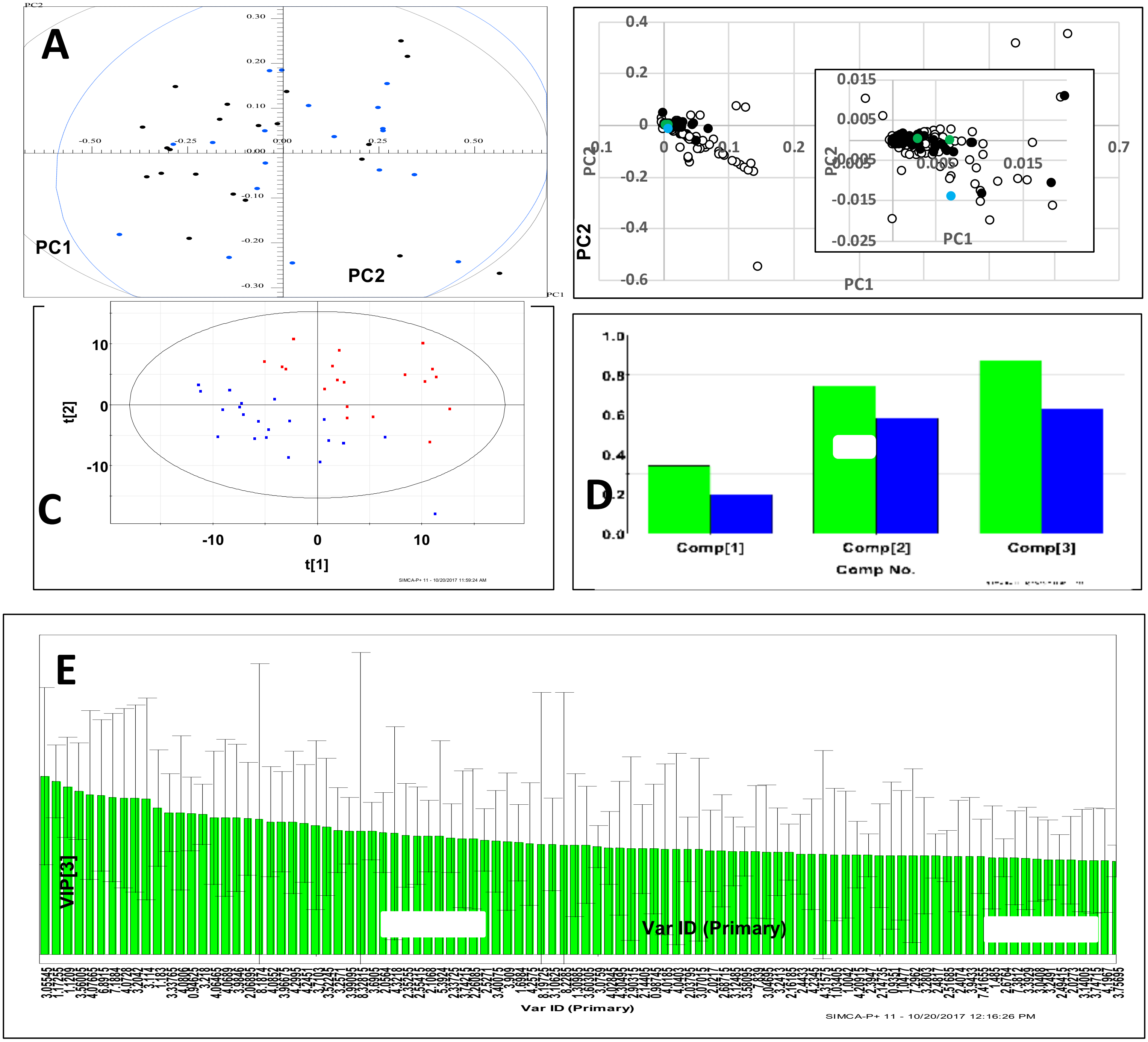

Fig AY. PCA and PLS-DA of Female 5-Month Serum Samples. (A) PCA scores plot analysis showing no separation between the control and study groups. (B) PCA loadings plot displaying the color-coded buckets. Red (10-9 – 10-13), yellow (10-9 – 10-7), green (10-7 – 10-5), blue (10-5 – 1.82X10-4), black closed (<0.05), black open (>0.05). (C) PLS-DA scores plot. (D) A plot of the first three PCs displaying the R2Y explained variation and the Q2 predicted variation. (E) VIP numbers from the PLS-DA analysis that are greater than or less than 1.

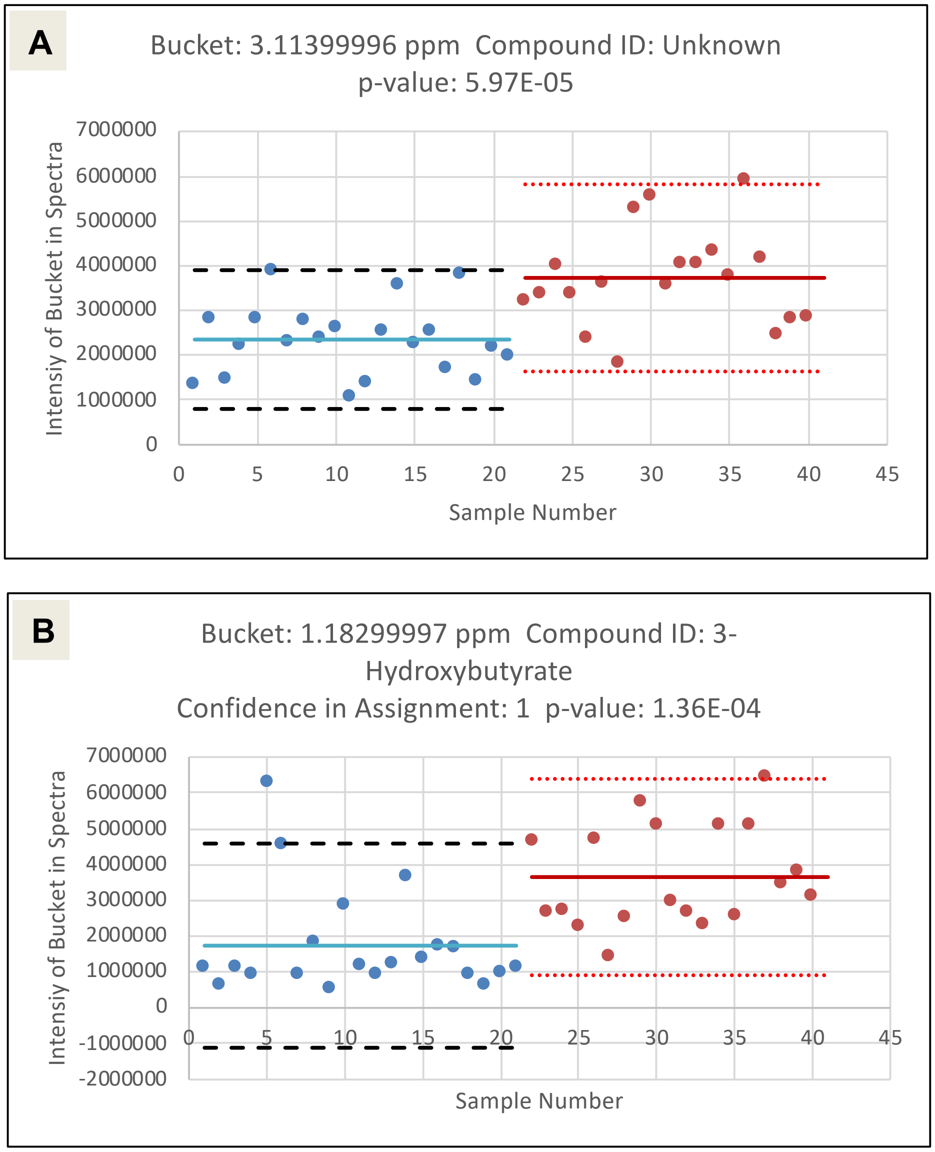

Fig AZ. Intensity Plots of 5-Month Female Serum Samples. (A) Unidentified bucket at 3.11 ppm with a p-value of 5.97E-05. (B) Bucket identified as 3-Hydroxybutyrate at 1.18 ppm with a p-value of 1.36E-04.

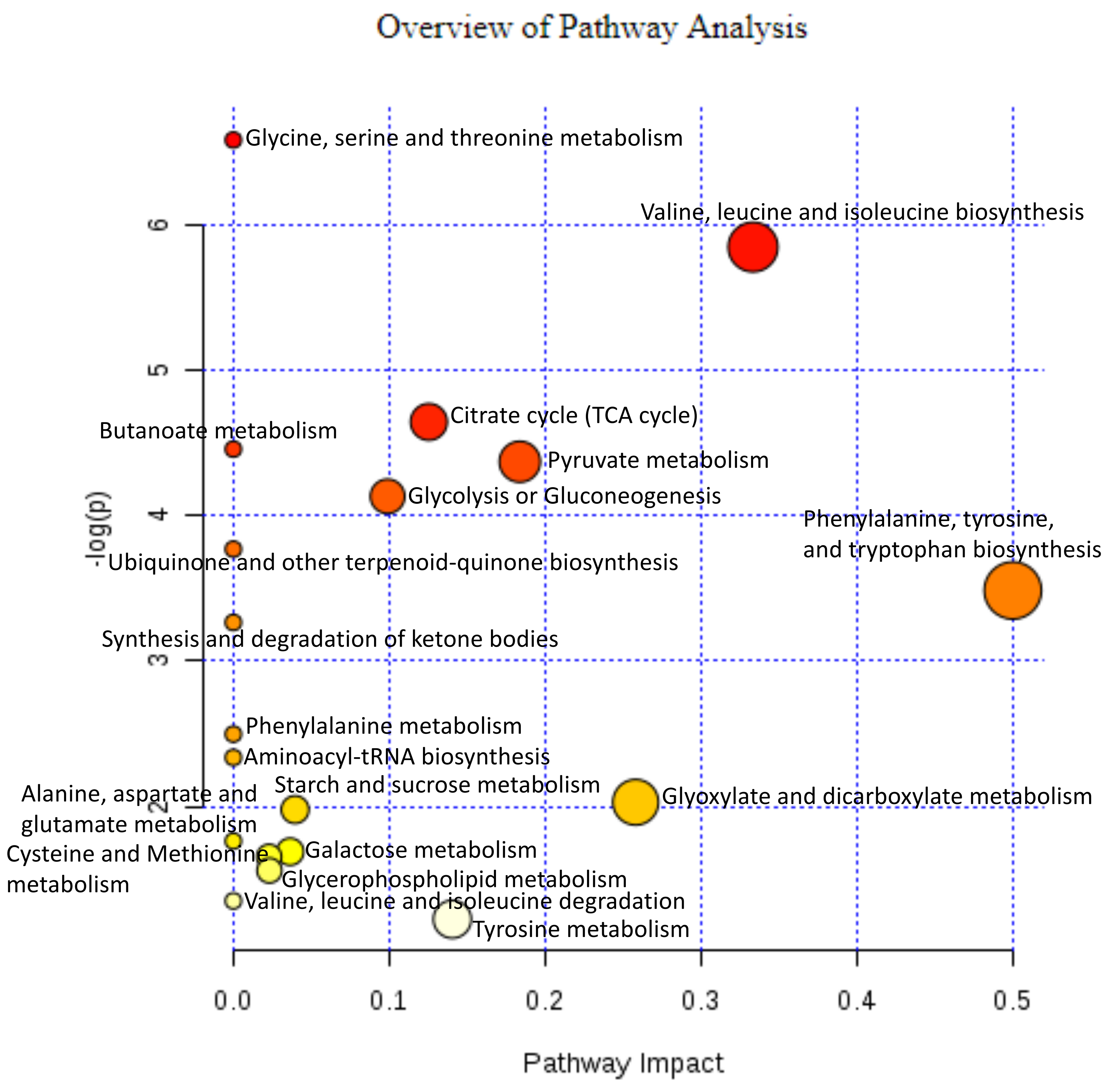

Fig BA. Pathway Analysis from 5-Month Female Serum Samples. The "metabolome view" from MetaboAnalyst 3.0 showing the pathway impact on the x-axis versus the negative log p values on the y-axis for the metabolic pathways. Pathway names have been added.

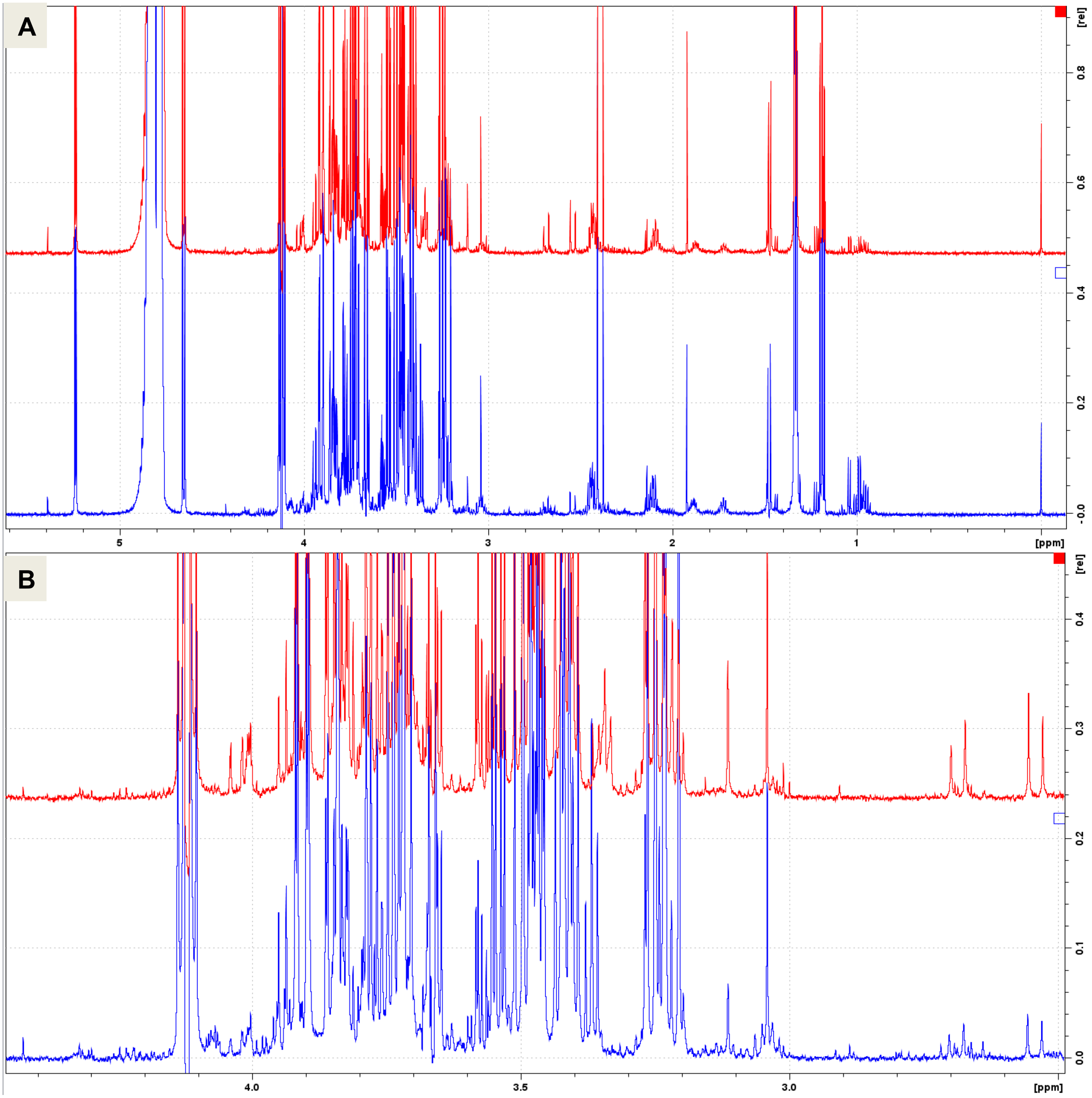

Fig BB. Representative ^{1}H NMR CPMG Spectra of 11-Month Serum Samples. Image displays the control spectra in blue and the study spectra in red. (B) Zoomed in image of the control (blue) and red (study) spectra so all visual differences are easier to be seen.

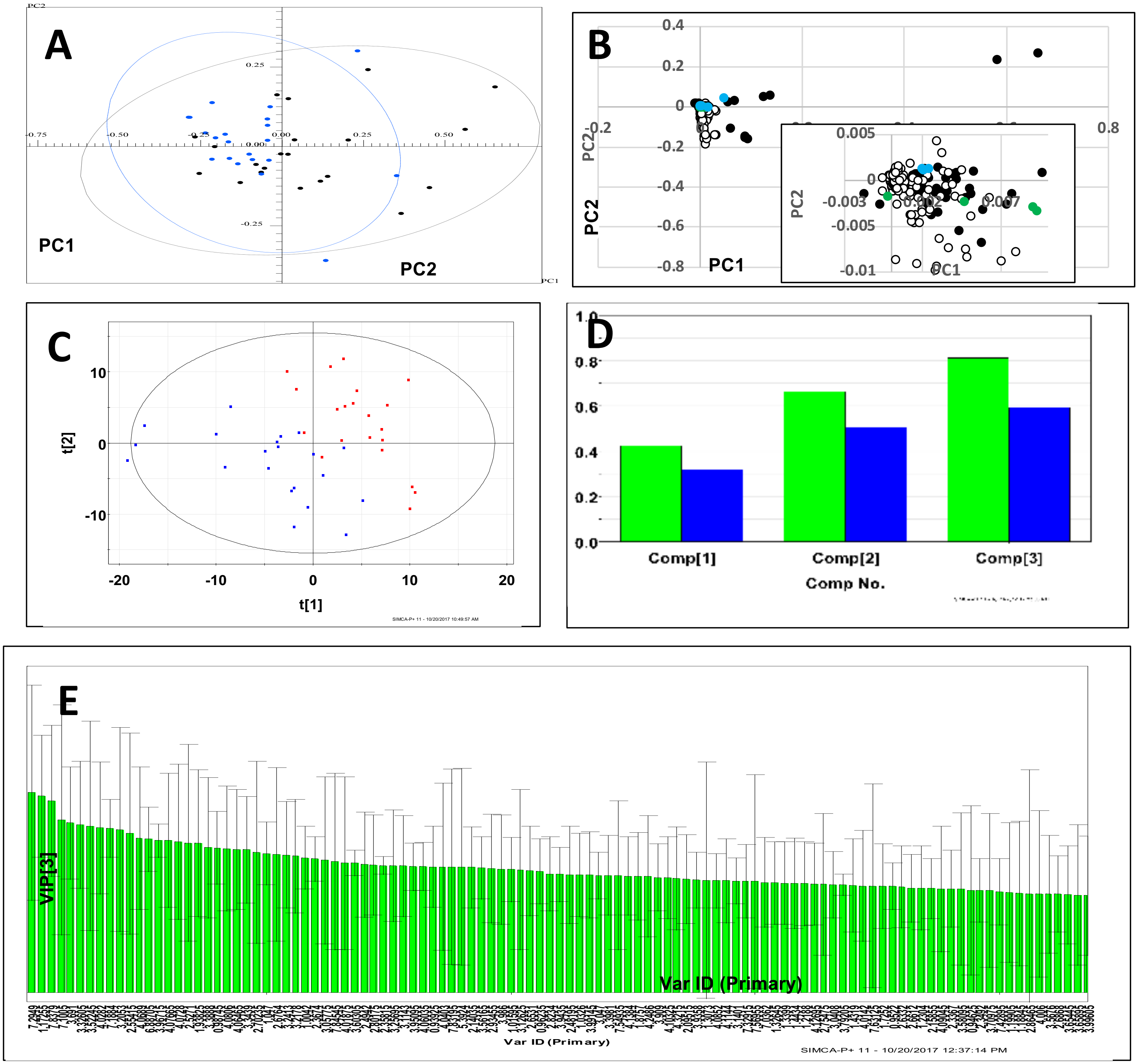

Fig BC. PCA and PLS-DA of female 5-Month Serum Samples. (A) PCA scores plot analysis showing no separation between the control and study groups. (B) PCA loadings plot displaying the color-coded buckets. Red (10-9 – 10-13), yellow (10-9 – 10-7), green (10-7 – 10-5), blue (10-5 – 1.82X10-4), black closed (<0.05), black open (>0.05). (C) PLS-DA scores plot. (D) A plot of the first three PCs displaying the R2Y explained variation and the Q2 predicted variation. (E) VIP numbers from the PLS-DA analysis that are greater than or less than 1.

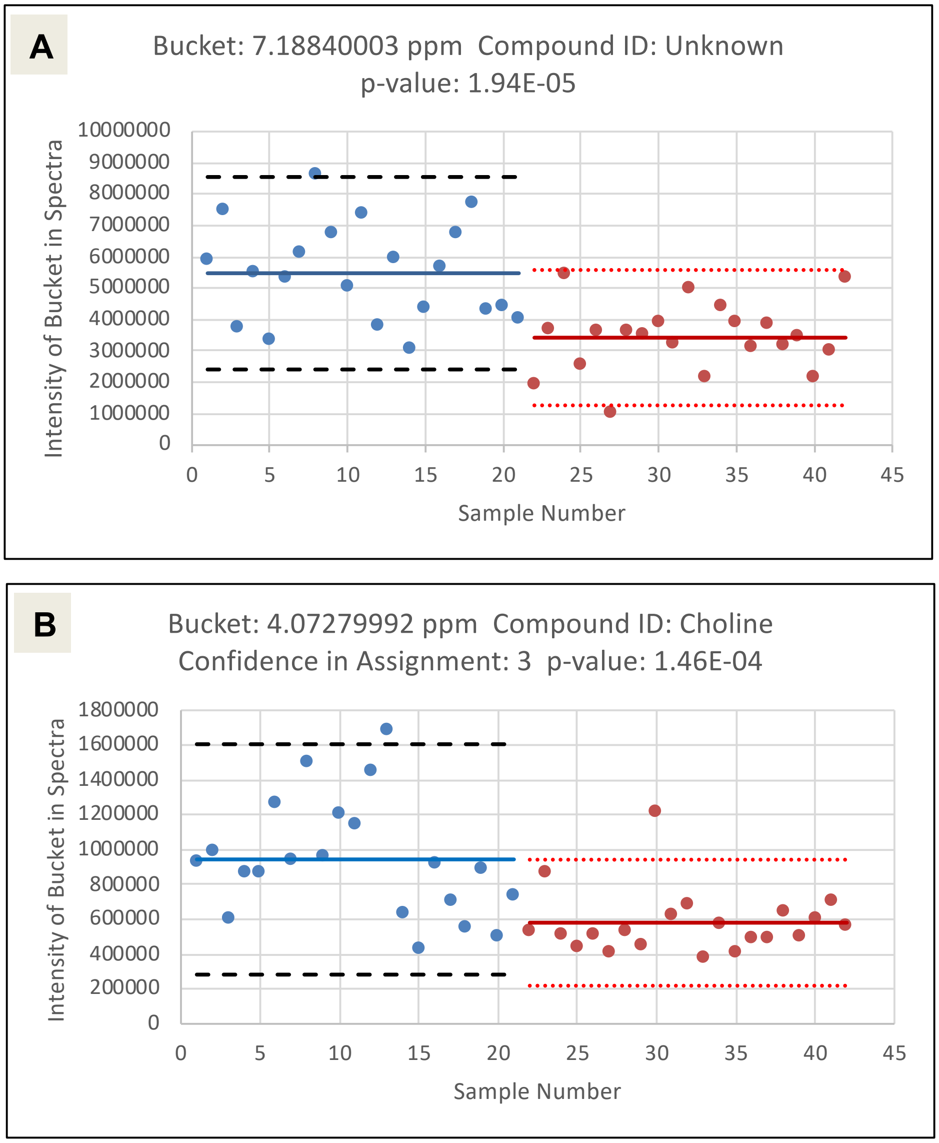

Fig BD. Intensity Plots of 11-Month Female Serum Samples. Unidentified bucket at 7.19 ppm with a p-value of 1.94E-05. (B) Bucket identified as choline at 4.07 ppm with a p-value of 1.46E-04.

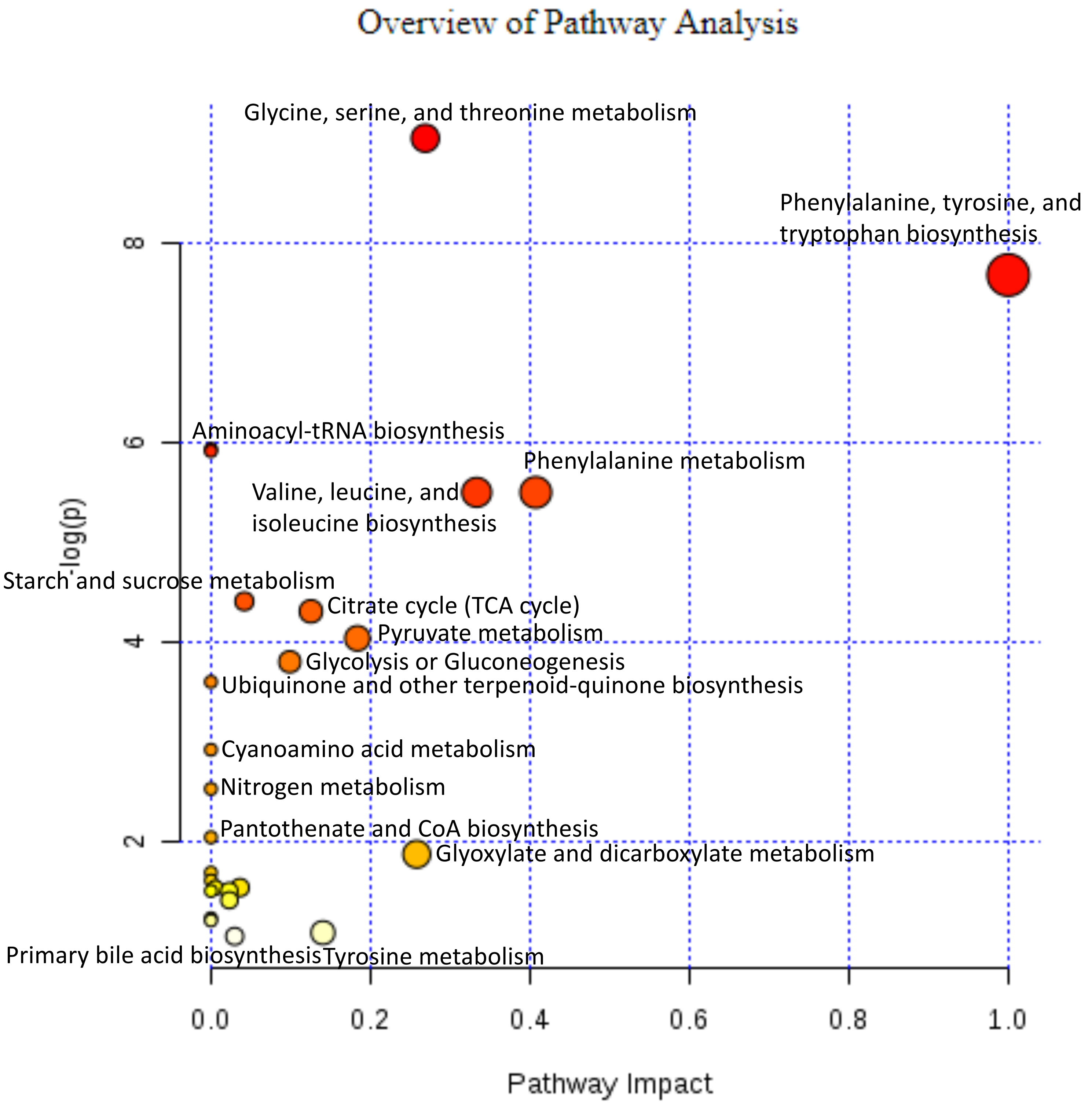

Fig BE. Pathway Analysis of 11-Month Female Serum Samples. The "metabolome view" from MetaboAnalyst 3.0 showing the pathway impact on the x-axis versus the negative log p values on the y-axis for the metabolic pathways. Pathway names have been added.

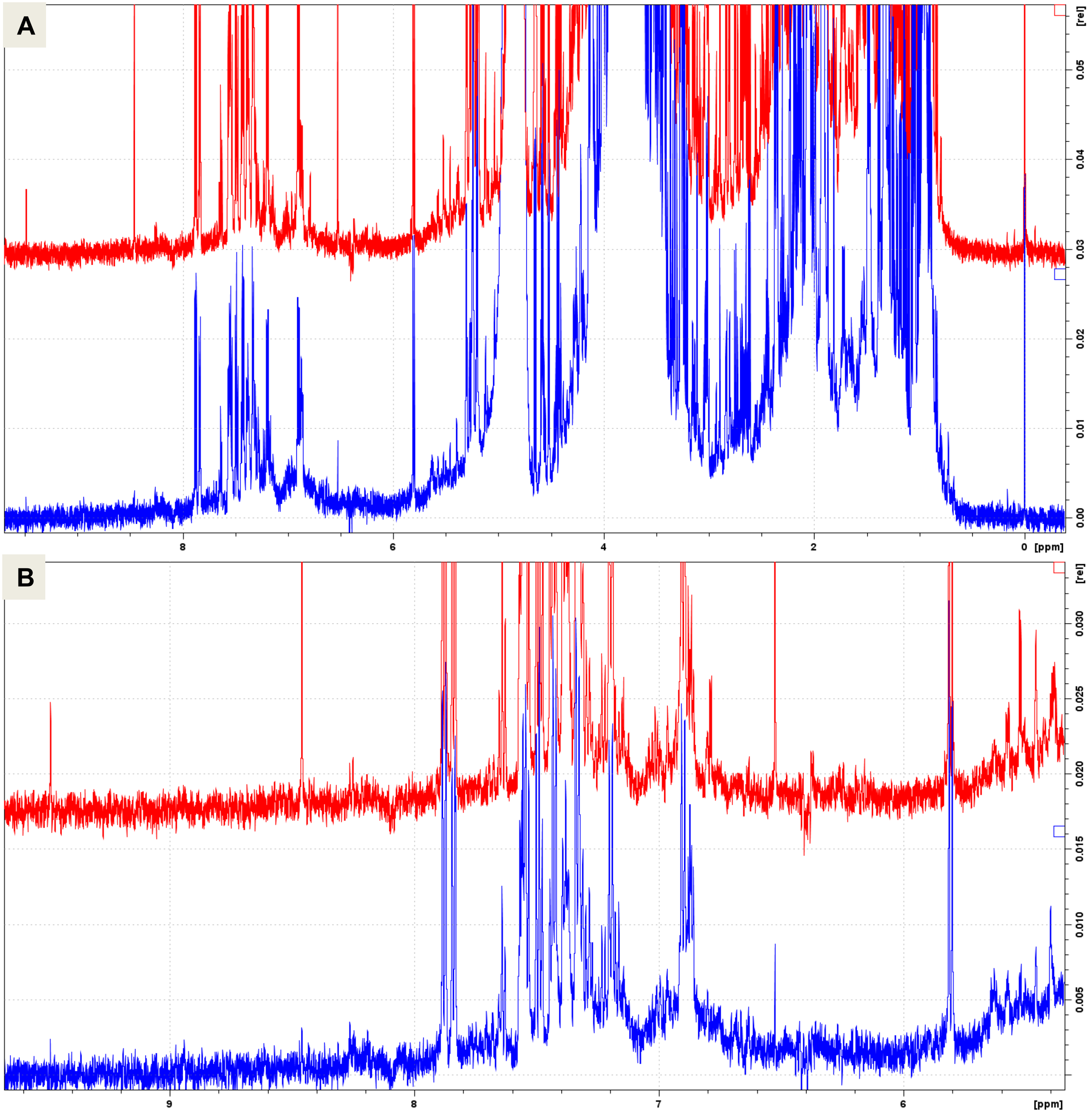

Fig BF. Representative ^{1}H NMR CPMG Spectra of 5-Month Female Fecal Samples. (A) Image displays the control spectra in blue and the study spectra in red. (B) Zoomed in image of the control (blue) and red (study) spectra so all visual differences are easier to be seen.

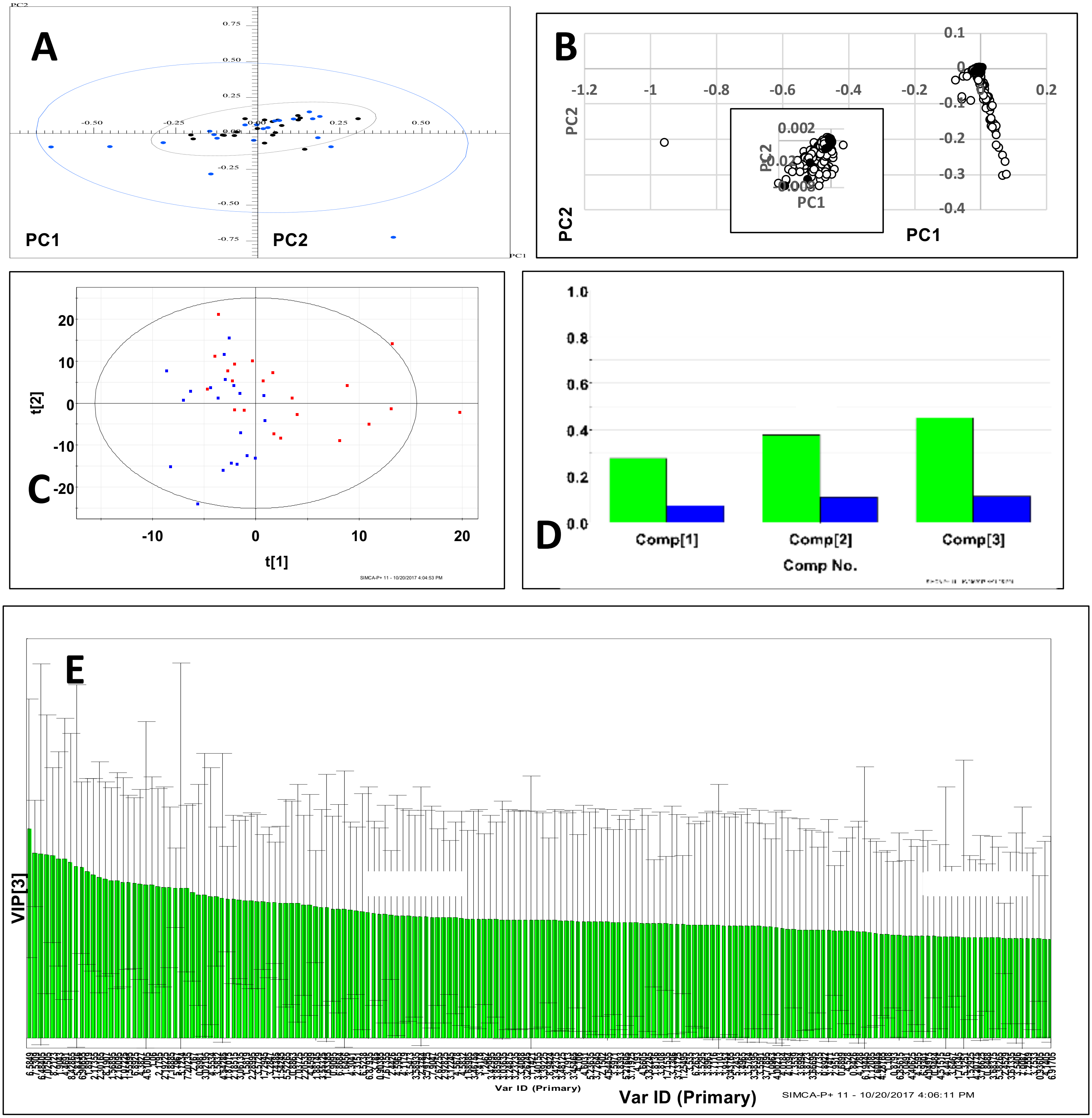

Fig BG. PCA and PLS-DA of the Female 5-Month Fecal Samples.

(A) PCA scores plot analysis showing no separation between the control and study groups. (B) PCA loadings plot displaying the color-coded buckets. Red (10-9 – 10-13), yellow (10-9 – 10-7), green (10-7 – 10-5), blue (10-5 – 1.15X10-4), black closed (<0.05), black open (>0.05). (C) PLS-DA scores plot. (D) A plot of the first three PCs displaying the R2Y explained variation and the Q2 predicted variation. (E) VIP numbers from the PLS-DA analysis that are greater than or less than 1.

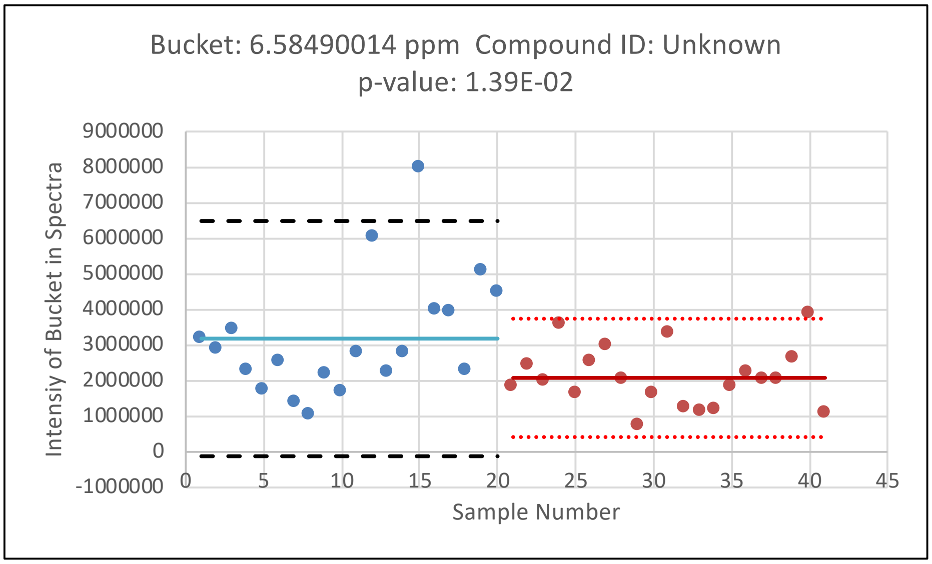

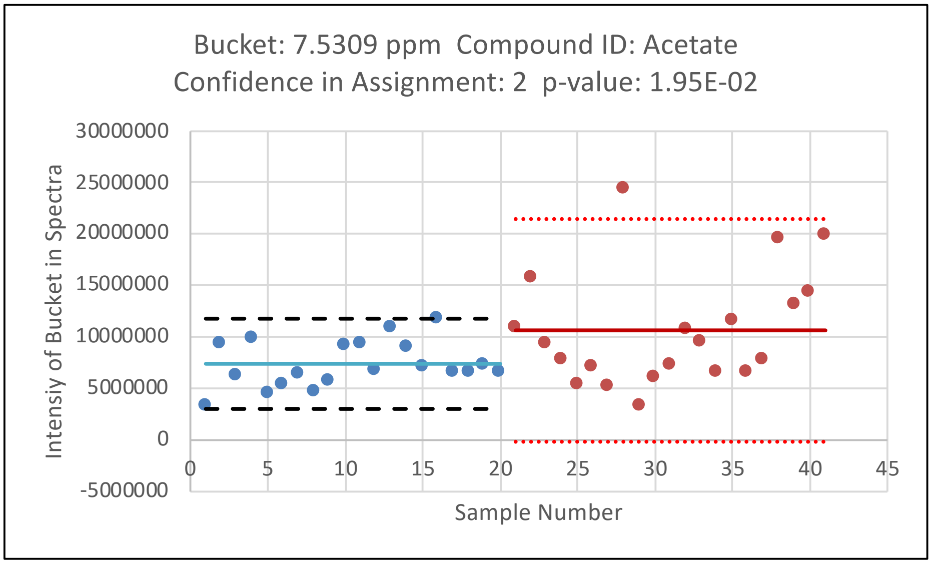

Fig BH. Intensity Plots of 5-Month Female Fecal Samples.
(A) Unidentified bucket at 6.58 ppm with a p-value of 1.39E-02.
(B) Bucket identified as Acetate at 7.53 ppm with a p-value of 1.95E-02.

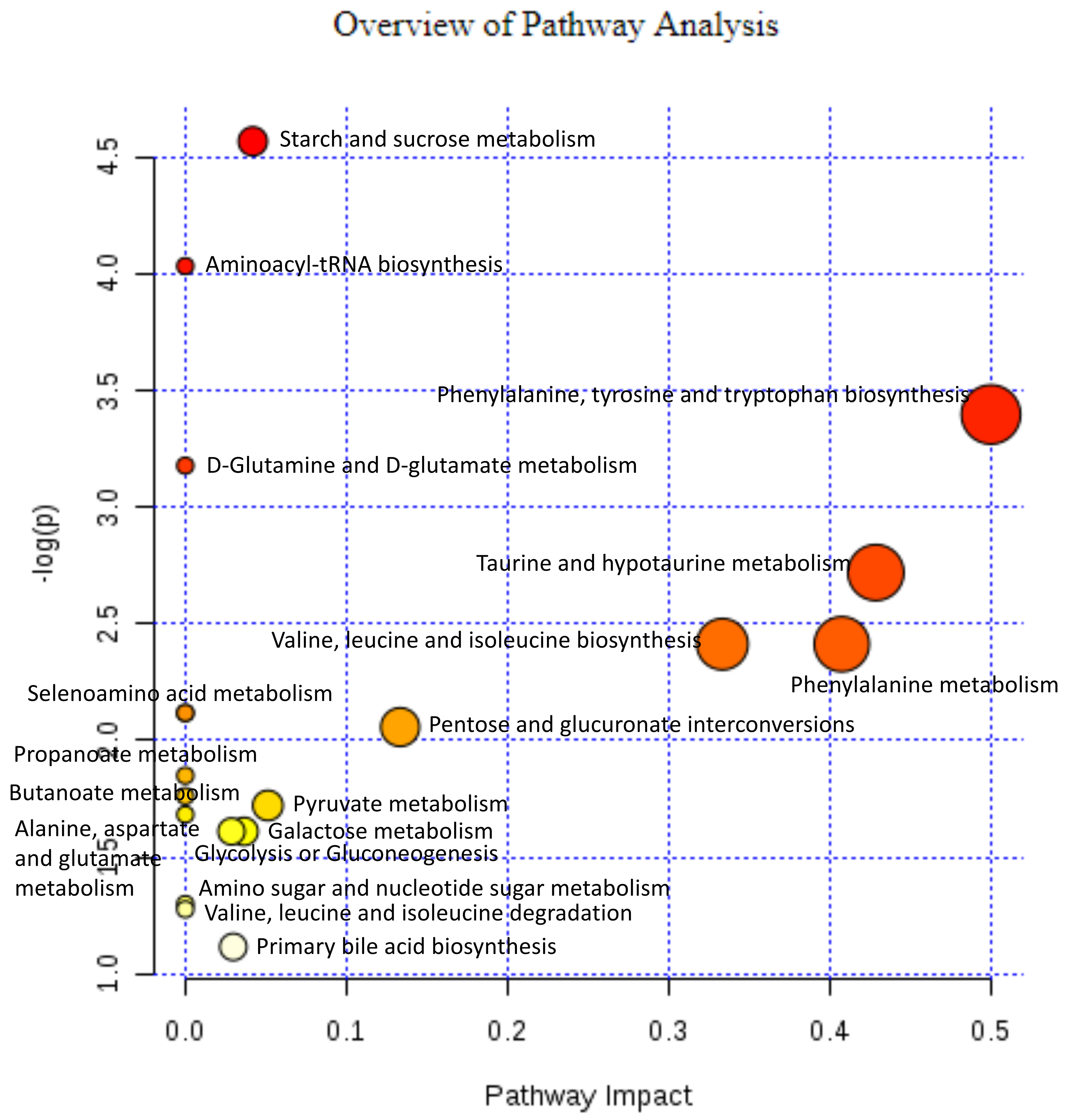

Fig BI. Pathway Analysis from the 5-Month Female Fecal Samples.
The "metabolome view" from MetaboAnalyst 3.0 showing the pathway impact on the x-axis versus the negative log p values on the y-axis for the metabolic pathways. Pathway names have been added.

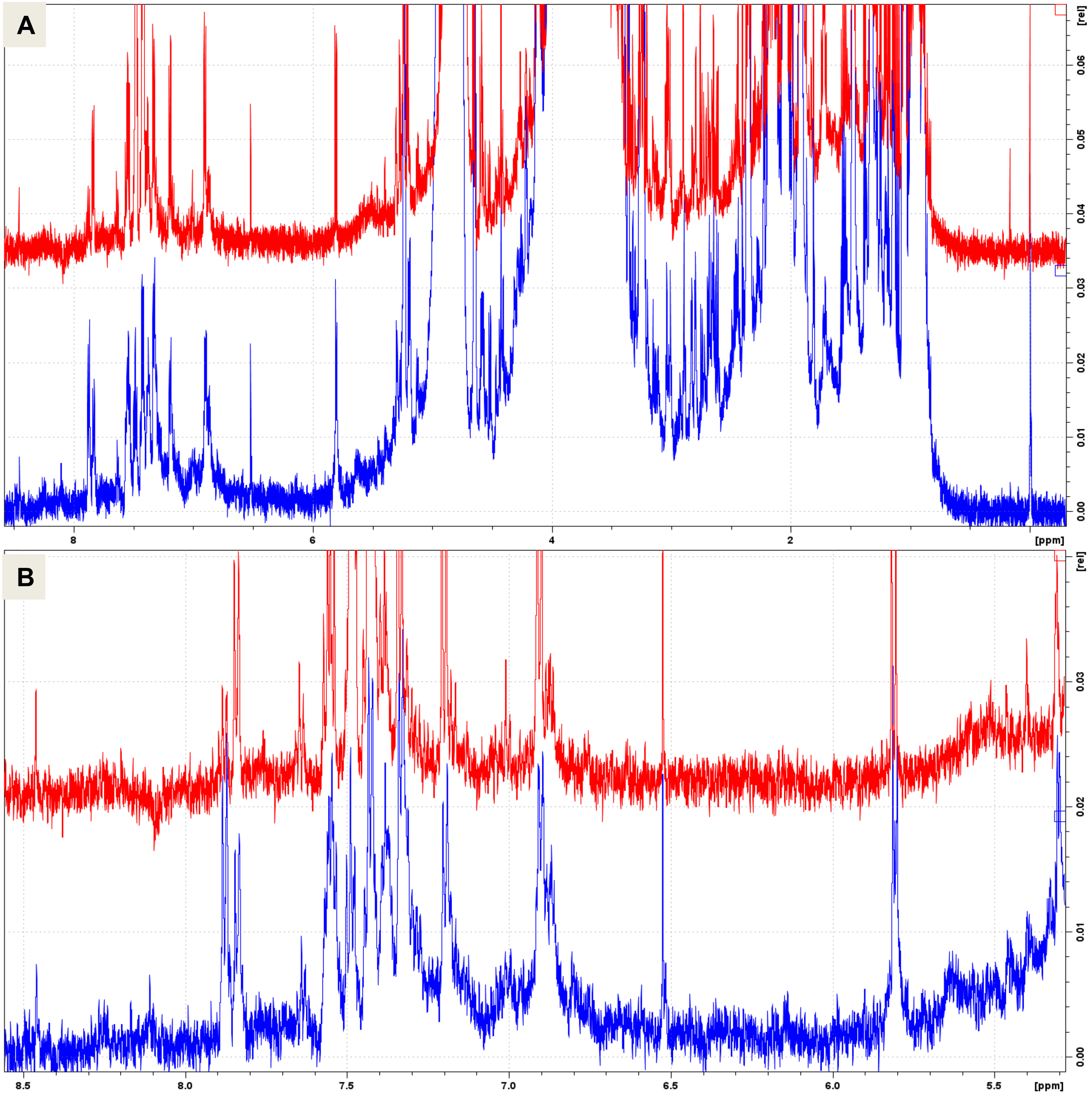

Fig BJ. Representative ^{1}H NMR CPMG Spectra of 11-Month Female Fecal Samples. (A) Image displays the control spectra in blue and the study spectra in red. (B) Zoomed in image of the control (blue) and red (study) spectra so all visual differences are easier to be seen.

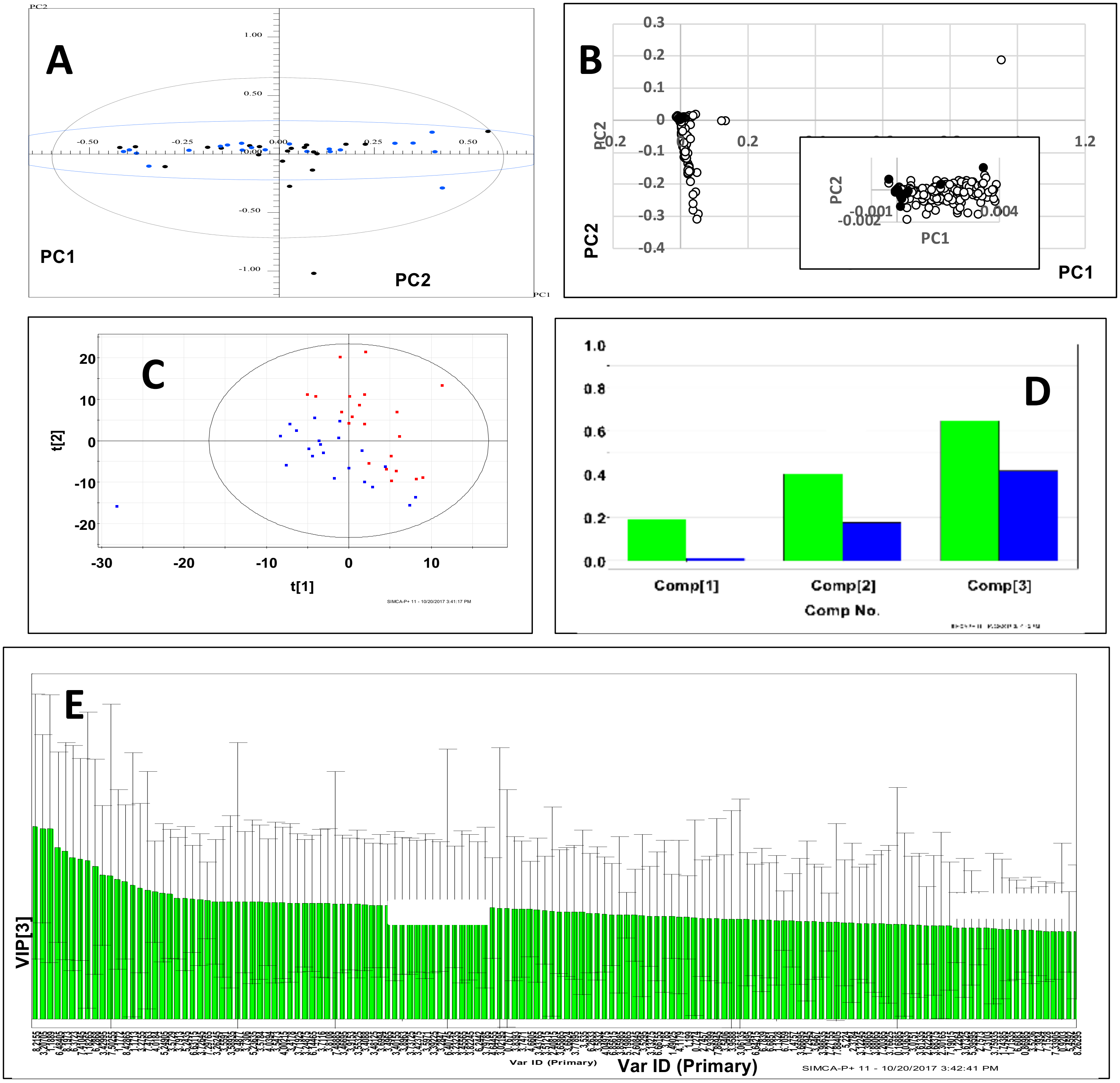

Fig BK. PCA and PLS-DA Analysis of the Female 11-Month Fecal Samples.
(A) PCA scores plot analysis showing no separation between the control and study groups. (B) PCA loadings plot displaying the color-coded buckets. Red (10-9 – 10-13), yellow (10-9 – 10-7), green (10-7 – 10-5), blue (10-5 – 1.21X10-4), black closed (<0.05), black open (>0.05). (C) PLS-DA scores plot. (D) A plot of the first three PCs displaying the R2Y explained variation and the Q2 predicted variation. (E) VIP numbers from the PLS-DA analysis that are greater than or less than 1.

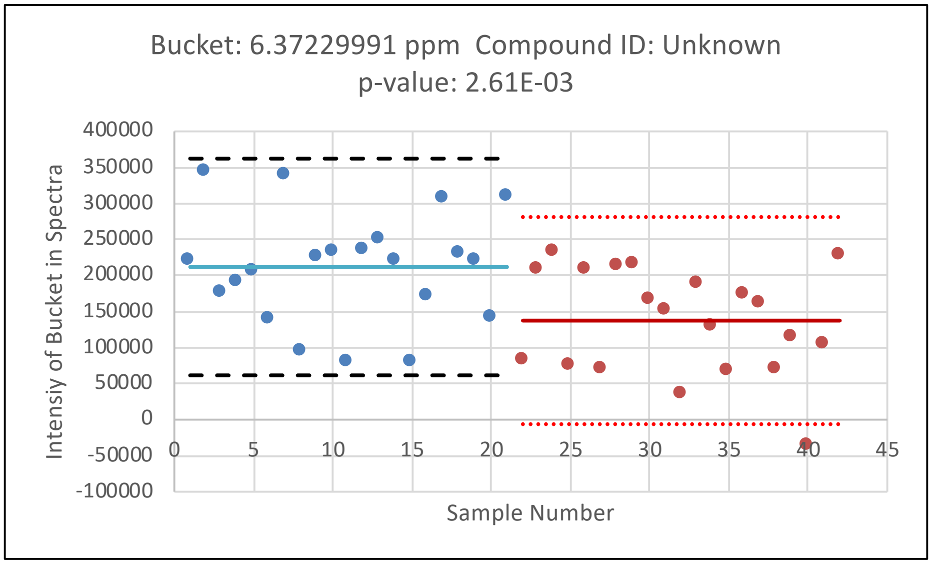

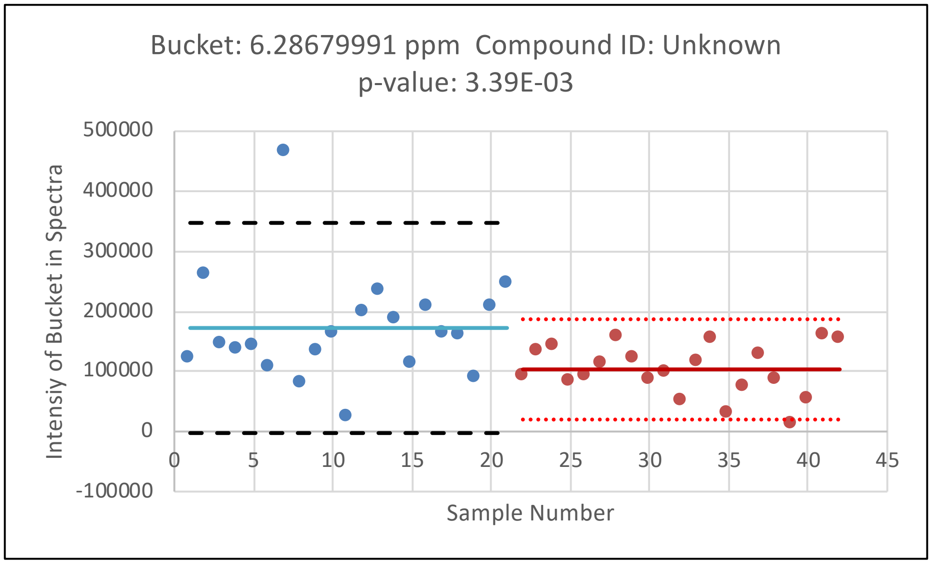

Fig BL. Intensity Plots of 11-Month Female Fecal Samples.
(A) Unidentified bucket at 6.37 ppm with a p-value of 2.61E-03. (B) Unidentified bucket at 6.29 ppm with a p-value of 3.39E-03.

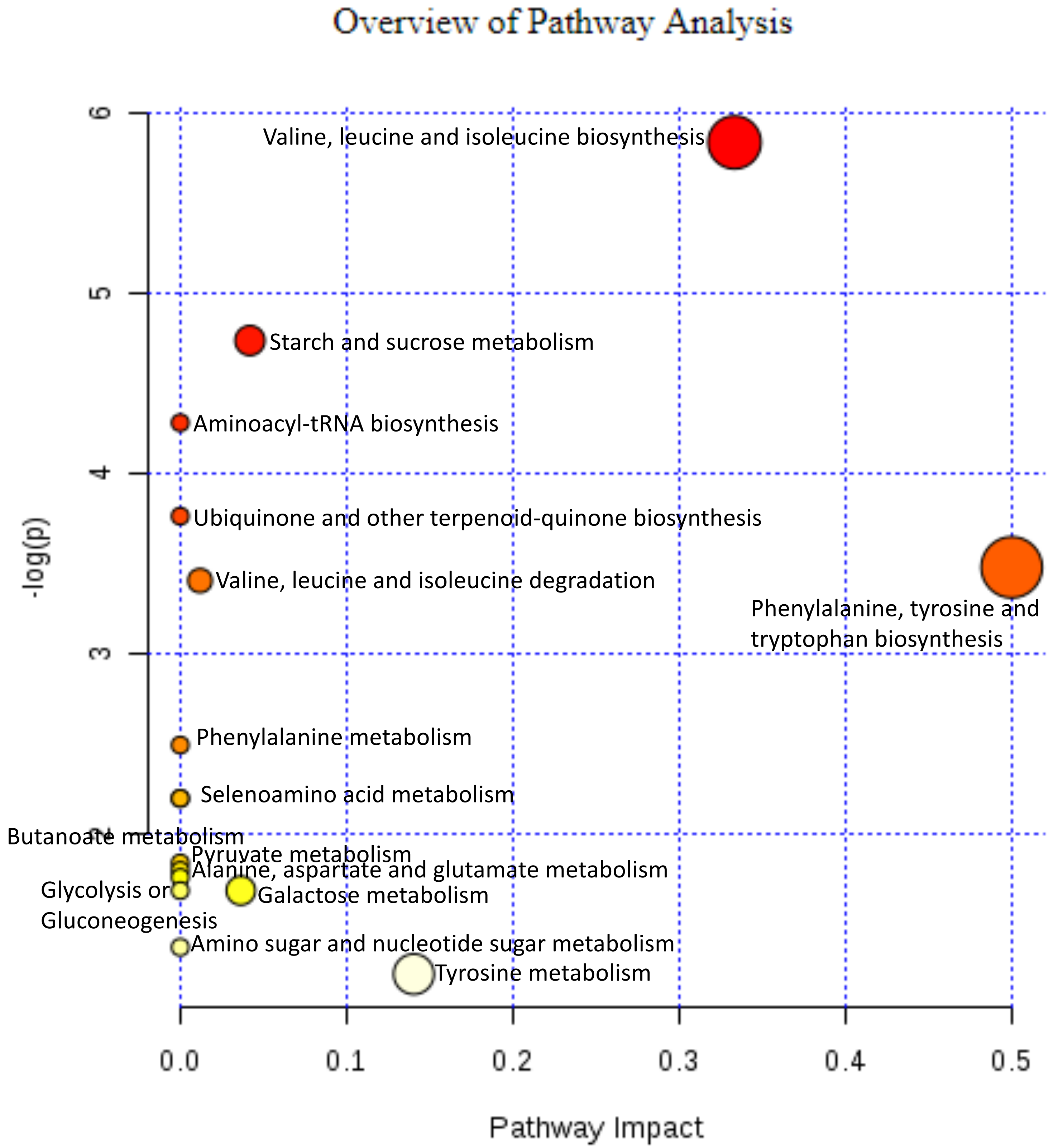

Fig BM. Pathway Analysis from the 11-Month Female Fecal Samples. The "metabolome view" from MetaboAnalyst 3.0 showing the pathway impact on the x-axis versus the negative log p values on the y-axis for the metabolic pathways. Pathway names have been added.

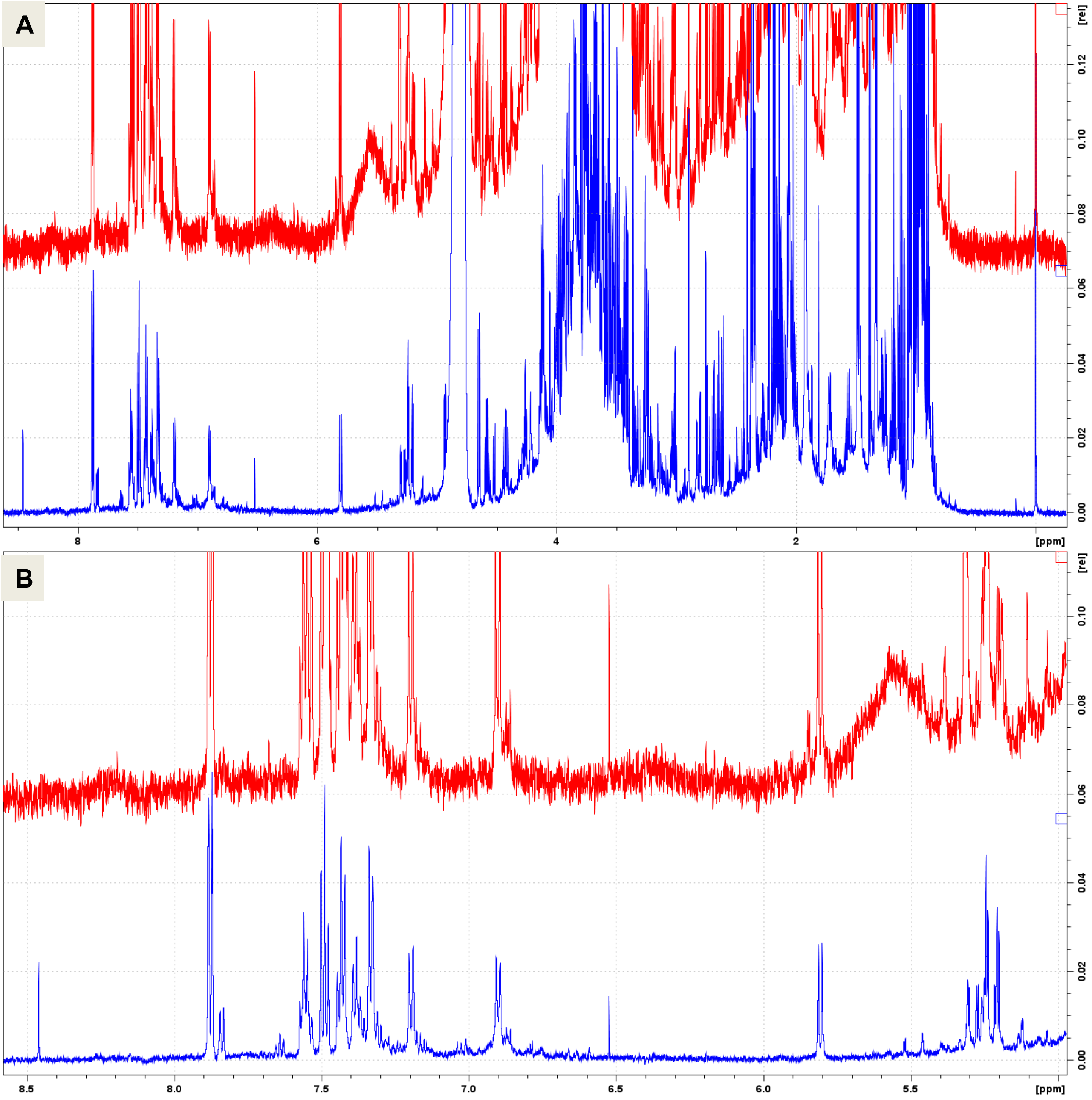

Fig BN. Representative ^{1}H NMR CPMG Spectra of 5-Month Male Fecal Samples. (A) Image displays the control spectra in blue and the study spectra in red. (B) Zoomed in image of the control (blue) and red (study) spectra so all visual differences are easier to be seen.

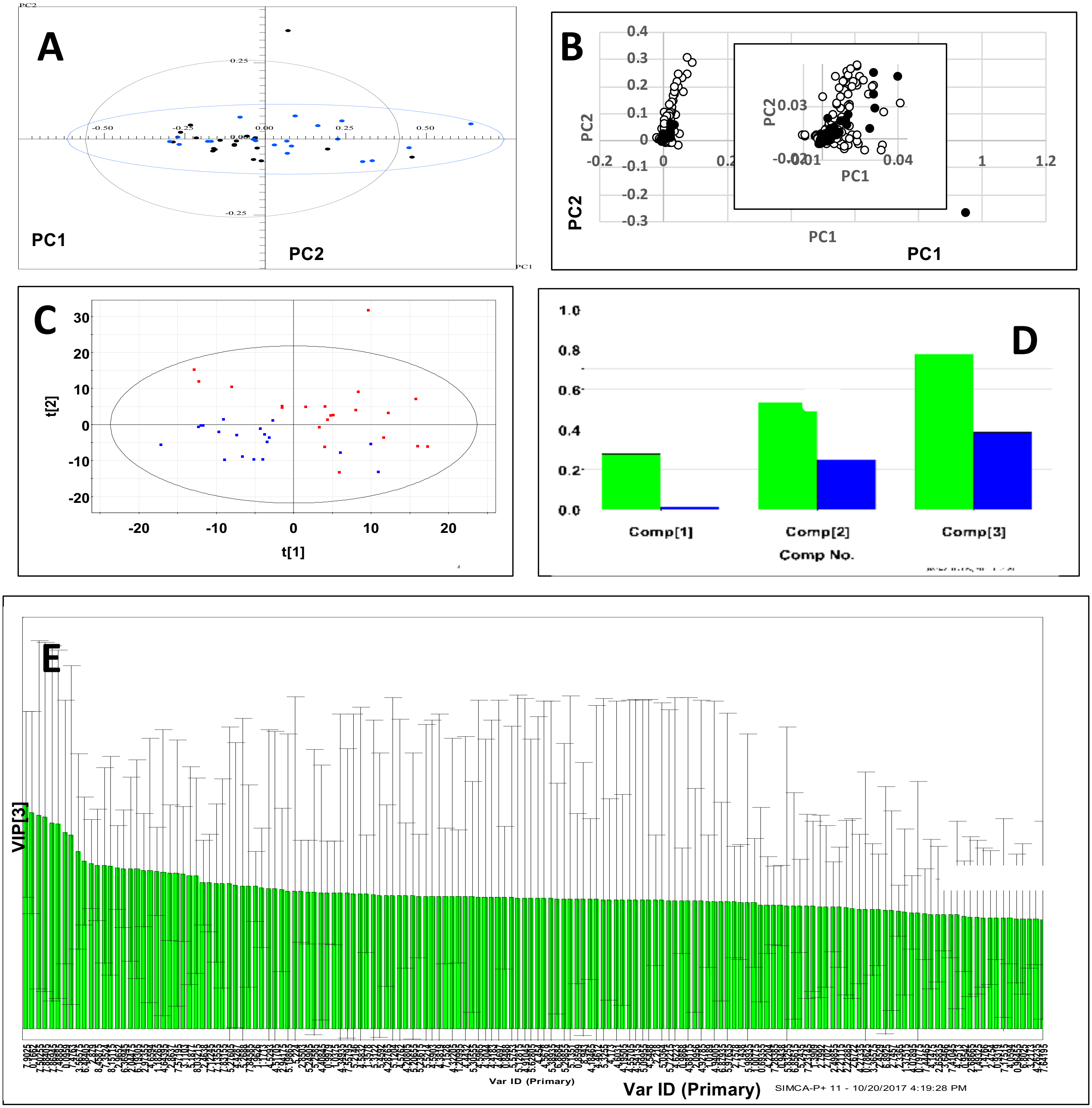

Fig BO. PCA and PLS-DA of the Male 5-Month Fecal Samples.

(A) PCA scores plot for comparison between the control and study groups. (B) PCA loadings plot displaying the color-coded buckets. Red (10-9 – 10-13), yellow (10-9 – 10-7), green (10-7 – 10-5), blue (10-5 – 1.11X10-4), black closed (<0.05), black open (>0.05). (C) PLS-DA scores plot. (D) A plot of the first three PCs displaying the R2Y explained variation and the Q2 predicted variation. (E) VIP numbers from the PLS-DA analysis that are greater than or less than 1.

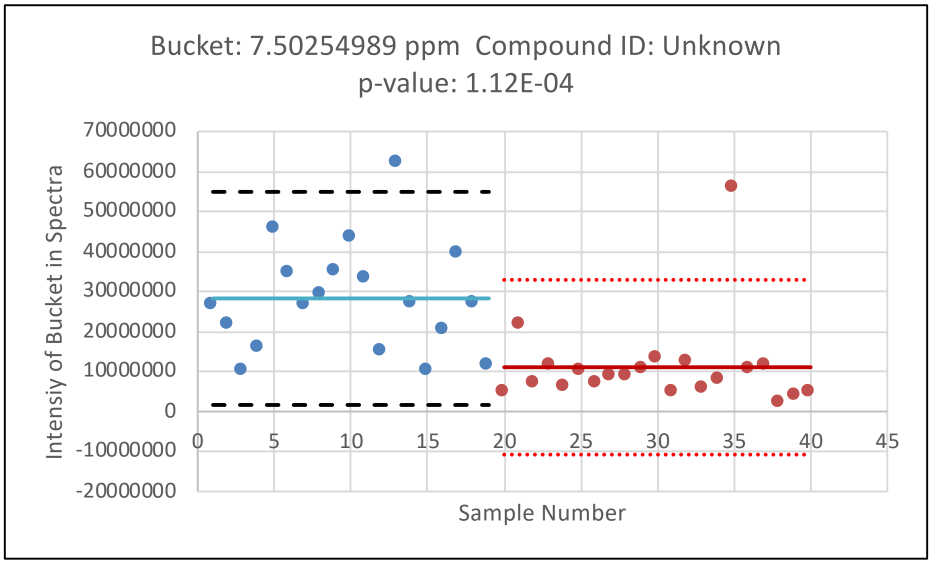

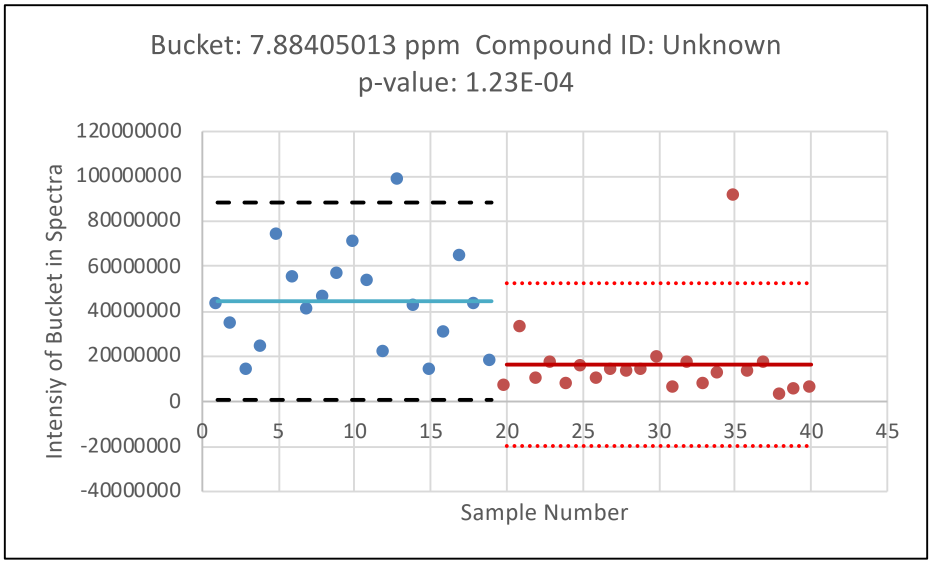

Fig BP. Intensity Plots of 5-Month Male Fecal Samples.
(A) Unidentified bucket at 7.50 ppm with a p-value of 1.12E-04. (B) Unidentified bucket at 7.88 ppm with a p-value of 1.23E-04.

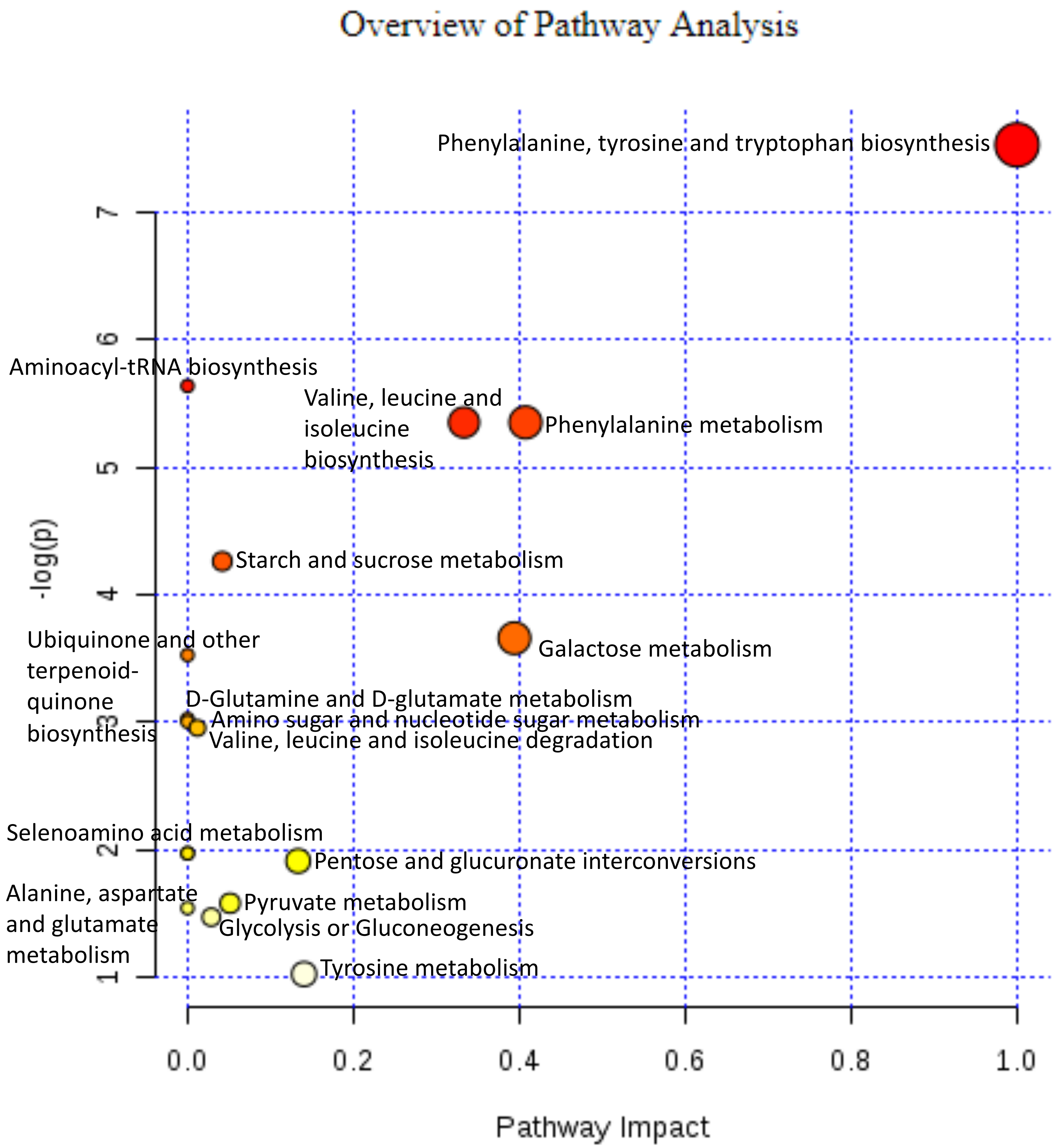

Fig BQ. Pathway Analysis from the 5-Month Male Fecal Samples. The “metabolome view” from MetaboAnalyst 3.0 showing the pathway impact on the x-axis versus the negative log p values on the y-axis for the metabolic pathways. Pathway names have been added.

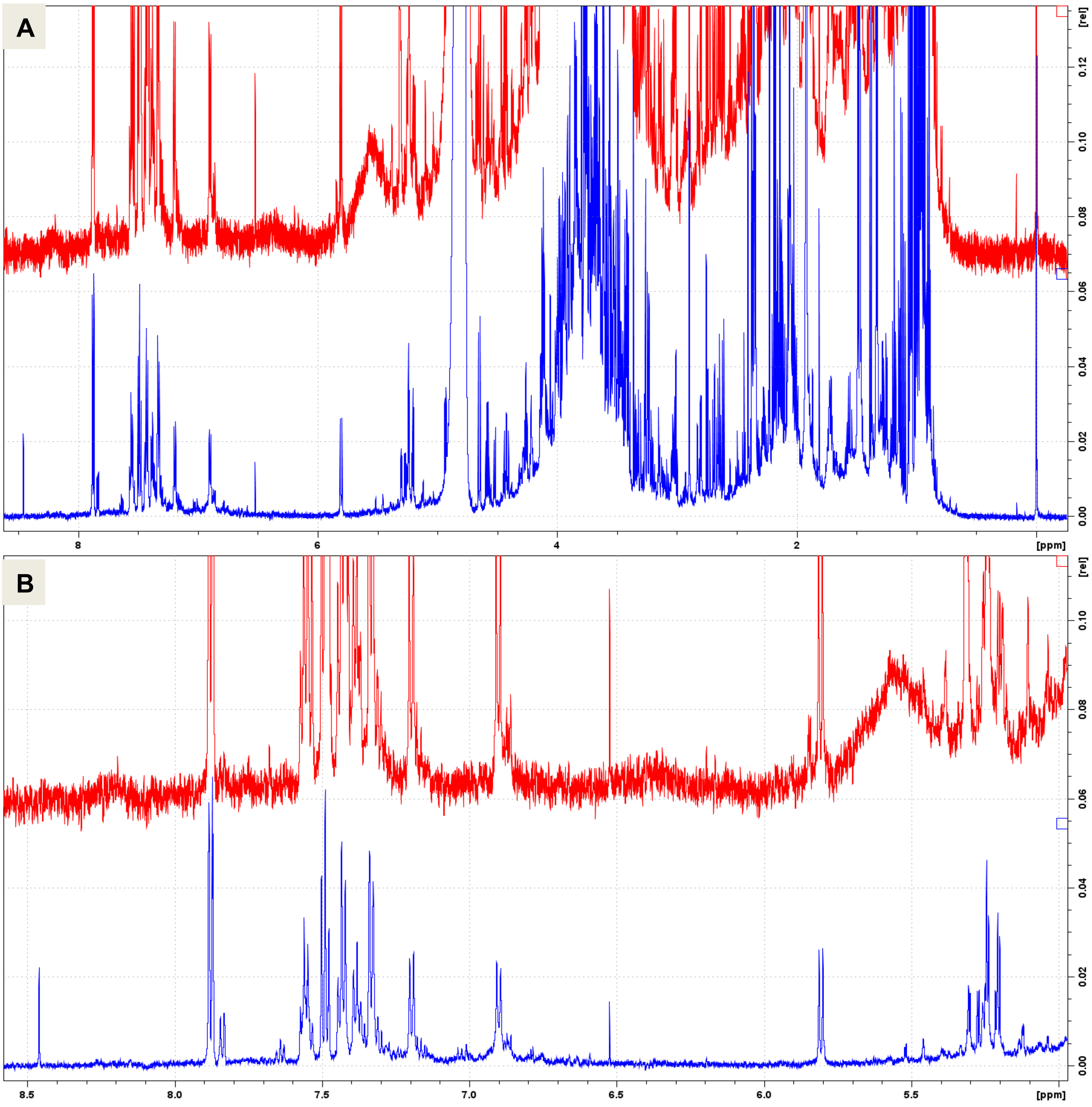

Fig BR. Representative ^{1}H NMR CPMG spectra of 11-month Male Fecal Samples. (A) Image displays the control spectra in blue and the study spectra in red. (B) Zoomed in image of the control (blue) and red (study) spectra so all visual differences are easier to be seen.

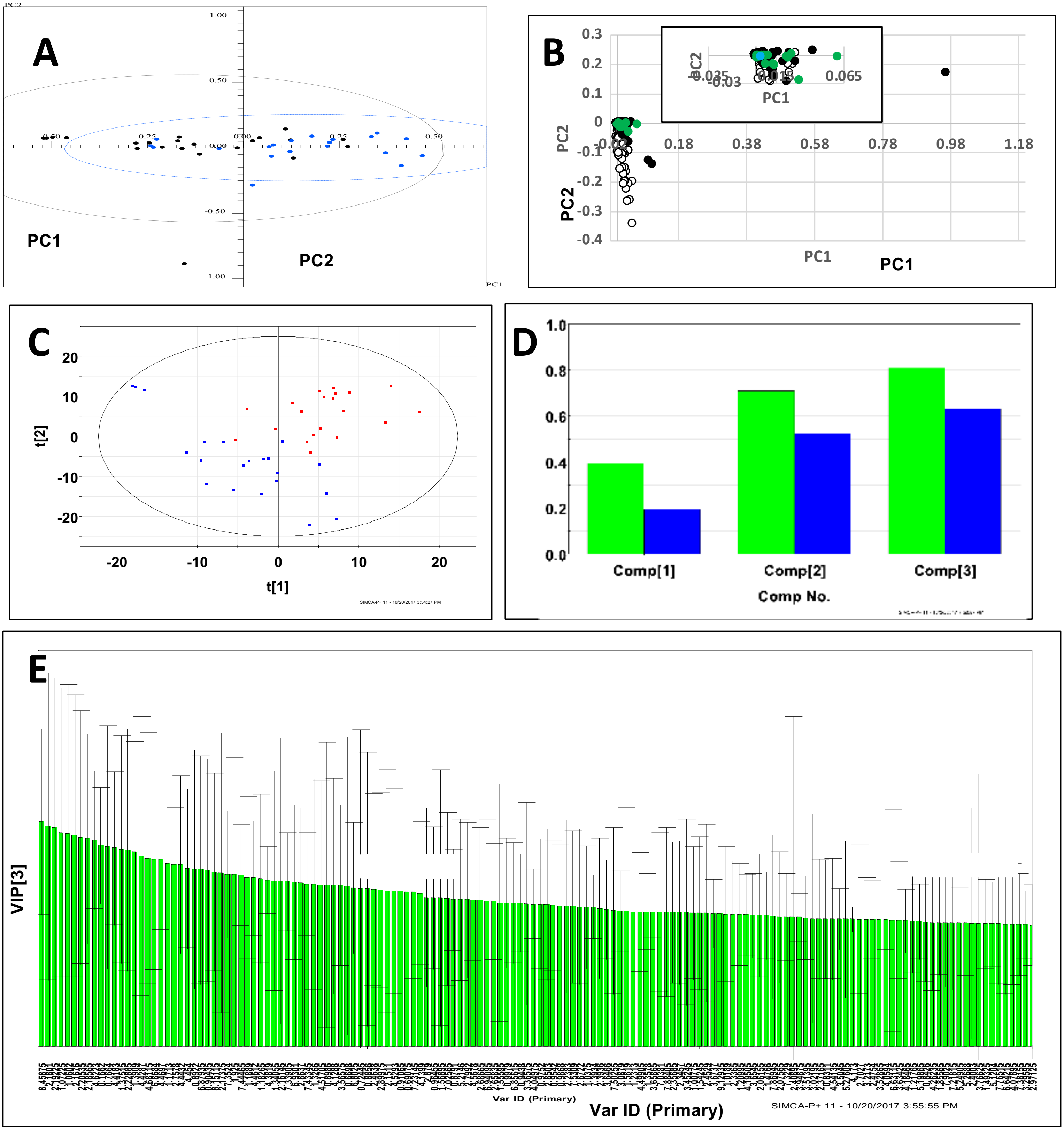

Fig BS. PCA and PLS-DA of Male 11-month Fecal Samples. (A) PCA scores plot for comparison between the control and study groups. (B) PCA loadings plot displaying the color-coded buckets. Red (10-9 – 10-13), yellow (10-9 – 10-7), green (10-7 – 10-5), blue (10-5 – 1.13X10-4), black closed (<0.05), black open (>0.05). (C) PLS-DA scores plot. (D) A plot of the first three PCs displaying the R2Y explained variation and the Q2 predicted variation. (E) VIP numbers from the PLS-DA analysis that are greater than or less than 1.

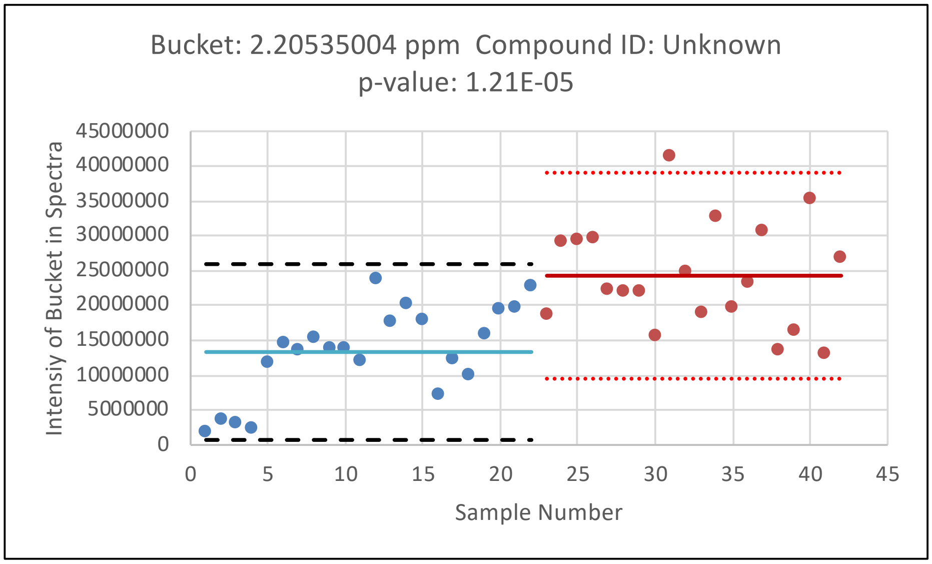

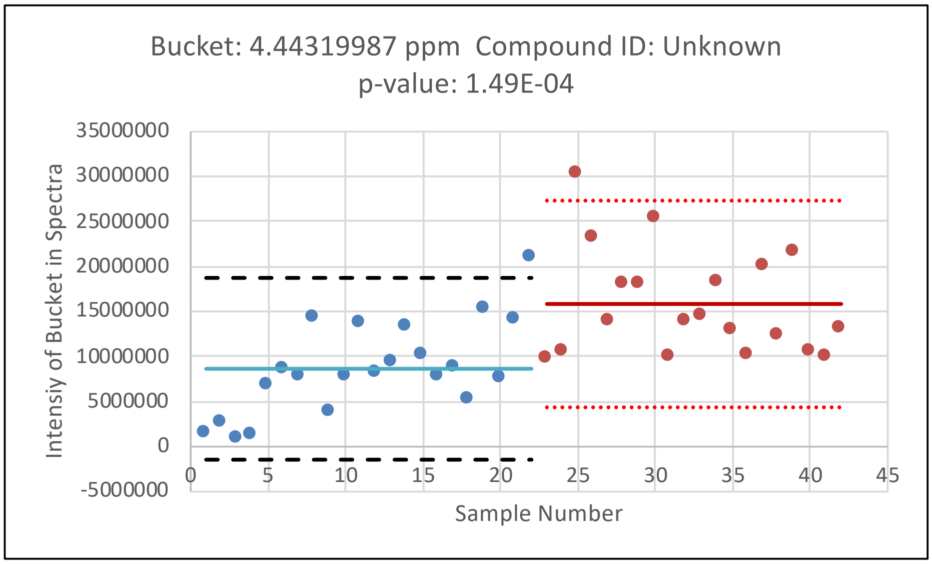

Fig BT. Intensity Plots of 11-Month Male Fecal Samples. (A) Unidentified bucket at 2.20ppm with a p-value of 1.21E-05. (B) Unidentified bucket at 4.44 ppm with a p-value of 1.49E-04.

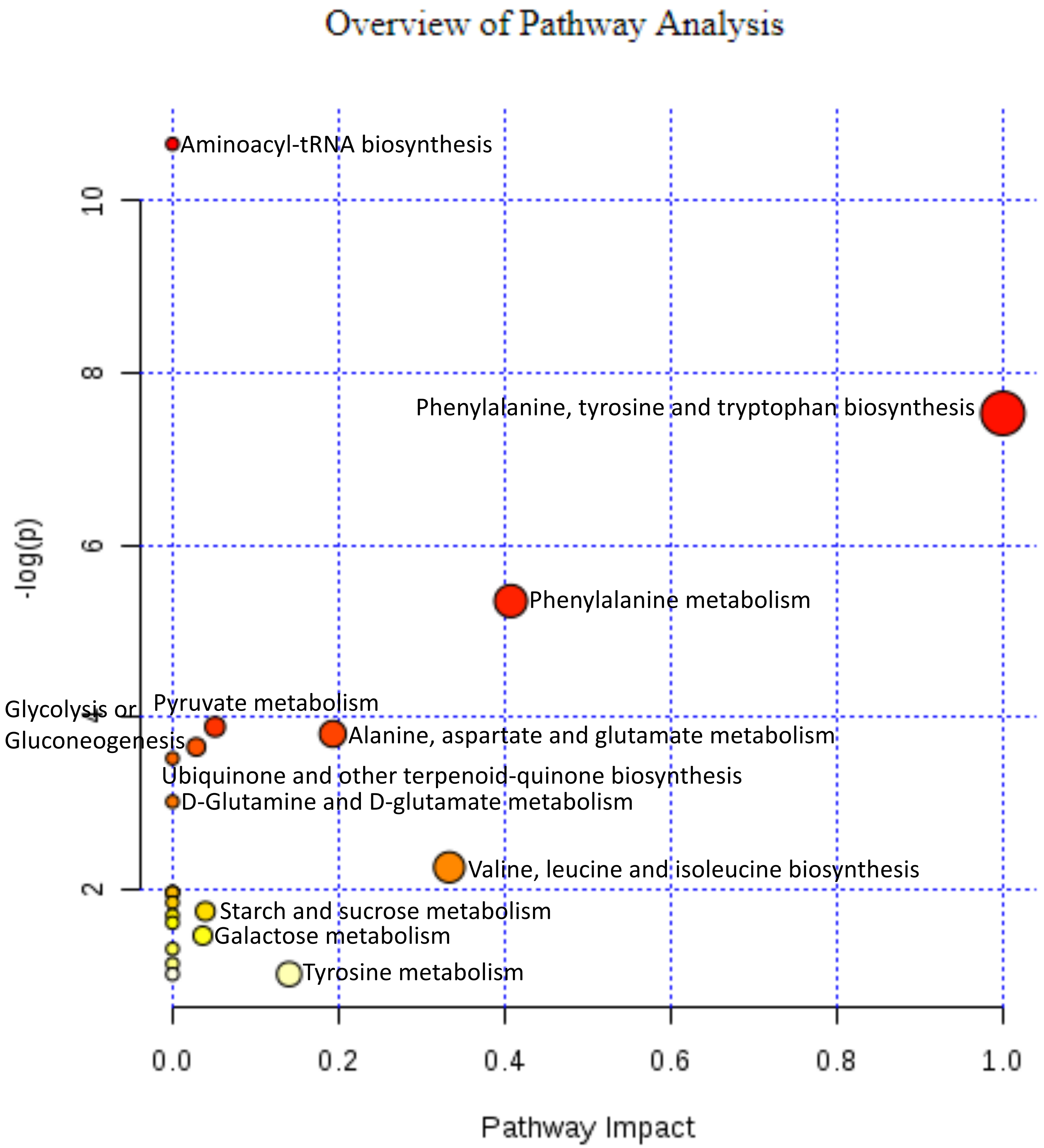

Fig BU. Pathway Analysis from the 11-Month Male Fecal Samples.
The "metabolome view" from MetaboAnalyst 3.0 showing the pathway impact on the x-axis versus the negative log p values on the y-axis for the metabolic pathways. Pathway names have been added.
