# Supplementary material for: NMR-based metabolic profiling of urine, serum, fecal, and pancreatic tissue samples from the Ptf1a-Cre; LSL-KrasG12D transgenic mouse model of pancreatic cancer
Source: PLoS One. 2018 Jul 17;13(7):e0200658. doi: 10.1371/journal.pone.0200658 (PMC6049928; doi:10.1371/journal.pone.0200658)
Supplement: S2 File — (PDF) [file pone.0200658.s002.pdf]

Table A: List of unidentified buckets with p-value < 0.05 and fold change < 2 for 15-month old male urine samples (colored red in the volcano plot).

ppm	VIP	p-value	**Fold Change (Error)**	**AUC**
1.778	1.40	1.31E-05	-1.82 (0.51)	0.90
6.933	1.44	1.39E-05	-1.86 (0.44)	0.89
7.312	1.46	2.97E-05	-1.75 (0.31)	0.89
7.325	1.42	4.82E-05	-1.89 (0.25)	0.90
7.297	1.27	6.61E-05	-1.82 (0.32)	0.88
5.973	1.15	1.00E-04	-1.74 (0.60)	0.82
1.765	1.24	1.01E-04	-1.80 (0.57)	0.86
5.984	1.16	1.12E-04	-1.69 (0.65)	0.82
7.070	1.16	1.35E-04	-1.81 (0.56)	0.84
7.099	1.16	1.37E-04	-1.94 (0.45)	0.88
3.074	1.37	2.03E-04	-1.53 (0.41)	0.82
1.788	1.09	2.77E-04	-1.62 (0.48)	0.81
2.201	1.11	3.90E-04	-1.58 (0.41)	0.81
6.689	1.11	4.29E-04	-1.86 (0.43)	0.82
7.124	1.11	5.07E-04	-1.99 (0.26)	0.88
7.035	1.07	5.14E-04	-1.52 (0.42)	0.80
6.388	1.09	6.24E-04	-1.98 (0.47)	0.80
2.853	1.09	6.46E-04	-1.47 (0.34)	0.79
6.065	1.06	6.62E-04	-1.93 (0.56)	0.82
7.248	1.07	6.72E-04	-1.72 (0.38)	0.83
7.111	1.07	6.81E-04	-1.81 (0.64)	0.86
2.214	1.06	6.99E-04	-1.58 (0.33)	0.80
5.862	1.02	7.43E-04	-1.41 (0.42)	0.78
6.960	1.18	7.54E-04	-1.76 (0.39)	0.81
0.946	1.11	7.87E-04	-1.66 (0.51)	0.80
6.644	1.05	8.56E-04	-1.66 (0.32)	0.81
0.935	1.09	8.70E-04	-1.63 (0.45)	0.80
1.575	1.04	9.25E-04	-1.56 (0.37)	0.80
6.657	1.04	9.26E-04	-1.82 (0.39)	0.80
6.205	1.25	9.57E-04	-1.50 (0.58)	0.78
1.591	1.07	9.60E-04	-1.82 (0.31)	0.82
7.392	1.01	9.66E-04	-1.57 (0.44)	0.79
5.851	1.03	9.95E-04	-1.44 (0.41)	0.79
2.805	1.11	1.04E-03	-1.62 (0.47)	0.82
7.365	1.08	1.05E-03	-1.71 (0.57)	0.83

3.085	1.31	1.06E-03	-1.48 (0.30)	0.80
6.803	1.04	1.07E-03	-1.87 (0.32)	0.84
2.313	1.04	1.12E-03	-1.54 (0.34)	0.79
7.174	1.03	1.19E-03	-1.88 (0.39)	0.84
2.490	1.02	1.23E-03	-1.58 (0.34)	0.79
2.043	1.01	1.24E-03	-1.55 (0.36)	0.79
6.859	1.03	1.29E-03	-1.64 (0.31)	0.79
0.870	1.17	1.39E-03	-1.90 (0.25)	0.84
1.318	1.24	1.40E-03	-1.65 (0.31)	0.82
2.323	1.01	1.46E-03	-1.50 (0.36)	0.77
2.335	0.998	1.54E-03	-1.47 (0.38)	0.78
1.129	1.01	1.57E-03	-1.64 (0.37)	0.79
1.852	1.01	1.65E-03	-1.51 (0.32)	0.77
2.794	1.02	1.74E-03	-1.50 (0.51)	0.79
6.906	0.98	1.78E-03	-1.64 (0.38)	0.79
2.783	1.02	1.84E-03	-1.46 (0.33)	0.79
7.674	0.98	1.87E-03	-1.42 (0.42)	0.75
3.098	0.99	1.89E-03	-1.45 (0.34)	0.77
2.965	1.03	1.90E-03	-1.50 (0.47)	0.80
2.097	0.99	1.95E-03	-1.46 (0.40)	0.77
3.043	0.97	1.98E-03	-1.90 (0.75)	0.89
2.814	0.9998	2.06E-03	-1.46 (0.33)	0.75
0.890	1.14	2.08E-03	-1.86 (0.30)	0.81
1.108	1.05	2.15E-03	-1.57 (0.48)	0.78
7.220	0.95	2.21E-03	-1.56 (0.57)	0.78
2.065	1.03	2.26E-03	-1.43 (0.43)	0.76
2.764	0.99	2.31E-03	-1.41 (0.35)	0.75
1.615	0.996	2.41E-03	-1.75 (0.27)	0.81
6.211	1.38	2.43E-03	-1.52 (0.41)	0.78
6.893	0.96	2.45E-03	-1.69 (0.39)	0.79
2.624	0.96	2.53E-03	-1.65 (0.42)	0.78
6.673	0.97	2.57E-03	-1.78 (0.46)	0.77
3.165	0.999	2.71E-03	-1.57 (0.51)	0.76
1.988	1.01	2.76E-03	-1.49 (0.35)	0.76
8.257	0.95	2.77E-03	-1.62 (0.50)	0.77
6.759	0.95	2.79E-03	-1.68 (0.50)	0.76
2.005	1.01	2.86E-03	-1.52 (0.36)	0.78
2.611	0.92	2.95E-03	-1.44 (0.54)	0.75
1.839	0.98	2.99E-03	-1.41 (0.39)	0.75

2.828	0.97	3.12E-03	-1.39 (0.38)	0.75
2.935	1.07	3.18E-03	-1.61 (0.45)	0.74
8.741	0.94	3.25E-03	-1.85 (0.43)	0.76
2.283	0.95	3.30E-03	-1.63 (0.30)	0.78
2.843	0.95	3.55E-03	-1.38 (0.35)	0.74
6.200	0.95	3.61E-03	-1.87 (0.48)	0.82
6.560	0.95	3.61E-03	-1.99 (0.51)	0.81
2.790	0.97	3.72E-03	-1.43 (0.38)	0.74
2.265	0.95	3.93E-03	-1.51 (0.38)	0.75
3.135	0.95	4.07E-03	-1.51 (0.38)	0.75
2.496	0.97	4.14E-03	-1.50 (0.48)	0.76
0.860	1.01	4.20E-03	-1.52 (0.34)	0.76
6.967	1.14	4.25E-03	-1.52 (0.32)	0.76
2.506	0.96	4.27E-03	-1.46 (0.58)	0.74
5.778	1.0002	4.62E-03	-1.44 (0.49)	0.75
6.701	0.96	4.98E-03	-1.89 (0.41)	0.76
2.087	0.95	5.07E-03	-1.44 (0.47)	0.76
1.408	0.95	5.17E-03	-1.37 (0.42)	0.74
1.306	1.10	5.31E-03	-1.72 (0.33)	0.79
2.799	0.95	5.31E-03	-1.34 (0.42)	0.74
1.733	1.03	5.41E-03	-1.42 (0.82)	0.79
2.298	0.90	5.42E-03	-1.67 (0.35)	0.78
7.762	0.91	5.97E-03	-1.60 (0.31)	0.78
2.054	0.95	6.07E-03	-1.36 (0.57)	0.77
2.487	0.91	6.07E-03	-1.70 (0.30)	0.78
1.096	0.90	6.29E-03	-1.43 (0.47)	0.74
1.114	0.94	6.29E-03	-1.39 (0.38)	0.72
1.692	0.94	6.29E-03	-1.39 (0.43)	0.73
1.121	0.93	6.35E-03	-1.50 (0.53)	0.76
1.704	0.94	6.40E-03	-1.36 (0.51)	0.75
6.976	0.87	6.53E-03	-1.53 (0.54)	0.74
2.478	1.08	6.55E-03	-1.56 (0.46)	0.75
7.750	0.87	6.59E-03	-1.48 (0.45)	0.75
6.551	1.10	6.68E-03	-1.81 (0.98)	0.77
2.222	1.004	6.77E-03	-1.37 (0.42)	0.73
2.774	0.95	6.86E-03	-1.42 (0.36)	0.74
1.508	0.95	6.87E-03	-1.43 (0.57)	0.75
2.252	0.98	6.94E-03	-1.41 (0.41)	0.75
1.713	0.94	7.11E-03	-1.37 (0.54)	0.75

1.520	0.93	7.13E-03	-1.43 (0.61)	0.75
2.484	1.03	7.16E-03	-1.53 (0.62)	0.75
1.211	1.09	7.20E-03	-1.47 (0.59)	0.73
6.990	0.85	7.31E-03	-1.44 (0.37)	0.73
0.920	1.11	7.45E-03	-1.40 (0.61)	0.77
6.569	0.93	7.57E-03	-1.56 (0.58)	0.74
0.916	1.01	7.59E-03	-1.56 (0.36)	0.77
1.971	0.96	7.91E-03	-1.34 (0.39)	0.71
1.295	1.01	8.08E-03	-1.58 (0.35)	0.78
6.190	0.83	8.19E-03	-1.56 (1.45)	0.79
0.911	1.01	8.43E-03	-1.51 (0.54)	0.78
2.393	0.94	8.53E-03	-1.36 (0.41)	0.71
2.080	0.92	8.74E-03	-1.37 (0.48)	0.75
7.739	0.86	8.82E-03	-1.72 (0.30)	0.77
8.345	0.86	8.82E-03	-1.57 (0.53)	0.75
1.875	0.87	8.83E-03	-1.76 (0.25)	0.78
1.665	0.91	8.87E-03	-1.43 (0.39)	0.73
1.580	0.90	8.93E-03	-1.54 (0.58)	0.77
2.471	0.87	9.21E-03	-1.80 (0.25)	0.82
0.894	1.01	9.42E-03	-1.46 (0.45)	0.77
0.682	1.36	9.61E-03	1.63 (1.77)	0.70
4.476	0.94	9.67E-03	-1.39 (0.50)	0.73
2.957	1.02	9.70E-03	-1.42 (0.36)	0.72
8.325	0.84	9.86E-03	-1.97 (0.45)	0.74
6.621	1.11	1.01E-02	-1.57 (0.77)	0.77
0.926	0.88	1.04E-02	-1.78 (0.30)	0.79
1.561	0.91	1.06E-02	-1.34 (0.48)	0.75
1.195	0.91	1.09E-02	-1.31 (0.42)	0.72
2.755	0.98	1.10E-02	-1.37 (0.39)	0.72
2.170	0.94	1.14E-02	-1.42 (0.77)	0.76
0.905	1.03	1.20E-02	-1.39 (0.54)	0.77
6.592	0.83	1.21E-02	-1.86 (0.82)	0.74
2.001	0.94	1.22E-02	-1.36 (0.38)	0.72
2.074	0.87	1.24E-02	-1.42 (0.60)	0.74
2.107	0.91	1.29E-02	-1.34 (0.49)	0.73
5.901	0.46	0.01290807	-1.42 (0.47)	0.76
1.944	0.93	1.31E-02	-1.35 (0.38)	0.71
1.513	0.94	1.33E-02	-1.39 (0.63)	0.74
1.727	0.89	1.34E-02	-1.39 (0.70)	0.76

2.924	0.88	1.35E-02	-1.40 (0.47)	0.71
6.875	0.80	1.35E-02	-1.48 (0.45)	0.70
2.025	0.94	1.42E-02	-1.35 (0.42)	0.71
2.950	0.84	1.42E-02	-1.37 (0.65)	0.72
7.053	1.06	1.42E-02	-1.47 (0.52)	0.73
1.436	0.95	1.44E-02	-1.35 (0.53)	0.72
7.379	0.88	1.45E-02	-1.34 (0.51)	0.75
1.274	0.89	1.51E-02	-1.36 (0.44)	0.72
1.502	0.93	1.57E-02	-1.37 (0.66)	0.74
1.133	0.84	1.58E-02	-1.43 (0.59)	0.75
2.012	1.06	1.58E-02	-1.32 (0.35)	0.71
1.628	0.88	1.64E-02	-1.42 (0.37)	0.72
0.720	1.38	1.65E-02	1.78 (4.92)	0.67
1.498	0.90	1.84E-02	-1.45 (0.63)	0.74
1.102	0.90	1.95E-02	-1.34 (0.38)	0.68
2.918	0.95	2.01E-02	-1.32 (0.34)	0.69
2.409	0.77	2.02E-02	-1.52 (0.71)	0.71
7.232	0.88	2.04E-02	-1.43 (0.47)	0.69
0.885	0.92	2.10E-02	-1.43 (0.59)	0.76
4.565	0.77	2.31E-02	-1.35 (0.60)	0.69
1.676	0.98	2.42E-02	-1.34 (0.43)	0.70
7.729	0.75	2.42E-02	-1.47 (0.89)	0.78
8.900	0.79	2.42E-02	-1.89 (0.54)	0.70
5.889	1.03	0.02541548	-1.37 (0.65)	0.76
7.421	0.69	2.75E-02	-1.37 (0.69)	0.77
1.886	0.76	2.79E-02	-1.75 (0.25)	0.76
4.457	0.88	3.04E-02	-1.27 (0.43)	0.69
4.339	1.06	3.06E-02	-1.39 (0.46)	0.69
8.123	0.82	3.08E-02	-1.45 (0.44)	0.69
8.109	0.86	3.12E-02	-1.43 (0.48)	0.68
2.730	0.90	3.17E-02	-1.31 (0.39)	0.69
2.895	0.78	3.19E-02	-1.55 (0.78)	0.70
1.206	0.82	3.23E-02	-1.27 (0.50)	0.67
1.228	0.84	3.25E-02	-1.25 (0.52)	0.69
1.449	0.90	3.26E-02	-1.29 (0.54)	0.70
1.239	0.77	3.28E-02	-1.48 (0.31)	0.72
8.940	0.85	3.30E-02	-1.90 (1.12)	0.74
3.030	0.76	3.44E-02	-1.81 (0.38)	0.73
5.835	1.20	3.66E-02	1.80 (3.73)	0.59

1.864	0.97	3.72E-02	-1.32 (0.59)	0.71
6.484	0.73	3.75E-02	-1.60 (0.43)	0.68
1.428	0.85	3.77E-02	-1.24 (0.57)	0.70
1.485	0.85	3.89E-02	-1.31 (0.67)	0.74
5.488	1.13	4.07E-02	1.59 (3.36)	0.60
1.456	0.91	4.15E-02	-1.27 (0.56)	0.68
1.385	0.82	4.16E-02	-1.27 (0.44)	0.66
2.208	0.85	4.49E-02	-1.35 (0.71)	0.72
1.222	0.81	4.52E-02	-1.30 (0.61)	0.69
4.417	0.996	4.57E-02	-1.64 (0.37)	0.73
4.317	0.81	4.62E-02	-1.24 (0.60)	0.65
2.240	1.16	4.84E-02	-1.29 (0.50)	0.67
4.281	0.88	4.93E-02	-1.29 (0.58)	0.65

Table B: List of unidentified buckets with p-value < 0.05 and fold change < 2 for 15-month old female urine samples (colored red in the volcano plot).

**ppm**	**VIP**	**p-value**	**Fold Change (Error)**	AUC
1.7783	0.782692	1.31E-05	-1.82 (0.60)	0.65646
6.9332	1.2043	1.39E-05	-1.86 (0.60)	0.51134
7.31195	1.09563	2.97E-05	-1.75 (0.54)	0.50907
7.3253	1.38167	4.82E-05	-1.89 (0.50)	0.64286
7.29695	1.34753	6.61E-05	-1.82 (0.46)	0.62925
5.973	0.808922	1.00E-04	-1.74 (0.54)	0.55669
1.765	0.790484	1.01E-04	-1.80 (0.58)	0.53741
5.9838	0.775959	1.12E-04	-1.69 (0.63)	0.52041
7.0703	0.937385	1.35E-04	-1.81 (0.74)	0.68141
7.09905	0.464333	1.37E-04	-1.94 (1.62)	0.61905
7.516	2.29	1.92E-04	-1.62 (0.31)	0.61451
3.0741	1.20848	2.03E-04	-1.53 (1.01)	0.54082
1.7884	0.590373	2.77E-04	-1.62 (0.70)	0.78231
7.6556	2.23005	3.32E-04	1.25 (0.32)	0.57937
2.20145	0.800558	3.90E-04	-1.58 (0.54)	0.52381
6.6887	0.921674	4.29E-04	-1.86 (1.00)	0.54535
7.1239	0.867181	5.07E-04	-1.99 (0.69)	0.62472
7.0352	0.74087	5.14E-04	-1.52 (0.54)	0.64286
6.3875	1.07389	6.24E-04	-1.98 (0.77)	0.68707
2.8533	0.875667	6.46E-04	-1.47 (0.51)	0.56689
6.06505	0.562056	6.62E-04	-1.93 (0.49)	0.51814
7.24825	0.918218	6.72E-04	-1.72 (0.74)	0.54649
7.11075	1.10874	6.81E-04	-1.81 (0.43)	0.59864
2.2135	0.797945	6.99E-04	-1.58 (0.50)	0.64172
5.86165	0.735168	7.43E-04	-1.41 (0.33)	0.53288
6.9599	0.935871	7.54E-04	-1.76 (0.38)	0.71429
7.56945	2.11982	7.71E-04	1.25 (0.33)	0.59637
0.9459	0.957511	7.87E-04	-1.66 (0.57)	0.60431
6.6442	0.650551	8.56E-04	-1.66 (0.58)	0.57029

0.93455	0.957718	8.70E-04	-1.63 (0.57)	0.61678
1.5749	0.798385	9.25E-04	-1.56 (0.45)	0.5805
6.65675	0.862213	9.26E-04	-1.82 (0.96)	0.57937
6.20515	1.15239	9.57E-04	-1.50 (0.42)	0.74036
1.5914	0.661481	9.60E-04	-1.82 (0.59)	0.68367
7.39215	0.815754	9.66E-04	-1.57 (0.51)	0.59297
5.8508	1.26566	9.95E-04	-1.44 (0.29)	0.50794
2.8051	0.873405	1.04E-03	-1.62 (0.68)	0.62812
7.36495	1.25938	1.05E-03	-1.71 (0.62)	0.61111
3.0853	1.28067	1.06E-03	-1.48 (1.63)	0.56236
6.80345	1.1597	1.07E-03	-1.87 (0.70)	0.54989
2.3132	0.786319	1.12E-03	-1.54 (0.54)	0.50113
7.1739	0.905625	1.19E-03	-1.88 (0.61)	0.6644
2.49045	0.858811	1.23E-03	-1.58 (0.60)	0.50113
2.04305	0.795887	1.24E-03	-1.55 (0.66)	0.51814
6.8591	0.777213	1.29E-03	-1.64 (0.73)	0.5102
0.86985	0.704403	1.39E-03	-1.90 (0.48)	0.65986
1.3175	0.809887	1.40E-03	-1.65 (0.36)	0.62585
2.3229	0.784356	1.46E-03	-1.50 (0.48)	0.50227
2.33465	0.82673	1.54E-03	-1.47 (0.48)	0.61338
1.1285	1.26686	1.57E-03	-1.64 (0.80)	0.6712
1.85245	0.876392	1.65E-03	-1.51 (0.53)	0.56803
2.7939	0.800803	1.74E-03	-1.50 (0.59)	0.52494
6.90615	0.993258	1.78E-03	-1.64 (0.54)	0.59751
2.7834	0.798224	1.84E-03	-1.46 (0.54)	0.61111
7.67385	0.745998	1.87E-03	-1.42 (0.54)	0.5941
3.0981	1.07177	1.89E-03	-1.45 (0.60)	0.53061
2.96455	0.792239	1.90E-03	-1.50 (0.64)	0.50227
2.0973	0.791354	1.95E-03	-1.46 (0.48)	0.52154
3.0433	1.52581	1.98E-03	-1.90 (0.44)	0.52948
2.81385	0.887712	2.06E-03	-1.46 (0.64)	0.51134
0.8896	0.847336	2.08E-03	-1.86 (0.50)	0.75397
1.1077	1.32113	2.15E-03	-1.57 (0.65)	0.64399
7.21955	1.09641	2.21E-03	-1.56 (0.71)	0.52154
2.0645	0.784504	2.26E-03	-1.43 (0.45)	0.67007
2.7642	1.02394	2.31E-03	-1.41 (0.72)	0.5068
1.61515	0.840357	2.41E-03	1.10 (0.46)	0.60998
6.2109	1.18149	2.43E-03	-1.52 (0.32)	0.59977
6.8933	0.973333	2.45E-03	-1.69 (0.60)	0.5068

2.6236	0.925715	2.53E-03	-1.65 (0.58)	0.51814
3.977	2.02209	2.57E-03	1.23 (0.36)	0.56236
6.67295	0.912933	2.57E-03	-1.78 (0.95)	0.56349
3.1651	0.726378	2.71E-03	-1.57 (0.51)	0.50113
1.9875	0.806205	2.76E-03	-1.49 (0.62)	0.64286
8.2571	0.701427	2.77E-03	-1.62 (0.49)	0.52608
6.7589	0.972247	2.79E-03	-1.68 (0.91)	0.5034
2.0054	0.914327	2.86E-03	-1.52 (0.52)	0.52041
2.6114	0.942089	2.95E-03	-1.44 (0.57)	0.53628
1.8391	0.806525	2.99E-03	-1.41 (0.51)	0.56009
2.8277	0.770898	3.12E-03	-1.39 (0.68)	0.62925
2.9347	0.829522	3.18E-03	-1.61 (0.44)	0.57823
8.74105	1.13658	3.25E-03	-1.85 (0.72)	0.50113
2.28335	0.879505	3.30E-03	-1.63 (0.48)	0.54762
2.8433	0.945086	3.55E-03	-1.38 (0.50)	0.52268
6.20005	0.628541	3.61E-03	-1.87 (0.69)	0.56236
6.5599	0.744647	3.61E-03	-1.99 (0.59)	0.70408
2.7895	0.945721	3.72E-03	-1.43 (0.67)	0.76417
2.2653	0.787531	3.93E-03	-1.51 (0.60)	0.52154
2.8636	0.716573	3.98E-03	-1.45 (0.82)	0.52608
3.1351	0.756326	4.07E-03	-1.51 (0.50)	0.59184
2.49555	0.915406	4.14E-03	-1.50 (0.67)	0.58163
0.8599	0.705644	4.20E-03	-1.52 (0.45)	0.62585
6.96685	0.950932	4.25E-03	-1.52 (0.34)	0.50794
2.50565	0.985425	4.27E-03	-1.46 (0.80)	0.55556
5.77805	0.818453	4.62E-03	-1.44 (0.26)	0.54875
6.70095	0.95202	4.98E-03	-1.89 (1.04)	0.50794
2.0866	0.812571	5.07E-03	-1.44 (0.45)	0.59864
1.40825	0.795218	5.17E-03	-1.37 (0.48)	0.5873
1.3064	0.87048	5.31E-03	-1.72 (0.36)	0.62358
2.7987	1.05604	5.31E-03	-1.34 (0.65)	0.51474
1.73295	0.755122	5.41E-03	-1.42 (0.57)	0.5805
2.2983	0.801149	5.42E-03	-1.67 (0.49)	0.54989
7.7618	1.06174	5.97E-03	-1.60 (0.27)	0.59977
2.0541	0.795807	6.07E-03	-1.36 (0.47)	0.68254
2.4871	0.931448	6.07E-03	-1.70 (0.75)	0.72789
1.0955	1.26725	6.29E-03	-1.43 (0.62)	0.56009
1.1142	0.902884	6.29E-03	-1.39 (0.63)	0.58163
1.69165	0.820032	6.29E-03	-1.39 (0.43)	0.56689

1.12135	1.39459	6.35E-03	-1.50 (0.69)	0.56349
1.70355	0.799226	6.40E-03	-1.36 (0.44)	0.52381
6.9757	0.857789	6.53E-03	-1.53 (1.01)	0.65533
2.4781	0.846631	6.55E-03	-1.56 (0.57)	0.57823
7.75045	1.08561	6.59E-03	-1.48 (0.37)	0.56122
6.5513	0.937193	6.68E-03	-1.81 (0.64)	0.5102
2.22175	0.92899	6.77E-03	-1.37 (0.69)	0.69728
2.77405	0.929386	6.86E-03	-1.42 (0.80)	0.58503
1.5075	0.853717	6.87E-03	-1.43 (0.58)	0.64626
2.2518	0.785688	6.94E-03	-1.41 (0.59)	0.56803
1.7131	0.767389	7.11E-03	-1.37 (0.42)	0.54649
1.5199	0.847569	7.13E-03	-1.43 (0.56)	0.59864
2.48375	0.817402	7.16E-03	-1.53 (0.66)	0.52834
1.2114	0.820352	7.20E-03	-1.47 (0.35)	0.6712
6.9898	0.835118	7.31E-03	-1.44 (0.82)	0.661
0.92035	0.671898	7.45E-03	-1.40 (0.67)	0.63719
6.56905	0.878695	7.57E-03	-1.56 (0.52)	0.51587
0.91645	0.806019	7.59E-03	-1.56 (0.46)	0.57483
1.97095	0.798213	7.91E-03	-1.34 (0.57)	0.54082
1.29495	0.751875	8.08E-03	-1.58 (0.34)	0.63492
6.18955	0.95066	8.19E-03	-1.56 (0.50)	0.64626
0.9109	0.890435	8.43E-03	-1.51 (0.62)	0.58163
2.39325	0.888312	8.53E-03	-1.36 (0.45)	0.61791
2.0803	0.775542	8.74E-03	-1.37 (0.44)	0.52494
7.73885	1.14908	8.82E-03	-1.72 (0.30)	0.53855
8.3453	0.322186	8.82E-03	-1.57 (0.56)	0.51474
1.8753	0.983335	8.83E-03	-1.76 (0.53)	0.65646
1.66505	0.7788	8.87E-03	-1.43 (0.48)	0.52494
1.58045	0.714555	8.93E-03	-1.54 (0.52)	0.65873
2.4713	0.819359	9.21E-03	-1.80 (0.47)	0.52494
0.89385	0.940307	9.42E-03	-1.46 (0.42)	0.72449
0.6823	0.735169	9.61E-03	1.63 (0.46)	0.68481
4.47605	0.936199	9.67E-03	-1.39 (0.57)	0.51587
2.95745	1.17527	9.70E-03	-1.42 (0.34)	0.55782
8.32505	1.07545	9.86E-03	-1.97 (0.91)	0.51247
6.6213	0.833149	1.01E-02	-1.57 (0.77)	0.54422
0.9255	0.954795	1.04E-02	-1.78 (0.64)	0.67687
1.56145	0.830958	1.06E-02	-1.34 (0.37)	0.50113
1.1952	0.811772	1.09E-02	-1.31 (0.36)	0.68254

2.7549	0.84373	1.10E-02	-1.37 (0.62)	0.65193
7.543	1.185	1.11E-02	1.20 (0.44)	0.52834
2.16985	0.82835	1.14E-02	-1.42 (0.48)	0.53628
3.93375	1.82745	1.18E-02	-1.51 (0.50)	0.55782
0.90525	0.88293	1.20E-02	-1.39 (0.44)	0.73696
6.5922	0.750159	1.21E-02	-1.86 (1.31)	0.5102
2.00055	0.82361	1.22E-02	-1.36 (0.58)	0.5771
2.07445	0.805123	1.24E-02	-1.42 (0.60)	0.65533
2.1065	0.796311	1.29E-02	-1.34 (0.43)	0.52834
5.9007	0.455074	1.29E-02	-1.42 (0.34)	0.69048
1.94365	0.835076	1.31E-02	-1.35 (0.51)	0.6644
1.51345	0.833997	1.33E-02	-1.39 (0.47)	0.62245
1.7267	0.825077	1.34E-02	-1.39 (0.50)	0.56576
2.9236	0.767897	1.35E-02	-1.40 (0.91)	0.63039
6.8751	0.984059	1.35E-02	-1.48 (0.90)	0.5034
2.02535	0.821484	1.42E-02	-1.35 (0.58)	0.58503
2.9504	0.850991	1.42E-02	-1.37 (0.33)	0.57937
7.05295	0.904417	1.42E-02	-1.47 (0.64)	0.67234
5.46115	1.78587	1.43E-02	1.22 (0.49)	0.5737
1.4357	0.801023	1.44E-02	-1.35 (0.47)	0.79592
7.37895	0.902902	1.45E-02	-1.34 (0.60)	0.68141
1.2741	0.803866	1.51E-02	-1.36 (0.45)	0.52381
1.50235	0.835114	1.57E-02	-1.37 (0.44)	0.72109
1.13335	1.40148	1.58E-02	-1.43 (0.70)	0.52834
2.012	0.872277	1.58E-02	-1.32 (0.58)	0.5907
1.62835	0.770486	1.64E-02	-1.42 (0.43)	0.54989
0.7203	0.738925	1.65E-02	1.78 (1.00)	0.68141
7.97505	1.83737	1.83E-02	1.22 (0.43)	0.57256
1.49825	0.807787	1.84E-02	-1.45 (0.55)	0.60204
1.10195	0.835416	1.95E-02	-1.34 (0.60)	0.661
2.9177	0.788979	2.01E-02	-1.32 (0.88)	0.61678
2.40915	0.837659	2.02E-02	-1.52 (0.92)	0.61678
7.23205	1.08135	2.04E-02	-1.43 (0.94)	0.67347
0.88535	0.987054	2.10E-02	-1.43 (0.47)	0.73016
4.56465	1.02249	2.31E-02	-1.35 (0.55)	0.55329
1.67635	0.800976	2.42E-02	-1.34 (0.48)	0.54082
8.8998	0.679227	2.42E-02	-1.89 (1.21)	0.52948
5.8894	1.02874	2.54E-02	-1.37 (0.42)	0.52268
7.42135	1.31643	2.75E-02	-1.37 (0.49)	0.64626

1.8857	0.945214	2.79E-02	-1.75 (0.50)	0.57937
4.4566	0.844084	3.04E-02	-1.27 (0.52)	0.52608
4.33855	0.761004	3.06E-02	-1.39 (0.39)	0.55782
8.12295	1.60991	3.08E-02	-1.45 (0.49)	0.50227
8.109	1.15	3.12E-02	-1.43 (0.42)	0.57143
2.7298	1.01497	3.17E-02	-1.31 (0.62)	0.53741
2.8946	1.46488	3.19E-02	-1.55 (1.60)	0.62245
1.2063	0.819508	3.23E-02	-1.27 (0.56)	0.67687
1.2281	0.82427	3.25E-02	-1.25 (0.48)	0.62585
1.4488	0.809158	3.26E-02	-1.29 (0.48)	0.52834
1.23945	0.818726	3.28E-02	-1.48 (0.48)	0.60771
3.03	0.85	3.44E-02	-1.81 (0.56)	0.58617
5.53535	1.61605	3.48E-02	1.15 (0.26)	0.51361
5.83495	0.844498	3.66E-02	1.80 (0.30)	0.73696
1.8635	0.833176	3.72E-02	-1.32 (0.59)	0.51814
7.50285	1.4298	3.74E-02	-1.53 (0.62)	0.61791
6.48405	0.814774	3.75E-02	-1.60 (0.42)	0.72222
1.42825	0.795345	3.77E-02	-1.24 (0.40)	0.68367
1.48515	0.791421	3.89E-02	-1.31 (0.47)	0.51701
7.9916	1.74924	4.01E-02	1.42 (0.75)	0.53855
5.61395	1.03635	4.06E-02	-1.22 (0.31)	0.55556
5.4878	1.20709	4.07E-02	1.59 (0.33)	0.50227
1.45615	0.788121	4.15E-02	-1.27 (0.49)	0.56689
1.38495	0.772866	4.16E-02	-1.27 (0.48)	0.65646
2.20825	0.778053	4.49E-02	-1.35 (0.63)	0.50794
1.22205	0.768472	4.52E-02	-1.30 (0.47)	0.57483
4.4174	0.454957	4.57E-02	-1.64 (0.52)	0.51474
4.3171	0.758641	4.62E-02	-1.24 (0.51)	0.5805
2.23975	0.886702	4.84E-02	-1.29 (0.66)	0.5102
4.28105	0.799397	4.93E-02	-1.29 (0.56)	0.54308

Table C: List of unidentified buckets with p-value < 0.05 and fold change < 2 for 15-month old female serum samples (colored red in the volcano plot).

ppm	VIP	p-value	Fold Change (Error)	AUC
3.076	2.28	1.59E-06	1.67 (0.60)	0.92
4.029	2.23	1.20E-05	1.62 (0.74)	0.91
2.073	1.92	7.24E-05	1.91 (0.75)	0.86

4.069	1.99	1.22E-04	-1.45 (0.20)	0.85
3.064	1.84	2.98E-04	1.54 (0.60)	0.83
2.528	1.82	3.10E-04	1.32 (0.60)	0.85
3.339	1.77	4.40E-04	1.79 (0.80)	0.81
0.166	1.77	8.92E-04	1.69 (0.61)	0.79
5.390	1.67	9.74E-04	1.32 (0.50)	0.82
1.307	1.62	1.58E-03	1.37 (0.48)	0.80
2.701	1.62	1.68E-03	1.29 (0.67)	0.79
3.967	1.62	2.11E-03	-1.43 (0.25)	0.77
2.676	1.56	2.64E-03	1.28 (0.84)	0.77
3.213	1.54	2.64E-03	1.83 (1.00)	0.78
7.101	1.53	3.08E-03	1.47 (0.73)	0.77
4.023	1.54	3.19E-03	1.33 (0.63)	0.75
7.832	1.44	4.94E-03	1.85 (1.22)	0.75
7.838	1.43	6.39E-03	1.79 (1.06)	0.71
7.181	1.38	9.62E-03	-1.37 (0.33)	0.73
3.241	1.40	1.00E-02	-1.57 (0.31)	0.72
0.987	1.35	1.15E-02	-1.27 (0.25)	0.72
1.938	1.56	1.20E-02	-1.74 (0.13)	0.73
7.295	1.37	1.23E-02	1.10 (0.64)	0.73
3.686	1.30	1.37E-02	1.35 (0.68)	0.73
4.019	1.33	1.38E-02	1.58 (1.35)	0.70
4.040	1.29	1.54E-02	1.58 (1.40)	0.69
1.047	1.30	1.67E-02	-1.26 (0.27)	0.72
4.085	1.28	1.68E-02	-1.20 (0.19)	0.74
3.231	1.25	1.83E-02	-1.24 (0.40)	0.73
3.156	1.30	1.98E-02	1.18 (0.52)	0.72
1.923	1.27	2.11E-02	-1.25 (0.16)	0.70
3.011	1.19	2.19E-02	1.43 (0.83)	0.76
1.004	1.29	2.21E-02	-1.31 (0.24)	0.68
2.999	1.18	2.33E-02	1.33 (0.82)	0.68
6.881	1.21	2.56E-02	-1.29 (0.32)	0.70
1.291	1.23	3.27E-02	1.91 (2.61)	0.67
4.225	1.20	3.30E-02	-1.62 (0.70)	0.68
7.554	1.08	3.79E-02	1.48 (1.02)	0.69
2.160	1.28	3.83E-02	-1.36 (0.21)	0.63
3.223	1.17	3.87E-02	1.40 (0.95)	0.67
4.005	1.16	4.06E-02	1.52 (1.51)	0.66
3.196	1.08	4.33E-02	1.36 (1.08)	0.67

3.792	1.07	4.48E-02	-1.17 (0.26)	0.68
2.484	1.14	4.60E-02	-1.31 (0.30)	0.65
3.257	1.13	4.82E-02	-1.41 (0.30)	0.65
2.337	1.15	4.83E-02	1.17 (0.50)	0.71

Table D: List of unidentified buckets with p-value < 0.05 and fold change < 2 for 15-month old female fecal samples (colored red in the volcano plot).

ppm	VIP	p-value	Fold Change (Error)
1.199	2.15	4.03E-07	1.81 (0.73)
1.1772	2.18	1.80E-06	1.85 (0.93)
4.92785	1.51	1.05E-05	1.52 (0.40)
5.72215	1.51	8.65E-05	1.95 (0.42)
4.94005	1.26	1.12E-04	1.48 (0.47)
6.48405	1.20	2.16E-04	1.73 (0.50)
4.6031	1.61	2.37E-04	1.65 (0.41)
0.70765	1.31	2.52E-04	1.89 (0.89)
2.7992	1.16	3.74E-04	1.64 (0.77)
6.19445	1.24	3.79E-04	1.54 (0.66)
5.4592	1.29	4.87E-04	1.57 (0.64)
0.0968	1.27	5.60E-04	1.62 (1.31)
6.6083	1.30	7.64E-04	1.62 (0.69)
5.32385	1.16	7.76E-04	1.67 (0.43)
6.6851	1.11	8.44E-04	1.66 (0.69)
2.65835	1.17	1.01E-03	1.68 (0.50)
1.2617	0.99	1.04E-03	1.37 (0.60)
2.63535	1.02	1.13E-03	1.52 (0.54)
6.66155	1.07	1.13E-03	1.66 (0.67)
6.7913	1.15	1.42E-03	1.53 (0.90)
4.5965	1.19	1.55E-03	1.56 (0.71)
0.1669	1.70	1.55E-03	1.59 (0.53)
2.68665	1.18	1.62E-03	1.59 (0.64)
6.63115	1.08	1.73E-03	1.71 (0.72)
5.12715	1.002	1.89E-03	1.47 (0.41)
2.6722	1.09	2.24E-03	1.55 (0.65)

0.8488	0.91	2.43E-03	1.38 (0.55)
7.02565	1.07	2.51E-03	1.65 (0.62)
0.9438	1.05	2.53E-03	1.49 (0.89)
3.65865	1.19	2.54E-03	1.35 (0.56)
0.8599	0.91	2.71E-03	1.41 (0.60)
8.26295	1.24	2.98E-03	1.46 (0.72)
0.9542	1.10	3.02E-03	1.52 (0.75)
7.2559	0.90	3.05E-03	1.48 (0.77)
2.8286	0.93	3.42E-03	1.44 (0.79)
6.64215	1.08	3.44E-03	1.52 (0.63)
3.97805	0.93	3.47E-03	1.35 (0.92)
5.62805	1.18	3.59E-03	1.39 (0.72)
6.7059	0.93	3.70E-03	1.54 (0.72)
6.9301	1.13	4.06E-03	1.54 (0.65)
1.23805	0.91	4.14E-03	1.35 (0.65)
7.50255	1.14	4.32E-03	1.89 (1.13)
2.7457	0.88	5.07E-03	1.47 (0.41)
7.0381	1.10	5.25E-03	1.57 (0.60)
7.88405	1.14	5.41E-03	1.98 (1.26)
7.41045	0.98	5.56E-03	1.76 (0.60)
0.96455	1.12	5.71E-03	1.48 (0.78)
2.0507	0.87	5.90E-03	1.36 (0.61)
2.7573	1.06	5.95E-03	1.42 (0.60)
1.26735	0.86	6.40E-03	1.29 (0.64)
7.1528	0.86	6.51E-03	1.39 (0.79)
0.78715	0.90	6.90E-03	1.38 (0.90)
7.27155	1.03	8.24E-03	1.46 (0.73)
6.7839	0.997	8.99E-03	1.44 (0.82)
1.0189	0.89	9.17E-03	1.43 (0.77)
6.5849	0.92	9.66E-03	1.89 (0.40)
6.9615	0.84	1.13E-02	1.36 (0.69)
0.8793	0.82	1.24E-02	1.30 (0.52)
1.00775	0.83	1.37E-02	1.41 (0.78)
0.9979	0.78	1.40E-02	1.38 (0.71)
8.2493	1.05	1.40E-02	1.37 (0.95)
7.44465	0.84	1.49E-02	1.38 (1.13)
7.16245	0.81	1.51E-02	1.39 (0.80)
7.28385	0.85	1.57E-02	1.37 (0.93)
3.96825	0.74	1.60E-02	1.32 (0.80)

7.57195	1.03	1.60E-02	1.76 (0.75)
7.33905	1.002	1.65E-02	1.38 (1.17)
0.67345	0.81	1.70E-02	1.88 (1.75)
1.2264	0.82	1.71E-02	1.27 (0.62)
1.20515	0.82	1.74E-02	1.26 (0.66)
2.73755	0.88	1.87E-02	1.37 (0.45)
3.5236	0.84	1.88E-02	1.31 (0.69)
1.8338	0.85	1.90E-02	1.24 (0.55)
0.7991	0.75	1.97E-02	1.35 (0.92)
2.0956	1.04	2.00E-02	1.28 (0.77)
7.2437	0.84	2.11E-02	1.34 (0.90)
4.5514	0.71	2.50E-02	1.32 (0.65)
1.85565	0.79	2.65E-02	1.23 (0.58)
3.5406	0.94	3.04E-02	1.23 (0.70)
2.04475	0.82	3.07E-02	1.25 (0.71)
7.29765	1.03	3.14E-02	1.38 (0.79)
7.17515	0.92	3.20E-02	1.57 (0.42)
5.38935	0.86	3.34E-02	1.45 (0.39)
7.65445	0.86	3.38E-02	1.79 (0.89)
1.2462	0.79	3.45E-02	1.23 (0.69)
3.1103	0.87	3.53E-02	1.36 (0.63)
0.6923	0.73	3.62E-02	1.68 (1.82)
2.5075	0.90	3.69E-02	1.26 (0.63)
5.83255	0.73	3.71E-02	1.59 (1.05)
5.27605	0.81	3.78E-02	1.27 (0.71)
7.1395	0.80	3.88E-02	1.3 (0.82)
7.007	0.94	3.91E-02	1.41 (0.71)
6.7555	0.91	3.98E-02	1.35 (0.80)
2.40895	1.53	4.06E-02	-1.77 (3.37)
2.5232	0.92	4.27E-02	1.49 (0.35)
3.16175	1.05	4.32E-02	1.23 (0.59)
1.05	0.72	4.43E-02	1.24 (0.55)
5.81295	0.85	4.64E-02	1.27 (0.75)
3.58935	0.80	4.83E-02	1.21 (0.54)
5.224	0.93	4.95E-02	1.20 (0.63)

Table E: List of unidentified buckets with p-value < 0.05 and fold change < 2 for 15-month old male fecal samples (colored red in the volcano plot).

ppm	VIP	p-value	Fold Change	AUC

			(Error)	
6.585	2.06	1.76E-04	-1.88 (0.31)	0.86
0.766	1.63	2.17E-04	-1.65 (0.83)	0.79
2.900	1.69	1.02E-03	-1.56 (0.37)	0.77
7.256	1.54	1.24E-03	-1.36 (0.56)	0.77
0.670	1.55	1.42E-03	-1.82 (0.80)	0.80
0.799	1.58	1.45E-03	-1.41 (0.42)	0.75
6.930	1.55	1.79E-03	-1.47 (0.69)	0.77
7.026	1.43	2.11E-03	-1.56 (0.60)	0.79
6.194	1.31	2.47E-03	-1.47 (0.41)	0.76
6.642	1.34	2.91E-03	-1.46 (0.66)	0.74
0.870	1.23	4.40E-03	-1.34 (0.71)	0.76
7.272	1.25	4.52E-03	-1.35 (0.64)	0.74
8.263	1.30	4.53E-03	-1.52 (0.66)	0.73
0.787	1.32	4.92E-03	-1.33 (0.38)	0.73
0.860	1.16	6.18E-03	-1.37 (0.67)	0.74
6.685	1.17	6.98E-03	-1.42 (0.49)	0.71
6.662	1.14	7.02E-03	-1.42 (0.57)	0.73
0.944	1.15	7.44E-03	-1.56 (0.79)	0.78
7.244	1.38	7.71E-03	-1.33 (0.69)	0.73
6.706	1.20	9.04E-03	-1.37 (0.63)	0.70
6.791	1.43	9.12E-03	-1.35 (0.48)	0.70
5.813	1.19	1.04E-02	-1.38 (0.30)	0.71
1.108	1.15	1.32E-02	-1.35 (0.67)	0.69
5.833	1.38	1.41E-02	-1.71 (0.49)	0.81
4.140	1.43	1.43E-02	1.60 (1.99)	0.68
5.722	1.30	1.50E-02	-1.51 (0.61)	0.78
8.429	1.15	1.50E-02	-1.75 (1.42)	0.79
0.849	1.07	1.52E-02	-1.31 (0.62)	0.72
1.095	1.13	1.58E-02	-1.35 (0.67)	0.68
5.324	1.29	1.79E-02	-1.57 (0.26)	0.82
8.249	1.06	1.83E-02	-1.38 (0.76)	0.70
6.631	1.03	1.92E-02	-1.42 (0.61)	0.70
7.298	1.01	2.07E-02	-1.32 (0.43)	0.70
2.076	1.05	2.16E-02	-1.38 (0.57)	0.71
0.954	1.01	2.42E-02	-1.55 (1.03)	0.76
2.738	0.98	2.59E-02	-1.26 (0.42)	0.72
0.965	1.001	2.63E-02	-1.55 (1.06)	0.74
2.758	0.997	2.94E-02	-1.37 (0.57)	0.69

4.940	0.97	2.94E-02	-1.53 (0.21)	0.78
0.998	0.996	2.95E-02	-1.47 (0.64)	0.73
7.153	1.16	3.13E-02	-1.25 (0.68)	0.68
1.121	1.001	3.25E-02	-1.30 (0.64)	0.66
1.267	1.01	3.31E-02	-1.28 (0.52)	0.71
6.608	0.95	3.35E-02	-1.31 (0.63)	0.67
3.022	1.22	3.39E-02	1.73 (4.06)	0.69
2.508	1.04	3.41E-02	-1.27 (0.49)	0.68
1.008	0.99	3.44E-02	-1.51 (0.88)	0.73
6.484	0.93	3.48E-02	-1.28 (0.38)	0.68
7.007	1.11	3.48E-02	-1.35 (0.70)	0.69
7.038	1.06	3.52E-02	-1.33 (0.59)	0.68
2.229	1.14	3.62E-02	1.91 (2.77)	0.65
1.019	0.98	3.80E-02	-1.49 (0.91)	0.72
4.928	0.93	3.96E-02	-1.43 (0.21)	0.76
6.784	1.12	4.18E-02	-1.31 (0.66)	0.66
2.523	0.97	4.28E-02	-1.22 (0.45)	0.67
1.452	0.99	4.35E-02	-1.25 (0.41)	0.68
0.691	1.04	4.74E-02	-1.47 (1.71)	0.79
1.391	1.14	4.87E-02	1.63 (2.32)	0.62
2.746	0.89	4.93E-02	-1.22 (0.57)	0.65
